# The vinylogous Catellani reaction: a combined computational and experimental study†
†Electronic supplementary information (ESI) available. CCDC 1557549. For ESI and crystallographic data in CIF or other electronic format see DOI: 10.1039/c7sc04265e


**DOI:** 10.1039/c7sc04265e

**Published:** 2017-11-30

**Authors:** Y. Yamamoto, T. Murayama, J. Jiang, T. Yasui, M. Shibuya

**Affiliations:** a Department of Basic Medicinal Sciences , Graduate School of Pharmaceutical Sciences , Nagoya University , Chikusa , Nagoya 464-8601 , Japan . Email: yamamoto-yoshi@ps.nagoya-u.ac.jp

## Abstract

In the presence of 5 mol% Pd(OAc)_2_, 1 equiv. of norbornene, and K_2_CO_3_, the reaction of 4-iodo-2-quinolones with tertiary *o*-bromobenzylic alcohols produced the desired benzopyran-fused 2-quinolones in moderate to high yields. A Catellani-type mechanism involving vinylic C–H cleavage is proposed based on the results of control experiments and density functional theory calculations.

## Abstract 2

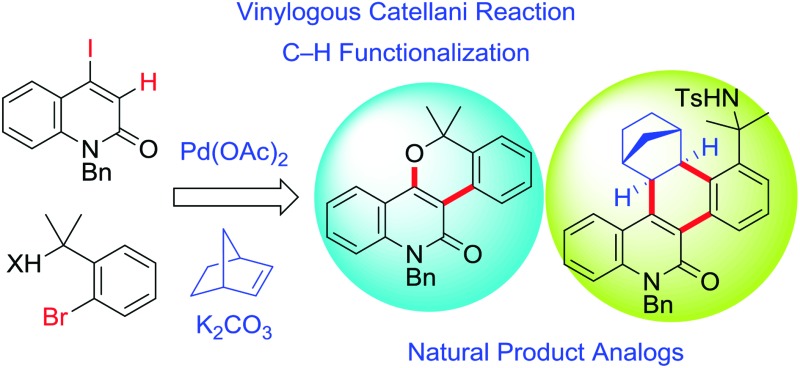
In the presence of 5 mol% Pd(OAc)_2_, 1 equiv. of norbornene, and K_2_CO_3_, the reaction of 4-iodo-2-quinolones with tertiary *o*-bromobenzylic alcohols produced the desired benzopyran-fused 2-quinolones in moderate to high yields.

## Introduction

**Scheme 1:**
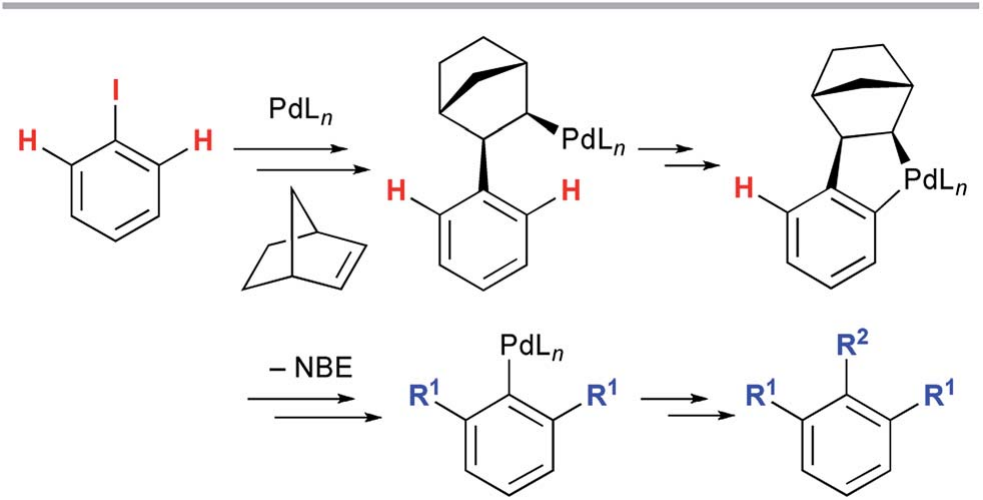
The palladium-catalyzed, norbornene-mediated C–H functionalization of an iodoarene (the Catellani reaction).

The late-stage functionalization of heterocycles by C–H bond activation has an enormous potential for straightforward access to diverse bioactive compounds.^1^ However, this strategy has been applied much less frequently to non-aromatic heterocyclic scaffolds,^2^ because vinylic C–H bonds are generally more challenging to functionalize than (hetero)aromatic C–H bonds.^3^ Therefore, a method to assemble fused heterocyclic structures by vinylic C–H functionalization remains elusive. We focused on the palladium-catalyzed, norbornene (NBE)-mediated C–H functionalization reaction (the Catellani reaction). This is because this reaction allows multiple functionalizations of contiguous sp^2^ carbon centers starting from an iodinated carbon, enabling divergent transformations of relatively simple iodo(hetero)arenes (Scheme 1).^4^ However, the current scope of the Catellani reaction is limited to (hetero)aromatic substrates; no example of a Catellani-type vinylic C–H functionalization has been reported, to the best of our knowledge.^4^

**Scheme 2:**
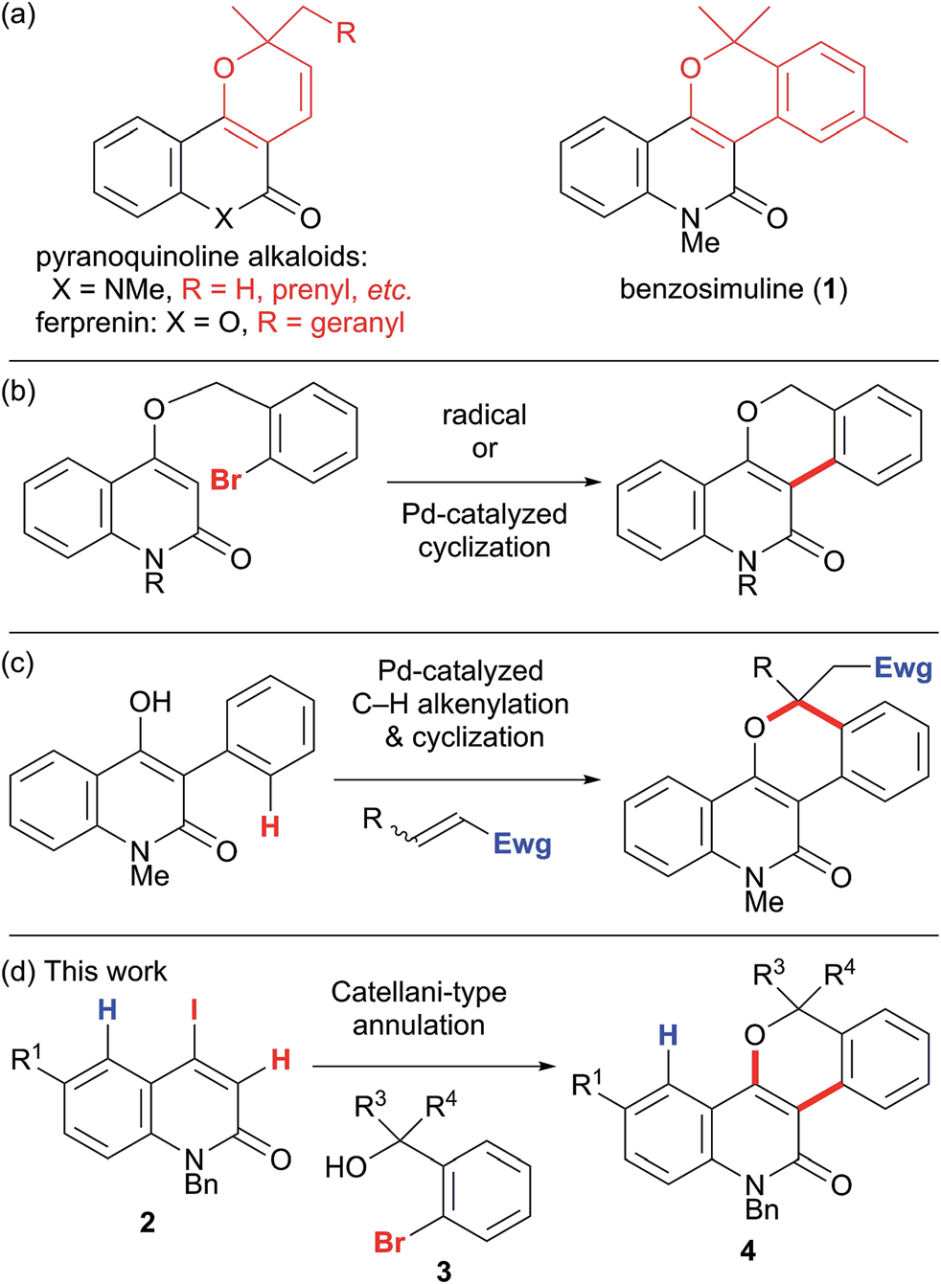
(a) Pyran-fused 2-quinolone scaffolds in nature, (b and c) previous syntheses of benzopyran-fused 2-quinolones, and (d) the synthesis of benzopyran-fused 2-quinolones using the vinylogous Catellani reaction.

Pyran-fused 2-quinolone derivatives are widespread in nature (pyranoquinoline alkaloids), and the construction of this privileged structure has attracted continual attention, owing to the interesting biological activities of these compounds (Scheme 2a).^5^ Ferprenin, a related natural product with the pyranocoumarin framework, was also reported to have interesting biological activity.^6^ In contrast, the synthesis of benzopyran-fused derivatives (*e.g.* the natural product benzosimuline (**1**) which shows antiplatelet aggregation activity)^7^ has been less investigated. Therefore, an efficient synthetic method that enables the divergent synthesis of benzopyran-fused derivatives would be highly beneficial for drug discovery. However, construction of the benzopyran-fused 2-quinolone framework is limited to a few methods. One practical example is the radical or palladium-catalyzed cyclization of 4-(*o*-bromophenyl)methoxy-2-quinolones (Scheme 2b).^8^ This route reliably produces the desired products in high yields. Nevertheless, preparation of the cyclization precursors is necessary and no example with a tertiary benzylic center has been reported. Alternatively, a palladium-catalyzed oxidative annulation of 4-hydroxy-3-phenyl-2-quinolones has recently been developed (Scheme 2c).^9^ This method is remarkable because an aromatic C–H alkenylation is utilized to obviate the use of aryl halides. However, the introduction of the desired aryl group to 4-hydroxyquinolone at the 3-position is required; thus, only a phenyl group has been investigated. Michael acceptors are also required for the final ring-closing step. Therefore, the synthesis of benzosimuline analogs has been severely limited with the existing methods. To address this problem, we envisaged that the Catellani-type annulation of 4-iodo-2-quinolones **2** with *o*-bromobenzyl alcohols **3** would afford the desired benzosimuline derivatives **4** in a modular manner (Scheme 2d). This method has an advantage over previous examples as the combination of 4-iodo-2-quinolines with *o*-bromobenzylic alcohols enables the divergent synthesis of benzosimuline analogs. Herein, we report the results of our study on the vinylogous Catellani reaction of 4-iodo-2-quinolones. To elucidate the reaction mechanism, we also performed density functional theory (DFT) calculations.

## Results and discussion

**Scheme 3:**
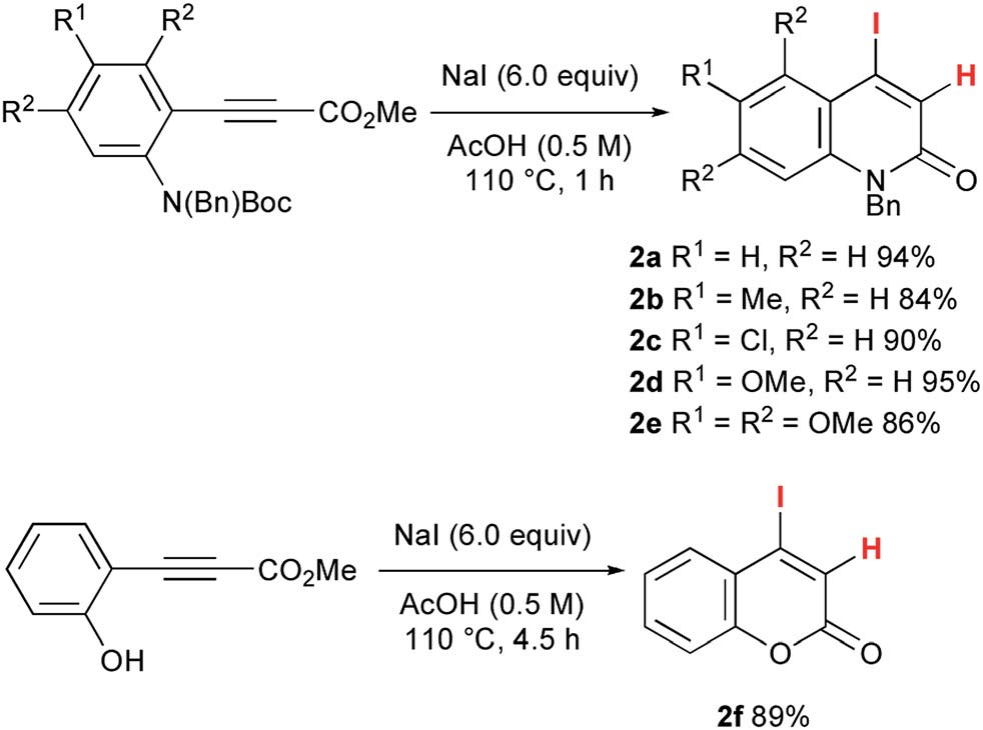
The synthesis of 4-iodo-2-quinolones **2a–e** and 4-iodocoumarin **2f**.

To accomplish the elusive Catellani-type annulation, the use of 4-iodo-2-quinolones, which are expected to be more susceptible to oxidative addition than bromoarenes, is necessary, because the oxidative addition of the 2-quinolone precursors should precede the oxidative addition of *o*-bromobenzyl alcohols. However, hardly any 4-iodo-2-quinolones have been accessible previously, even though diverse 4-bromo-2-quinolones are available. Therefore, this study commenced with the preparation of unknown 4-iodo-2-quinolones **2** (Scheme 3). In a similar manner to the procedure reported by Piers and co-workers,^10^ propiolates with orthogonally protected *o*-aniline terminals, which were previously prepared by our group,^11^ were treated with NaI in AcOH at 110 °C for 1 h. Under the reaction conditions, hydroiodination with concomitant removal of the Boc group was followed by lactam formation to afford the desired 4-iodo-2-quinolones **2a–e** in good yields. Notably, a similar reaction with ethyl 3-phenylpropiolate was reported to give the *anti*-hydroiodination product ((*Z*)-ethyl 3-iodo-3-phenylacrylate).^10^ In this case, the initial *syn*-hydroiodination product presumably underwent isomerization to the observed *anti*-hydroiodination product. In striking contrast, lactam formation proceeded faster than the *E*/*Z* isomerization to afford 4-iodo-2-quinolones **2**. Similarly, 4-iodocoumarin **2f** was prepared from (*o*-hydroxyphenyl)propiolate in 89% yield.

**Scheme 4:**
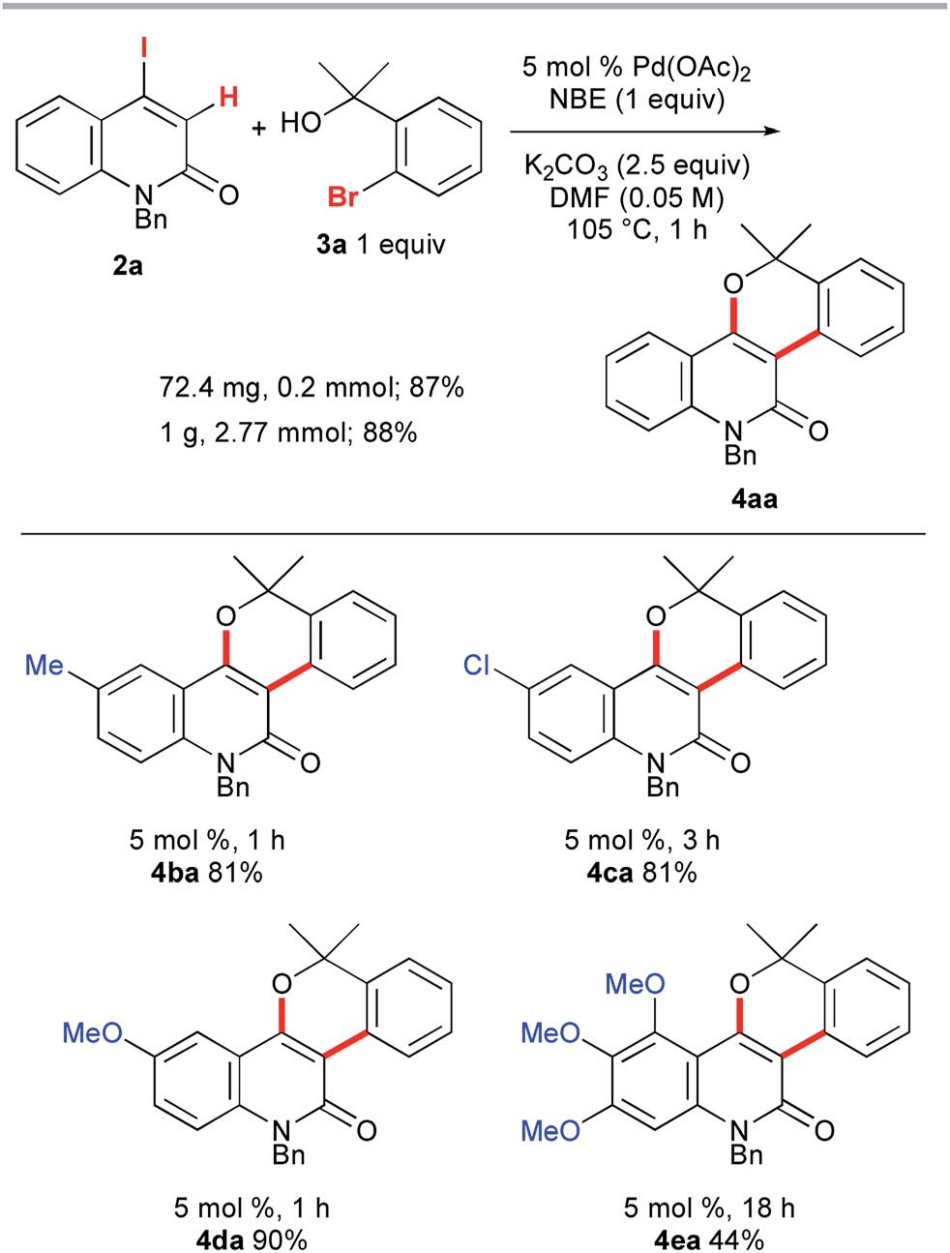
The Catellani-type annulation of 4-iodo-2-quinolones **2a–e** with **3a**.

Once we had obtained precursor **2a**, its Catellani-type annulation with *o*-bromobenzyl alcohol **3a** was investigated (Scheme 4). Catellani and co-workers reported that the reaction of iodobenzenes with *o*-bromobenzylic alcohols bearing no benzylic hydrogen proceeded in the presence of a palladium catalyst and NBE to produce dibenzopyrans.^12^ According to their procedure, **2a** and **3a** were treated with 5 mol% Pd(OAc)_2_, 1 equiv. of NBE, and 2.5 equiv. of K_2_CO_3_ in DMF at 105 °C for 1 h. Gratifyingly, the desired annulation product **4aa** was obtained in 87% yield after purification by silica gel chromatography. The same reaction was conducted using KOAc (2.5 equiv.) instead of K_2_CO_3_, affording **4aa** with a similar efficiency (93% NMR). Therefore, KOAc functions as the net base. In contrast, the use of Cs_2_CO_3_ or Na_2_CO_3_ as the base significantly reduced the efficiency (4% and 29% NMR, respectively), indicating that the potassium ion plays an important role. To examine the scalability, the reaction of **2a** with **3a** was performed at the 1 g scale to obtain **4aa** in 88% yield.

Next, the influence of substituents on the quinolone benzene ring was investigated. The reactions of benzyl alcohol **3a** with 4-iodo-2-quinolones **2b–d**, bearing methyl, chloro, or methoxy substituents at the 6-position, afforded the corresponding annulation products **4ba**, **4ca**, and **4da** in 81–90% yields (Scheme 4). However, the formation of the trimethoxy analog **4ea** was sluggish (18 h) and the yield was moderate (44%). In striking contrast, the reaction of 4-iodocoumarin **2f** under the same conditions led to a complex product mixture, although a modified procedure enabled the desired annulation of **2f** in a high yield (see below).

**Fig. 1:**
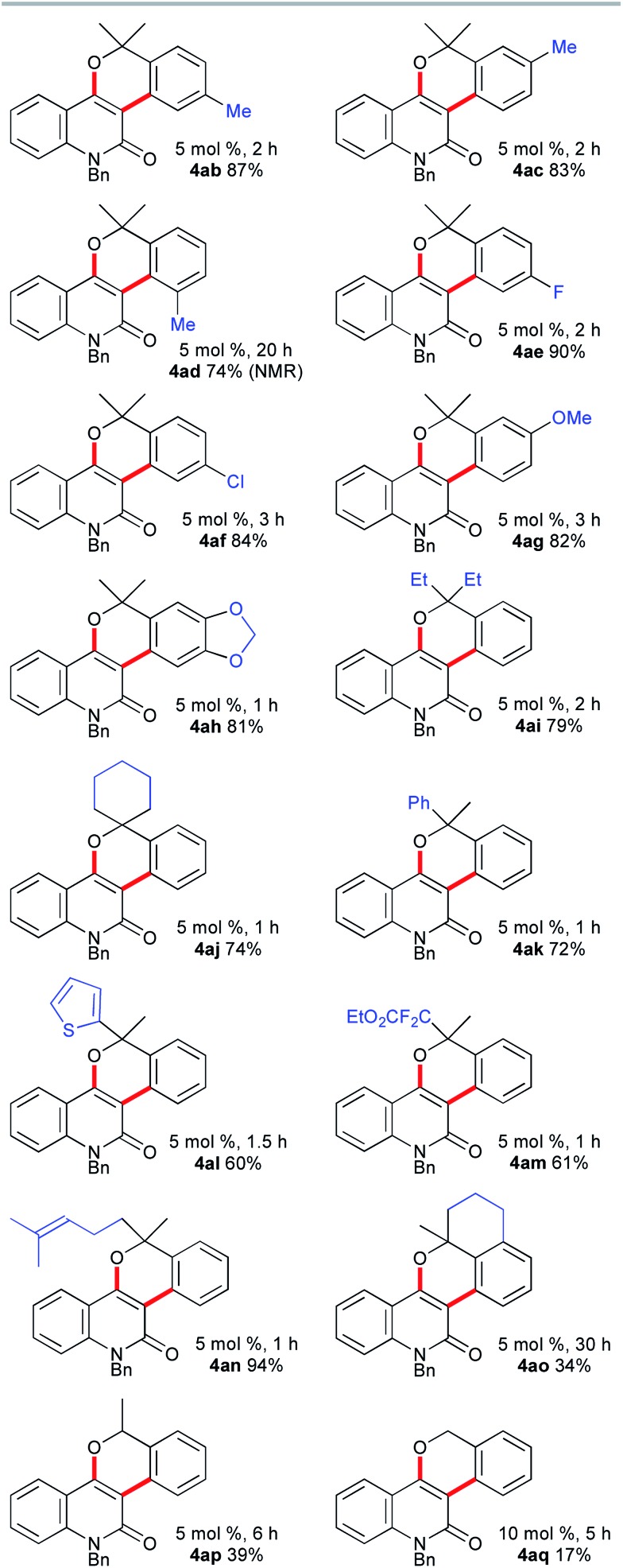
The scope of benzylic alcohols **3**.

The scope of the synthesis of benzopyran-fused 2-quinolones with the vinylogous Catellani reaction was investigated, and the results are summarized in Fig. 1. The use of 4-methyl- and 5-methyl-2-bromobenzyl alcohols **3b** and **3c** under similar conditions afforded **4ab**, which is an *N*-benzyl analog of benzosimuline (**1**), and **4ac** in 87% and 83% yields, respectively. In contrast, the reaction with **3d** was not complete within 20 h, suggesting that the methyl group next to the bromine substituent slowed the oxidative addition and/or subsequent reductive elimination (see below). Other benzyl alcohols, bearing fluoro, chloro, or methoxy substituents, also underwent smooth annulation to afford the corresponding products **4ae–4ag** in high yields. The 1,3-benzodioxole-fused analog **4ah** was obtained in 81% yield. The reaction of **2a** with benzyl alcohols **3i** and **3j** bearing a diethylmethylene and cyclohexyl moiety, respectively, successfully afforded **4ai** and spirocycle **4aj** in 79% and 74% yields. Alcohols **3k–n** substituted unsymmetrically at the benzylic position were used, producing **4ak**, **4al**, **4am** and **4an** in 60–94% yields. Thus, 2-thienyl, α,α-difluoro ester, and trisubstituted alkene moieties were well tolerated. Although the reaction of the fused benzyl alcohol **3o** was very sluggish and required a prolonged reaction time, an interesting polyfused derivative **4ao** was obtained in 34% yield. In this case, a small amount of the three-component annulation product was also formed along with unidentified byproducts (see below). Moreover, the reactions of **2a** with secondary and primary benzyl alcohols **3p** and **3q** afforded **4ap** and **4aq** in much lower yields than that of **4aa**. These results suggest that tertiary benzyl alcohols are essential for high-yield formation of the chromeno-2-quinolones because primary and secondary benzyl alcohols undergo oxidation under the reaction conditions, as reported by Catellani and coworkers.^12^

**Scheme 5:**
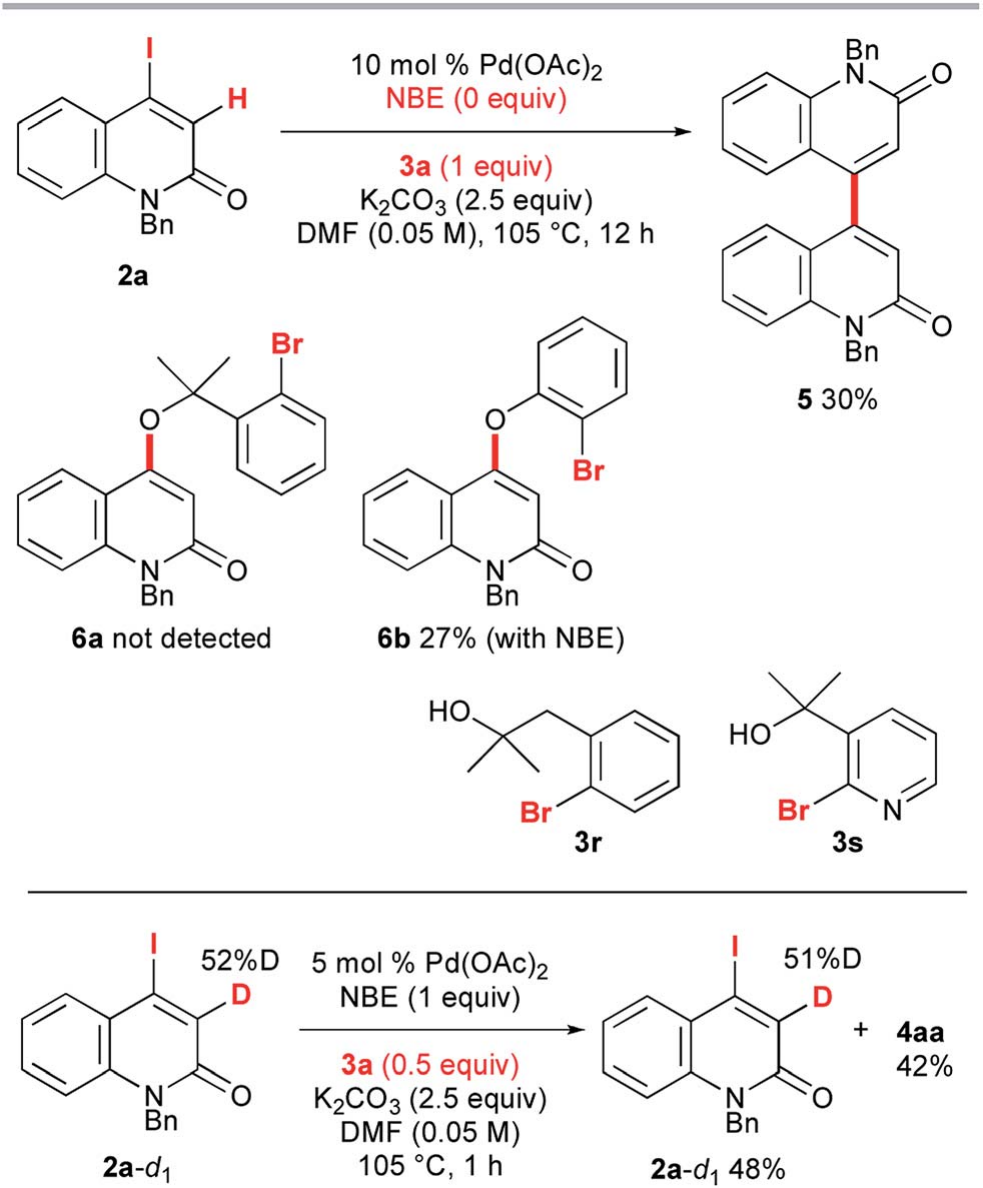
Control experiments.

To gain insight into the reaction mechanism, several control experiments were performed (Scheme 5). First, NBE proved to be indispensable for the reaction: in its absence, **2a** and **3a** were treated with 10 mol% Pd(OAc)_2_ and 2.5 equiv. of K_2_CO_3_ in DMF at 105 °C for 12 h, but no trace of the annulation product was observed. Instead, the homocoupling product **5** was obtained in 30% yield. The C–O coupling product **6a** could not be detected. When *o*-bromophenol was used instead of **3a**, 4-aryloxy-2-quinolone **6b** was obtained in 27% yield, even when 1 equiv. of NBE was added under the same conditions. Presumably, the acidic proton of *o*-bromophenol was abstracted by K_2_CO_3_ and the conjugate addition of the resultant aryloxide was followed by removal of iodide to produce **6b**. These results suggest that the intramolecular Heck pathway *via* the initial formation of **6a** and **6b** can be disregarded. No reaction occurred when the homobenzylic alcohol **3r** and pyridyl derivative **3s** were used. The reaction of 3-deutero-2-quinolone **2a**-*d*_1_ with 52% deuterium content was performed with 0.5 equiv. of **3a** to investigate the kinetic isotope effect. The ^1^H NMR spectroscopic analysis of recovered **2a**-*d*_1_ showed that the deuterium content remained almost the same as that of the starting material **2a**-*d*_1_. Therefore, the reaction rates for **2a** and **2a**-*d*_1_ are almost the same (*k*_H_/*k*_D_ ≈ 1) and it can be concluded that the vinylic C–H cleavage is not the rate-determining step.^13^

**Scheme 6:**
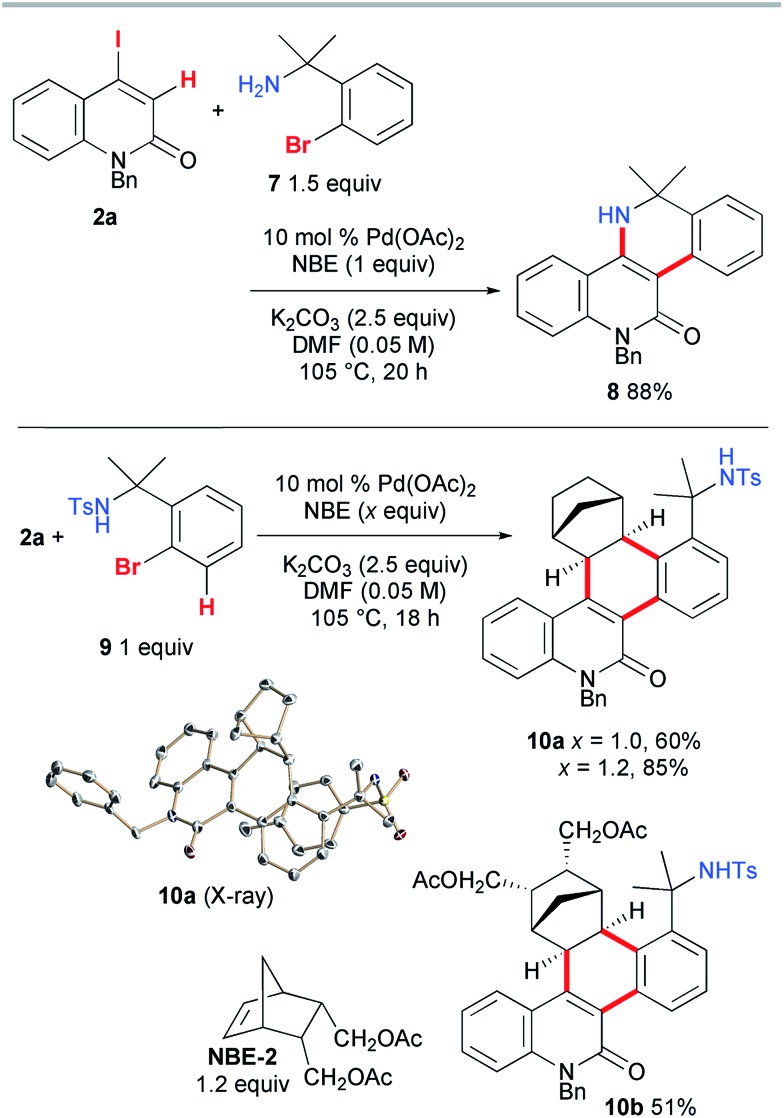
Vinylic Catellani-type reactions involving benzylamines.

Next, the vinylogous Catellani reaction was performed with benzylamines (Scheme 6). Under similar conditions, except for an increased loading of Pd(OAc)_2_ (10 mol%), **2a** and 1.5 equiv. of **7** were heated for 20 h, affording the desired 5,6-dihydrodibenzo[*c*,*h*][1,6]naphthyridin-11(12*H*)-one **8** in 88% yield. In contrast, the use of tosylamide **9** instead of **7** led to the interrupted Catellani product **10a** in 60% yield. A good quality single crystal was obtained, so the structure of **10a** was unambiguously confirmed by X-ray diffraction analysis. As expected, NBE reacted from its *exo* face, and the C–H bond at the 3-position of **9** was involved in the reaction instead of the amide moiety. This indicates that, as proposed for other Catellani reactions, carbopalladation of NBE indeed occurred, and in addition to the vinylic C–H bond of **2a**, the aromatic C–H bond of **9** was cleaved, leading to dehydrogenative coupling.^14^ Because one equivalent of NBE was required for the formation of **10a**, the increased loading of NBE (1.2 equiv.) improved the yield to 85%. When a substituted NBE derivative (**NBE-2**) was used, the corresponding three-component annulation product **10b** was obtained in a moderate yield (51%), indicating that the substitution of NBE has a negative effect on the reaction efficiency.

To gain insight into the fundamental steps of the vinylogous Catellani reaction, DFT calculations were performed at the PCM (DMF) rM06L/6-311G++(d,p)-SDD//rB3LYP/6-31G(d)-LanL2DZ level of theory (for details, see ESI†). This method has been reliably used for the computational analysis of carboxylate-assisted C–H activations.^15^ For computational efficiency, the *N*-benzyl groups of the real molecules were replaced by smaller methyl groups in the model molecules. In addition, the primary benzyl alcohol **3q** was used instead of the tertiary benzyl alcohol **3a** for the model reactions, because the inefficiency of **3q** can be attributed to its susceptibility to oxidation under the experimental conditions.^12^ Calculations on simple ligand exchanges were omitted because they are facile and reversible.

**Scheme 7:**
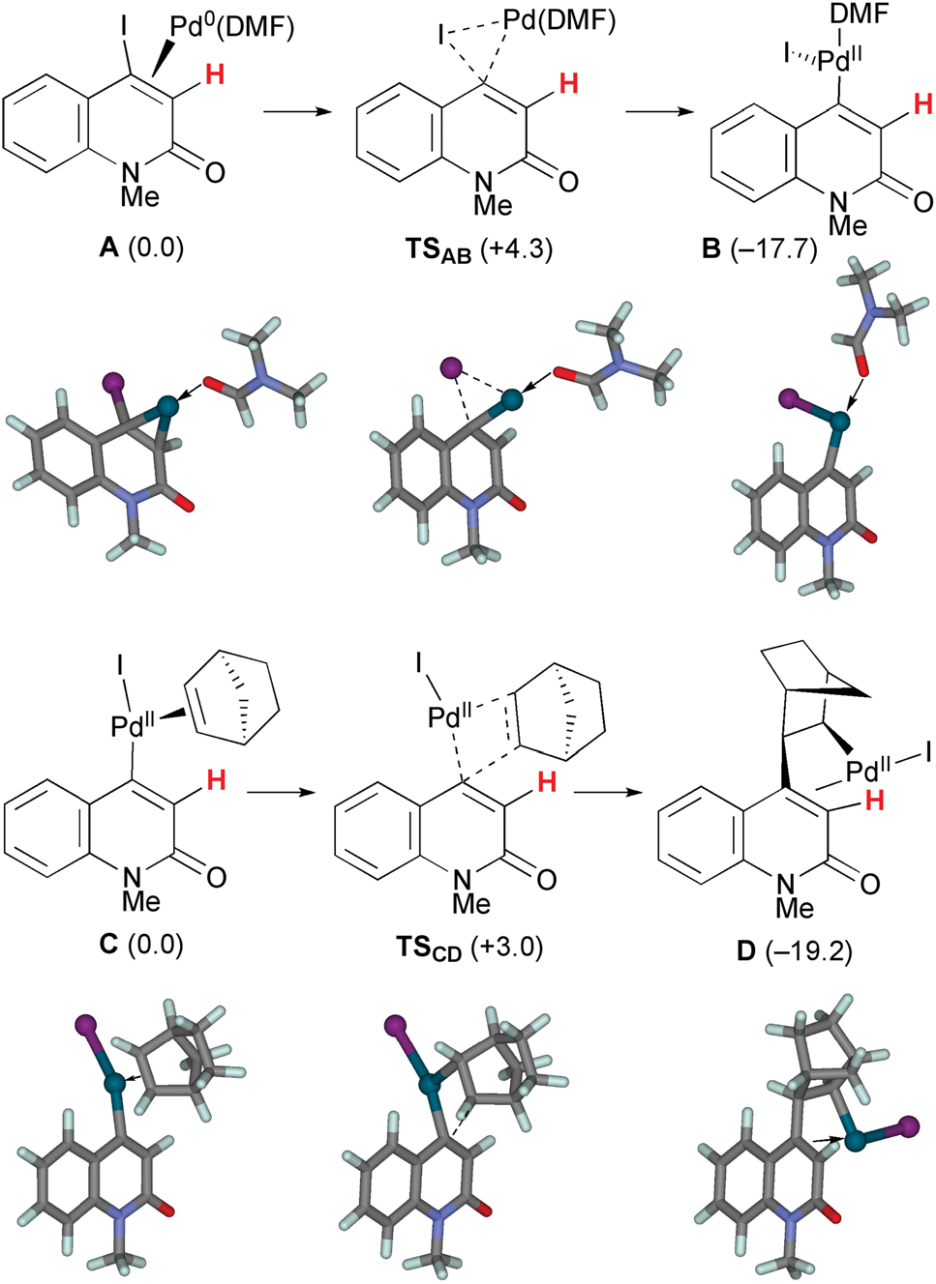
The oxidative addition and NBE insertion steps of model complexes **A** and **C** leading to complexes **B** and **D**, respectively. The relative Gibbs free energies in DMF at 298 K and 1 atm are indicated in parentheses.

The first oxidative addition step starts with the 4-iodo-2-quinolone Pd(0) complex **A** with a DMF ligand (Scheme 7). Under ligandless conditions, the abundant solvent molecule, DMF, should act as the supporting ligand for the Pd(0) species. *o*-Bromobenzyl alcohol **3q** can be a bidentate ligand; however, the smaller and more electron-donating DMF ligand more efficiently promotes oxidative addition.^16^ The Pd(0)(DMF) fragment was coordinated by the 2-quinolone at the electron-deficient alkene moiety. Oxidative addition proceeded *via***TS_AB_** with a small activation energy of Δ*G*^‡^ = +4.3 kcal mol^–1^, affording the Pd(ii) complex **B** which has a T-shape geometry with the 2-quinolone and iodide ligands being mutually *trans*. The formation of **B** is exergonic by 17.7 kcal mol^–1^. The DMF ligand was replaced by NBE to generate complex **C**, in which the 2-quinoline and iodide ligands occupy mutually *trans* positions. The subsequent insertion of NBE proceeded *via***TS_CD_** with a small activation energy of Δ*G*^‡^ = +3.0 kcal mol^–1^, affording the Pd(ii) alkyl complex **D** coordinated by the electron-deficient alkene moiety of the 2-quinolone. The iodide ligand occupies the *trans* position of the alkene ligand. The formation of **D** is exergonic by 19.2 kcal mol^–1^. Therefore, these two steps are both kinetically and thermodynamically feasible.

**Scheme 8:**
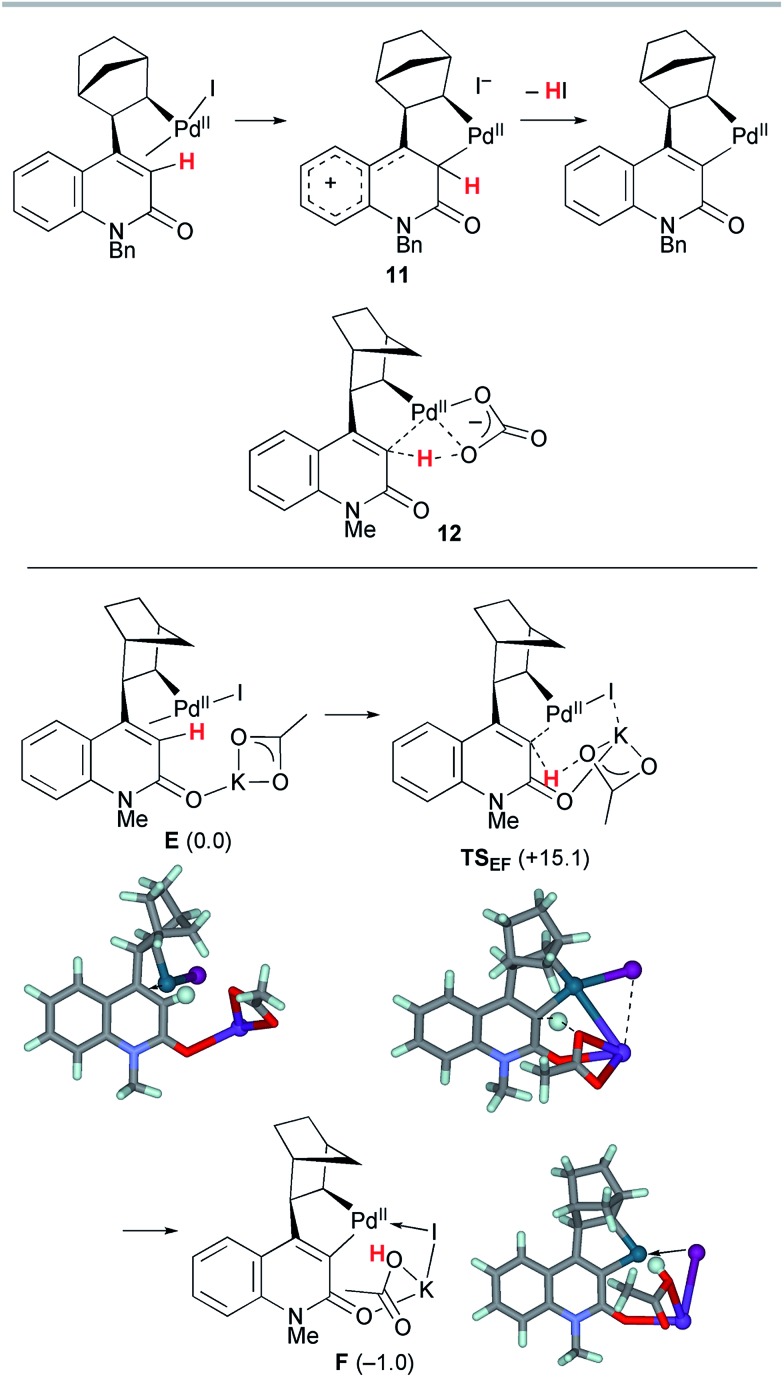
The CMD step of model complex **E** leading to palladacycle complex **F**. The relative Gibbs free energies in DMF at 298 K and 1 atm are indicated in parentheses.

Next, the palladacycle formation was investigated (Scheme 8). As previously proposed,4*a* electrophilic palladation *via* the cationic intermediate **11** is operative for the 2-quinolone system; however, such a transition state (TS) could not be located for a model complex. Therefore, concerted metallation–deprotonation (CMD) mechanisms were considered.^17^,^18^ Because the activation energy for intramolecular proton abstraction by a κ^2^-carbonate ligand on the Pd(ii) center (**12**) was estimated to be as large as 50 kcal mol^–1^ (Scheme S1, ESI†), an alternative TS for intermolecular proton abstraction by an external base was examined. Such a CMD of complex **E** with KOAc as a base proceeded *via***TS_EF_** with a reasonable activation energy of Δ*G*^‡^ = +15.1 kcal mol^–1^. In this TS, the cleaved C–H and formed O_acetate_–H bond distances are 1.346 and 1.318 Å, respectively, while the Pd–C_quinolone_ distance is 2.103 Å. The potassium ion has interactions with the Br center and carbonyl group, as the K–I and K–O_carbonyl_ distances are 3.772 and 2.789 Å, respectively. These values are markedly smaller than the sums of the corresponding van der Waals radii (K/I 4.73 Å, K/O 4.27 Å). Therefore, the amide group plays the important role of a directing group that places KOAc in close proximity to the vinylic proton. The formation of palladacycle complex **F** with the concomitant extrusion of the iodide ligand is slightly endergonic by 1 kcal mol^–1^.

**Scheme 9:**
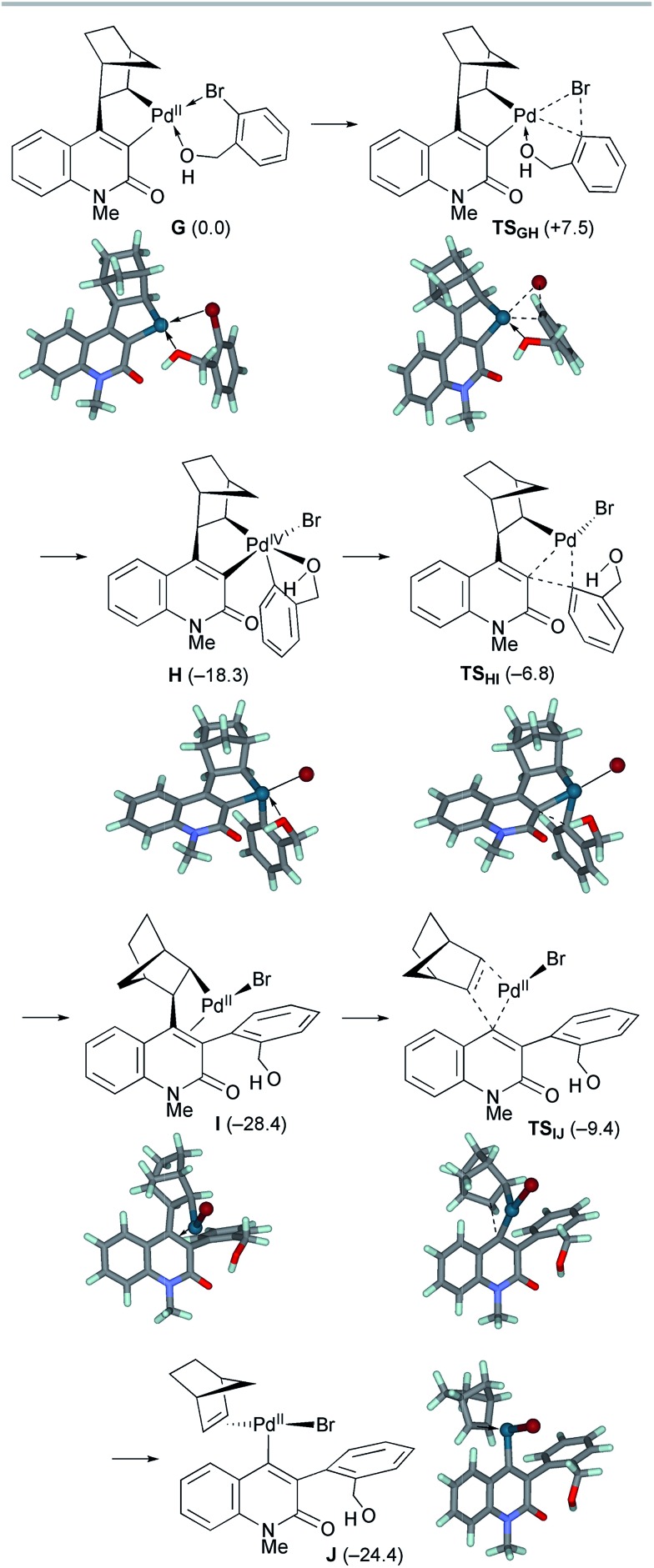
The oxidative addition/reductive elimination steps of model complex **G** and subsequent deinsertion of NBE from intermediate complex **I**. The relative Gibbs free energies in DMF at 298 K and 1 atm are indicated in parentheses.

The second oxidative addition of *o*-bromobenzyl alcohol started from palladacycle complex **G** (Scheme 9). This step proceeded *via***TS_GH_** with a small activation energy of Δ*G*^‡^ = +7.5 kcal mol^–1^, generating the Pd(iv) complex **H** with an exergonicity of 18.3 kcal mol^–1^. Because the five-coordinated complex **H** has a square pyramidal geometry with the *o*-(hydroxymethyl)phenyl ligand at the apical position, the subsequent reductive elimination facilely proceeded *via***TS_HI_** with an activation energy of Δ*G*^‡^ = +11.5 kcal mol^–1^. As a result, the Pd(ii) complex **I** with a 3-arylquinolone ligand was formed with an exergonicity of 10.1 kcal mol^–1^. Subsequently, deinsertion of NBE from **I** proceeded *via***TS_IJ_** with an activation energy of Δ*G*^‡^ = +19.0 kcal mol^–1^, generating the vinyl Pd(ii) complex **J** with a slight endergonicity of 4.0 kcal mol^–1^. Thus, the overall process from **G** to **J** is efficient and highly exergonic by 24.4 kcal mol^–1^.

**Scheme 10:**
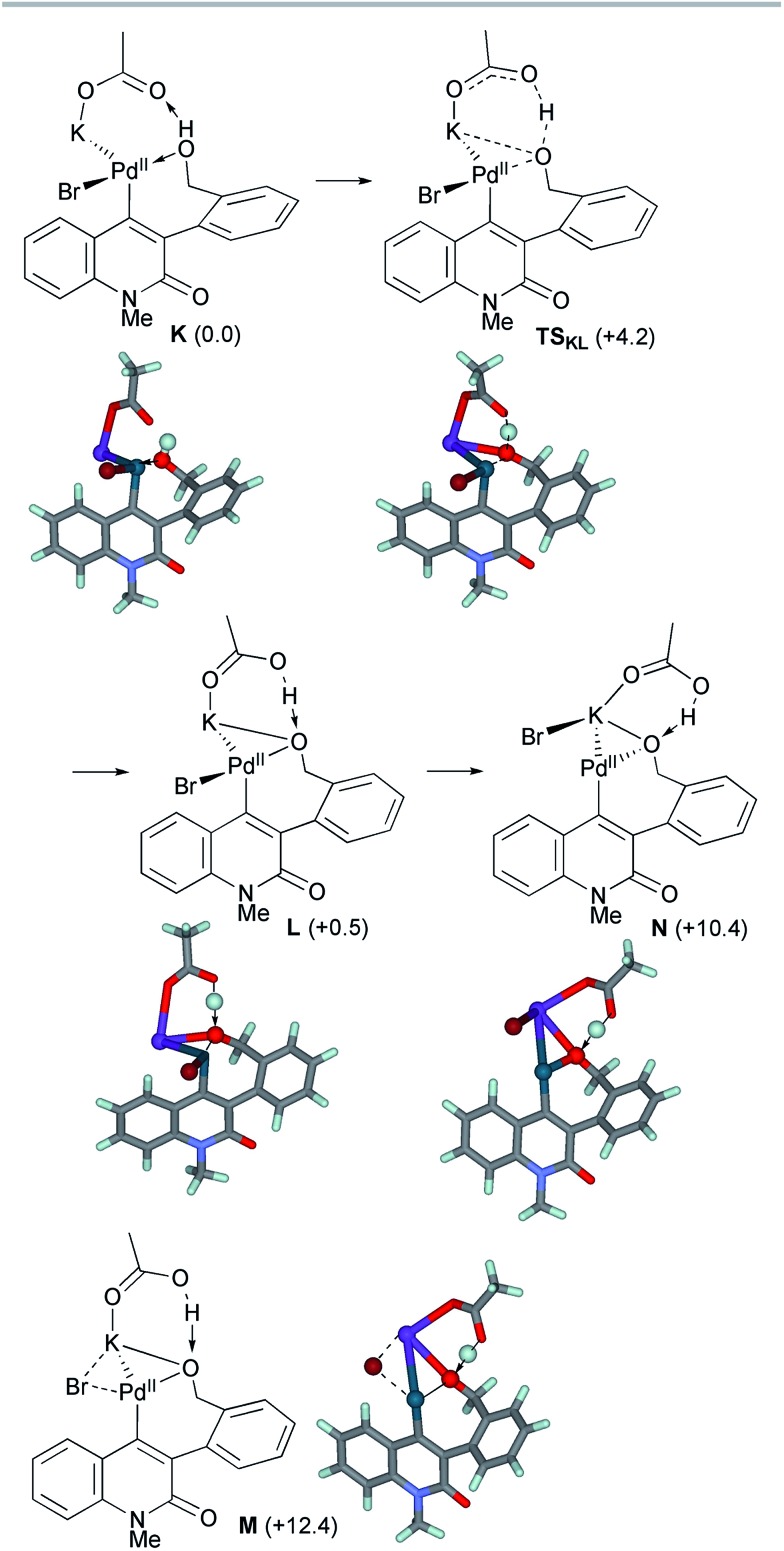
The deprotonation of the benzylic alcohol of model complex **K** and subsequent bromide dissociation from the intermediate complex **L**. The relative Gibbs free energies in DMF at 298 K and 1 atm are indicated in parentheses.

The ligand exchange between the labile NBE ligand and the benzylic hydroxyl group was followed by the association of KOAc to generate intermediate **K** (Scheme 10). Facile deprotonation of the benzylic hydroxyl group proceeded *via***TS_KL_** with a small activation energy of Δ*G*^‡^ = +4.2 kcal mol^–1^, generating the alkoxo complex **L** with a slight endergonicity of 0.5 kcal mol^–1^. The Pd–O_benzyl_ distance was shortened from 2.245 Å in **K** to 2.081 Å in **L**, while the Pd–Br bond length remained almost constant (2.492–2.503 Å). Subsequent dissociation of the bromide from the palladium center ultimately produced alkoxo complex **N**. We could not find the TS for this step, but instead, the TS-like stationary point **M** with one small imaginary frequency (18.5i cm^–1^) was located 11.9 kcal mol^–1^ above **L**. At this point, the bromide ion is placed at the bridging position between the Pd and K centers (Pd–Br 2.637 Å and K–Br 3.382 Å). In the resulting alkoxo complex **N**, the Pd–O_benzyl_ distance was shortened to 2.054 Å. The formation of **N** from **K** was endergonic by 10.4 kcal mol^–1^.

**Scheme 11:**
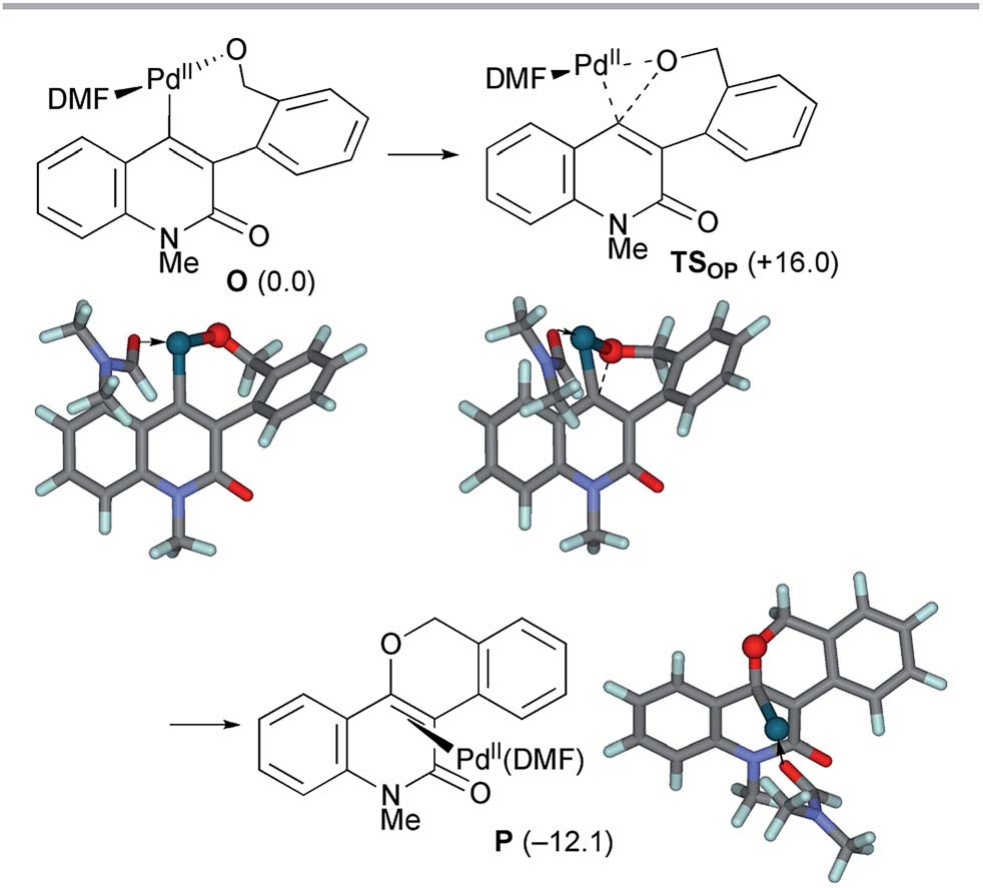
The reductive elimination from model complex **O** affording the final product complex **P**. The relative Gibbs free energies in DMF at 298 K and 1 atm are indicated in parentheses.

After the dissociation of AcOH·KBr, the C–O bond-forming reductive elimination proceeded from the alkoxo complex **O***via***TS_OP_** with an activation energy of Δ*G*^‡^ = +16.0 kcal mol^–1^ (Scheme 11). The C_alkene_–O_benzyl_ distance decreased from 2.878 Å in **O** to 1.947 Å in **TS_OP_**. The formation of the benzopyranyl-2-quinolone complex **P** from **O** is exergonic by 12.1 kcal mol^–1^. Therefore, the final step is both kinetically and thermodynamically feasible.

**Fig. 2:**
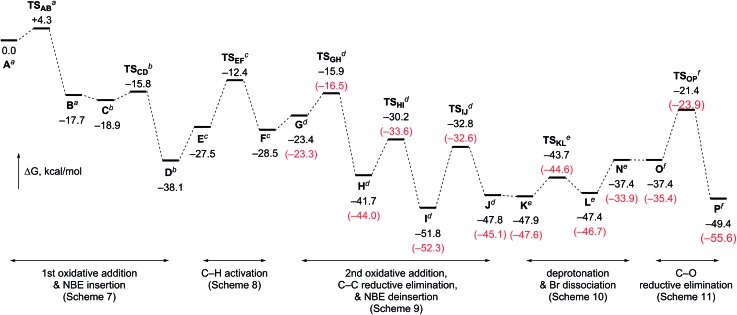
The calculated energy surface for the overall process, with relative Gibbs free energies in DMF at 298 K and 1 atm. The relative Gibbs free energies include those of ^*a*^NBE/2KOAc/*o*-bromobenzyl alcohol, ^*b*^DMF/2KOAc/*o*-bromobenzyl alcohol, ^*c*^DMF/KOAc/*o*-bromobenzyl alcohol, ^*d*^DMF/KOAc/AcOH·KI, ^*e*^DMF/NBE/AcOH·KI, and ^*f*^NBE/KOAc·KI/AcOH·KBr. The relative Gibbs free energies for the reactions associated with **1a** are shown in parentheses (red).

Fig. 2 outlines the energy surface of the overall process. A reasonable amount of the largest energy span^19^ (30.4 kcal mol^–1^) is estimated for steps between **I** and **TS_OP_**, and thus, the later, C–O bond-forming cyclization stage is the rate limiting process. This analysis is in good accordance with the fact that no kinetic isotope effect was observed in the deuterium-labeling experiment shown in Scheme 5. The overall transformation of the starting complex **A** into the final product complex **P** is exergonic by 49.4 kcal mol^–1^, and thus, the vinylogous Cattelani reaction of 4-iodo-2-quinolone is thermodynamically favorable.

The DFT calculations were also performed for the tertiary benzylic alcohol **3a**, which experimentally proved to be more efficient compared to primary or secondary benzylic alcohols. Therefore, very similar results were obtained for the steps involving benzylic alcohols (Fig. 1 and Schemes S2–S4, ESI†). Notably, the largest energy span ΔΔ*G*(**I**–**TS_OP_**) was found to be 2 kcal mol^–1^ smaller for the reaction with **3a** than for that with **3q**. This result is in good agreement with the experimental results.

**Scheme 12:**
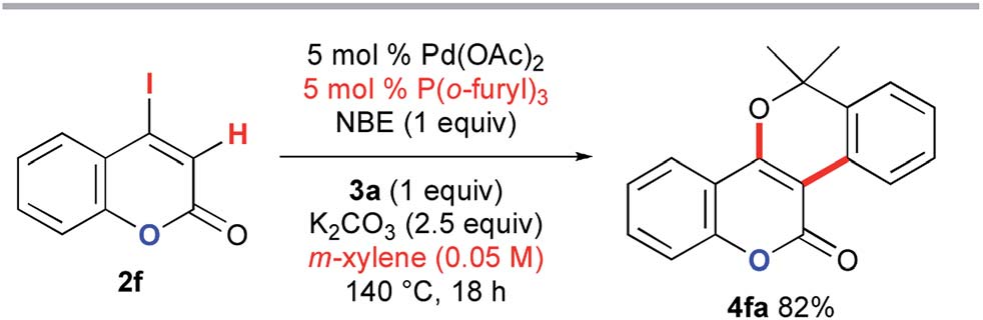
The Catellani-type annulation of 4-iodocoumarin **2f** with **3a**.

The above DFT study implies that the lactam carbonyl oxygen directs KOAc in close proximity to the abstracted hydrogen atom in the CMD step. Therefore, the inefficiency of the coumarin substrate **2f** can be attributed to the weaker coordination ability of its lactone carbonyl group than that of the DMF solvent. To enable the desired annulation with *o*-bromobenzyl alcohol **3a**, modified reaction conditions were investigated using P(*o*-furyl)_3_ as the supporting ligand in xylene as a non-coordinating solvent (Scheme 12). Although a higher reaction temperature and a prolonged reaction time were required, the desired benzopyran-fused coumarin **4fa** was obtained in 82% yield.

## Conclusions

In conclusion, we successfully realized the first vinylogous Catellani reaction of 4-iodo-2-quinolones with benzyl alcohols bearing a tertiary benzyl moiety. By using this method, a variety of chromeno-2-quinolones, which are analogs of the natural alkaloid benzosimuline, were synthesized in moderate to high yields. Moreover, the reactions with the corresponding benzylamine and its tosylamide produced a dibenzonaphthyridin-11(12*H*)-one derivative and a three-component annulation product involving norbornene, respectively. The results of the control experiments corroborate the Catellani-type mechanism *via* norbornene-assisted vinylic C–H functionalization. Moreover, DFT calculations of model complexes suggest that the C–H bond fission proceeds *via* a CMD mechanism and the later C–O bond-forming cyclization stage is the rate-limiting process.

## Conflicts of interest

There are no conflicts to declare.

## Supplementary Material

Supplementary information

Crystal structure data
