# Supplementary material for: The vinylogous Catellani reaction: a combined computational and experimental study
Source: Chem Sci. 2017 Nov 30;9(5):1191–9. doi: 10.1039/c7sc04265e (PMC5885779; doi:10.1039/c7sc04265e)
Supplement: Supplementary file 1 [file SC-009-C7SC04265E-s001.pdf]

Electronic Supplementary Material (ESI) for Chemical Science.
This journal is © The Royal Society of Chemistry 2017

The vinylogous Catellani reaction: A combined computational and experimental study

Yoshihiko Yamamoto,* Tsukasa Murayama, Jiyue Jiang, Takeshi Yasui and Masatoshi Shibuya

Department of Basic Medicinal Sciences, Graduate School of Pharmaceutical Sciences, Nagoya University, Chikusa, Nagoya 464-8601

yamamoto-yoshi@ps.nagoya-u.ac.jp

Supporting Information

1. General Considerations S2
2. Preparation of Benzylic Alcohol and Sulfonamide S2
3. Preparation of 4-Iodo-2-quinolones S4
4. Vinylogous Catellani Reactions S7
5. Control Experiments S15
6. Single Crystal X-ray Diffraction Study S16
7. DFT Calculations S18
8. NMR Charts S81

1. General considerations

Column chromatography was performed on silica gel (Cica silica gel 60N) with solvents specified below. ^{1}H and ^{13}C NMR spectra were obtained for samples in $CDCl_3$ solutions at 25 °C. ^{1}H NMR chemical shifts are reported in terms of chemical shift (δ, ppm) relative to the singlet at 7.26 ppm for chloroform. Splitting patterns are designated as follows: s, singlet; d, doublet; t, triplet; q, quartet; quint, quintet; sext, sextet; sept, septet; m, multiplet. Coupling constants are reported in Hz. ^{13}C NMR spectra were fully decoupled and are reported in terms of chemical shift (δ, ppm) relative to the triplet at δ 77.0 ppm for $CDCl_3$. Reagents and dry solvents were purchased and used as received. *N*-protected (*o*-aminophenyl)propiolates were reported in our previous paper.[1] Benzylic alcohols **3a**,[2] **3b**,[3] **3c**,[3] **3e**,[4] **3g**,[2] **3h**,[2] **3i**,[2] **3j**,[5] **3k**,[6] **3l**,[6] **3p**,[7] **3r**,[8] **3s**,[9] and benzylic amines **7**,[10] were synthesized according to literature

2. Preparation of benzylic alcohols and sulfonamide

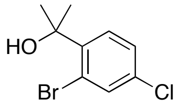

3f

Synthesis of benzylic alcohol 3f: To a solution of methyl 2-bromo-4-chlorobenzoate (461.9 mg, 1.85 mmol) in Et_2O (10.0 mL) was added MeMgI (3.0 M in Et_2O, 2.0 mL, 6.0 mmol) at 0 °C. The reaction mixture was stirred for 12 h at room temperature. The reaction was quenched with sat. NH_4Cl (10 mL) at 0 °C and the whole mixture was extracted with AcOEt (3 × 20 mL). The combined organic layer was washed with brine (10 mL) and dried over Mg_2SO_4. After concentration in vacuo, the crude material was purified by flash column chromatography on silica gel (hexane/EtOAc = 20:1) to afford **3f** (308.3 mg, 86%) as a colorless oil; ^{1}H NMR (400 MHz, $CDCl_3$, 25 °C) δ 7.64 (d, J = 8.8 Hz, 1 H), 7.59 (d, J = 1.6 Hz, 1 H), 7.27 (dd, J = 8.8, 1.6 Hz, 1 H), 2.51 (s, 1 H), 1.73, (s, 6 H); ^{13}C NMR (100 MHz, $CDCl_3$, 25 °C) δ 144.8, 134.3, 133.1, 128.1, 127.4, 120.5, 73.3, 29.3; IR (neat) 3410 (O–H) cm^{-1}; HRMS (DART) *m*/*z* calcd for C_9H_9BrCl 230.9576, found 230.9573 $[M–OH]^+$.

---

[1] T. Murayama, M. Shibuya, Y. Yamamoto, *Adv. Synth. Catal.* **2016**, *358*, 166-171.
[2] L. Mahendar, J. Krishna, A. G. K. Reddy, B. V. Ramulu, G. Satyanarayana, *Org. Lett.* **2012**, *14*, 628-631.
[3] A. Tsutomu, B. T. William, D. J. Marie, P. Jacob J.; W. William Hunter, W. J. Raymond; Z. Yong-Kang, Z. Yasheen, Anacor Pharmaceuticals, Inc., USA; Eli Lilly and Company, US Patent 20130131017 A1
[4] G. Tomasz, H. Robert, H. Scott, E. Brian, R. Olga, Rempex Pharmaceuticals, Inc., USA, WO 2012109164 A1
[5] H. Kinoshita, K. Yaguchi, T. Tphjima, N. Hirai, K. Miura, *Tetrahedron Lett.* **2016**, *57*, 2039-2043.
[6] L. Mahendar, G. Satyanarayana, *J. Org. Chem.* **2014**, *79*, 2059-2074.
[7] B. Schulte, R. Fröhlich, A. Studer, *Tetrahedron* **2008**, *64*, 11852-11859.
[8] A. Raskosova, R. Stößer, W. Abraham, *Chem. Commun.* **2013**, *49*, 3964-3966.
[9] A. I. Subota, O. O. Grygorenko, Y. B. Valter, M. A. Tairov, O. S. Artamonov, D. M. Volochnyuk, S. V. Ryabukhin, *Synlett* **2015**, *26*, 408-411.
[10] I. Erdelmeier, C. Tailhan-Lomont, J.-C. Yadan, *J. Org. Chem.* **2000**, *65*, 8152-8157.

Similarly, benzylic alcohol **3d** was prepared from the corresponding ester.

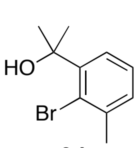

3d

Analytical data for 3d: 554 mg, 55%; white solid (m.p. 60.1–61.9 °C); ^{1}H NMR (400 MHz, $CDCl_3$, 25 °C) δ 7.48 (dd, *J* = 7.3, 2.3 Hz, 1 H), 7.22–7.13 (m, 2 H), 3.08 (br s, 1 H), 2.45 (s, 3 H), 1.78 (s, 6 H); ^{13}C NMR (100 MHz, $CDCl_3$, 25 °C) δ 146.2, 139.8, 129.8, 127.0, 124.9, 123.5, 74.0, 29.7, 24.7; IR (neat) 3423 (O–H) cm^{-1}; HRMS (DART) *m*/*z* calcd for $C_{10}H_{12}Br$ 211.0122, found 211.0129 $[M–OH]^+$.

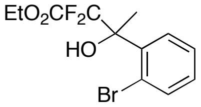

3m

Synthesis of benzylic alcohol 3m: To a suspension of Zn (653.9 mg, 10.0 mmol) in THF (1 mL) was added dibromoethane (32 μL), and this mixture was heated at 65 °C for 1 min. After cooled to room temperature, Me_3SiCl (40 μL) was added. After stirring for 15 min, a solution of o-bromoacetophenone (266.5 μL, 2.0 mmol) and ethyl bromodifluoroacetate (512.9 μL, 4.0 mmol) in THF (3 mL) was added and the resultant mixture was stirred at room temperature for 3 h. The reaction was quenched with sat. NH_4Cl (10 mL) and the whole mixture was extracted with Et_2O (3 × 10 mL). The combined organic layer was washed with brine (10 mL) and dried over Mg_2SO_4. After concentration in vacuo, the crude material was purified by flash column chromatography on silica gel (hexane/EtOAc = 8:1) to afford **3m** (485.5 mg, 75%) as a colorless oil; ^{1}H NMR (400 MHz, $CDCl_3$, 25 °C) δ 7.63 (d, *J* = 8.0 Hz, 1 H), 7.57 (d, *J* = 7.6 Hz, 1 H), 7.32 (dt, *J* = 7.6, 0.8 Hz, 1 H), 7.18 (dt, *J* = 7.6, 1.2 Hz, 1 H), 4.28 (q, *J* = 7.2 Hz, 2 H), 4.14 (s, 1 H), 1.93, (s, 3 H), 1.27 (t, *J* = 7.2 Hz, 3 H); ^{13}C NMR (100 MHz, $CDCl_3$, 25 °C) δ 163.2 (t, *J* = 31.5 Hz), 137.2, 135.7, 130.3, 129.8, 127.2, 120.8, 115.5 (t, *J* = 261.3 Hz), 77.8 (t, *J* = 25.3 Hz), 63.1, 23.7, 13.7; ^{19}F NMR (376 MHz, $CDCl_3$, 25 °C): δ –112.7 (*d*, *J* = 254.2 Hz), –114.1 (*d*, *J* = 254.2 Hz); IR (neat) 3521 (O–H), 1761 (C=O) cm^{-1}; HRMS (DART) *m*/*z* calcd for $C_{12}H_{13}BrF_2O_3$•NH_4 340.0356, found 340.0357 $[M+NH_4]^+$.

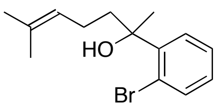

3n

Synthesis of benzylic alcohol 3n: The reported procedure for the synthesis of **3j**[5] was applied to 6-methyl-5-hepten-2-one (1.2 mL, 10.0 mmol). The crude material was purified by flash column chromatography on silica gel (hexane/EtOAc = 100:1~40:1) to afford **3n** (200.6 mg, 7%) as a yellow oil; ^{1}H NMR (400 MHz, $CDCl_3$, 25 °C) δ 7.72 (dd, *J* = 8.0, 1.6 Hz, 1 H), 7.58 (dd, *J* = 8.0, 1.2 Hz, 1 H), 7.30 (dt, *J* = 7.6, 1.2 Hz, 1 H), 7.09 (dt, *J* = 7.6, 1.6 Hz, 1 H), 5.10 (t, *J* = 7.0 Hz, 1 H), 2.54 (s, 1 H), 2.52–2.45 (m, 1 H), 1.97–1.80 (m, 3 H), 1.70 (s, 3 H), 1.65 (s, 3 H), 1.47 (s, 3 H); ^{13}C NMR (100 MHz, $CDCl_3$, 25 °C) δ 145.3, 134.9, 132.4, 128.4, 128.3, 127.4, 124.0, 120.1, 76.2, 40.4, 28.0, 25.7, 23.1, 17.6; IR (neat) 3458 (O–H) cm^{-1}; HRMS (DART) *m*/*z* calcd for $C_{14}H_{19}BrO$•NH_4 300.0963, found 300.0943 $[M+NH_4]^+$.

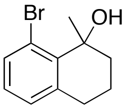

3o

Synthesis of benzylic alcohol 3o: To a solution of 8-bromo-3,4-dihydronaphthalen-1(2*H*)-one[11] (596.1 mg, 2.64 mmol) in Et_2O (5 mL) was added MeMgI (3.0 M in Et_2O, 1.1 mL, 3.17 mmol) at 0 °C. The reaction mixture was stirred for 1.5 h at room temperature. The reaction was quenched with sat. NH_4Cl (10 mL) at 0 °C and the whole mixture was extracted with Et_2O (3 × 10 mL). The combined organic layer was washed with brine (10 mL) and dried over Mg_2SO_4. After concentration in vacuo, the crude material was purified by flash column chromatography on silica gel (hexane/EtOAc = 8:1) to afford **3o** (355.5 mg, 56%) as a colorless solid (m.p. 68.0–70.5 °C); ^{1}H NMR (400 MHz, $CDCl_3$, 25 °C) δ 7.41 (d, J = 7.6 Hz, 1 H), 7.06 (d, J = 7.6 Hz, 1 H), 6.97 (t, J = 7.6 Hz, 1 H), 3.70 (s, 1 H), 2.89 (ddd, J = 16.0, 10.8, 5.6 Hz, 1 H), 2.81 (dt, J = 16.0, 4.4 Hz, 1 H), 2.02–1.97 (m, 2 H), 1.94–1.86 (m, 1 H), 1.82–1.71 (m, 1 H), 1.76 (s, 3 H); ^{13}C NMR (100 MHz, $CDCl_3$, 25 °C) δ 140.0, 139.6, 132.7, 128.9, 127.7, 121.7, 72.3, 40.3, 31.4, 28.4, 20.0; IR (neat) 3425 (O–H) cm^{-1}; HRMS (DART) *m*/*z* calcd for $C_{11}H_{13}BrO{\bullet}NH_4$ 258.0494, found 258.0516 $[M+NH_4]^+$.

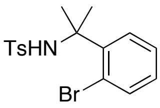

9

Synthesis of sulfonamide 9: To a solution of *o*-bromobenzylamine **7**[10] (214.6 mg, 1.0 mmol) in CH_2Cl_2 (3.0 mL) was added triethylamine (140 μl, 0.95 mmol) and *p*-toluenesulfonyl chloride (180.3 mg, 1.0 mmol) at 0 °C. The reaction mixture was stirred at room temperature for 2 days. The reaction was quenched with 10% HCl (10 mL) and the whole mixture was extracted with CH_2Cl_2 (2 × 20 mL). The combined organic layer was washed with brine (10 mL) and dried over Na_2SO_4. After concentration in vacuo, the crude material was purified by flash column chromatography on silica gel (hexane/EtOAc = 4:1) to afford tosylamide **9** (219.6 mg, 60%) as a brown solid (m.p. 96.8–98.9 °C); ^{1}H NMR (400 MHz, $CDCl_3$, 25 °C) δ 7.44 (d, J = 8.4 Hz, 2 H), 7.30 (dd, J = 8.0, 1.6 Hz, 1 H), 7.25 (dd, J = 8.4, 1.2 Hz, 1 H), 7.15 (dt, J = 7.6, 1.6 Hz, 1 H), 6.97 (d, J = 8.8 Hz, 2 H), 6.96 (dt, J = 7.6, 1.6 Hz, 1 H), 5.89 (s, 1 H), 2.30 (s, 3 H), 1.81 (s, 6 H); ^{13}C NMR (100 MHz, $CDCl_3$, 25 °C) δ 142.5, 141.1, 137.7, 135.4, 128.7, 128.5, 128.2, 127.3, 127.2, 121.7, 58.5, 29.2, 21.3; IR (neat) 3292 (N–H) cm^{-1}; HRMS (ESI) *m*/*z* calcd for $C_{16}H_{18}BrNO_2S{\bullet}Na$ 390.0139, found 390.0141 $[M+Na]^+$.

3. Synthesis of 4-iodo-2-quinolones

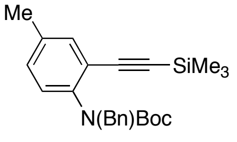

S1

Synthesis of silylalkyne S1: In a sealed tube, a solution of 2-iodo-4-methylaniline (2.33 g, 10.0 mmol) in di-*t*-butyl dicarbonate (3.68 mL, 16.0 mmol) was heated at 90 °C for 2 days. To a solution of the crude product in DMF (50 mL) was added sodium hydride (60% oil, 0.80 g, 20.0 mmol) at 0 °C. The reaction mixture was stirred at room temperature for 1 h. To the

[11] A. Y. Lebedev, A. S. Khartulyari, A. Z. Voskoboynikov, *J. Org. Chem.* **2005**, *70*, 596-602.

resultant mixture was added benzyl bromide (1.43 mL, 12.0 mmol). The reaction mixture was stirred at room temperature for 1 h. The reaction was quenched with sat. NH_4Cl (20 mL), and the mixture was extracted with AcOEt (3 × 20 mL). The combined organic layer was washed with water (2 × 20 mL), brine (20 mL), and dried over $MgSO_4$. After concentration in vacuo, the crude product was purified by flash column chromatography on silica gel (hexane/EtOAc = 50:1~20:1) to afford *tert*-butyl benzyl(2-iodo-4-methylphenyl)carbamate*N*-protected *o*-iodoaniline as a yellow oil.

To a solution of the crude product obtained above (2.01 g, 4.75 mmol) in *t*-butyl methyl ether (20 mL) and diisopropylamine (4 mL) was added $PdCl_2(PPh_3)_2$ (33.3 mg, 0.0475 mmol) and CuI (18.1 mg, 0.095 mmol) at room temperature. The reaction mixture was degassed at –78 °C and to this mixture was added trimethylsilylacetylene (0.724 mL, 5.23 mmol). The reaction mixture was stirred at 50 °C for 5 h. Insoluble materials were filtered with a pad of Celite® and the filtrate was concentrated in vacuo. The obtained crude material was purified by flash column chromatography on silica gel (hexane/EtOAc = 20:1~15:1) to afford silylalkyne **S2** (898 mg, 23% over 3 steps) as a yellow oil; a mixture of two rotamers; ^{1}H NMR ($CDCl_3$, 400 MHz, 25 °C) δ 7.31–7.19 (major+minor) (br m, 6 H), 7.03–6.89 (minor) (br m, 2 H), 6.89 (major) (d, J = 8.0 H, 1 H), 6.70 (major) (d, J = 8.0 H, 1 H), 5.20–4.34 (major+minor) (br m, 2 H), 2.27 (s, 3 H), 1.49 (minor)/1.36 (major) (br s, 9 H), 0.25 (major+minor) (br s, 9 H); ^{13}C NMR ($CDCl_3$, 100 MHz, 25 °C) δ 154.5 (major)/154.2 (minor), 141.8 (minor)/141.4 (major), 138.3 (minor)/137.8 (major), 136.2 (minor)/135.9 (major), 133.5 (minor)/132.9 (major), 129.6 (minor)/129.3 (major), 128.8 (minor)/128.4 (major), 127.8 (major)/127.6 (minor), 126.8 (major/minor), 121.8 (major/minor), 102.0 (major/minor), 97.8 (major+minor), 79.9 (minor)/79.2 (major), 53.7 (minor)/52.6 (major), 28.1 (minor)/27.9 (major), 20.4 (major+minor), –0.4 (major+minor); IR (neat) 2154 (C≡C), 1703 (C=O), 1250 (Si–CH_3), 848 (Si–CH_3) cm^{-1}; HRMS (ESI) *m*/*z* calcd for $C_{24}H_{31}NO_2Si$•Na 416.2022, found 416.2012 $[M+Na]^+$.

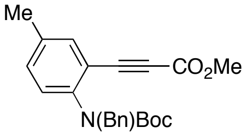

S2

Synthesis of propiolate S2: In a flask, CsF (693 g, 4.56 mmol) was heated under vacuum at 120 °C for 1 h. The flask was filled with CO_2 gas (balloon) and then with dry DMF (1 mL). To the resultant suspension was added drop-wise a solution of silylalkyne **S1** (898 mg, 2.28 mmol) in dry DMF (6 mL) at room temperature. The reaction mixture was stirred at room temperature for 2 h. After addition of methyl iodide (170 μL, 2.74 mmol), the stirring was continued at room temperature for another 1.5 h. The reaction was quenched with sat. NH_4Cl (10 mL) and the whole mixture was extracted with AcOEt (3 × 10 mL). The combined organic layer was washed with water (2 × 10 mL), brine (10 mL), and dried over $MgSO_4$. After concentration in vacuo, the crude material was purified by flash column chromatography on silica gel (hexane/EtOAc = 30:1~15:1) to afford propiolate **S2** (641 mg,

85%) as a yellow oil: a mixture of two rotamers; ^{1}H NMR (400 MHz, $CDCl_3$, 25 °C) δ 7.45–6.81 (major+minor) (br m, 8 H), 5.10–4.30 (major+minor) (br s, 2 H), 3.82 (major+minor) (s, 3 H), 2.29 (major+minor) (s, 3 H), 1.50 (minor)/1.37 (major) (br s, 9 H); ^{13}C NMR ($CDCl_3$, 100 MHz, 25 °C) δ 154.2 (major+minor), 153.9 (major+minor), 143.0 (minor)/142.1 (major), 137.7 (minor)/137.3 (major), 136.4, (major+minor), 134.4 (minor)/134.0 (major), 132.0 (minor)/131.7 (major), 128.4 (major+minor), 128.1 (major+minor), 127.9 (major+minor), 127.7 (minor)/127.0 (major) 118.9 (major or minor), 83.6 (major)/83.2 (minor), 80.7 (minor)/80.1 (major), 54.1 (minor)/53.0 (major), 52.3 (major+minor), 27.8 (major+minor) 20.4 (major+minor); IR (neat) 2218 (C≡C), 1712 (C=O) cm^{-1}; HRMS (ESI) *m/z* calcd for $C_{23}H_{25}NO_4$•Na 402.1681, found 402.1672 $[M+Na]^+$.

Representative procedure for preparation of 4-iodo-2-quinolones 2a –Synthesis of 2a:

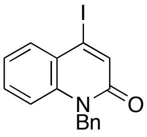

2a

To a solution of methyl 3-(2-(benzyl(*tert*-butoxycarbonyl)amino)phenyl)propiolate (365.2 mg, 1.0 mmol) in acetic acid (2 mL) was added sodium iodide (890.0 mg, 6.0 mmol) at room temperature. The reaction mixture was stirred at 110 °C for 1 h. The reaction was quenched with H_2O (10 mL) and the resultant mixture was extracted with EtOAc (3 × 10 mL). The combined organic layer was washed with sat. Na_2CO_3 (10 mL), sat. $Na_2S_2O_3$ (10 mL), and brine (10 mL). After dried over $MgSO_4$, the solvents were removed in vacuo. The obtained crude material was purified by flash column chromatography on silica gel (hexane/EtOAc = 7:1) to afford 4-iodo-2-quinolone **2a** (340.0 mg, 94%) as a white solid (m.p. 123.6–125.4 °C): ^{1}H NMR (400 MHz, $CDCl_3$, 25 °C) δ 7.85 (dd, J = 8.4, 1.6 Hz, 1 H), 7.61 (s, 1 H), 7.45 (ddd, J = 8.4, 7.2, 1.6 Hz, 1 H) 7.33–7.18 (m, 7 H), 5.54 (s, 2 H); ^{13}C NMR (100 MHz, $CDCl_3$, 25 °C) δ 160.6, 137.8, 135.8, 133.9, 133.1, 131.7, 128.9, 127.4, 126.5, 123.1, 122.2, 115.31, 115.25, 46.3; IR (neat) 1645 (C=O) cm^{-1}; HRMS (ESI) *m/z* calcd for $C_{16}H_{12}INO$•Na 383.9861, found 383.9851 $[M+Na]^+$.

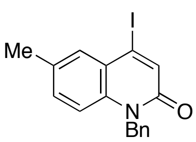

2b

Analytical data for 2b: 306.7 g, 84%; yellow solid (m.p. 178.7–182.1 °C); ^{1}H NMR (400 MHz, $CDCl_3$, 25 °C) δ 7.62 (s, 1 H), 7.58 (s, 1 H), 7.32–7.21 (m, 5 H), 7.19 (d, J = 6.8 Hz, 1 H), 7.10 (d, J = 8.8 Hz, 1 H), 5.52 (s, 2 H), 2.42 (s, 3 H); ^{13}C NMR (100 MHz, $CDCl_3$, 25 °C) δ 160.4, 135.9, 135.7, 133.5, 132.9, 132.8, 132.7, 128.7, 127.3, 126.4, 122.0, 115.14, 115.09, 46.1, 20.6; IR (neat) 1643 (C=O) cm^{-1}; HRMS (ESI) *m/z* calcd for $C_{17}H_{14}INO$•Na 398.0018, found 398.0013 $[M+Na]^+$.

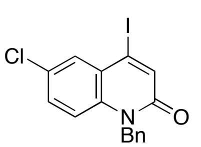

2c

Analytical data for 2c: 365.6 mg, 90%; white solid (m.p. 168.3–169.1 °C); ^{1}H NMR (400 MHz, $CDCl_3$, 25 °C) δ 7.85 (d, *J* = 2.4 Hz, 1 H), 7.62 (s, 1 H), 7.37 (dd, *J* = 8.8 2.4 Hz, 1 H) 7.34–7.20 (m, 6 H), 5.51 (br s, 2 H); ^{13}C NMR (100 MHz, $CDCl_3$, 25 °C) δ 160.2, 136.4 135.4, 134.3, 133.1, 131.7, 129.0, 128.8, 127.6, 126.4, 123.5, 116.9, 113.3, 46.4; IR (neat) 1647 (C=O) cm^{-1}; HRMS (ESI) *m*/*z* calcd for $C_{16}H_{11}ClINO$•Na 417.9472, found 417.9477 $[M+Na]^+$.

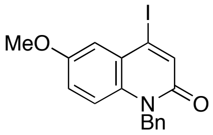

2d

Analytical data for 2d: 369.7 mg, 95%; white solid (m.p. 151.5–153.2 °C); ^{1}H NMR (400 MHz, $CDCl_3$, 25 °C) δ 7.61 (s, 1 H), 7.35–7.13 (m, 7 H), 7.04 (dd, *J* = 9.2, 2.8 Hz, 1 H), 5.52 (br s, 2 H), 3.87 (s, 3 H); ^{13}C NMR (100 MHz, $CDCl_3$, 25 °C) δ 160.2, 155.3, 135.9, 133.6, 132.2, 128.8, 127.4, 126.4, 123.1, 120.1, 116.8, 116.1, 114.6, 55.7, 46.3; IR (neat) 1639 (C=O) cm^{-1}; HRMS (ESI) *m*/*z* calcd for $C_{17}H_{14}INO_2$•Na 413.9967, found 413.9960 $[M+Na]^+$.

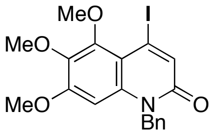

2e

Analytical data for 2e: 391.7 mg, 87%; white solid (m.p. 137.6–138.0 °C); ^{1}H NMR (400 MHz, $CDCl_3$, 25 °C) δ 7.59 (s, 1 H) 7.33–7.18 (m, 5 H), 6.47 (s, 1 H), 5.50 (br s, 2 H), 3.95 (s, 3 H), 3.81 (s, 3 H), 3.70 (s, 3 H); ^{13}C NMR (100 MHz, $CDCl_3$, 25 °C) δ 160.6, 155.8, 150.3, 138.1, 136.1, 136.0, 133.2, 129.0, 127.5, 126.6, 109.2, 101.0, 94.2, 61.5, 60.9, 55.7, 47.0; IR (neat) 1643 (C=O) cm^{-1}; HRMS (ESI) *m*/*z* calcd for $C_{19}H_{18}INO_4$•Na 474.0178, found 474.0180 $[M+Na]^+$.

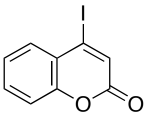

2f

Analytical data for 2f: 145.2 mg, 89%; pale-yellow solid (m.p. 103.3–104.3 °C); ^{1}H NMR (400 MHz, $CDCl_3$, 25 °C) δ 7.70 (dd, *J* = 8.4, 1.2 Hz, 1 H) 7.57 (ddd, *J* = 8.4, 7.2, 1.2 Hz, 1 H), 7.34 (ddd, *J* = 8.4, 7.2, 1.2 Hz, 1 H), 7.27 (dd, *J* = 8.4, 1.2 Hz, 1 H), 7.22 (s, 1 H); ^{13}C NMR (100 MHz, $CDCl_3$, 25 °C) δ 158.1, 151.3, 132.9, 132.4, 127.4, 125.1, 120.6, 120.1, 116.9; IR (neat) 1711 (C=O) cm^{-1}; HRMS (ESI) *m*/*z* calcd for $C_9H_5IO_2$•Na 294.9232, found 294.9218 $[M+Na]^+$.

4. Vinylogous Catellani reactions

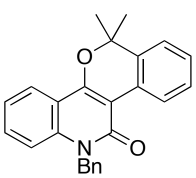

4aa

Representative procedure (1) – Synthesis of 4aa: A solution of 4-iodo-2-quinolone **2a** (72.4 mg, 0.20 mmol), 2-(2-bromophenyl)propan-2-ol **3a** (43.2 mg, 0.20 mmol), $Pd(OAc)_2$ (2.26 mg, 0.01 mmol), norbornene (18.6 mg, 0.20 mmol), and K_2CO_3 (69.3 mg, 0.50 mmol) in dry DMF (4.0 mL) was degassed at –78 °C. The reaction mixture was heated at 105 °C for 2 h. The reaction was quenched with H_2O (10 mL) and the whole mixture was extracted with AcOEt (3 × 20 mL). The combined organic layer was washed with water (2 × 10 mL), brine (10 mL) and dried over $MgSO_4$. After concentration in

vacuo, the crude material was purified by flash column chromatography on silica gel (hexane/EtOAc = 20:1) to afford **4aa** (63.7 mg, 87%) as a white solid (m.p. 154.2–157.9 °C): ^{1}H NMR (400 MHz, $CDCl_3$, 25 °C) δ 9.01 (dd, *J* = 8.0, 1.2 Hz, 1 H), 8.10 (dd, *J* = 8.0, 1.2 Hz, 1 H), 7.45 (ddd, *J* = 8.8, 6.8, 1.6 Hz, 1 H), 7.38 (dt, *J* = 6.8, 1.2 Hz, 1 H), 7.35–7.18 (m, 9 H), 5.64 (br s, 2 H), 1.78 (s, 6 H); ^{13}C NMR (100 MHz, $CDCl_3$, 25 °C) δ 161.5, 156.2, 138.8, 137.0, 136.9, 131.2, 128.8, 127.9, 127.8, 127.1, 126.9, 126.6, 125.8, 123.7, 122.1, 121.8, 116.5, 114.7, 106.5, 80.0, 45.9, 27.5; IR (neat) 1634 (C=O) cm^{-1}; HRMS (ESI) *m*/*z* calcd for $C_{25}H_{21}NO_2$•Na 390.1470, found 390.1461 [M+Na]$^+$.

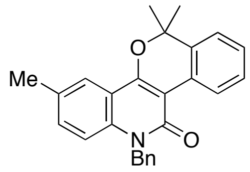

4ba

Analytical data for 4ba: 61.7 mg, 81%; yellow solid (m.p. 177.1–179.5 °C); ^{1}H NMR (400 MHz, $CDCl_3$, 25 °C) δ 9.00 (dd, *J* = 8.0, 1.2 Hz, 1 H), 7.86 (s, 1 H), 7.37 (dt, *J* = 7.6, 1.2 Hz, 1 H), 7.33–7.19 (m, 8 H), 7.14 (d, *J* = 8.4 Hz, 1 H), 5.61 (br s, 2 H), 2.40 (s, 3 H), 1.77 (s, 6 H); ^{13}C NMR (100 MHz, $CDCl_3$, 25 °C) δ 161.3, 156.0, 137.0, 136.9, 132.5, 131.4, 128.7, 127.80, 127.78, 127.0, 126.5, 125.8, 123.2, 122.1, 116.4, 114.6, 106.5, 79.9, 45.8, 27.4, 20.7; IR (neat) 1631 (C=O) cm^{-1}; HRMS (ESI) *m*/*z* calcd for $C_{26}H_{23}NO_2$•Na 404.1627, found 404.1620 [M+Na]$^+$.

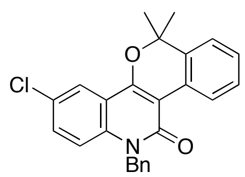

4ca

Analytical data for 4ca: 64.9 mg, 81%; white solid (m.p. 175.8–179.4 °C); ^{1}H NMR (400 MHz, $CDCl_3$, 25 °C) δ 8.99 (d, *J* = 7.6 Hz, 1 H), 8.04 (d, *J* = 2.4 Hz, 1 H), 7.42–7.22 (m, 9 H), 7.18 (d, *J* = 8.8 Hz, 1 H), 5.60 (br s, 2 H), 1.78 (s, 6 H); ^{13}C NMR (100 MHz, $CDCl_3$, 25 °C) δ 161.1, 155.0, 137.2, 137.1, 136.5, 131.1, 128.8, 128.3, 127.9, 127.6, 127.3, 126.5, 125.9, 123.0, 122.2, 117.6, 116.2, 107.3, 80.4, 46.0, 27.5; IR (neat) 1637 (C=O) cm^{-1}; HRMS (ESI) *m*/*z* calcd for $C_{25}H_{20}ClNO_2$•Na 424.1080, found 424.1085 [M+Na]$^+$.

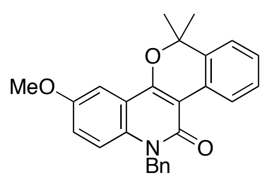

4da

Analytical data for 4da: 72.6 mg, 90%; white solid (m.p. 199.2–201.2 °C); ^{1}H NMR (400 MHz, $CDCl_3$, 25 °C) δ 9.02 (dd, *J* = 8.0, 1.2 Hz, 1 H), 7.50 (d, *J* = 2.4 Hz, 1 H), 7.37 (dt, *J* = 7.6, 1.6 Hz, 1 H), 7.34–7.20 (m, 7 H), 7.18 (d, *J* = 9.6 Hz, 1 H), 7.06 (dd, *J* = 9.2, 2.8 Hz, 1 H), 5.61 (br s, 2 H), 3.87 (s, 3 H), 1.77 (s, 6 H); ^{13}C NMR (100 MHz, $CDCl_3$, 25 °C) δ 161.0, 155.6, 154.6, 137.04, 136.98, 133.5, 128.7, 127.9, 127.8, 127.09, 126.97, 126.5, 125.9, 122.1, 119.9, 117.2, 116.1, 106.9, 105.4, 80.0, 55.7, 45.9, 27.5; IR (neat) 1631 (C=O) cm^{-1}; HRMS (ESI) *m*/*z* calcd for $C_{26}H_{23}NO_3$•Na 420.1576, found 420.1576 [M+Na]$^+$.

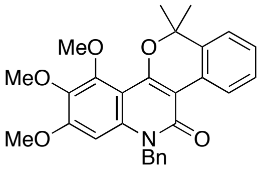

4ea

Analytical data for 4ea: 39.3 mg, 44%; white solid (m.p. 158.0–158.5 °C); ^{1}H NMR (400 MHz, $CDCl_3$, 25 °C) δ 8.92 (dd, J = 8.0, 1.2 Hz, 1 H), 7.36 (dt, J = 7.6, 1.2 Hz, 1 H), 7.33–7.19 (m, 7 H), 6.53 (s, 1 H), 5.60 (br s, 2 H), 3.93 (s, 3 H), 3.84 (s, 3 H), 3.72 (s, 3 H), 1.78 (s, 6 H); ^{13}C NMR (100 MHz, $CDCl_3$, 25 °C) δ 161.3, 157.6, 155.8, 151.8, 138.9, 137.1, 137.0, 136.7, 128.9, 127.7, 127.4, 127.3, 127.2, 126.7, 125.6, 121.7, 105.5, 105.4, 94.6, 79.5, 62.1, 61.2, 55.8, 46.8, 27.0; IR (neat) 1631 (C=O) cm^{-1}; HRMS (ESI) *m*/*z* calcd for $C_{28}H_{27}NO_5$•Na 480.1787, found 480.1789 $[M+Na]^+$.

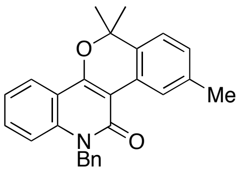

4ab

Analytical data for 4ab: 66.5 mg, 87%; white solid (m.p. 172.0–172.7 °C); ^{1}H NMR (400 MHz, $CDCl_3$, 25 °C) δ 8.86 (s, 1 H), 8.10 (dd, J = 8.0, 1.2 Hz, 1 H), 7.44 (ddd, J = 8.8, 7.2, 1.6 Hz, 1 H), 7.34–7.18 (m, 7 H), 7.14 (d, J = 1.2 Hz, 2 H), 5.64 (br s, 2 H), 2.41 (s, 3 H) , 1.76 (s, 6 H); ^{13}C NMR (100 MHz, $CDCl_3$, 25 °C) δ 161.5, 156.3, 138.7, 137.4, 136.8, 134.3, 131.1, 128.7, 128.6, 127.1, 126.7, 126.5, 126.2, 123.7, 122.1, 121.8, 116.6, 114.6, 106.5, 80.0, 45.8, 27.6, 21.5; IR (neat) 1633 (C=O) cm^{-1}; HRMS (ESI) *m*/*z* calcd for $C_{26}H_{23}NO_2$•Na 404.1627, found 404.1619 $[M+Na]^+$.

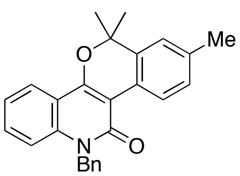

4ac

Analytical data for 4ac: 63.6 mg, 83%; white solid (m.p. 128.2–129.1 °C); ^{1}H NMR (400 MHz, $CDCl_3$, 25 °C) δ 8.90 (d, J = 8.4 Hz, 1 H), 8.09 (dd, J = 7.6, 1.2 Hz, 1 H), 7.43 (ddd, J = 8.8, 6.8, 1.6 Hz, 1 H), 7.34–7.17 (m, 8 H), 7.04 (d, J = 1.2 Hz, 1 H), 5.63 (br s, 2 H), 2.40 (s, 3 H), 1.76 (s, 6 H); ^{13}C NMR (100 MHz, $CDCl_3$, 25 °C) δ 161.5, 155.6, 138.6, 137.7, 137.1, 136.9, 130.9, 128.7, 128.4, 127.1, 126.5, 125.8, 124.1, 123.6, 122.8, 121.7, 116.6, 114.6, 106.6, 79.9, 45.8, 27.6, 21.5; IR (neat) 1633 (C=O) cm^{-1}; HRMS (ESI) *m*/*z* calcd for $C_{26}H_{23}NO_2$•Na 404.1627, found 404.1615 $[M+Na]^+$.

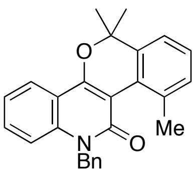

4ad

Analytical data for 4ad: This compound was obtained as an inseparable mixture with remained **3d** as a yellow foam. Thus, the yield was estimated as 74% by internal standard. Analytical sample was obtained by purification with HPLC; ^{1}H NMR (400 MHz, $CDCl_3$, 25 °C) δ 8.06 (dd, J = 8.0, 1.2 Hz, 1 H), 7.44 (ddd, J = 8.8, 6.8, 1.6 Hz, 1 H), 7.35–7.17 (m, 9 H), 7.14–7.08 (m, 1 H), 5.65 (br s, 2 H), 2.42 (s, 3 H), 1.74 (s, 6 H); ^{13}C NMR (100 MHz, $CDCl_3$, 25 °C) δ 160.3, 158.0, 140.7, 139.0, 137.1, 135.6, 131.2, 131.0, 128.8, 127.4, 127.1, 126.5, 126.0, 123.2, 121.6, 119.5, 117.0, 114.6, 109.9, 81.6, 46.0, 26.5, 22.9; IR (neat) 1641 (C=O) cm^{-1}; HRMS (DART) *m*/*z* calcd for $C_{26}H_{23}NO_2$•H 382.1807,, found 382.1821 $[M+H]^+$.

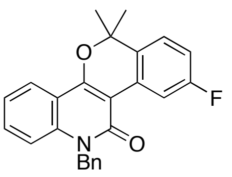

4ae

Analytical data for 4ae: 69.2 mg, 90%; white solid (m.p. 159.9–160.2 °C); ^{1}H NMR (400 MHz, $CDCl_3$, 25 °C) δ 8.82 (dd, *J* = 7.6, 2.8 Hz, 1 H), 8.10 (dd, *J* = 8.0, 1.2 Hz, 1 H), 7.47 (ddd, *J* = 8.8, 7.2, 2.0 Hz, 1 H), 7.34–7.16 (m, 8 H), 7.00, (dt, *J* = 8.0, 2.8 Hz, 1 H), 5.63 (br s, 2 H), 1.77 (s, 6 H); ^{13}C NMR (100 MHz, $CDCl_3$, 25 °C) δ 162.4 (d, *J* = 242.2 Hz), 161.3, 156.8, 139.0, 136.7, 132.6 (d, *J* = 2.8 Hz), 131.6, 129.1 (d, *J* = 10.5 Hz), 128.8, 127.2, 126.5, 123.9, 123.6 (d, *J* = 8.6 Hz), 121.9, 116.2, 114.7, 114.3 (d, *J* = 22.9 Hz), 112.8 (d, *J* = 25.8 Hz), 105.7 (d, *J* = 1.9 Hz), 80.1, 46.0, 27.7; ^{19}F NMR (376 MHz, $CDCl_3$, 25 °C): δ –113.7; IR (neat) 1763 (C=O), 1637 (C=O) cm^{-1}; IR (neat) 1633 (C=O) cm^{-1}; HRMS (ESI) *m*/*z* calcd for $C_{25}H_{20}FNO_2$•Na 408.1376, found 408.1369 $[M+Na]^+$.

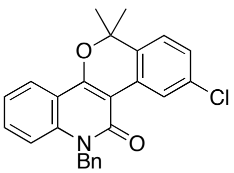

4af

Analytical data for 4af: 67.7 mg, 84%; white solid (m.p. 157.5–158.7 °C); ^{1}H NMR (400 MHz, $CDCl_3$, 25 °C) δ 9.10 (d, *J* = 2.0 Hz, 1 H), 8.10 (dd, *J* = 8.0, 1.2 Hz, 1 H), 7.47 (ddd, *J* = 8.4, 5.6, 1.2 Hz, 1 H), 7.35–7.20 (m, 8 H), 7.16 (d, *J* = 8.8 Hz, 1 H), 5.63 (br s, 2 H), 1.76 (s, 6 H); ^{13}C NMR (100 MHz, $CDCl_3$, 25 °C) δ 161.2, 156.8, 139.0, 136.6, 135.1, 134.0, 131.7, 128.8, 128.6, 127.7, 127.2, 126.5, 125.7, 123.8, 123.5, 122.0, 116.2, 114.7, 105.5, 79.9, 45.9, 27.5; IR (neat) 1633 (C=O) cm^{-1}; HRMS (ESI) *m*/*z* calcd for $C_{25}H_{20}ClNO_2$•Na 424.1073, found 424.1080 $[M+Na]^+$.

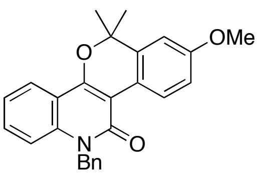

4ag

Analytical data for 4ag: 65.1 mg, 82%; white solid (m.p. 172.8–173.9 °C); ^{1}H NMR (400 MHz, $CDCl_3$, 25 °C) δ 8.97 (d, *J* = 7.2 Hz, 1 H), 8.07 (dd, *J* = 8.0, 1.2 Hz, 1 H), 7.42 (ddd, *J* = 8.4, 6.8, 1.6 Hz, 1 H), 7.34–7.18 (m, 7 H), 6.90 (dd, *J* = 7.2, 2.8 Hz, 1 H), 6.79 (d, *J* = 2.8 Hz, 1 H), 5.62 (br s, 2 H), 3.86 (s, 3 H), 1.74 (s, 6 H); ^{13}C NMR (100 MHz, $CDCl_3$, 25 °C) δ 161.5, 159.4, 154.7, 139.1, 138.4, 136.9, 130.7, 128.7, 127.5, 127.1, 126.5, 123.4, 121.8, 119.8, 116.6, 114.6, 111.6, 109.2, 106.5, 79.7, 55.3, 45.9, 27.4; IR (neat) 1633 (C=O) cm^{-1}; HRMS (ESI) *m*/*z* calcd for $C_{26}H_{23}NO_3$•Na 420.1576, found 420.1563 $[M+Na]^+$.

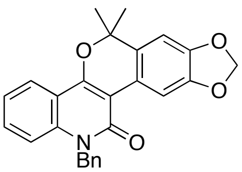

4ah

Analytical data for 4ah: 65.5 mg, 81%; pale-brown solid (m.p. 216.3–221.4 °C); ^{1}H NMR (400 MHz, $CDCl_3$, 25 °C) δ 8.62 (s, 1 H), 8.07 (dd, *J* = 8.0, 1.2 Hz, 1 H), 7.43 (ddd, *J* = 8.6, 7.0, 1.6 Hz, 1 H), 7.32–7.19 (m, 7 H), 6.75 (s, 1 H), 5.98 (s, 2 H), 5.63 (br s, 2 H), 1.73 (s, 6 H); ^{13}C NMR (100 MHz, $CDCl_3$, 25 °C) δ 161.4, 155.0, 147.2, 146.9, 138.4, 136.8, 131.4, 130.9, 128.7, 127.1, 126.5, 123.4, 121.8, 121.2, 116.4, 114.6, 106.7, 106.6, 103.1, 101.1, 79.8, 45.9, 27.5; IR (neat) 1633 (C=O) cm^{-1}; HRMS (ESI) *m*/*z* calcd for $C_{26}H_{21}NO_4$•Na 434.1368, found 434.1367 $[M+Na]^+$.

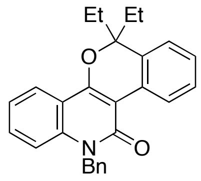

4ai

Analytical data for 4ai: 62.7 mg, 79%; white solid (m.p. 158.9–159.2 °C); ^{1}H NMR (400 MHz, $CDCl_3$, 25 °C) δ 9.10 (dd, J = 8.0, 1.2 Hz, 1 H), 8.06 (dd, J = 8.0, 1.6 Hz, 1 H), 7.44 (ddd, J = 8.8, 7.2, 1.6 Hz, 1 H), 7.36 (dt, J = 8.0, 1.6 Hz, 1 H), 7.33–7.18 (m, 8 H), 7.11 (dd, J = 7.2, 1.6 Hz, 1 H), 5.62 (br s, 2 H), 2.10 (q, J = 7.6 Hz, 4 H), 0.95 (t, J = 7.6 Hz, 6 H); ^{13}C NMR (100 MHz, $CDCl_3$, 25 °C) δ 161.6, 156.5, 138.9, 136.9, 133.7, 131.1, 128.7, 128.0, 127.6, 127.3, 127.1, 126.6, 126.0, 123.8, 123.6, 121.8, 116.3, 114.7, 105.5, 85.7, 45.9, 31.1, 8.1; IR (neat) 1633 (C=O) cm^{-1}; HRMS (ESI) m/z calcd for $C_{27}H_{25}NO_2$•Na 418.1783, found 418.1778 $[M+Na]^+$.

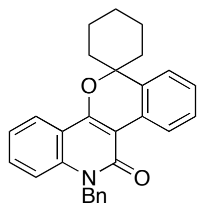

4aj

Analytical data for 4aj: 59.5 mg, 74%; pale-brown solid (m.p. 219.6–221.5 °C); ^{1}H NMR (400 MHz, $CDCl_3$, 25 °C) δ 9.00 (dd, J = 8.0, 1.2 Hz, 1 H), 8.17 (dd, J = 8.0, 1.2 Hz, 1 H), 7.46 (ddd, J = 8.0, 7.6, 1.2 Hz, 1 H), 7.40–7.21 (m, 10 H), 5.63 (br s, 2 H), 2.37 (br d, J = 13.6 Hz, 2 H), 2.00–1.76 (m, 5 H), 1.71 (br d, J = 13.6 Hz, 2 H), 1.43–1.32 (m, 1 H); ^{13}C NMR (100 MHz, $CDCl_3$, 25 °C) δ 161.4, 155.6, 138.9, 137.2, 136.9, 131.1, 128.7, 127.9, 127.7, 127.14, 127.09, 126.6, 125.8, 123.5, 121.92, 121.88, 116.6, 114.7, 107.0, 80.4, 45.9, 34.8, 25.3, 21.7; IR (neat) 1633 (C=O) cm^{-1}; HRMS (ESI) m/z calcd for $C_{28}H_{25}NO_2$•Na 430.1783, found 430.1775 $[M+Na]^+$.

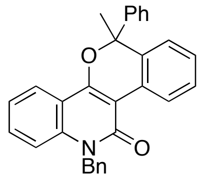

4ak

Analytical data for 4ak: 60.0 mg, 72%; yellow solid (m.p. 171.5–177.4 °C); ^{1}H NMR (400 MHz, $CDCl_3$, 25 °C) δ 9.01 (dd, J = 8.0, 1.2 Hz, 1 H), 8.24 (dd, J = 8.0, 1.6 Hz, 1 H), 7.48–7.42 (m, 2 H), 7.39 (dt, J = 7.2, 1.6 Hz, 1 H), 7.33–7.19 (m, 13 H), 5.72 (br d, J = 16.8 Hz, 1 H), 5.39 (br d, J = 16.8 Hz, 1 H), 2.18 (s, 3 H); ^{13}C NMR (100 MHz, $CDCl_3$, 25 °C) δ 161.2, 156.3, 144.1, 138.8, 136.7, 135.0, 131.2, 128.7, 128.3, 128.0, 127.9, 127.7, 127.6, 127.1, 126.4, 125.9, 125.8, 124.5, 123.5, 122.0, 116.3, 114.7, 107.6, 83.2, 45.9, 28.3; IR (neat) 1633 (C=O) cm^{-1}; HRMS (ESI) m/z calcd for $C_{30}H_{23}NO_2$•Na 452.1627, found 452.1619 $[M+Na]^+$.

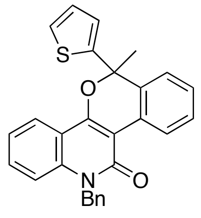

4al

Analytical data for 4al: 51.8 mg, 60%; pale-yellow solid (m.p. 192.5–195.3 °C); ^{1}H NMR (400 MHz, $CDCl_3$, 25 °C) δ 9.06 (dd, J = 8.0, 1.2 Hz, 1 H), 8.18 (dd, J = 8.0, 1.2 Hz, 1 H), 7.52–7.40 (m, 2 H), 7.37 (dt, J = 7.6, 1.2 Hz, 1 H), 7.32–7.18 (m, 9 H), 6.80 (dd, J = 5.2, 3.6 Hz, 1 H), 6.70 (dd, J = 3.6, 1.2 Hz, 1 H), 5.73 (br d, J = 16.8 Hz, 1 H), 5.43 (br d, J = 16.8 Hz, 1 H), 2.29 (s, 3 H); ^{13}C NMR (100 MHz, $CDCl_3$, 25 °C) δ 161.2, 155.8, 148.6, 138.7, 136.7, 134.8, 131.2, 128.7, 128.6, 127.8, 127.6, 127.0, 126.4, 126.2, 126.0,

125.9, 125.7, 123.8, 123.7, 121.9, 116.2, 114.6, 107.3, 80.8, 45.8, 29.0; IR (neat) 1633 (C=O) cm^{-1}; HRMS (ESI) *m/z* calcd for $C_{28}H_{21}NO_2S$•Na 458.1191, found 458.1172 [M+Na]$^+$.

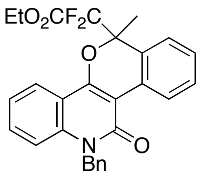

4am

Analytical data for 4am: Because of instability under the reaction conditions, 2.5 equivs of **3m** was used. The title compound was purified by short column chromatography and recrystallization; 54.6 mg, 61%; white solid (m.p. 126.8–128.5 °C); ^{1}H NMR (400 MHz, $CDCl_3$, 25 °C) δ 9.21 (d, J = 7.6 Hz, 1 H), 8.01 (dd, J = 8.0, 1.6 Hz, 1 H), 7.52–7.44 (m, 2 H), 7.39–7.34 (m, 2 H), 7.32–7.20 (m, 7 H), 5.69 (br d, J = 16.8 Hz, 1 H), 5.54 (br d, J = 16.8 Hz, 1 H), 4.13 (dq, J = 10.8, 7.2 Hz, 1 H), 4.08 (dq, J = 10.8, 7.2 Hz, 1 H), 2.11 (s, 3 H), 1.02 (t, J = 7.2 Hz, 3 H); ^{13}C NMR (100 MHz, $CDCl_3$, 25 °C) δ 162.7 (t, J = 31.5 Hz), 161.0, 155.3, 138.7, 136.6, 131.6, 129.5, 128.7, 128.0, 127.8, 127.7, 127.1, 126.5, 126.0, 125.3, 123.6, 121.9, 115.1, 114.7, 114.2 (t, J = 262.2 Hz), 105.2, 81.5 (t, J = 26.2 Hz), 63.2, 45.9, 22.7, 13.4; ^{19}F NMR (376 MHz, $CDCl_3$, 25 °C): δ –115.3; IR (neat) 1763 (C=O), 1637 (C=O) cm^{-1}; HRMS (ESI) *m/z* calcd for $C_{28}H_{23}F_2NO_4$•Na 498.1493, found 498.1495 [M+Na]$^+$.

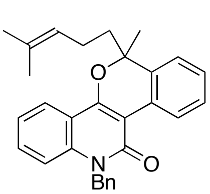

4an

Analytical data for 4an: 82.4 mg, 94%; yellow oil; ^{1}H NMR (400 MHz, $CDCl_3$, 25 °C) δ 9.04 (d, J = 8.0 Hz, 1 H), 8.10 (d, J = 8.0 Hz, 1 H), 7.44 (ddd, J = 8.0, 7.2, 1.2 Hz, 1 H), 7.37 (dt, J = 7.2, 1.6 Hz, 1 H), 7.34–7.18 (m, 9 H), 5.63 (br s, 2 H), 5.06 (br s, 1 H), 2.16–2.08 (m, 3 H), 2.01–1.96 (m, 1 H), 1.78 (s, 3 H), 1.62 (s, 3 H), 1.48 (s, 3 H); ^{13}C NMR (100 MHz, $CDCl_3$, 25 °C) δ 161.4, 156.0, 138.7, 136.8, 135.9, 132.0, 131.1, 128.7, 127.7, 127.6, 127.1, 127.0, 126.5, 125.8, 123.53, 123.49, 122.8, 121.8, 116.3, 114.6, 106.1, 82.2, 45.8, 39.7, 25.8, 25.5, 22.6, 17.4; IR (neat) 1636 (C=O) cm^{-1}; HRMS (ESI) *m/z* calcd for $C_{30}H_{29}NO_2$•Na 458.2096, found 458.2091 [M+Na]$^+$.

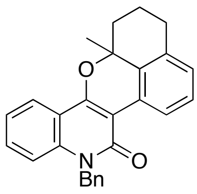

4ao

Analytical data for 4ao: 25.0 mg, 34%; white solid (m.p. 188.2–191.9 °C); ^{1}H NMR (400 MHz, $CDCl_3$, 25 °C) δ 8.77 (d, J = 8.0 Hz, 1 H), 8.11 (dd, J = 8.0, 1.6 Hz, 1 H), 7.44 (ddd, J = 8.8, 7.2, 1.6 Hz, 1 H), 7.45–7.18 (m, 8 H), 7.10 (d, J = 7.2 Hz, 1 H), 5.75 (br d, J = 16.8 Hz, 1 H), 5.54 (br d, J = 16.8 Hz, 1 H), 2.92–2.88 (m, 2 H), 2.40–2.28 (m, 2 H), 2.18–1.96 (m, 2 H), 1.51 (s, 3 H); ^{13}C NMR (100 MHz, $CDCl_3$, 25 °C) δ 161.5, 156.0, 138.7, 136.9, 133.3, 131.3, 131.1, 128.7, 128.4, 127.7, 127.1, 127.0, 126.6, 123.7, 123.2, 121.8, 116.6, 114.6, 107.6, 78.4, 45.9, 35.9, 28.1, 24.5, 20.1; IR (neat) 1633 (C=O) cm^{-1}; HRMS (ESI) *m/z* calcd for $C_{27}H_{23}NO_2$•Na 416.1627, found 416.1628 [M+Na]$^+$.

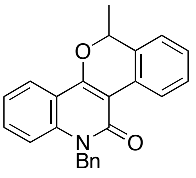

4ap

Analytical data for 4ap: 27.9 mg, 39%; orange solid (m.p. 157.6–159.1 °C); ^{1}H NMR (400 MHz, $CDCl_3$, 25 °C) δ 8.92 (dd, *J* = 8.0, 1.2 Hz, 1 H), 8.09 (dd, *J* = 8.0, 1.6 Hz, 1 H), 7.45 (ddd, *J* = 8.4, 7.2, 1.6 Hz, 1 H), 7.40 (dt, *J* = 8.0, 1.2 Hz, 1 H), 7.35–7.14 (m, 9 H), 5.80–5.43 (br m, 2 H), 5.53 (q, *J* = 6.4 Hz, 1 H), 1.73 (d, *J* = 6.4 Hz, 3 H); ^{13}C NMR (100 MHz, $CDCl_3$, 25 °C) δ 161.5, 156.9, 138.8, 136.8, 133.1, 131.3, 128.8, 128.3, 127.8, 127.6, 127.1, 126.6, 125.6, 123.6, 123.1, 121.9, 116.3, 114.7, 107.0, 75.3, 45.9, 20.1; IR (neat) 1633 (C=O) cm^{-1}; HRMS (ESI) *m*/*z* calcd for $C_{24}H_{19}NO_2$•Na 376.1314, found 376.1303 $[M+Na]^+$.

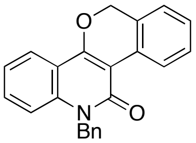

4aq

Analytical data for 4aq: 11.9 mg, 17%; orange solid (m.p. 130.4–132.3 °C); ^{1}H NMR (400 MHz, $CDCl_3$, 25 °C) δ 8.86 (d, *J* = 8.0 Hz, 1 H), 8.06 (dd, *J* = 7.6, 1.6 Hz, 1 H), 7.45 (ddd, *J* = 8.4, 7.2, 1.6 Hz, 1 H), 7.42 (t, *J* = 6.8, 1 H), 7.35–7.14 (m, 9 H), 5.64 (br s, 2 H), 5.34 (s, 2 H); ^{13}C NMR (100 MHz, $CDCl_3$, 25 °C) δ 161.4, 158.3, 138.7, 136.8, 131.4, 128.8, 128.7, 128.6, 128.5, 127.7, 127.2, 126.6, 125.4, 123.8, 123.6, 121.9, 115.8, 114.8, 107.7, 69.5, 45.8; IR (neat) 1633 (C=O) cm^{-1}; HRMS (ESI) *m*/*z* calcd for $C_{23}H_{17}NO_2$•Na 362.1157, found 362.1149 $[M+Na]^+$.

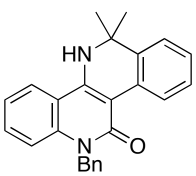

8

Analytical data for 8: 64.6 mg, 88%; white solid (m.p. 150.2–153.8 °C); ^{1}H NMR (400 MHz, $CDCl_3$, 25 °C) δ 9.05 (d, *J* = 8.4 Hz, 1 H), 7.60 (dd, *J* = 7.2, 1.2 Hz, 1 H), 7.42 (ddd, *J* = 8.8, 7.2, 1.6 Hz, 1 H), 7.37–7.15 (m, 10 H), 5.61 (br s, 2 H), 4.91 (br s, 1 H), 1.63 (s, 6 H); ^{13}C NMR (100 MHz, $CDCl_3$, 25 °C) (one overlapping C*sp*^{2} signal)) δ 160.9, 145.2, 138.9, 137.6, 137.3, 130.6, 129.6, 128.7, 127.0, 126.9, 126.5, 126.0, 122.0, 121.3, 120.3, 115.7, 114.3, 102.3, 53.7, 45.7, 29.6; IR (neat) 3338 (N–H), 1606 (C=O) cm^{-1}; HRMS (ESI) *m*/*z* calcd for $C_{25}H_{22}N_2O$•Na 389.1630, found 389.1622 $[M+Na]^+$.

Three-component coupling products **10a,b** and **11** were also obtained under the Catellani reaction condition.

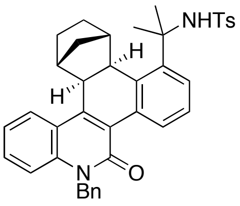

10a

Analytical data for 10a: 103.3 mg, 85%; white solid (m.p. 203.4–205.3 °C); ^{1}H NMR (400 MHz, $CDCl_3$, 25 ˚C) δ 9.27 (dd, *J* = 8.4, 1.2 Hz, 1 H), 7.69 (dd, *J* = 7.2, 1.2 Hz, 1 H), 7.48–7.21 (m, 11 H), 7.20 (t, *J* = 8.0 Hz, 1 H), 6.92 (d, *J* = 8.0 Hz, 2 H), 5.86 (br d, *J* = 13.6 Hz, 1 H), 5.42 (br d, *J* = 13.6 Hz, 1 H), 4.72 (s, 1 H), 3.80 (d, *J* = 9.2 Hz, 1 H), 3.29 (d, *J* = 9.6 Hz, 1 H), 2.19 (d, *J* = 3.2 Hz, 1 H), 2.00 (s, 1 H), 1.98 (s, 3 H), 1.80 (s, 6 H), 1.79–1.51 (m, 4 H), 1.65 (d, *J* = 11.0 Hz, 1 H), 1.00 (d, *J* = 11.0 Hz, 1 H); ^{13}C NMR (100 MHz,

$CDCl_3$, 25 °C) δ 160.3, 143.2, 142.7, 139.4, 139.1, 138.8, 136.8, 136.6, 132.3, 129.8, 129.0, 128.8, 128.1, 127.5, 127.2, 127.0, 126.6, 125.7, 124.9, 123.8, 122.0, 120.0, 115.0, 60.3, 51.5, 46.8, 46.5, 44.9, 42.6, 33.6, 33.4, 31.2, 30.6, 29.2, 20.8; IR (neat) 3271 (N–H), 1630 (C=O) cm^{-1}; HRMS (ESI) *m/z* calcd for $C_{39}H_{38}N_2O_3S$•Na 637.2501, found 637.2494 $[M+Na]^+$.

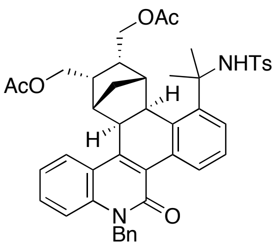

10b

Analytical data for 10b: 76.5 mg, 51%; brown solid (m.p. 114.2–115.4 °C); ^1H NMR (400 MHz, $CDCl_3$, 25 ˚C) δ 9.20 (d, *J* = 8.0 Hz, 1 H), 7.85 (d, *J* = 8.0 Hz, 1 H), 7.47 (d, *J* = 8.0 Hz, 2 H), 7.41–7.14 (m, 10 H), 7.03 (d, *J* = 8.0 Hz, 2 H), 5.84 (br s, 1 H), 5.44 (br s, 1 H), 5.15 (s, 1 H), 4.73 (dd, J = 10.4, 6.0 Hz, 1 H), 4.68 (d, *J* = 9.2 Hz, 1 H), 4.61–4.55 (m, 1 H), 4.41 (dd, *J* = 12.0, 6.4 Hz, 1 H), 4.19 (t, *J* = 11.4 Hz, 1 H), 3.60 (d, *J* = 9.6 Hz, 1 H), 2.57 (br s, 1 H), 2.45 (br s, 1 H), 2.31 (s, 2 H), 2.21 (s, 3 H), 2.14 (s, 3 H), 2.07 (s, 3 H), 1.98 (d, *J* = 11.2 Hz, 1 H), 1.85 (s, 3 H), 1.70 (s, 3 H), 1.22 (d, *J* = 12.0 Hz, 1 H); ^{13}C NMR (100 MHz, $CDCl_3$, 25 °C) δ 171.0, 170.5, 160.1, 142.8, 140.7, 139.8, 138.7, 136.6, 135.0, 133.2, 129.7, 129.4, 128.7, 128.2, 127.1 (2C), 126.7, 126.5, 125.9, 125.2, 124.7, 121.7, 119.8, 114.9, 62.5, 62.3, 60.7, 52.8, 49.2, 46.5, 42.2, 39.7, 38.2, 36.5, 35.7, 33.4, 32.1, 21.18, 21.11; IR (neat) 3253 (N–H), 1739 (C=O), 1633 (C=O) cm^{-1}; HRMS (ESI) *m/z* calcd for $C_{45}H_{46}N_2O_7S$•Na 781.2923, found 781.2904 $[M+Na]^+$.

The reaction of **2a** with **3o** afforded an inseparable mixture of **S3** and **S4** (35.5 mg) in a ratio of **S3**/**S4** = 2:1. Analytically pure sample of **S4** was obtained by partial separation using preparative HPLC. In comparison with the spectral data of **10**, **S4** was tentatively assigned as the three-component annulation product. Although **S4** was obtained as a single diastereomer, its stereochemistry could not be elucidated. The structure of **S3** was tentatively assigned as a singlet of the C4 proton of the quinolone ring was observed at δ 7.69 ppm.

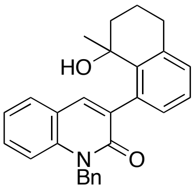

S3

Analytical data for S3: 21.9 mg, 28% based on ^1H NMR analysis; colorless oil; ^1H NMR (400 MHz, $CDCl_3$, 25 ˚C) δ 7.69 (s, 1 H), 7.59 (dd, *J* = 8.0, 1.2 Hz, 1 H), 7.44 (ddd, *J* = 8.8, 7.2, 1.6 Hz, 1 H), 7.34 (d, *J* = 8.8 Hz, 1 H), 7.33–7.22 (m, 5 H), 7.22 (d, *J* = 6.8 Hz, 1 H), 7.21 (d, *J* = 6.8 Hz, 1 H), 7.17 (d, *J* = 7.2 Hz, 1 H), 6.97 (dd, *J* = 7.2, 1.2 Hz, 1 H), 5.72 (br d, *J* = 15.8 Hz, 1 H), 5.52 (br d, *J* = 15.8 Hz, 1 H), 3.01–2.84 (m, 2 H), 2.09–1.76 (m, 4 H), 1.75 (br s, 1 H), 1.40 (s, 3 H); ^{13}C NMR (100 MHz, $CDCl_3$, 25 °C) δ 164.1, 141.8, 138.8, 137.9, 137.5, 137.3, 136.4, 136.2, 130.2, 130.1, 128.8, 128.7, 127.3, 126.9, 126.8, 122.5, 121.1, 115.1, 71.1, 46.8, 41.8, 31.1, 30.2, 20.0; IR (neat) 3365 (O–H), 1631 (C=O) cm^{-1}; HRMS (ESI) *m/z* calcd for $C_{27}H_{25}NO_2$•Na 418.1783, found 418.1792 $[M+Na]^+$.

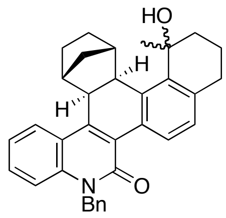
S4

Analytical data for S4: 13.6 mg, 14% based on ^{1}H NMR analysis; single diastereomer; white solid (m.p. 191.4–195.0 °C); ^{1}H NMR (400 MHz, $CDCl_3$, 25 ˚C) δ 7.41 (t, *J* = 7.8 Hz, 1 H), 7.33–7.19 (m, 6 H), 7.15 (d, *J* = 8.0 Hz, 1 H), 7.07 (d, *J* = 7.2 Hz, 1 H), 7.02 (d, *J* = 8.8 Hz, 1 H), 6.96 (d, *J* = 7.2 Hz, 1 H), 5.61 (d, *J* = 15.6 Hz, 1 H), 5.35 (d, *J* = 15.6 Hz, 1 H), 3.99 (br s, 1 H), 3.35 (d, *J* = 6.0 Hz, 1 H), 3.00 (d, *J* = 6.0 Hz, 1 H), 2.98–2.84 (m, 2 H), 2.53 (d, *J* = 3.6 Hz, 1 H), 2.41 (d, *J* = 3.6 Hz, 1 H), 2.04–1.74 (m, 4 H), 1.66–1.46 (m, 4 H), 1.39 (s, 3 H), 1.44–1.14 (m, 2 H); ^{13}C NMR (100 MHz, $CDCl_3$, 25 °C) δ 154.6, 150.2, 148.5, 144.1, 141.9, 137.9, 136.7, 136.0, 133.8, 131.7, 131.4, 129.9, 128.7, 128.3, 127.2, 126.4, 117.5, 110.9, 70.8, 60.4, 54.0, 53.4, 46.5, 42.3, 41.3, 32.7, 31.5, 29.5, 29.2, 28.7, 20.1; IR (neat) 3396 (O–H), 1631 (C=O) cm^{-1}; HRMS (ESI) *m*/*z* calcd for $C_{34}H_{33}NO_2$•Na 510.2409, found 510.2384 $[M+Na]^+$.

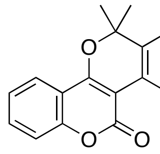
4fa

Synthesis of 4fa: A solution of 4-iodocoumarin **2f** (30.0 mg, 0.11 mmol), 2-(2-bromophenyl)propan-2-ol **3a** (23.7 mg, 0.11 mmol), $Pd(OAc)_2$ (1.2 mg, 0.006 mmol), P(2-furyl)$_3$ (2.6 mg, 0.011 mmol), norbornene (10.4 mg, 0.11 mmol), and K_2CO_3 (38.1 mg, 0.28 mmol) in dry *m*-xylene (2.2 mL) was degassed at –78 °C. The reaction mixture was heated at 140 °C for 18 h. After cooling to room temperature, the crude mixture was purified by flash column chromatography on silica gel (hexane/EtOAc = 20:1~8:1) to afford **4fa** (25.0 mg, 82%) as a yellow foam: ^{1}H NMR (400 MHz, $CDCl_3$, 25 °C) δ 8.68 (d, *J* = 8.0 Hz, 1 H), 7.91 (dd, *J* = 8.0, 1.2 Hz, 1 H), 7.57 (ddd, *J* = 8.0, 7.2, 1.2 Hz, 1 H), 7.40 (dt, *J* = 8.0, 1.2 Hz, 1 H), 7.36–7.29 (m, 3 H), 7.22 (d, *J* = 8.0 Hz, 1 H), 1.78 (s, 6 H); ^{13}C NMR (100 MHz, $CDCl_3$, 25 °C) δ 160.2, 159.2, 152.9, 136.0, 132.3, 128.3, 128.1, 125.1, 125.0, 123.9, 123.1, 122.3, 116.3, 115.8, 101.4, 81.5, 27.8; IR (neat) 1714 (C=O) cm^{-1}; HRMS (ESI) *m*/*z* calcd for $C_{18}H_{14}O_3$•Na 301.0841, found 301.0864 $[M+Na]^+$.

5. Control Experiments

Reaction of 2a with 3a in the absence of norbornene. Homo coupling product **5** was obtained under the Catellani reaction conditions, except for using 10 mol % of $Pd(OAc)_2$ in the absence of norbornene.

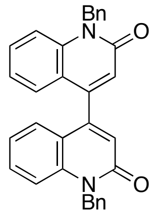
5

Analytical data for 5: 64.6 mg, 88%; white solid (m.p. 246.8–249.7 °C); ^{1}H NMR (400 MHz, $CDCl_3$, 25 °C) δ 7.47 (ddd, *J* = 8.4, 7.2, 1.2 Hz, 2 H), 7.42–7.27 (m, 14 H), 7.08 (ddd, *J* = 8.0, 7.2, 0.8 Hz, 2 H), 6.87 (s, 2 H), 5.70 (br d, *J* = 15.6 Hz, 2 H), 5.62 (br d, *J* = 15.6 Hz, 2 H); ^{13}C NMR (100 MHz, $CDCl_3$, 25 °C) δ 161.6, 146.6, 139.6, 136.1, 131.3, 128.9, 127.5, 126.7, 122.5, 121.8, 119.9, 115.5, 46.3; IR (neat) 1652 (C=O) cm^{-1}; HRMS (ESI) *m*/*z* calcd for $C_{32}H_{24}N_2O_2$•Na 491.1736, found 491.1747 $[M+Na]^+$.

Reaction of 2a with *o*-bromophenol. 4-aryloxy-2-quinolone **6b** was obtained under the Catellani reaction conditions, except for using 10 mol % of $Pd(OAc)_2$ and 1 equiv of norbornene.

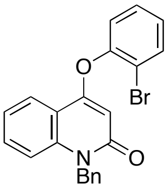

6b

Analytical data for 6b: 21.7 mg, 27%; white solid (m.p. 246.8–249.7 °C); ^{1}H NMR (400 MHz, $CDCl_3$, 25 °C) δ 8.23 (dd, J = 7.6, 1.2 Hz, 1 H), 7.71 (dd, J = 8.0, 1.2 Hz, 1 H), 7.51 (dt, J = 7.6, 1.6 Hz, 1 H), 7.43 (dt, J = 7.6, 1.6 Hz, 1 H), 7.34–7.18 (m, 9 H), 5.76 (s, 1 H), 5.53 (br s, 2 H); ^{13}C NMR (100 MHz, $CDCl_3$, 25 °C) δ 163.5, 161.7, 150.3, 139.6, 136.5, 134.3, 131.7, 129.2, 128.8, 127.7, 127.2, 126.5, 123.6, 122.1, 116.4, 116.0, 115.1, 100.0, 45.7; IR (neat) 1647 (C=O) cm^{-1}; HRMS (ESI) *m/z* calcd for $C_{22}H_{16}BrNO_2$•Na 428.0262 found 428.0268 $[M+Na]^+$.

Preparation of 2a-*d*$_1$: To a solution of methyl 3-(2-(benzyl(*tert*-butoxycarbonyl)amino)phenyl)propiolate (182.9 mg, 0.5 mmol) in acetic acid-*d*$_4$ (1 mL) was added sodium iodide (448.7 mg, 3.0 mmol) at room temperature. The reaction mixture was stirred at 110 °C for 1 h. The reaction was quenched with H_2O (10 mL) and the resultant mixture was extracted with EtOAc (3 × 10 mL). The combined organic layer was washed with sat. Na_2CO_3 (10 mL), sat. $Na_2S_2O_3$ (10 mL), and brine (10 mL). After dried over $MgSO_4$, the solvents were removed in vacuo. The obtained crude material was purified by flash column chromatography on silica gel (hexane/EtOAc = 8:1) to afford 4-iodo-2-quinolone **2a**-*d*$_1$ (85%D, 169.2 mg, 94%) as a white solid (m.p. 125.1–127.1 °C).

Competition experiment using 2a-*d*$_1$: In a similar manner with the reaction of **2a** with **3a**, the reaction of 4-iodo-2-quinolone **2a** (51.1 mg, 0.14 mmol) and **2a-*d*$_1$** (85%D, 72.4 mg, 0.20 mmol) with 2-(2-bromophenyl)propan-2-ol **3a** (37.0 mg, 0.17 mmol) was performed in the presence of $Pd(OAc)_2$ (1.97 mg, 0.009 mmol), norbornene (37.0 mg, 0.17 mmol), and K_2CO_3 (58.8 mg, 0.42 mmol) in dry DMF (3.4 mL) at 105 °C for 1 h. After an usual purification, **2a**-*d*$_1$ (51%D, 60.0 mg, 49%) was recovered along with **4aa** (52.9 mg, 42%).

6. Single Crystal X-ray Diffraction Study

A single crystal of **10a** was mounted on a glass fiber, and diffraction data were collected in θ ranges specified in Table S1 at 123 K on a Brucker D8 QUEST diffractomter with graphite monochromatized Mo Ka radiation (λ = 0.71073 Å). The absorption correction was made using SADABS. The structure was solved by direct methods and refined by the full-matrix least-squares on F^2 by using SHELXL-2013.[12] All non-hydrogen atoms were refined with anisotropic displacement parameters. All hydrogen atoms were placed in calculated positions. Final refinement details are compiled in Table S1. The supplementary crystallographic data

[12] Sheldrick, G. M. SHELXL-2013, Bruker AXS Inc., Madison, Wisconsin, **2013**.

for this paper (CCDC 1557549) can also be obtained free of charge via www.ccdc.cam.ac.uk/conts/retrieving.html (or from the Cambridge Crystallographic Data Centre, 12, Union Road, Cambridge CB2 1EZ, UK; fax: +44 1223 336033; or deposit@ccdc.cam.ac.uk).

Table S1. Selected crystallographic data and collection parameters for **10a**.

formula	$C_{39}H_{38}N_2O_3S$
fw	614.77
crystal system	monoclinic
space group	P21/c
a, Å	16.5942(7)
b, Å	8.7199(4)
c, Å	21.4742(10)
β, deg	93.5160(10)
volume, Å^3	3101.5(2)
Z	4
D (calcd), Mg m^{-3}	1.317
μ, mm^{-1}	0.147
F(000)	1304
crystal size, mm	0.25 x 0.2 x 0.05
θ range for data collection, deg	2.33 to 25.04
index ranges	-19≤h≤17, -10≤k≤10, -24≤l≤25
reflections collected	20548
independent reflections [R(int)]	5453 [R(int) = 0.0258]
coverage of independent reflections	99.4%
max. and min. transmission	0.9930/0.9640
data / restraints / parameters	5453 / 0 / 410
goodness-of-fit on F^2	1.038
R_1, wR_2 [$I > 2\sigma(I)$]/	0.0371, 0.0940
R_1, wR_2 (all data)	0.0455, 0.0992
Weighting scheme	$w = 1/[\sigma^2(F_o^2) + (0.0454P)^2]$
	Where $P = (F_o^2 + 2F_c^2)/3$
extinction coefficient	0.0058(5)
largest diff. peak and hole, e Å^{-3}	0.400 and -0.756
R.M.S. deviation from mean, e Å^{-3}	0. 048

7. DFT Calculations

The Gaussian 09 program package was used for all geometry optimizations.[13] The geometries of the stationary points and transition states were fully optimized using the Becke's three-parameter hybrid density functional method (B3LYP)[14] with a double-ζ basis set with the relativistic effective core potential of Hay and Wadt (LanL2DZ)[15] for PD, K, Br, and I and the 6-31G(d)[16] basis sets for other elements. The vibrational frequencies and thermal correction to Gibbs free energy (TCGFE) including zero-point energy were calculated at the same level of theory. The obtained structures were characterized by the number of imaginary frequencies (IF, one or zero for transition or ground states, respectively). The connectivity of each step was also confirmed by intrinsic reaction coordinate (IRC) calculation[17] from the transition states followed by optimization of the resultant geometries. Single-point energies for geometries obtained by the above method were calculated using the Truhlar's M06L functional[18] with the basis sets including the Stuttgart-Dresden-Bonn energy-consistent pseudopotential (SDD)[19] for Pd, K, Br, and I, and the 6-311++G(d,p) basis sets[20] for other elements. To examine the solvent effect, the above single-point energy calculations were performed using the polarizable continuum model (PCM)[21] method with

---

13 Gaussian 09, Revision C.01, Frisch, M. J.; Trucks, G. W.; Schlegel, H. B.; Scuseria, G. E.; Robb, M. A.; Cheeseman, J. R.; Scalmani, G.; Barone, V.; Mennucci, B.; Petersson, G. A.; Nakatsuji, H.; Caricato, M.; Li, X.; Hratchian, H. P.; Izmaylov, A. F.; Bloino, J.; Zheng, G.; Sonnenberg, J. L.; Hada, M.; Ehara, M.; Toyota, K.; Fukuda, R.; Hasegawa, J.; Ishida, M.; Nakajima, T.; Honda, Y.; Kitao, O.; Nakai, H.; Vreven, T.; Montgomery, Jr., J. A.; Peralta, J. E.; Ogliaro, F.; Bearpark, M.; Heyd, J. J.; Brothers, E.; Kudin, K. N.; Staroverov, V. N.; Kobayashi, R.; Normand, J.; Raghavachari, K.; Rendell, A.; Burant, J. C.; Iyengar, S. S.; Tomasi, J.; Cossi, M.; Rega, N.; Millam, J. M.; Klene, M.; Knox, J. E.; Cross, J. B.; Bakken, V.; Adamo, C.; Jaramillo, J.; Gomperts, R.; Stratmann, R. E.; Yazyev, O.; Austin, A. J.; Cammi, R.; Pomelli, C.; Ochterski, J. W.; Martin, R. L.; Morokuma, K.; Zakrzewski, V. G.; Voth, G. A.; Salvador, P.; Dannenberg, J. J.; Dapprich, S.; Daniels, A. D.; Farkas, Ö.; Foresman, J. B.; Ortiz, J. V.; Cioslowski, J.; Fox, D. J., Gaussian, Inc., Wallingford CT, 2009.

14 (a) Kohn, W.; Becke, A. D.; Parr, R. G. *J. Phys. Chem.* **1996**, *100*, 12974-12980. (b) Stephen, P. J.; Devlin, F. J.; Chabalowski, C. F.; Frisch, M. *J. Phys. Chem.* **1994**, *98*, 11623-11627. (c) Becke, A. D. *J. Chem. Phys.* **1993**, *98*, 5648-5652. (d) Lee, C.; Yang, W.; Parr, R. G. *Phys. Rev. B* **1988**, *37*, 785-789.

15 Hay, P. J.; Wadt, W. R. *J. Chem. Phys.* **1985**, *82*, 299-310.

16 (a) Hehre, W. J.; Ditchfield, R.; Pople, J. A. *J. Chem. Phys.* **1972**, *56*, 2257-2261. (b) Hariharan, P. C.; Pople, J. A. *Theor. Chim. Acta* **1973**, *28*, 213-222. (c) Fracl, M. M.; Pietro, W. J.; Hehre, W. J.; Binkley, J. S.; Gordon, M. S.; DeFrees, D. J.; Pople, J. A. *J. Chem. Phys.* **1982**, *77*, 3654-3665.

17 (a) Fukui, K. *Acc. Chem. Res.* **1981**, *14*, 363-368. (b) Gonzalez, C.; Schlegel, H. B. *J. Chem. Phys.* **1989**, *90*, 2154-2161. (c) Gonzalez, C.; Schlegel, H. B. *J. Phys. Chem.* **1990**, *94*, 5523-5527.

18 Zhao, Y.; Truhlar, D. G. *Acc. Chem. Res.* **2008**, *41*, 157-167.

19 Andrae, D.; Häussermann, U.; Dolg, M.; Stoll, H.; Preuß, H. *Theor. Chim. Acta* **1990**, *77*, 123-141.

20 (a) Krishnan, R.; Binkley, J. S.; Seeger, R.; Pople, J. A. *J. Chem. Phys.* **1980**, *72*, 650-654. (b) McLean, A. D.; Chandler, G. S. *J. Chem. Phys.* **1980**, *72*, 5639-5648. (c) Frisch, M. J.; Pople, J. A.; Binkley, J. S. *J. Chem. Phys.* **1984**, *80*, 3265-3269. (d) Clark, T.; Chandrasekhar, J.; Spitznagel, G. W.; Schleyer, P. v. R. *J. Comp. Chem.* **1983**, *4*, 294-301.

21 (a) Miertuš, S.; Scrocco, E.; Tomasi, J. *Chem. Phys.* **1981**, *55*, 117-129. (b) Pascual-Ahuir, J. L.; Silla, E.; Tomasi, J.; Bonaccorsi, R. *J. Comput. Chem.* **1987**, *8*, 778-787. (c) Floris, F.; Tomasi, J. *J. Comput. Chem.* **1989**, *10*, 616-627. (d) Cossi, M.; Barone, V.; Cammi, R.; Tomasi, J. *Chem. Phys. Lett.* **1996**, *255*, 327-335. (e) Cancès, E.; Mennucci, B.; Tomasi, J. *J. Chem. Phys.* **1997**, *22*, 3032-3041. (f)

dielectric constants (e) of 37.219 for DMF. The obtained energies, ZPEs, TCGFEs, and IF are summarized in Tables S1 and S2.

Table S1. Summary of theoretical calculations.

Model	Energy/au	TCGFE/au	IF/cm^{-1}
**A**	–903.92380121	0.208200	
**TS$_{AB}$**	–903.91752127	0.208824	90.6105i
**B**	–903.95240808	0.208546	
**C**	–928.15699874	0.262028	
**TS$_{CD}$**	–928.15480827	0.264639	186.7456i
**D**	–928.19347245	0.267841	
**E**	–1185.15306660	0.304747	
**TS$_{EF}$**	–1185.12721816	0.302948	1158.9234i
**F**	–1185.15737748	0.307417	
**G**	–1275.72843281	0.37030	
**TS$_{GH}$**	–1275.71791518	0.374412	183.3263i
**H**	–1275.76040296	0.375902	
**TS$_{HI}$**	–1275.74269491	0.376440	293.3224i
**I**	–1275.77716853	0.376514	
**TS$_{IJ}$**	–1275.74207050	0.371758	216.5692i
**J**	–1275.76300169	0.368791	
**K**	–1259.96674081	0.264666	
**TS$_{KL}$**	–1259.95703610	0.261685	183.1084i
**L**	–1259.96373424	0.262421	
**M**	–1259.94610607	0.263780	18.4857i
**N**	–1259.94765157	0.262201	
**O**	–1237.51654708	0.309351	
**TS$_{OP}$**	–1237.48926672	0.307520	285.5029i
**P**	–1237.53496141	0.308553	
**DMF**	–248.55653071	0.073913	
**NBE**	–272.75700877	0.125076	
**KOAc**	–256.95553030	0.015930	
**3q**	–359.58744560	0.087729	
**AcOH•KI**	–269.01218346	0.026055	
**AcOH•KBr**	–270.98715185	0.026425	

Barone, V.; Cossi, M.; Tomasi, J. *J. Chem. Phys.* **1997**, *22*, 3210-3221.

Table S2. Summary of theoretical calculations.

Model	Energy/au	TCGFE/au	IF/cm^{-1}
**a**	–1180.74998304	0.282833	
**TS$_{ab}$**	–1180.66655689	0.276263	1452.2522i
**b**	–1180.74762287	0.281407	
**sG**	–1354.36647709	0.426254	
**TS$_{sGH}$**	–1354.35771842	0.428265	177.35333i
**sH**	–1354.40327149	0.430054	
**TS$_{sHI}$**	–1354.38681257	0.430146	284.8040i
**sI**	–1354.41635366	0.429870	
**TS$_{sIJ}$**	–1354.38036698	0.425249	218.9568i
**sJ**	–1354.39827864	0.423347	
**sK**	–1338.60414126	0.317568	
**TS$_{sKL}$**	–1338.59692906	0.315178	158.5032i
**sL**	–1338.60227920	0.317204	
**sN**	–1338.58196257	0.317234	120.4171i*
**sO**	–1316.15156516	0.362564	
**TS$_{sOP}$**	–1316.13181474	0.361192	249.2817i
**sP**	–1316.18100897	0.359778	
**3a**	–438.22767274	0.143038	

*Residual imaginary frequency due to the twist motion of one of two methyl groups at the benzylic position.

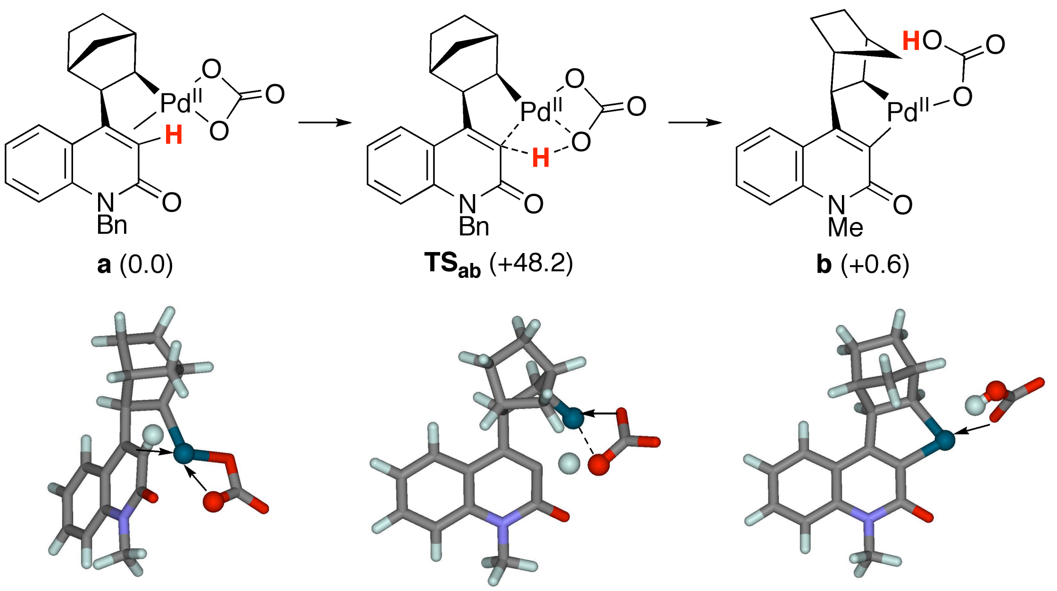

Scheme S1 CMD step of model complex **a** leading to palladacycle complex **b**. Relative Gibbs free energies in DMF at 298 K, 1 atm are indicated in parentheses.

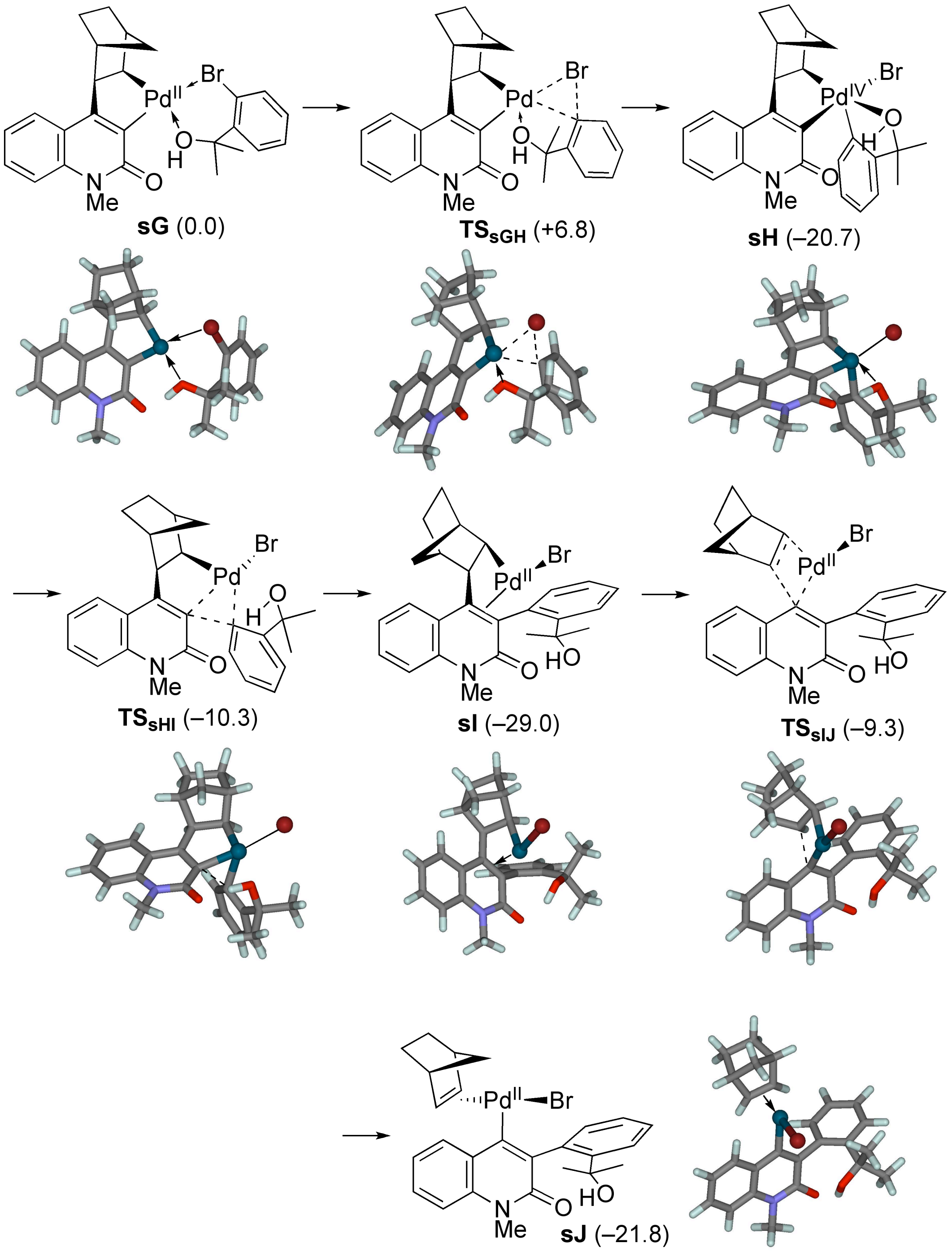

Scheme S2 Oxidative addition/reductive elimination steps of model complex **sG** and subsequent deinsertion of NBE from intermediate complex **sI**. Relative Gibbs free energies in DMF at 298 K, 1 atm are indicated in parentheses.

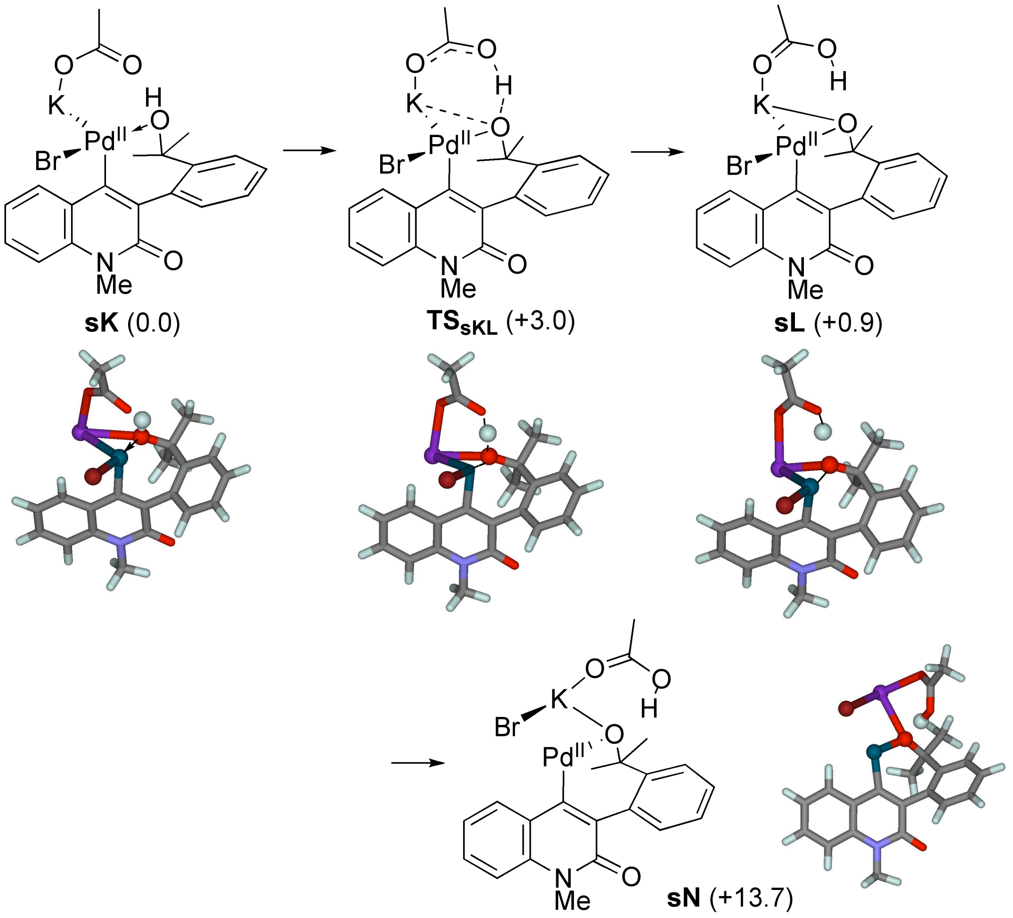

Scheme S3 Deprotonation of benzylic alcohol of model complex **sK** and subsequent bromide dissociation from intermediate complex **sL**. Relative Gibbs free energies in DMF at 298 K, 1 atm are indicated in parentheses.

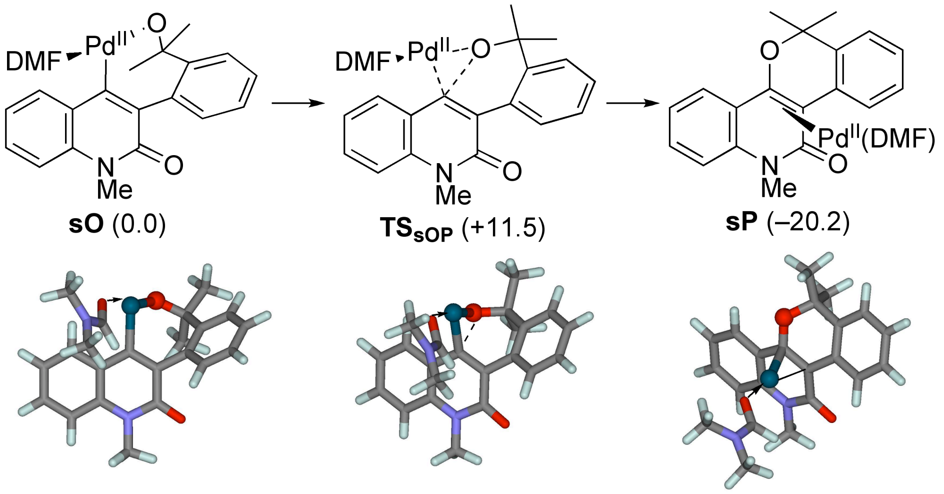

Scheme S4 Reductive elimination from model complex **sO** afforting the final product complex **sP**. Relative Gibbs free energies in DMF at 298 K, 1 atm are indicated in parentheses.

Standard orientations

A

Center Number	Atomic Number	Atomic Type	Coordinates (Angstroms) X	Y	Z
1	6	0	0.919466	0.092290	-0.964743
2	6	0	0.865256	-1.246274	-1.560230
3	6	0	1.611929	0.302746	0.244330
4	6	0	0.182617	-1.539225	-2.751856
5	6	0	1.517888	-2.305026	-0.863569
6	6	0	2.280009	-0.803272	0.948308
7	1	0	1.965227	1.283589	0.540469
8	6	0	0.127863	-2.828353	-3.264872
9	1	0	-0.307926	-0.726210	-3.275881
10	6	0	1.453691	-3.606532	-1.397132
11	6	0	0.769513	-3.863160	-2.580361
12	1	0	-0.406821	-3.023726	-4.189574
13	1	0	1.942585	-4.426246	-0.886298
14	1	0	0.741523	-4.878141	-2.967303
15	8	0	2.901392	-0.636908	1.996204
16	7	0	2.206125	-2.064075	0.331680
17	46	0	-0.535838	0.298205	0.482570
18	53	0	0.750889	1.832966	-2.320223
19	8	0	-2.213404	0.440621	1.895252
20	6	0	-2.136069	0.396996	3.131613
21	7	0	-3.192224	0.504701	3.959319
22	6	0	-4.539634	0.690430	3.434639
23	1	0	-5.185694	-0.137005	3.750878
24	1	0	-4.963086	1.630415	3.807614
25	1	0	-4.488184	0.719026	2.346729
26	6	0	-3.032199	0.447561	5.404246
27	1	0	-3.382735	1.377963	5.867171
28	1	0	-3.607205	-0.387603	5.822087
29	1	0	-1.977951	0.307292	5.655353
30	1	0	-1.175004	0.264371	3.646685
31	6	0	2.886424	-3.162986	1.011367
32	1	0	2.170895	-3.933883	1.320289
33	1	0	3.639470	-3.616536	0.357965
34	1	0	3.370322	-2.741525	1.890307

TS_{AB}

Center Number	Atomic Number	Atomic Type	Coordinates (Angstroms) X	Y	Z
1	6	0	2.014595	-0.076452	-0.747475
2	6	0	1.963876	-1.326211	-1.495346
3	6	0	2.926093	0.097366	0.280243
4	6	0	1.077296	-1.562197	-2.560588
5	6	0	2.844660	-2.367911	-1.082210
6	6	0	3.803799	-0.980184	0.725574
7	1	0	3.123927	1.063577	0.726250
8	6	0	1.044885	-2.781425	-3.221016
9	1	0	0.419591	-0.756238	-2.866892
10	6	0	2.798953	-3.601095	-1.764579
11	6	0	1.913477	-3.800666	-2.816163
12	1	0	0.355672	-2.937499	-4.045298
13	1	0	3.459690	-4.407457	-1.472719
14	1	0	1.904883	-4.760652	-3.325296
15	8	0	4.581713	-0.863839	1.671338
16	7	0	3.725431	-2.177490	-0.016981
17	46	0	0.455374	0.341148	0.420053
18	53	0	1.398939	1.903697	-1.911590
19	8	0	-1.294586	0.636430	1.768625
20	6	0	-1.273499	0.606489	3.006262
21	7	0	-2.357823	0.774011	3.787685
22	6	0	-3.671293	1.013881	3.202523
23	1	0	-4.366879	0.218679	3.495795
24	1	0	-4.068686	1.975047	3.549309
25	1	0	-3.570205	1.030046	2.117809
26	6	0	-2.263265	0.726970	5.238407
27	1	0	-2.593273	1.676626	5.676753
28	1	0	-2.890690	-0.078198	5.639651
29	1	0	-1.227797	0.545165	5.536911
30	1	0	-0.342924	0.436498	3.566662
31	6	0	4.622596	-3.256318	0.388531
32	1	0	4.054027	-4.132707	0.719983
33	1	0	5.277813	-3.547422	-0.439572
34	1	0	5.221390	-2.877065	1.214449

B

Center Number	Atomic Number	Atomic Type	Coordinates (Angstroms) X	Y	Z
1	6	0	0.988298	-1.245600	0.676459
2	6	0	0.956630	-1.789990	-0.662823
3	6	0	2.052909	-1.451180	1.491074
4	6	0	-0.090734	-1.548942	-1.572630
5	6	0	2.044845	-2.620406	-1.062328
6	6	0	3.171643	-2.313591	1.111324
7	1	0	2.143530	-0.998213	2.472737
8	6	0	-0.088016	-2.106470	-2.841911
9	1	0	-0.896940	-0.892896	-1.260235
10	6	0	2.030635	-3.183908	-2.354718
11	6	0	0.978852	-2.928809	-3.225259
12	1	0	-0.901098	-1.901779	-3.531702
13	1	0	2.844814	-3.818473	-2.681520
14	1	0	0.995092	-3.372966	-4.216950
15	8	0	4.121563	-2.547946	1.855161
16	7	0	3.097155	-2.865410	-0.184416
17	46	0	-0.602591	-0.531658	1.563791
18	53	0	-0.084038	1.879400	0.592818
19	8	0	-2.417451	-0.160926	2.798945
20	6	0	-2.887803	0.959479	3.062469
21	7	0	-3.957871	1.158804	3.847996
22	6	0	-4.660701	0.039748	4.464632
23	1	0	-4.644460	0.140486	5.556066
24	1	0	-5.703937	0.018454	4.128425
25	1	0	-4.164443	-0.885924	4.175315
26	6	0	-4.459467	2.499416	4.115179
27	1	0	-5.494600	2.597607	3.766995
28	1	0	-4.431201	2.711227	5.190614
29	1	0	-3.842380	3.235916	3.595412
30	1	0	-2.440012	1.873627	2.651392
31	6	0	4.211628	-3.716519	-0.596942
32	1	0	3.859876	-4.725778	-0.836711
33	1	0	4.717293	-3.294945	-1.472324
34	1	0	4.904769	-3.758932	0.241019

C

Center Number	Atomic Number	Atomic Type	Coordinates (Angstroms)		
			X	Y	Z
1	6	0	-2.062091	-2.365957	3.123496
2	6	0	-0.868482	-1.461334	2.659942
3	6	0	-2.690269	-0.226689	2.096849
4	6	0	-3.316227	-1.520056	2.732365
5	1	0	-2.046967	-3.352010	2.648328
6	1	0	-2.005796	-2.523410	4.205504
7	1	0	-3.986361	-2.042496	2.042545
8	1	0	-3.905029	-1.242407	3.612627
9	6	0	-1.438320	-0.063403	2.986635
10	1	0	-0.792976	0.763515	2.673392
11	1	0	-1.687196	0.053251	4.046512
12	6	0	-0.960069	-1.496407	1.141800
13	1	0	-0.614987	-2.348100	0.566026
14	6	0	-2.091837	-0.729583	0.794288
15	1	0	-2.693974	-0.887033	-0.095934
16	1	0	0.100072	-1.728084	3.084704
17	1	0	-3.367602	0.622468	2.012989
18	6	0	1.287376	-0.724899	0.172525
19	6	0	1.660927	-1.574077	-0.935215
20	6	0	2.169658	-0.413191	1.150374
21	6	0	0.768317	-1.955172	-1.956480
22	6	0	3.012192	-2.027862	-0.989787
23	6	0	3.568315	-0.847195	1.102448
24	1	0	1.912420	0.177525	2.023076
25	6	0	1.171440	-2.769004	-3.003689
26	1	0	-0.251781	-1.584130	-1.912930
27	6	0	3.407089	-2.852662	-2.062997
28	6	0	2.498248	-3.215568	-3.048494
29	1	0	0.468204	-3.050542	-3.781146
30	1	0	4.426796	-3.210804	-2.127649
31	1	0	2.830336	-3.852622	-3.863501
32	8	0	4.387175	-0.551331	1.967715
33	7	0	3.915662	-1.654648	0.000166
34	46	0	-0.369554	0.417332	0.071757
35	53	0	-1.714645	2.596302	-0.697950
36	6	0	5.305596	-2.106279	-0.067320
37	1	0	5.360962	-3.199349	-0.026286
38	1	0	5.779218	-1.755482	-0.989994
39	1	0	5.819713	-1.682659	0.793179

TS_{CD}

Center Number	Atomic Number	Atomic Type	Coordinates (Angstroms) X	Y	Z
1	6	0	1.064080	0.053192	0.786232
2	6	0	2.295785	0.920178	0.360058
3	6	0	0.523714	2.255011	-0.131040
4	6	0	-0.153819	0.973940	0.459716
5	1	0	1.028407	-0.906903	0.260986
6	1	0	1.128037	-0.164989	1.857084
7	1	0	-0.861399	0.515091	-0.238229
8	1	0	-0.711624	1.237686	1.364241
9	6	0	1.795684	2.330961	0.742392
10	1	0	2.471458	3.139710	0.447984
11	1	0	1.571273	2.420817	1.810213
12	6	0	2.214482	0.945855	-1.161645
13	1	0	2.389656	0.037649	-1.729483
14	6	0	1.086590	1.795391	-1.468628
15	1	0	0.416583	1.589331	-2.301123
16	1	0	3.247484	0.586637	0.774503
17	1	0	-0.113523	3.137990	-0.173078
18	6	0	4.128987	1.587176	-1.926069
19	6	0	4.558551	0.794481	-3.063070
20	6	0	5.009153	1.929649	-0.950755
21	6	0	3.688052	0.386670	-4.093047
22	6	0	5.935090	0.431675	-3.137096
23	6	0	6.430146	1.589249	-1.018521
24	1	0	4.722716	2.479101	-0.061707
25	6	0	4.137219	-0.367358	-5.166038
26	1	0	2.646918	0.691847	-4.041861
27	6	0	6.376863	-0.334832	-4.235053
28	6	0	5.489307	-0.726657	-5.228454
29	1	0	3.449181	-0.668764	-5.949506
30	1	0	7.417196	-0.624085	-4.312777
31	1	0	5.857535	-1.316920	-6.062829
32	8	0	7.234111	1.921024	-0.151753
33	7	0	6.820795	0.832919	-2.141214
34	46	0	2.617096	2.960632	-2.265274
35	53	0	1.290372	5.078860	-3.154885

36	6	0	8.235762	0.471199	-2.227036
37	1	0	8.358434	-0.617055	-2.219481
38	1	0	8.682114	0.878328	-3.140121
39	1	0	8.727000	0.900398	-1.355996

D

Center Number	Atomic Number	Atomic Type	Coordinates (Angstroms) X	Y	Z
1	6	0	-2.349244	-2.361252	2.748576
2	6	0	-0.870690	-1.901679	2.672451
3	6	0	-2.006892	-0.036349	2.035731
4	6	0	-3.131124	-1.107745	2.231954
5	1	0	-2.541785	-3.259501	2.151908
6	1	0	-2.618624	-2.594150	3.784141
7	1	0	-3.684245	-1.303058	1.307028
8	1	0	-3.855330	-0.749439	2.971325
9	6	0	-1.027555	-0.436778	3.156908
10	1	0	-0.118598	0.166833	3.166329
11	1	0	-1.482444	-0.386836	4.152408
12	6	0	-0.557456	-1.782434	1.149216
13	1	0	-0.992208	-2.639618	0.628481
14	6	0	-1.258523	-0.457232	0.778804
15	1	0	-1.824199	-0.435133	-0.153149
16	1	0	-0.170208	-2.534331	3.224686
17	1	0	-2.364097	0.993361	2.026430
18	6	0	0.900022	-1.614950	0.696866
19	6	0	1.382024	-2.197479	-0.554927
20	6	0	1.799277	-0.924697	1.493790
21	6	0	0.544974	-2.897233	-1.446844
22	6	0	2.761291	-2.055420	-0.888642
23	6	0	3.222965	-0.782094	1.165277
24	1	0	1.566811	-0.641235	2.510864
25	6	0	1.030571	-3.456249	-2.618946
26	1	0	-0.511687	-2.990167	-1.225076
27	6	0	3.241613	-2.632198	-2.080180
28	6	0	2.387504	-3.322151	-2.930000
29	1	0	0.360546	-3.987230	-3.287652
30	1	0	4.286049	-2.537437	-2.346739
31	1	0	2.784289	-3.754591	-3.844039

32	8	0	4.011875	-0.210943	1.911459
33	7	0	3.628004	-1.352472	-0.050212
34	46	0	0.433878	0.643947	0.347875
35	53	0	-0.796040	2.763606	-0.610509
36	6	0	5.041609	-1.209479	-0.402660
37	1	0	5.523928	-2.189952	-0.473354
38	1	0	5.147067	-0.683611	-1.356934
39	1	0	5.510111	-0.629651	0.389962

E

Center Number	Atomic Number	Atomic Type	Coordinates (Angstroms) X	Y	Z
1	6	0	-3.667608	-2.428525	1.630709
2	6	0	-2.121739	-2.359926	1.516690
3	6	0	-2.745278	-0.227711	1.030666
4	6	0	-4.109180	-0.983432	1.221219
5	1	0	-4.110249	-3.203509	0.994302
6	1	0	-3.958115	-2.649993	2.662728
7	1	0	-4.732017	-0.967134	0.319808
8	1	0	-4.680263	-0.492418	2.016274
9	6	0	-1.872697	-0.940925	2.082549
10	1	0	-0.835553	-0.609322	2.137581
11	1	0	-2.295066	-0.842974	3.089879
12	6	0	-1.842319	-2.230164	-0.012720
13	1	0	-2.514499	-2.902360	-0.547818
14	6	0	-2.208927	-0.758915	-0.285572
15	1	0	-2.802224	-0.547561	-1.179937
16	1	0	-1.590317	-3.183903	2.000045
17	1	0	-2.826768	0.858096	1.089218
18	6	0	-0.421358	-2.410216	-0.571069
19	6	0	-0.171827	-3.241108	-1.749898
20	6	0	0.661700	-1.792359	0.050498
21	6	0	-1.191106	-3.923250	-2.446675
22	6	0	1.171705	-3.419052	-2.194026
23	6	0	2.012492	-1.830780	-0.534614
24	1	0	0.645898	-1.382003	1.063113
25	6	0	-0.917266	-4.762491	-3.515820
26	1	0	-2.223894	-3.793075	-2.148257
27	6	0	1.438575	-4.279357	-3.275135

28	6	0	0.407006	-4.943048	-3.925246
29	1	0	-1.727666	-5.271900	-4.027584
30	1	0	2.455015	-4.425397	-3.616295
31	1	0	0.639003	-5.599148	-4.759136
32	8	0	2.918515	-1.090293	-0.134408
33	7	0	2.218277	-2.722184	-1.582492
34	46	0	-0.382729	-0.056178	-0.952701
35	19	0	2.420050	1.407547	0.998171
36	8	0	2.963109	0.792465	3.491990
37	6	0	1.999414	-0.012357	3.547764
38	8	0	1.113601	-0.157845	2.638424
39	53	0	-0.617382	2.540494	-1.447450
40	6	0	1.851446	-0.897421	4.792193
41	1	0	0.892120	-0.688796	5.280724
42	1	0	1.837291	-1.953640	4.497862
43	1	0	2.665261	-0.725117	5.500276
44	6	0	3.583511	-2.840005	-2.100824
45	1	0	3.925353	-3.877315	-2.037143
46	1	0	3.631763	-2.504499	-3.141937
47	1	0	4.217669	-2.205740	-1.485742

TS_{EF}

Center Number	Atomic Number	Atomic Type	Coordinates (Angstroms) X	Y	Z
1	6	0	0.554381	0.736902	-0.019335
2	6	0	2.097371	0.644558	0.069580
3	6	0	1.720309	2.881882	0.212373
4	6	0	0.290533	2.278398	0.038972
5	1	0	0.155885	0.272800	-0.929067
6	1	0	0.094139	0.225565	0.833341
7	1	0	-0.214675	2.657055	-0.856064
8	1	0	-0.332395	2.545024	0.900041
9	6	0	2.403520	1.789198	1.062798
10	1	0	3.464366	1.971842	1.242277
11	1	0	1.906455	1.635346	2.028320
12	6	0	2.663061	1.199469	-1.278606
13	1	0	2.061365	0.813462	-2.111936
14	6	0	2.415295	2.734199	-1.144304
15	1	0	1.865291	3.167750	-1.987937

16	1	0	2.476184	-0.346801	0.335289
17	1	0	1.721946	3.898037	0.611250
18	6	0	4.111021	0.928142	-1.599986
19	6	0	4.548765	-0.262401	-2.288701
20	6	0	5.017899	1.929098	-1.295404
21	6	0	3.689172	-1.352056	-2.552210
22	6	0	5.908238	-0.348398	-2.711895
23	6	0	6.392085	1.868744	-1.800688
24	1	0	5.155157	2.371555	-0.031992
25	6	0	4.138337	-2.493007	-3.194500
26	1	0	2.654605	-1.290190	-2.231951
27	6	0	6.350224	-1.512101	-3.371565
28	6	0	5.476867	-2.565799	-3.604376
29	1	0	3.460600	-3.320295	-3.380812
30	1	0	7.377759	-1.596729	-3.701226
31	1	0	5.842796	-3.453362	-4.112838
32	8	0	7.192050	2.804857	-1.629822
33	7	0	6.782474	0.713999	-2.483260
34	46	0	4.123915	3.831501	-1.343789
35	19	0	7.020809	4.917516	0.182812
36	8	0	7.717740	2.950862	1.948983
37	6	0	6.751110	2.253476	1.620550
38	8	0	5.654343	2.772304	1.120198
39	53	0	4.022230	6.559048	-1.411085
40	6	0	6.766959	0.744385	1.808778
41	1	0	6.039323	0.468826	2.581066
42	1	0	6.461864	0.236620	0.886745
43	1	0	7.759463	0.404374	2.110016
44	6	0	8.155828	0.655746	-2.988831
45	1	0	8.702963	-0.171152	-2.524480
46	1	0	8.158307	0.527242	-4.075795
47	1	0	8.632387	1.598597	-2.730384

F

Center Number	Atomic Number	Atomic Type	Coordinates (Angstroms) X	Y	Z
1	6	0	-4.167163	-2.316256	0.813537
2	6	0	-2.632667	-2.512543	0.824629
3	6	0	-2.863177	-0.325419	1.388033

4	6	0	-4.330525	-0.802587	1.170744
5	1	0	-4.611506	-2.574103	-0.154744
6	1	0	-4.641142	-2.953282	1.569056
7	1	0	-4.834989	-0.231845	0.383741
8	1	0	-4.912442	-0.670723	2.090354
9	6	0	-2.223611	-1.591736	1.994153
10	1	0	-1.140130	-1.504386	2.119529
11	1	0	-2.672693	-1.894608	2.947645
12	6	0	-2.055246	-1.759765	-0.421064
13	1	0	-2.685657	-1.976371	-1.296571
14	6	0	-2.195425	-0.257116	0.006807
15	1	0	-2.753733	0.350207	-0.715970
16	1	0	-2.315342	-3.556380	0.900524
17	1	0	-2.788809	0.596415	1.969981
18	6	0	-0.619514	-2.034033	-0.762133
19	6	0	-0.141316	-3.278259	-1.324953
20	6	0	0.237987	-0.984414	-0.553508
21	6	0	-0.986684	-4.373473	-1.601413
22	6	0	1.242217	-3.396059	-1.634686
23	6	0	1.654692	-1.106872	-0.854177
24	1	0	0.766838	0.314416	2.204901
25	6	0	-0.497723	-5.548916	-2.149922
26	1	0	-2.044979	-4.283016	-1.380953
27	6	0	1.729340	-4.596201	-2.187915
28	6	0	0.868464	-5.657139	-2.438991
29	1	0	-1.169531	-6.377313	-2.354575
30	1	0	2.779893	-4.703564	-2.427300
31	1	0	1.265648	-6.573231	-2.867176
32	8	0	2.472308	-0.180893	-0.641266
33	7	0	2.099909	-2.319275	-1.387060
34	46	0	-0.435796	0.788542	0.037644
35	19	0	3.038496	2.036238	0.654631
36	8	0	3.749091	-0.072696	2.598746
37	6	0	2.574258	-0.380729	2.621719
38	8	0	1.654811	0.609490	2.517860
39	53	0	-0.186318	3.525234	0.583372
40	6	0	2.047323	-1.784338	2.788007
41	1	0	1.455285	-1.853774	3.707671
42	1	0	1.391302	-2.045796	1.950143
43	1	0	2.881909	-2.484415	2.837010
44	6	0	3.522527	-2.443302	-1.706064
45	1	0	3.976404	-3.259607	-1.134377
46	1	0	3.661603	-2.633801	-2.775337

47	1	0	3.997474	-1.502789	-1.438139

G

Center Number	Atomic Number	Atomic Type	Coordinates (Angstroms)		
			X	Y	Z
1	6	0	-3.050451	-2.453386	2.858778
2	6	0	-1.545351	-2.463667	2.501386
3	6	0	-2.056156	-0.257886	2.457586
4	6	0	-3.416134	-0.933724	2.798338
5	1	0	-3.647275	-3.060919	2.168156
6	1	0	-3.208355	-2.856352	3.866020
7	1	0	-4.183168	-0.712346	2.047560
8	1	0	-3.794906	-0.578103	3.764348
9	6	0	-1.062916	-1.175577	3.200890
10	1	0	-0.017667	-0.927562	2.994568
11	1	0	-1.221218	-1.203070	4.286204
12	6	0	-1.421724	-2.097797	0.983241
13	1	0	-2.190206	-2.650377	0.421622
14	6	0	-1.720331	-0.556793	0.981496
15	1	0	-2.541124	-0.293975	0.301492
16	1	0	-1.027039	-3.385230	2.780775
17	1	0	-2.027150	0.803856	2.723477
18	6	0	-0.081203	-2.350884	0.357706
19	6	0	0.413968	-3.681282	0.070877
20	6	0	0.652485	-1.239717	0.025804
21	6	0	-0.318934	-4.852714	0.357720
22	6	0	1.689751	-3.816379	-0.539351
23	6	0	1.945465	-1.378864	-0.629288
24	6	0	0.181587	-6.112890	0.069265
25	1	0	-1.298275	-4.756652	0.813676
26	6	0	2.192683	-5.101380	-0.824098
27	6	0	1.446118	-6.232500	-0.521031
28	1	0	-0.403069	-6.998784	0.299674
29	1	0	3.164020	-5.220403	-1.287317
30	1	0	1.851807	-7.213978	-0.750661
31	8	0	2.653151	-0.411468	-1.004698
32	7	0	2.421348	-2.671606	-0.858218
33	46	0	-0.131650	0.561912	0.366734
34	6	0	3.736446	-2.810669	-1.486604

35	1	0	4.406116	-3.390110	-0.842922
36	1	0	3.648735	-3.311247	-2.456902
37	1	0	4.136962	-1.810364	-1.628479
38	35	0	-1.338760	3.160825	0.141885
39	6	0	-0.695356	3.163175	-1.740565
40	6	0	-1.661703	3.236825	-2.735295
41	6	0	0.674823	3.072683	-2.002188
42	6	0	-1.249572	3.223944	-4.070358
43	6	0	1.050046	3.058831	-3.353524
44	6	0	0.107215	3.133593	-4.378837
45	1	0	-1.993343	3.281096	-4.859858
46	1	0	2.107547	2.985744	-3.595263
47	1	0	0.432605	3.118840	-5.414932
48	6	0	1.729994	3.012560	-0.913887
49	1	0	2.719901	3.133096	-1.370704
50	1	0	1.582597	3.843890	-0.216571
51	1	0	-2.713083	3.301290	-2.478091
52	8	0	1.708547	1.826765	-0.127474
53	1	0	2.168824	1.053781	-0.580368

TS_{GH}

Center Number	Atomic Number	Atomic Type	Coordinates (Angstroms) X	Y	Z
1	6	0	-3.690517	-2.297742	1.183567
2	6	0	-2.174920	-2.392064	0.891868
3	6	0	-2.507181	-0.159680	1.093279
4	6	0	-3.929808	-0.757620	1.299066
5	1	0	-4.295569	-2.763720	0.396959
6	1	0	-3.932315	-2.806591	2.123660
7	1	0	-4.645264	-0.380935	0.559771
8	1	0	-4.315150	-0.487448	2.289239
9	6	0	-1.625038	-1.238559	1.755422
10	1	0	-0.552757	-1.063252	1.622263
11	1	0	-1.834193	-1.374051	2.823211
12	6	0	-1.940610	-1.863356	-0.566083
13	1	0	-2.705082	-2.289482	-1.232282
14	6	0	-2.147161	-0.318942	-0.397137
15	1	0	-2.918006	0.080765	-1.062435
16	1	0	-1.743351	-3.378949	1.078054

17	1	0	-2.408991	0.858603	1.475064
18	6	0	-0.578051	-2.147054	-1.125438
19	6	0	-0.161802	-3.481922	-1.506406
20	6	0	0.263088	-1.074651	-1.280017
21	6	0	-1.009361	-4.606727	-1.415656
22	6	0	1.152065	-3.665695	-2.013312
23	6	0	1.605207	-1.264170	-1.814171
24	6	0	-0.584242	-5.869889	-1.795767
25	1	0	-2.018477	-4.472944	-1.042098
26	6	0	1.577818	-4.954640	-2.391710
27	6	0	0.718632	-6.039583	-2.281384
28	1	0	-1.256493	-6.719164	-1.717465
29	1	0	2.577087	-5.110989	-2.777580
30	1	0	1.066167	-7.024510	-2.580615
31	8	0	2.424890	-0.334611	-2.000165
32	7	0	1.998856	-2.565009	-2.136255
33	46	0	-0.457540	0.769700	-0.841114
34	6	0	3.359019	-2.755868	-2.646192
35	1	0	3.909863	-3.452647	-2.006464
36	1	0	3.334364	-3.147726	-3.668508
37	1	0	3.848532	-1.785644	-2.639984
38	35	0	-1.641354	3.161600	-0.697789
39	6	0	-1.032076	2.065544	-2.638033
40	6	0	-2.126678	1.706396	-3.416020
41	6	0	0.192580	2.473509	-3.181988
42	6	0	-1.972171	1.682887	-4.805511
43	6	0	0.311865	2.430592	-4.575607
44	6	0	-0.756760	2.044925	-5.387891
45	1	0	-2.814125	1.387300	-5.425883
46	1	0	1.265797	2.702045	-5.022659
47	1	0	-0.640199	2.029126	-6.467434
48	6	0	1.335673	2.877601	-2.281079
49	1	0	2.265401	2.962473	-2.856675
50	1	0	1.129144	3.850782	-1.823857
51	1	0	-3.075005	1.450797	-2.958422
52	8	0	1.505334	1.957401	-1.198422
53	1	0	1.974469	1.134643	-1.533692

H

Center Number　Atomic Number　Atomic Type　Coordinates (Angstroms)

X　Y　Z

1	6	0	-3.174281	-2.038877	2.461118
2	6	0	-1.670505	-2.182263	2.131923
3	6	0	-1.880979	0.037143	2.537080
4	6	0	-3.328141	-0.508684	2.728854
5	1	0	-3.813745	-2.395081	1.645090
6	1	0	-3.427493	-2.625371	3.351227
7	1	0	-4.044405	-0.023394	2.058285
8	1	0	-3.659950	-0.312880	3.754626
9	6	0	-1.041997	-1.139851	3.076176
10	1	0	0.037610	-1.016219	2.935255
11	1	0	-1.233090	-1.354789	4.133425
12	6	0	-1.428149	-1.537763	0.718615
13	1	0	-2.199748	-1.886425	0.018127
14	6	0	-1.596057	-0.013055	1.024914
15	1	0	-2.354226	0.495631	0.429327
16	1	0	-1.286342	-3.201365	2.215039
17	1	0	-1.727882	1.016294	2.988504
18	6	0	-0.063013	-1.842376	0.165407
19	6	0	0.304991	-3.177659	-0.263050
20	6	0	0.835904	-0.814435	0.087247
21	6	0	-0.602121	-4.259120	-0.276547
22	6	0	1.632059	-3.402311	-0.716832
23	6	0	2.189237	-1.034886	-0.404362
24	6	0	-0.221722	-5.520405	-0.705378
25	1	0	-1.622180	-4.094614	0.051722
26	6	0	2.011835	-4.690914	-1.143009
27	6	0	1.095020	-5.732699	-1.133866
28	1	0	-0.939087	-6.335394	-0.708910
29	1	0	3.019777	-4.878737	-1.489761
30	1	0	1.407601	-6.717400	-1.469639
31	8	0	3.049691	-0.137408	-0.526472
32	7	0	2.537891	-2.343758	-0.743567
33	46	0	0.138365	1.032033	0.620513
34	6	0	3.914561	-2.581219	-1.189029
35	1	0	4.386510	-3.344189	-0.562724
36	1	0	3.929295	-2.909350	-2.233525
37	1	0	4.454527	-1.642642	-1.097014
38	35	0	-0.891165	3.279474	1.432840
39	6	0	-0.131581	1.322214	-1.357379
40	6	0	-1.087000	0.693070	-2.143635
41	6	0	0.761260	2.267862	-1.875235
42	6	0	-1.182222	1.039405	-3.496830

43	6	0	0.653591	2.585337	-3.235665
44	6	0	-0.312655	1.983755	-4.041594
45	1	0	-1.934203	0.559797	-4.117737
46	1	0	1.337938	3.317207	-3.660027
47	1	0	-0.379898	2.245674	-5.093763
48	6	0	1.762498	2.959465	-0.975593
49	1	0	2.682651	3.201775	-1.521961
50	1	0	1.330876	3.886897	-0.587701
51	1	0	-1.757040	-0.050651	-1.729651
52	8	0	2.072513	2.170122	0.179228
53	1	0	2.589802	1.363994	-0.111343

TS$_{HI}$

Center Number	Atomic Number	Atomic Type	Coordinates (Angstroms)		
			X	Y	Z
1	6	0	-3.123017	-2.105514	2.400610
2	6	0	-1.612909	-2.105091	2.073761
3	6	0	-1.998057	0.062067	2.593567
4	6	0	-3.404423	-0.601628	2.704375
5	1	0	-3.731849	-2.498937	1.577903
6	1	0	-3.318842	-2.732258	3.277963
7	1	0	-4.125895	-0.153650	2.012760
8	1	0	-3.800147	-0.464472	3.716930
9	6	0	-1.080753	-1.074081	3.084166
10	1	0	-0.014271	-0.851394	2.971850
11	1	0	-1.270250	-1.369042	4.122496
12	6	0	-1.443968	-1.363484	0.688071
13	1	0	-2.248727	-1.699432	0.017743
14	6	0	-1.641605	0.133495	1.100947
15	1	0	-2.380338	0.670129	0.501224
16	1	0	-1.144815	-3.090435	2.102293
17	1	0	-1.928070	1.017447	3.107883
18	6	0	-0.107277	-1.639052	0.045560
19	6	0	0.261660	-2.988439	-0.323264
20	6	0	0.760756	-0.592230	-0.212222
21	6	0	-0.653433	-4.067632	-0.295375
22	6	0	1.577927	-3.242035	-0.793551
23	6	0	2.155813	-0.873988	-0.574837
24	6	0	-0.290235	-5.339728	-0.703458

25	1	0	-1.668696	-3.889352	0.037612
26	6	0	1.942543	-4.542127	-1.193475
27	6	0	1.018075	-5.575700	-1.146520
28	1	0	-1.015949	-6.146850	-0.679821
29	1	0	2.943717	-4.744474	-1.551398
30	1	0	1.316549	-6.570628	-1.464078
31	8	0	3.046508	-0.005246	-0.643787
32	7	0	2.495341	-2.196424	-0.841914
33	46	0	0.110594	1.159279	0.760732
34	6	0	3.891553	-2.474648	-1.197515
35	1	0	4.303224	-3.234747	-0.527442
36	1	0	3.960449	-2.825714	-2.232201
37	1	0	4.448877	-1.548020	-1.092072
38	35	0	-0.625885	3.215692	2.106880
39	6	0	0.104778	0.869049	-1.344293
40	6	0	-0.912542	0.322224	-2.146251
41	6	0	0.898865	1.918715	-1.862978
42	6	0	-1.120414	0.767434	-3.447121
43	6	0	0.688287	2.329755	-3.187500
44	6	0	-0.302462	1.765221	-3.982888
45	1	0	-1.908926	0.318820	-4.045084
46	1	0	1.300034	3.139591	-3.580640
47	1	0	-0.443563	2.105321	-5.004796
48	6	0	1.847203	2.754584	-1.018422
49	1	0	2.798644	2.908313	-1.544415
50	1	0	1.392204	3.735622	-0.846556
51	1	0	-1.530140	-0.481192	-1.765834
52	8	0	2.088334	2.207584	0.274500
53	1	0	2.640654	1.398268	0.102622

I

Center Number	Atomic Number	Atomic Type	Coordinates (Angstroms)		
			X	Y	Z
1	6	0	-3.726060	-1.134625	1.040196
2	6	0	-2.426145	-1.981410	0.953893
3	6	0	-1.844393	-0.265269	2.323843
4	6	0	-3.330580	0.048244	1.977445
5	1	0	-4.070032	-0.803120	0.054149
6	1	0	-4.530024	-1.735958	1.477898

7	1	0	-3.453173	1.028315	1.504143
8	1	0	-3.929793	0.054364	2.894892
9	6	0	-1.864855	-1.805937	2.385788
10	1	0	-0.869698	-2.229123	2.545654
11	1	0	-2.542431	-2.190899	3.156194
12	6	0	-1.438724	-1.163017	0.078198
13	1	0	-1.962650	-0.815939	-0.815725
14	6	0	-1.078801	0.005465	1.035120
15	1	0	-1.183143	1.014816	0.630053
16	1	0	-2.631670	-2.992089	0.598970
17	1	0	-1.458995	0.258047	3.196746
18	6	0	-0.030073	-1.654656	-0.374116
19	6	0	0.544589	-2.998137	-0.196763
20	6	0	0.679723	-0.711987	-1.171317
21	6	0	-0.090316	-4.038026	0.515230
22	6	0	1.807573	-3.298370	-0.785670
23	6	0	2.015109	-1.052064	-1.731384
24	6	0	0.461932	-5.303856	0.639551
25	1	0	-1.032516	-3.850885	0.998147
26	6	0	2.359545	-4.586350	-0.656831
27	6	0	1.694890	-5.580063	0.046628
28	1	0	-0.066245	-6.068328	1.200914
29	1	0	3.315830	-4.814282	-1.107892
30	1	0	2.143028	-6.565594	0.133034
31	8	0	2.667441	-0.236567	-2.397988
32	7	0	2.515958	-2.317960	-1.486508
33	46	0	0.964174	-0.194460	1.024223
34	6	0	3.845326	-2.621490	-2.027717
35	1	0	4.515562	-2.935408	-1.222284
36	1	0	3.782602	-3.414045	-2.780193
37	1	0	4.222300	-1.712668	-2.489243
38	35	0	1.478353	0.608885	3.352413
39	6	0	0.009283	0.408488	-1.937219
40	6	0	-0.897322	-0.007895	-2.930180
41	6	0	0.290690	1.787213	-1.812131
42	6	0	-1.556107	0.897466	-3.757007
43	6	0	-0.379453	2.683749	-2.659125
44	6	0	-1.300746	2.260423	-3.612865
45	1	0	-2.250381	0.538560	-4.511615
46	1	0	-0.158001	3.744775	-2.568715
47	1	0	-1.797774	2.986640	-4.250057
48	6	0	1.249106	2.408937	-0.799926
49	1	0	1.564127	3.387259	-1.193419

50	1	0	0.726155	2.604294	0.144834
51	1	0	-1.079201	-1.072980	-3.055061
52	8	0	2.384482	1.634635	-0.455345
53	1	0	2.708503	1.200289	-1.272340

TS_{IJ}

Center Number	Atomic Number	Atomic Type	Coordinates (Angstroms)		
			X	Y	Z
1	6	0	-3.811738	-1.804687	0.624842
2	6	0	-2.320711	-2.146058	0.963758
3	6	0	-2.708040	-0.252338	2.160703
4	6	0	-4.074526	-0.496258	1.437730
5	1	0	-3.979250	-1.682451	-0.450323
6	1	0	-4.455915	-2.622792	0.963033
7	1	0	-4.368474	0.346935	0.804568
8	1	0	-4.865302	-0.638254	2.181762
9	6	0	-2.274076	-1.704522	2.444616
10	1	0	-1.288694	-1.760913	2.915085
11	1	0	-2.995224	-2.250522	3.062101
12	6	0	-1.546325	-1.044769	0.238911
13	1	0	-1.673029	-0.964328	-0.832100
14	6	0	-1.757575	0.149717	1.042931
15	1	0	-1.852979	1.129087	0.576361
16	1	0	-2.032024	-3.170127	0.725291
17	1	0	-2.745281	0.434844	3.005568
18	6	0	0.466358	-1.383921	-0.238335
19	6	0	1.007692	-2.697858	0.072486
20	6	0	0.825639	-0.736628	-1.392182
21	6	0	0.715094	-3.400300	1.259792
22	6	0	1.906283	-3.296802	-0.856814
23	6	0	1.840392	-1.312935	-2.291928
24	6	0	1.244181	-4.654592	1.519460
25	1	0	0.069258	-2.932713	1.990089
26	6	0	2.428573	-4.577993	-0.588477
27	6	0	2.097854	-5.246418	0.581634
28	1	0	1.000458	-5.166786	2.445023
29	1	0	3.105827	-5.046681	-1.290693
30	1	0	2.518190	-6.230541	0.768228
31	8	0	2.281863	-0.691417	-3.264380

32	7	0	2.280405	-2.605568	-2.004776
33	46	0	0.255300	-0.103736	1.411971
34	6	0	3.248659	-3.194955	-2.932292
35	1	0	4.199770	-3.383987	-2.424172
36	1	0	2.862746	-4.134288	-3.340862
37	1	0	3.401571	-2.477604	-3.735235
38	35	0	0.635784	1.432823	3.363145
39	6	0	0.221967	0.545957	-1.885141
40	6	0	-0.906329	0.454099	-2.715386
41	6	0	0.765667	1.817735	-1.604639
42	6	0	-1.542939	1.592700	-3.208617
43	6	0	0.117613	2.951292	-2.112925
44	6	0	-1.034753	2.851696	-2.891755
45	1	0	-2.417472	1.493237	-3.845681
46	1	0	0.538726	3.931465	-1.902121
47	1	0	-1.514071	3.750150	-3.270735
48	6	0	2.040792	2.001463	-0.799370
49	1	0	2.399451	3.032648	-0.945817
50	1	0	1.838505	1.889732	0.274476
51	1	0	-1.279630	-0.529820	-2.992234
52	8	0	3.059119	1.062825	-1.104879
53	1	0	3.037600	0.882567	-2.062171

J

Center Number	Atomic Number	Atomic Type	Coordinates (Angstroms) X	Y	Z
1	6	0	-4.642080	-1.881985	1.397242
2	6	0	-3.253064	-1.410098	1.953596
3	6	0	-3.751953	0.172223	0.402480
4	6	0	-4.985335	-0.791909	0.332419
5	1	0	-4.595732	-2.891163	0.976912
6	1	0	-5.378458	-1.896621	2.207626
7	1	0	-5.126949	-1.205789	-0.670548
8	1	0	-5.899063	-0.249068	0.596753
9	6	0	-3.452580	0.123821	1.919055
10	1	0	-2.566875	0.703815	2.224248
11	1	0	-4.292778	0.460102	2.535781
12	6	0	-2.288323	-1.612843	0.792587
13	1	0	-1.785855	-2.552794	0.589952

14	6	0	-2.589729	-0.641182	-0.153694
15	1	0	-2.369934	-0.721896	-1.210812
16	1	0	-2.964913	-1.859081	2.905614
17	1	0	-3.906325	1.153999	-0.047623
18	6	0	0.522238	-0.792580	-0.517050
19	6	0	1.088254	-2.103977	-0.309551
20	6	0	0.742018	-0.071122	-1.644571
21	6	0	0.997579	-2.816300	0.903829
22	6	0	1.796723	-2.692363	-1.396136
23	6	0	1.500980	-0.675367	-2.759555
24	6	0	1.548513	-4.080031	1.046172
25	1	0	0.507234	-2.335741	1.743989
26	6	0	2.340943	-3.983408	-1.241279
27	6	0	2.212885	-4.665037	-0.038908
28	1	0	1.473700	-4.603965	1.994074
29	1	0	2.876263	-4.449853	-2.058443
30	1	0	2.647840	-5.656001	0.056857
31	8	0	1.713404	-0.066700	-3.811619
32	7	0	1.948277	-1.988169	-2.585048
33	46	0	-0.674403	-0.041613	0.893189
34	6	0	2.681355	-2.588808	-3.700806
35	1	0	3.717727	-2.794832	-3.413699
36	1	0	2.200304	-3.520220	-4.015954
37	1	0	2.667013	-1.870413	-4.517267
38	35	0	1.167686	1.020724	2.219637
39	6	0	0.252113	1.324371	-1.889054
40	6	0	-1.030658	1.519652	-2.418589
41	6	0	1.077177	2.447680	-1.653559
42	6	0	-1.533302	2.798620	-2.659951
43	6	0	0.556380	3.723812	-1.900354
44	6	0	-0.739127	3.909597	-2.382913
45	1	0	-2.530622	2.921153	-3.074147
46	1	0	1.189896	4.588827	-1.718683
47	1	0	-1.113103	4.913576	-2.565019
48	6	0	2.509751	2.321340	-1.167397
49	1	0	2.971585	3.321204	-1.190616
50	1	0	2.528002	1.977948	-0.127763
51	1	0	-1.633621	0.652783	-2.671625
52	8	0	3.296623	1.392110	-1.903772
53	1	0	2.989025	1.388232	-2.826424

K

Center Number	Atomic Number	Atomic Type	Coordinates (Angstroms) X	Y	Z
1	6	0	-0.373471	-0.809389	-0.494223
2	6	0	-0.075318	-2.212304	-0.266110
3	6	0	-1.501809	-0.457104	-1.180218
4	6	0	1.087227	-2.649576	0.407701
5	6	0	-1.004588	-3.196139	-0.714061
6	6	0	-2.428273	-1.460256	-1.725412
7	6	0	1.314534	-3.992101	0.683529
8	1	0	1.844675	-1.904759	0.636427
9	6	0	-0.765201	-4.556718	-0.423130
10	6	0	0.371391	-4.944415	0.273745
11	1	0	2.231529	-4.301836	1.177335
12	1	0	-1.467447	-5.313967	-0.748735
13	1	0	0.534154	-5.999218	0.478261
14	8	0	-3.430263	-1.171084	-2.378394
15	7	0	-2.127630	-2.807047	-1.431093
16	46	0	0.600344	0.674776	0.398213
17	6	0	-3.058122	-3.813572	-1.940667
18	1	0	-2.542076	-4.513057	-2.606545
19	1	0	-3.515895	-4.373432	-1.117384
20	1	0	-3.829668	-3.281628	-2.493634
21	35	0	2.686334	0.288776	-0.930080
22	6	0	-1.875051	0.972669	-1.421533
23	6	0	-1.820893	1.491181	-2.721582
24	6	0	-2.287007	1.820577	-0.368799
25	6	0	-2.133908	2.825080	-2.977507
26	6	0	-2.602409	3.158910	-0.638834
27	6	0	-2.522670	3.666623	-1.933701
28	1	0	-2.074893	3.206550	-3.993109
29	1	0	-2.921217	3.804839	0.176821
30	1	0	-2.767268	4.707143	-2.127385
31	6	0	-2.426635	1.302166	1.037280
32	1	0	-3.037292	1.977232	1.645800
33	1	0	-2.884074	0.310737	1.050958
34	1	0	-1.525520	0.836510	-3.535062
35	8	0	-1.151335	1.112016	1.732081
36	1	0	-0.688333	1.974176	1.881541
37	6	0	1.821279	2.245410	2.741174
38	8	0	1.174686	2.424559	1.635647
39	8	0	1.767210	1.215740	3.447027

40	6	0	2.706346	3.408548	3.166636
41	1	0	2.177886	4.358553	3.042180
42	1	0	3.040425	3.289747	4.199718
43	1	0	3.581511	3.439120	2.506815
44	19	0	0.419126	-0.917035	3.138017

TS_{KL}

Center Number	Atomic Number	Atomic Type	Coordinates (Angstroms) X	Y	Z
1	6	0	1.924317	-0.500265	-1.288308
2	6	0	2.251275	-1.883790	-1.020186
3	6	0	0.782181	-0.151869	-1.943643
4	6	0	3.429720	-2.289063	-0.355507
5	6	0	1.321747	-2.883861	-1.430482
6	6	0	-0.141835	-1.181797	-2.450151
7	6	0	3.675372	-3.622654	-0.054562
8	1	0	4.176939	-1.528338	-0.145603
9	6	0	1.583993	-4.234823	-1.116420
10	6	0	2.737142	-4.593752	-0.431127
11	1	0	4.601840	-3.911600	0.433635
12	1	0	0.886118	-5.007142	-1.414546
13	1	0	2.916257	-5.642019	-0.208151
14	8	0	-1.156131	-0.919276	-3.091701
15	7	0	0.181747	-2.519093	-2.132443
16	46	0	2.966333	0.985429	-0.526911
17	6	0	-0.748146	-3.546586	-2.601723
18	1	0	-0.238449	-4.252144	-3.265912
19	1	0	-1.179737	-4.094996	-1.757135
20	1	0	-1.538732	-3.035382	-3.147103
21	35	0	4.993647	0.655636	-1.938229
22	6	0	0.399520	1.272977	-2.186920
23	6	0	0.342719	1.756860	-3.500237
24	6	0	0.106479	2.149126	-1.117727
25	6	0	0.033675	3.091301	-3.759241
26	6	0	-0.202490	3.486548	-1.393455
27	6	0	-0.234734	3.963043	-2.703266
28	1	0	0.004508	3.448327	-4.784970
29	1	0	-0.423999	4.158830	-0.567563
30	1	0	-0.471940	5.005378	-2.897352

31	6	0	0.063156	1.658267	0.311932
32	1	0	-0.394972	2.420225	0.953512
33	1	0	-0.550556	0.752229	0.376851
34	1	0	0.546483	1.076957	-4.320971
35	8	0	1.346962	1.316449	0.877797
36	1	0	1.992985	2.175098	1.206621
37	6	0	3.841498	2.615214	2.342127
38	8	0	3.080217	2.923638	1.331031
39	8	0	3.658077	1.634733	3.087707
40	6	0	5.019411	3.548621	2.557991
41	1	0	4.664830	4.580391	2.650561
42	1	0	5.580688	3.263978	3.449931
43	1	0	5.675363	3.512972	1.680947
44	19	0	2.549643	-0.577110	2.383786

L

Center Number	Atomic Number	Atomic Type	Coordinates (Angstroms)		
			X	Y	Z
1	6	0	-0.324643	-0.876786	-0.909562
2	6	0	-0.021769	-2.252947	-0.574364
3	6	0	-1.548699	-0.510767	-1.382509
4	6	0	1.243848	-2.680874	-0.114961
5	6	0	-1.060860	-3.221193	-0.703787
6	6	0	-2.600111	-1.521929	-1.608785
7	6	0	1.477453	-3.997660	0.260950
8	1	0	2.052988	-1.955343	-0.121808
9	6	0	-0.810205	-4.554753	-0.314358
10	6	0	0.435952	-4.931592	0.169045
11	1	0	2.466689	-4.304969	0.587906
12	1	0	-1.590909	-5.300376	-0.397382
13	1	0	0.602877	-5.966505	0.454871
14	8	0	-3.700376	-1.254741	-2.084114
15	7	0	-2.293718	-2.842868	-1.213649
16	46	0	0.978722	0.569308	-0.584725
17	6	0	-3.347224	-3.841825	-1.396141
18	1	0	-3.011290	-4.636950	-2.069756
19	1	0	-3.635214	-4.282165	-0.435117
20	1	0	-4.200875	-3.328012	-1.833042
21	35	0	2.687518	-0.031600	-2.302481

22	6	0	-1.905114	0.904780	-1.694744
23	6	0	-2.268714	1.258005	-3.001086
24	6	0	-1.875005	1.898328	-0.692501
25	6	0	-2.566263	2.580339	-3.326364
26	6	0	-2.179621	3.220876	-1.034210
27	6	0	-2.518063	3.568222	-2.341739
28	1	0	-2.835682	2.836783	-4.347263
29	1	0	-2.156437	3.984305	-0.259564
30	1	0	-2.748385	4.601208	-2.588371
31	6	0	-1.560542	1.544766	0.745764
32	1	0	-1.835302	2.394064	1.388638
33	1	0	-2.199465	0.696949	1.047648
34	1	0	-2.314507	0.487582	-3.763445
35	8	0	-0.195801	1.221611	1.004332
36	1	0	0.485714	2.254932	1.870971
37	6	0	1.451272	2.409189	3.569464
38	8	0	0.938124	2.960463	2.489062
39	8	0	1.403600	1.203658	3.826581
40	6	0	2.131410	3.408904	4.476953
41	1	0	1.456381	4.242898	4.692417
42	1	0	2.443845	2.925359	5.403393
43	1	0	3.006942	3.825801	3.966774
44	19	0	0.594235	-0.852766	2.451780

M

Center Number	Atomic Number	Atomic Type	Coordinates (Angstroms) X	Y	Z
1	6	0	-0.433591	-1.237413	-0.651856
2	6	0	-0.248190	-2.671358	-0.565852
3	6	0	-1.557861	-0.679833	-1.180368
4	6	0	0.903357	-3.280510	-0.022907
5	6	0	-1.286585	-3.508027	-1.065670
6	6	0	-2.620410	-1.548417	-1.738651
7	6	0	1.033202	-4.659030	0.049006
8	1	0	1.714363	-2.643614	0.317748
9	6	0	-1.145134	-4.908244	-0.979784
10	6	0	-0.002376	-5.471431	-0.428129
11	1	0	1.935175	-5.100865	0.461800
12	1	0	-1.928138	-5.558346	-1.348903

13	1	0	0.083815	-6.553393	-0.377657
14	8	0	-3.639752	-1.104555	-2.262939
15	7	0	-2.419387	-2.936186	-1.631120
16	46	0	1.105245	-0.119384	-0.119465
17	6	0	-3.479416	-3.794041	-2.161856
18	1	0	-3.091752	-4.433464	-2.961864
19	1	0	-3.899180	-4.423342	-1.369872
20	1	0	-4.252732	-3.139832	-2.558282
21	35	0	3.736816	-0.272988	-0.043992
22	6	0	-1.808321	0.787751	-1.238505
23	6	0	-2.139855	1.394222	-2.460579
24	6	0	-1.731756	1.583440	-0.076553
25	6	0	-2.365254	2.766328	-2.539500
26	6	0	-1.969044	2.960442	-0.170184
27	6	0	-2.280978	3.555667	-1.390624
28	1	0	-2.612064	3.216984	-3.497036
29	1	0	-1.897363	3.569191	0.727817
30	1	0	-2.459712	4.626224	-1.445164
31	6	0	-1.363063	0.973377	1.254494
32	1	0	-1.679343	1.648429	2.063718
33	1	0	-1.879703	0.015885	1.399382
34	1	0	-2.227594	0.779618	-3.349217
35	8	0	0.045570	0.783010	1.400374
36	1	0	0.578777	2.277889	1.695046
37	6	0	1.516782	3.336513	3.038087
38	8	0	0.784295	3.249085	1.940561
39	8	0	1.930913	2.365990	3.670277
40	6	0	1.807664	4.764605	3.433677
41	1	0	0.875020	5.332726	3.507137
42	1	0	2.340488	4.788692	4.384883
43	1	0	2.416306	5.240633	2.657082
44	19	0	2.163538	-0.120719	2.946168

N

Center Number	Atomic Number	Atomic Type	Coordinates (Angstroms) X	Y	Z
1	6	0	-0.458709	-1.287179	-0.608430
2	6	0	-0.291300	-2.725737	-0.554448
3	6	0	-1.573444	-0.710705	-1.138618

4	6	0	0.833540	-3.355624	0.020933
5	6	0	-1.310860	-3.543593	-1.118335
6	6	0	-2.633875	-1.557220	-1.733814
7	6	0	0.962851	-4.735811	0.048724
8	1	0	1.619108	-2.732825	0.441441
9	6	0	-1.170989	-4.945950	-1.079285
10	6	0	-0.049549	-5.529135	-0.504455
11	1	0	1.842416	-5.193482	0.491378
12	1	0	-1.940022	-5.582135	-1.498849
13	1	0	0.035343	-6.612239	-0.489013
14	8	0	-3.652053	-1.096083	-2.245783
15	7	0	-2.427219	-2.948636	-1.691701
16	46	0	1.135299	-0.241501	-0.071099
17	6	0	-3.471198	-3.784228	-2.285681
18	1	0	-3.058009	-4.395707	-3.094608
19	1	0	-3.917847	-4.440479	-1.530957
20	1	0	-4.230856	-3.114488	-2.682553
21	35	0	3.753515	0.054253	0.218652
22	6	0	-1.803971	0.759806	-1.167807
23	6	0	-2.161779	1.390225	-2.371003
24	6	0	-1.676901	1.539042	0.001674
25	6	0	-2.368565	2.766061	-2.424812
26	6	0	-1.900156	2.920288	-0.066606
27	6	0	-2.240301	3.537527	-1.268062
28	1	0	-2.635704	3.233506	-3.368776
29	1	0	-1.792248	3.516026	0.836133
30	1	0	-2.405956	4.611025	-1.301794
31	6	0	-1.277334	0.912052	1.316594
32	1	0	-1.576492	1.578785	2.139455
33	1	0	-1.789245	-0.047369	1.462026
34	1	0	-2.285316	0.790385	-3.265080
35	8	0	0.135574	0.725441	1.440528
36	1	0	0.667358	2.269073	1.639002
37	6	0	1.492415	3.429301	2.969612
38	8	0	0.861231	3.252042	1.820073
39	8	0	1.827933	2.514885	3.719918
40	6	0	1.774124	4.883901	3.260262
41	1	0	0.856813	5.473200	3.166511
42	1	0	2.193046	4.990986	4.261406
43	1	0	2.484375	5.269669	2.520564
44	19	0	2.177735	0.014358	3.097408

O

Center Number	Atomic Number	Atomic Type	Coordinates (Angstroms) X	Y	Z
1	6	0	-0.244378	-0.211228	0.521461
2	6	0	0.124922	-1.579419	0.816035
3	6	0	-1.507128	0.116712	0.112958
4	6	0	1.399318	-1.944453	1.297690
5	6	0	-0.844758	-2.603189	0.620482
6	6	0	-2.513744	-0.942594	-0.116530
7	6	0	1.726247	-3.264548	1.568573
8	1	0	2.120760	-1.151092	1.468616
9	6	0	-0.500772	-3.942910	0.894467
10	6	0	0.767936	-4.264218	1.359890
11	1	0	2.712630	-3.517294	1.946120
12	1	0	-1.226268	-4.733587	0.749406
13	1	0	1.008022	-5.303496	1.568265
14	8	0	-3.649769	-0.719175	-0.533583
15	7	0	-2.111665	-2.265109	0.161391
16	46	0	1.140469	1.208090	0.658506
17	6	0	-3.117222	-3.309284	-0.029611
18	1	0	-2.785199	-4.035067	-0.780287
19	1	0	-3.311438	-3.833396	0.912265
20	1	0	-4.026086	-2.818737	-0.371347
21	6	0	-1.949733	1.520882	-0.130024
22	6	0	-2.556889	1.861411	-1.349732
23	6	0	-1.765356	2.524201	0.848401
24	6	0	-2.945152	3.172073	-1.620128
25	6	0	-2.168255	3.833983	0.562076
26	6	0	-2.749707	4.166732	-0.660580
27	1	0	-3.406863	3.412494	-2.574504
28	1	0	-2.024920	4.601153	1.319955
29	1	0	-3.055299	5.190925	-0.859428
30	6	0	-1.129432	2.212293	2.191049
31	1	0	-1.361940	3.032359	2.888151
32	1	0	-1.584436	1.298077	2.608098
33	1	0	-2.735685	1.084569	-2.085078
34	8	0	0.273307	2.099957	2.155624
35	6	0	1.954731	-0.061757	-1.968742
36	8	0	2.389077	0.493307	-0.942136
37	1	0	0.891305	-0.302334	-2.080224
38	7	0	2.724011	-0.415510	-3.008199

39	6	0	2.156918	-1.066827	-4.180863
40	1	0	2.337104	-0.462694	-5.077744
41	1	0	2.610841	-2.054138	-4.326261
42	1	0	1.079314	-1.190957	-4.051594
43	6	0	4.161273	-0.165038	-3.005211
44	1	0	4.431552	0.317586	-2.066820
45	1	0	4.706466	-1.110873	-3.102625
46	1	0	4.430538	0.486167	-3.844712

TS$_{OP}$

Center Number	Atomic Number	Atomic Type	Coordinates (Angstroms) X	Y	Z
1	6	0	-0.510003	-0.944760	-0.720225
2	6	0	-0.265291	-2.274972	-0.208902
3	6	0	-1.635788	-0.647963	-1.458699
4	6	0	0.817880	-2.583265	0.634889
5	6	0	-1.168255	-3.305972	-0.584926
6	6	0	-2.532994	-1.715951	-1.895515
7	6	0	1.024327	-3.872594	1.101221
8	1	0	1.484838	-1.775711	0.919537
9	6	0	-0.940873	-4.615203	-0.111145
10	6	0	0.138582	-4.888370	0.719280
11	1	0	1.862150	-4.089920	1.756943
12	1	0	-1.612051	-5.418797	-0.387591
13	1	0	0.288179	-5.904593	1.074496
14	8	0	-3.494290	-1.538873	-2.649485
15	7	0	-2.244597	-3.015796	-1.416548
16	46	0	1.069206	0.289731	-0.902457
17	6	0	-3.158670	-4.081189	-1.818874
18	1	0	-2.626633	-4.862824	-2.373296
19	1	0	-3.639679	-4.531742	-0.943481
20	1	0	-3.913891	-3.628137	-2.457975
21	6	0	-1.942575	0.766164	-1.779697
22	6	0	-2.445175	1.188979	-3.019582
23	6	0	-1.696238	1.730213	-0.774923
24	6	0	-2.667075	2.544122	-3.270351
25	6	0	-1.914851	3.083070	-1.043095
26	6	0	-2.394004	3.497194	-2.287908
27	1	0	-3.055856	2.854347	-4.237223

28	1	0	-1.722661	3.816001	-0.261998
29	1	0	-2.562028	4.553056	-2.483756
30	6	0	-1.309449	1.269002	0.617287
31	1	0	-1.073105	2.142929	1.239960
32	1	0	-2.177302	0.759631	1.071487
33	1	0	-2.681100	0.446349	-3.772276
34	8	0	-0.214825	0.364102	0.690157
35	6	0	1.649213	-0.465215	-3.714841
36	8	0	2.212199	-0.038339	-2.689384
37	1	0	0.605970	-0.804663	-3.692246
38	7	0	2.244601	-0.555225	-4.913292
39	6	0	1.531748	-1.069963	-6.074211
40	1	0	1.488987	-0.310238	-6.863474
41	1	0	2.037269	-1.958415	-6.470864
42	1	0	0.511625	-1.341510	-5.792925
43	6	0	3.628587	-0.134676	-5.101517
44	1	0	4.013271	0.237549	-4.152716
45	1	0	4.236949	-0.982191	-5.438030
46	1	0	3.679810	0.658279	-5.856476

P

Center Number	Atomic Number	Atomic Type	Coordinates (Angstroms) X	Y	Z
1	6	0	-0.412937	-0.187031	1.330204
2	6	0	-0.171385	-1.523245	1.866773
3	6	0	-1.390169	0.032711	0.321456
4	6	0	0.739299	-1.743901	2.912477
5	6	0	-0.897697	-2.613825	1.324219
6	6	0	-2.130702	-1.124640	-0.230903
7	6	0	0.966404	-3.022913	3.404414
8	1	0	1.260861	-0.887123	3.324407
9	6	0	-0.653271	-3.904396	1.829055
10	6	0	0.269679	-4.102623	2.852487
11	1	0	1.677055	-3.180116	4.210642
12	1	0	-1.186914	-4.757531	1.429219
13	1	0	0.437610	-5.109665	3.224938
14	8	0	-2.992327	-1.018161	-1.106302
15	7	0	-1.843767	-2.391410	0.321133
16	46	0	0.588525	-0.149117	-0.567248

17	6	0	-2.581408	-3.525211	-0.228104
18	1	0	-1.897645	-4.241656	-0.698634
19	1	0	-3.146301	-4.036721	0.558993
20	1	0	-3.268704	-3.132294	-0.974010
21	6	0	-1.879259	1.420866	0.107068
22	6	0	-2.566600	1.850726	-1.040355
23	6	0	-1.612229	2.358950	1.125538
24	6	0	-2.970950	3.180032	-1.163268
25	6	0	-2.006256	3.688516	0.986194
26	6	0	-2.690664	4.106391	-0.156952
27	1	0	-3.506645	3.493411	-2.055917
28	1	0	-1.783964	4.399105	1.779993
29	1	0	-2.999778	5.143170	-0.259655
30	6	0	-0.945613	1.860808	2.382225
31	1	0	-0.416987	2.662501	2.903965
32	1	0	-1.692583	1.437397	3.075575
33	1	0	-2.790327	1.131056	-1.816216
34	8	0	0.043376	0.858310	2.116277
35	6	0	1.795250	-0.126479	-3.430388
36	8	0	2.041916	-0.139065	-2.217793
37	1	0	0.764996	-0.124236	-3.812535
38	7	0	2.737922	-0.114043	-4.394285
39	6	0	2.381840	-0.098282	-5.803922
40	1	0	2.780979	0.798879	-6.292832
41	1	0	2.786295	-0.981147	-6.314017
42	1	0	1.294248	-0.100224	-5.910859
43	6	0	4.154899	-0.113249	-4.053367
44	1	0	4.250017	-0.123825	-2.968018
45	1	0	4.646179	-0.998675	-4.474168
46	1	0	4.640598	0.783496	-4.456272

DMF

Center Number	Atomic Number	Atomic Type	Coordinates (Angstroms) X	Y	Z
1	6	0	0.423519	0.260808	0.058164
2	8	0	1.135727	0.512259	-0.899725
3	1	0	0.696420	0.545044	1.093744
4	7	0	-0.778513	-0.386477	0.009962
5	6	0	-1.559488	-0.642147	1.202983

6	1	0	-2.544277	-0.159304	1.142256
7	1	0	-1.715764	-1.719452	1.349793
8	1	0	-1.034516	-0.246929	2.077486
9	6	0	-1.309842	-0.841423	-1.263143
10	1	0	-0.596528	-0.568610	-2.041588
11	1	0	-1.449612	-1.930347	-1.258391
12	1	0	-2.278599	-0.368529	-1.471067

NBE

Center Number	Atomic Number	Atomic Type	Coordinates (Angstroms) X	Y	Z
1	6	0	-0.108665	-0.608299	-0.009282
2	6	0	1.459680	-0.582058	-0.008525
3	6	0	0.922882	1.610256	-0.008753
4	6	0	-0.480307	0.908422	-0.008581
5	1	0	-0.506924	-1.144028	-0.876096
6	1	0	-0.483475	-1.109290	0.890770
7	1	0	-1.082130	1.199847	-0.874602
8	1	0	-1.043479	1.178796	0.892176
9	6	0	1.716815	0.642824	0.901884
10	1	0	2.777307	0.902467	0.978153
11	1	0	1.288711	0.538293	1.906943
12	6	0	1.876843	-0.008194	-1.355811
13	6	0	1.557566	1.293665	-1.356022
14	1	0	1.925586	-1.527502	0.280588
15	1	0	0.899002	2.664058	0.280114
16	1	0	1.615483	1.985597	-2.190901
17	1	0	2.247790	-0.595257	-2.190620

KOAc

Center Number	Atomic Number	Atomic Type	Coordinates (Angstroms) X	Y	Z
1	6	0	-0.573070	0.171577	-0.038078
2	8	0	0.693812	0.197059	0.021994
3	8	0	-1.330597	1.188372	-0.068806
4	19	0	0.682624	2.744457	-0.003402

5	6	0	-1.245900	-1.208312	-0.035547
6	1	0	-0.616083	-1.946302	-0.540701
7	1	0	-1.377745	-1.540082	1.002545
8	1	0	-2.233123	-1.162323	-0.503252

3q

Center Number	Atomic Number	Atomic Type	Coordinates (Angstroms)		
			X	Y	Z
1	6	0	-0.231786	-0.519625	0.004996
2	6	0	1.161762	-0.533164	-0.045128
3	6	0	1.864056	0.671138	-0.051321
4	6	0	1.208802	1.910217	-0.009332
5	6	0	-0.189146	1.882902	0.044452
6	6	0	-0.916465	0.696591	0.049127
7	1	0	-0.793343	-1.449686	0.008695
8	1	0	1.700274	-1.475834	-0.078629
9	1	0	2.950643	0.664078	-0.086054
10	1	0	-1.999739	0.721870	0.086107
11	6	0	2.017122	3.184877	0.009197
12	1	0	3.016311	2.977338	-0.404242
13	1	0	1.537862	3.934283	-0.634386
14	8	0	2.109424	3.646294	1.354744
15	1	0	2.334608	4.588636	1.328372
16	35	0	-1.225558	3.568412	0.099203

AcOH•KI

Center Number	Atomic Number	Atomic Type	Coordinates (Angstroms)		
			X	Y	Z
1	1	0	1.007606	0.779792	2.751118
2	19	0	3.212754	2.340122	1.157544
3	8	0	3.823832	-0.167983	2.406567
4	6	0	2.637552	-0.291999	2.654316
5	8	0	1.950279	0.803593	3.063099
6	53	0	-0.036689	1.584078	0.439471
7	6	0	1.848524	-1.567335	2.523835
8	1	0	1.368230	-1.815023	3.476506

9	1	0	1.055439	-1.421540	1.779019
10	1	0	2.505468	-2.380076	2.212586

AcOH•KBr

Center Number	Atomic Number	Atomic Type	Coordinates (Angstroms)		
			X	Y	Z
1	35	0	2.104766	0.241147	0.461687
2	1	0	1.738899	2.343844	1.218171
3	6	0	1.785427	3.416073	2.835033
4	8	0	1.573706	3.286519	1.538571
5	8	0	2.140533	2.505636	3.582594
6	6	0	1.546274	4.828576	3.316577
7	1	0	0.530137	5.143748	3.058539
8	1	0	1.694306	4.886278	4.395647
9	1	0	2.234895	5.512648	2.809247
10	19	0	2.739603	-0.012800	3.457343

a

Center Number	Atomic Number	Atomic Type	Coordinates (Angstroms)		
			X	Y	Z
1	6	0	-2.378850	-2.832599	2.587033
2	6	0	-0.966489	-2.277689	2.260422
3	6	0	-2.269649	-0.419782	2.210896
4	6	0	-3.291325	-1.561253	2.496456
5	1	0	-2.684345	-3.628350	1.896547
6	1	0	-2.393365	-3.256825	3.598821
7	1	0	-4.042814	-1.640898	1.702473
8	1	0	-3.830070	-1.393394	3.439036
9	6	0	-1.048727	-0.920550	3.007959
10	1	0	-0.183560	-0.265561	2.899188
11	1	0	-1.252969	-1.061857	4.078189
12	6	0	-1.011289	-1.904191	0.750102
13	1	0	-1.506742	-2.723109	0.214828
14	6	0	-1.779214	-0.555284	0.764980
15	1	0	-2.572247	-0.467726	0.014744
16	1	0	-0.148932	-2.949906	2.544475

17	1	0	-2.632347	0.579699	2.465770
18	6	0	0.287789	-1.533266	0.026496
19	6	0	0.476763	-1.816940	-1.401684
20	6	0	1.342162	-0.940131	0.716670
21	6	0	-0.514769	-2.420234	-2.195441
22	6	0	1.709708	-1.455444	-2.020913
23	6	0	2.644262	-0.659456	0.109259
24	1	0	1.363271	-0.901091	1.796800
25	6	0	-0.323741	-2.673027	-3.548681
26	1	0	-1.467548	-2.671267	-1.744443
27	6	0	1.888690	-1.711679	-3.393805
28	6	0	0.885742	-2.312866	-4.146199
29	1	0	-1.114944	-3.134335	-4.133289
30	1	0	2.815986	-1.433409	-3.878463
31	1	0	1.052211	-2.494781	-5.205160
32	8	0	3.617355	-0.290756	0.762940
33	7	0	2.732900	-0.871486	-1.276475
34	46	0	-0.192053	0.686292	0.149998
35	8	0	1.034124	2.244115	-0.605998
36	6	0	-0.002039	3.065020	-0.622202
37	8	0	-1.119815	2.461696	-0.160624
38	8	0	0.009214	4.231910	-0.998909
39	6	0	3.964359	-0.441709	-1.927745
40	1	0	4.498030	-1.292222	-2.370313
41	1	0	3.742665	0.294731	-2.706948
42	1	0	4.584510	0.012920	-1.157555

TS_{ab}

Center Number	Atomic Number	Atomic Type	Coordinates (Angstroms) X	Y	Z
1	6	0	0.618578	-0.076022	0.143725
2	6	0	2.081934	0.366322	-0.104615
3	6	0	1.055307	2.313434	0.444827
4	6	0	-0.096845	1.267712	0.507758
5	1	0	0.174664	-0.570173	-0.730453
6	1	0	0.568839	-0.787915	0.977167
7	1	0	-0.919548	1.507750	-0.177350
8	1	0	-0.519465	1.229056	1.519826
9	6	0	2.248579	1.476009	0.954574

10	1	0	3.198344	2.013397	0.906819
11	1	0	2.097341	1.102519	1.976244
12	6	0	2.111867	1.175177	-1.442383
13	1	0	1.477084	0.668564	-2.182981
14	6	0	1.406663	2.509449	-1.038257
15	1	0	0.522666	2.726731	-1.660164
16	1	0	2.808348	-0.451800	-0.072388
17	1	0	0.853919	3.245193	0.973867
18	6	0	3.480227	1.364023	-2.082282
19	6	0	4.012547	0.308572	-2.935277
20	6	0	4.200243	2.519521	-1.854511
21	6	0	3.366456	-0.930029	-3.132269
22	6	0	5.278743	0.502098	-3.565209
23	6	0	5.388203	2.792813	-2.671447
24	1	0	4.293974	3.188387	-0.684247
25	6	0	3.922531	-1.943642	-3.903597
26	1	0	2.408869	-1.102230	-2.653269
27	6	0	5.832828	-0.530289	-4.347763
28	6	0	5.163090	-1.738187	-4.513897
29	1	0	3.395231	-2.886197	-4.026718
30	1	0	6.792525	-0.388255	-4.830074
31	1	0	5.614014	-2.518817	-5.122205
32	8	0	5.920587	3.900821	-2.737288
33	7	0	5.938542	1.713916	-3.413157
34	46	0	2.524164	4.103033	-1.543304
35	8	0	4.574433	3.713322	0.489745
36	6	0	3.817868	4.679720	0.965819
37	8	0	2.698278	5.034945	0.309430
38	8	0	4.084648	5.278328	2.013049
39	6	0	7.200231	1.954625	-4.100442
40	1	0	7.961076	1.230928	-3.784082
41	1	0	7.079856	1.887096	-5.189248
42	1	0	7.511139	2.963995	-3.835166

b

Center Number	Atomic Number	Atomic Type	Coordinates (Angstroms)		
			X	Y	Z
1	6	0	-2.762462	-2.322163	1.587289
2	6	0	-1.286289	-2.186339	1.144436

3	6	0	-1.691245	-0.199364	2.161464
4	6	0	-3.051496	-0.946356	2.273100
5	1	0	-3.431744	-2.524344	0.741558
6	1	0	-2.878413	-3.150669	2.297705
7	1	0	-3.863378	-0.392322	1.788393
8	1	0	-3.332665	-1.078880	3.325605
9	6	0	-0.694909	-1.367968	2.311317
10	1	0	0.343291	-1.065612	2.146685
11	1	0	-0.773479	-1.887527	3.275456
12	6	0	-1.234146	-1.167499	-0.041976
13	1	0	-2.048817	-1.394965	-0.748633
14	6	0	-1.495595	0.203155	0.686594
15	1	0	-2.360938	0.741075	0.283099
16	1	0	-0.796436	-3.141001	0.930102
17	1	0	-1.586342	0.624622	2.869082
18	6	0	0.076611	-1.108181	-0.780497
19	6	0	0.571167	-2.181469	-1.622116
20	6	0	0.780601	0.056374	-0.631250
21	6	0	-0.149884	-3.366448	-1.870671
22	6	0	1.842339	-2.024586	-2.249549
23	6	0	2.080772	0.229067	-1.270466
24	1	0	0.306739	1.909822	2.829590
25	6	0	0.347515	-4.373551	-2.689361
26	1	0	-1.124708	-3.485300	-1.408788
27	6	0	2.340450	-3.054774	-3.070947
28	6	0	1.602105	-4.214738	-3.286286
29	1	0	-0.235306	-5.274883	-2.861757
30	1	0	3.306783	-2.948678	-3.549722
31	1	0	2.008865	-4.993726	-3.926909
32	8	0	2.759933	1.256729	-1.157530
33	7	0	2.567373	-0.853231	-2.045996
34	46	0	0.009956	1.554294	0.411272
35	8	0	-0.056751	2.564133	3.455150
36	6	0	-0.719579	3.518960	2.669467
37	8	0	-0.741473	3.267849	1.400528
38	8	0	-1.206497	4.476629	3.251902
39	6	0	3.873824	-0.686214	-2.669555
40	1	0	4.568533	-1.468024	-2.339399
41	1	0	3.797128	-0.721182	-3.763440
42	1	0	4.242968	0.290711	-2.360871

----

sG

Center Number	Atomic Number	Atomic Type	Coordinates (Angstroms)		
			X	Y	Z
1	6	0	-3.141387	-2.696816	2.871035
2	6	0	-1.625624	-2.642450	2.568468
3	6	0	-2.251029	-0.474001	2.384498
4	6	0	-3.584284	-1.204893	2.715897
5	1	0	-3.677598	-3.371340	2.193026
6	1	0	-3.316260	-3.055369	3.892178
7	1	0	-4.334545	-1.065785	1.929283
8	1	0	-4.015838	-0.820087	3.648007
9	6	0	-1.238246	-1.293755	3.210294
10	1	0	-0.201004	-1.000298	3.024186
11	1	0	-1.433628	-1.270886	4.289661
12	6	0	-1.462033	-2.352510	1.037728
13	1	0	-2.172982	-2.979098	0.477929
14	6	0	-1.847429	-0.833335	0.938742
15	1	0	-2.660190	-0.658627	0.221460
16	1	0	-1.069615	-3.517167	2.917293
17	1	0	-2.288644	0.600119	2.591564
18	6	0	-0.081730	-2.557117	0.486212
19	6	0	0.510461	-3.867525	0.313444
20	6	0	0.596200	-1.422706	0.117313
21	6	0	-0.165521	-5.065070	0.630419
22	6	0	1.825876	-3.956286	-0.215834
23	6	0	1.939919	-1.517062	-0.432969
24	6	0	0.426463	-6.305239	0.447449
25	1	0	-1.174430	-5.006161	1.023988
26	6	0	2.421602	-5.220592	-0.393609
27	6	0	1.728384	-6.377886	-0.063855
28	1	0	-0.116249	-7.211886	0.698831
29	1	0	3.424180	-5.303096	-0.793964
30	1	0	2.205456	-7.343065	-0.210294
31	8	0	2.617600	-0.524819	-0.798351
32	7	0	2.505920	-2.787109	-0.559082
33	46	0	-0.319612	0.343237	0.276798
34	6	0	3.866058	-2.875988	-1.093299
35	1	0	4.526576	-3.370873	-0.373937
36	1	0	3.874373	-3.437688	-2.033606
37	1	0	4.212161	-1.861329	-1.272026
38	35	0	-1.558979	2.832132	0.069891
39	6	0	-0.968458	2.942654	-1.836386

40	6	0	-2.025287	3.053280	-2.735119
41	6	0	0.380880	2.885844	-2.220391
42	6	0	-1.758852	3.120904	-4.101862
43	6	0	0.597344	2.954800	-3.609742
44	6	0	-0.437953	3.069413	-4.536207
45	1	0	-2.579000	3.207673	-4.808483
46	1	0	1.613062	2.914573	-3.982466
47	1	0	-0.204638	3.115044	-5.596110
48	6	0	1.584681	2.808441	-1.248628
49	1	0	-3.045505	3.082900	-2.369535
50	8	0	1.418885	1.735820	-0.301660
51	1	0	1.979020	0.940880	-0.554997
52	6	0	1.694906	4.119638	-0.447515
53	1	0	2.560229	4.060046	0.220307
54	1	0	1.827850	4.971769	-1.123504
55	1	0	0.807820	4.295140	0.162932
56	6	0	2.918859	2.567096	-1.975798
57	1	0	3.194379	3.407381	-2.620254
58	1	0	3.701006	2.457116	-1.218856
59	1	0	2.895785	1.648410	-2.569896

TS_{sGH}

Center Number	Atomic Number	Atomic Type	Coordinates (Angstroms)		
			X	Y	Z
1	6	0	-3.776913	-2.335546	1.057246
2	6	0	-2.247643	-2.398920	0.837601
3	6	0	-2.638596	-0.173205	0.999399
4	6	0	-4.054148	-0.800467	1.154957
5	1	0	-4.333886	-2.816689	0.244572
6	1	0	-4.052406	-2.846974	1.986647
7	1	0	-4.749721	-0.443397	0.387400
8	1	0	-4.482127	-0.535022	2.128916
9	6	0	-1.762301	-1.223978	1.711234
10	1	0	-0.689713	-1.025246	1.622714
11	1	0	-2.015301	-1.353089	2.770405
12	6	0	-1.953941	-1.880005	-0.613007
13	1	0	-2.665973	-2.338914	-1.314611
14	6	0	-2.216372	-0.340161	-0.474823
15	1	0	-2.983530	0.014639	-1.169584

16	1	0	-1.804608	-3.374129	1.055436
17	1	0	-2.577729	0.850951	1.372688
18	6	0	-0.551079	-2.124156	-1.083093
19	6	0	-0.054381	-3.452527	-1.381036
20	6	0	0.253524	-1.021598	-1.222782
21	6	0	-0.866400	-4.605843	-1.332973
22	6	0	1.305829	-3.600762	-1.761456
23	6	0	1.649081	-1.178394	-1.606971
24	6	0	-0.364100	-5.861958	-1.634276
25	1	0	-1.910207	-4.500192	-1.058755
26	6	0	1.810119	-4.882492	-2.058976
27	6	0	0.983165	-5.995896	-1.993374
28	1	0	-1.010790	-6.733392	-1.592168
29	1	0	2.845402	-5.011279	-2.348221
30	1	0	1.391017	-6.974868	-2.229377
31	8	0	2.451393	-0.222847	-1.729464
32	7	0	2.121031	-2.472162	-1.839227
33	46	0	-0.569203	0.808534	-0.926726
34	6	0	3.533845	-2.626919	-2.193614
35	1	0	4.034234	-3.286462	-1.477573
36	1	0	3.634741	-3.046077	-3.200403
37	1	0	3.987664	-1.639825	-2.163240
38	35	0	-1.808978	3.153142	-0.738631
39	6	0	-1.225970	2.142859	-2.714707
40	6	0	-2.391975	1.793697	-3.391492
41	6	0	-0.034694	2.508427	-3.361337
42	6	0	-2.378225	1.755293	-4.785727
43	6	0	-0.073756	2.468538	-4.765096
44	6	0	-1.217794	2.103990	-5.474867
45	1	0	-3.280476	1.471472	-5.320568
46	1	0	0.826462	2.717001	-5.317445
47	1	0	-1.197793	2.093353	-6.560686
48	6	0	1.252738	2.867500	-2.601033
49	1	0	-3.296613	1.564704	-2.841486
50	8	0	1.292977	2.098088	-1.374113
51	1	0	1.850903	1.277012	-1.515380
52	6	0	1.266127	4.353307	-2.207124
53	1	0	2.194444	4.579037	-1.671766
54	1	0	1.212114	4.984066	-3.101531
55	1	0	0.424765	4.595552	-1.556831
56	6	0	2.521537	2.544374	-3.408256
57	1	0	2.627026	3.202493	-4.276009
58	1	0	3.392421	2.704199	-2.764633

59	1	0	2.530052	1.503728	-3.744306

sH

Center Number	Atomic Number	Atomic Type	Coordinates (Angstroms)		
			X	Y	Z
1	6	0	-3.301856	-2.241053	2.458967
2	6	0	-1.791968	-2.382521	2.155988
3	6	0	-2.005634	-0.173855	2.609289
4	6	0	-3.454289	-0.720689	2.779649
5	1	0	-3.925419	-2.562929	1.616743
6	1	0	-3.577357	-2.858997	3.320705
7	1	0	-4.168553	-0.210488	2.125870
8	1	0	-3.786418	-0.561023	3.811688
9	6	0	-1.176043	-1.363153	3.133284
10	1	0	-0.094811	-1.238195	3.011883
11	1	0	-1.385153	-1.601102	4.182216
12	6	0	-1.515319	-1.707635	0.763074
13	1	0	-2.251868	-2.060199	0.027901
14	6	0	-1.720588	-0.193277	1.094686
15	1	0	-2.501148	0.300883	0.516760
16	1	0	-1.414171	-3.405016	2.223554
17	1	0	-1.855199	0.795859	3.081291
18	6	0	-0.119006	-1.973129	0.271651
19	6	0	0.311157	-3.300801	-0.121118
20	6	0	0.753687	-0.919835	0.234492
21	6	0	-0.570358	-4.399772	-0.210473
22	6	0	1.674031	-3.497753	-0.467814
23	6	0	2.155604	-1.122349	-0.100610
24	6	0	-0.132374	-5.650578	-0.614308
25	1	0	-1.616673	-4.256559	0.034875
26	6	0	2.112184	-4.775722	-0.868825
27	6	0	1.218119	-5.834924	-0.938044
28	1	0	-0.831164	-6.479019	-0.679305
29	1	0	3.148057	-4.941646	-1.135362
30	1	0	1.575424	-6.811173	-1.253242
31	8	0	3.014439	-0.213827	-0.100223
32	7	0	2.558800	-2.422660	-0.409023
33	46	0	-0.035301	0.922783	0.655886
34	6	0	3.978355	-2.637865	-0.705447

35	1	0	4.391127	-3.399910	-0.037599
36	1	0	4.109975	-2.957175	-1.744559
37	1	0	4.492420	-1.693355	-0.548379
38	35	0	-1.243091	3.082699	1.463570
39	6	0	-0.270834	1.103164	-1.340162
40	6	0	-1.283511	0.461781	-2.045150
41	6	0	0.626301	1.989507	-1.950976
42	6	0	-1.445097	0.730953	-3.407124
43	6	0	0.433104	2.239687	-3.320439
44	6	0	-0.586083	1.625087	-4.043778
45	1	0	-2.242219	0.239423	-3.958379
46	1	0	1.097159	2.936223	-3.825215
47	1	0	-0.705185	1.842231	-5.101566
48	6	0	1.742400	2.699170	-1.177372
49	1	0	-1.955449	-0.232488	-1.556764
50	8	0	1.842473	2.116510	0.148646
51	1	0	2.432046	1.312420	0.090107
52	6	0	1.404450	4.182381	-0.977878
53	1	0	2.206514	4.665346	-0.409918
54	1	0	1.306726	4.684087	-1.946717
55	1	0	0.469985	4.290350	-0.423270
56	6	0	3.106089	2.542178	-1.872116
57	1	0	3.112222	3.037458	-2.847819
58	1	0	3.880783	3.006795	-1.252962
59	1	0	3.357929	1.487266	-2.014964

TS_{sHI}

Center Number	Atomic Number	Atomic Type	Coordinates (Angstroms) X	Y	Z
1	6	0	-3.220868	-2.283420	2.439603
2	6	0	-1.703880	-2.260939	2.149696
3	6	0	-2.135140	-0.095193	2.633952
4	6	0	-3.533537	-0.780068	2.714559
5	1	0	-3.802967	-2.698670	1.608179
6	1	0	-3.427631	-2.901127	3.320930
7	1	0	-4.242499	-0.353385	1.996784
8	1	0	-3.960245	-0.635857	3.713525
9	6	0	-1.212924	-1.210300	3.160210
10	1	0	-0.148246	-0.970378	3.070706

11	1	0	-1.423150	-1.496600	4.197027
12	6	0	-1.514703	-1.530566	0.760599
13	1	0	-2.301174	-1.885985	0.078560
14	6	0	-1.739891	-0.030986	1.150256
15	1	0	-2.477156	0.483771	0.528815
16	1	0	-1.220411	-3.237888	2.199646
17	1	0	-2.093662	0.864103	3.142663
18	6	0	-0.160603	-1.796328	0.153844
19	6	0	0.235943	-3.148320	-0.175596
20	6	0	0.696348	-0.741355	-0.116186
21	6	0	-0.670103	-4.235590	-0.165843
22	6	0	1.569186	-3.396919	-0.595913
23	6	0	2.113046	-1.021427	-0.384906
24	6	0	-0.281949	-5.508874	-0.546803
25	1	0	-1.698210	-4.062971	0.128338
26	6	0	1.959347	-4.697289	-0.968662
27	6	0	1.042599	-5.738598	-0.942182
28	1	0	-1.001435	-6.321883	-0.539132
29	1	0	2.974189	-4.893857	-1.289698
30	1	0	1.359962	-6.734256	-1.238400
31	8	0	3.003122	-0.149761	-0.395396
32	7	0	2.477839	-2.343250	-0.616975
33	46	0	-0.004356	1.031337	0.791090
34	6	0	3.894097	-2.615157	-0.887317
35	1	0	4.269235	-3.368749	-0.189092
36	1	0	4.026400	-2.972447	-1.913767
37	1	0	4.439756	-1.685173	-0.754583
38	35	0	-0.725958	3.003736	2.266990
39	6	0	0.055068	0.651282	-1.310889
40	6	0	-0.961152	0.050712	-2.078649
41	6	0	0.830645	1.691903	-1.889530
42	6	0	-1.190000	0.413330	-3.399573
43	6	0	0.595929	2.010247	-3.238953
44	6	0	-0.387653	1.386290	-3.996697
45	1	0	-1.977730	-0.080529	-3.961751
46	1	0	1.182697	2.803233	-3.693157
47	1	0	-0.535405	1.666514	-5.035754
48	6	0	1.779502	2.611227	-1.099582
49	1	0	-1.562346	-0.740469	-1.651971
50	8	0	1.925974	2.117787	0.249003
51	1	0	2.505945	1.313818	0.170783
52	6	0	1.153487	4.009102	-0.964729
53	1	0	1.822461	4.645555	-0.377107

54	1	0	1.004435	4.464203	-1.948885
55	1	0	0.193672	3.957732	-0.444946
56	6	0	3.176180	2.710205	-1.738451
57	1	0	3.131343	3.183476	-2.724352
58	1	0	3.815526	3.326447	-1.097912
59	1	0	3.630478	1.722131	-1.846546

sI

Center Number	Atomic Number	Atomic Type	Coordinates (Angstroms)		
			X	Y	Z
1	6	0	-3.818014	-1.252172	0.895390
2	6	0	-2.502048	-2.076048	0.944300
3	6	0	-2.027694	-0.256836	2.218558
4	6	0	-3.495228	0.003132	1.763383
5	1	0	-4.110410	-1.000032	-0.130039
6	1	0	-4.635745	-1.836336	1.330959
7	1	0	-3.602980	0.943362	1.212054
8	1	0	-4.147906	0.065833	2.641414
9	6	0	-2.028547	-1.789103	2.390240
10	1	0	-1.037406	-2.178538	2.637057
11	1	0	-2.744410	-2.131581	3.145964
12	6	0	-1.478885	-1.299487	0.069870
13	1	0	-1.962954	-1.022969	-0.868983
14	6	0	-1.191322	-0.064488	0.960467
15	1	0	-1.281684	0.912407	0.479626
16	1	0	-2.670598	-3.112678	0.650593
17	1	0	-1.704503	0.333802	3.073568
18	6	0	-0.040910	-1.798854	-0.278523
19	6	0	0.518384	-3.131705	0.008964
20	6	0	0.712945	-0.909017	-1.101993
21	6	0	-0.138936	-4.112336	0.782692
22	6	0	1.787068	-3.489725	-0.531223
23	6	0	2.068307	-1.297671	-1.572696
24	6	0	0.391496	-5.375062	0.999689
25	1	0	-1.080775	-3.878694	1.244297
26	6	0	2.315901	-4.775391	-0.313457
27	6	0	1.625558	-5.711581	0.441821
28	1	0	-0.155347	-6.090247	1.606383
29	1	0	3.275786	-5.044955	-0.732523

30	1	0	2.055656	-6.696464	0.598521
31	8	0	2.776200	-0.514183	-2.225747
32	7	0	2.528844	-2.563716	-1.270750
33	46	0	0.853208	-0.221074	1.061749
34	6	0	3.864255	-2.920494	-1.762617
35	1	0	4.513889	-3.192650	-0.925542
36	1	0	3.802554	-3.758636	-2.463758
37	1	0	4.265459	-2.048529	-2.272066
38	35	0	1.225306	0.631921	3.406159
39	6	0	0.077937	0.158982	-1.973846
40	6	0	-0.852692	-0.358732	-2.899795
41	6	0	0.357531	1.548532	-1.997917
42	6	0	-1.565214	0.445322	-3.780222
43	6	0	-0.403289	2.343601	-2.877814
44	6	0	-1.353524	1.822198	-3.748365
45	1	0	-2.272583	0.002066	-4.475380
46	1	0	-0.233583	3.415434	-2.889330
47	1	0	-1.906793	2.485473	-4.407603
48	6	0	1.447398	2.303429	-1.188955
49	1	0	-1.011955	-1.434343	-2.920342
50	8	0	2.285976	1.436030	-0.421123
51	1	0	2.746587	0.863178	-1.070566
52	6	0	0.819828	3.272754	-0.175992
53	1	0	1.615439	3.816433	0.342697
54	1	0	0.162567	3.995440	-0.669032
55	1	0	0.243909	2.729742	0.578456
56	6	0	2.377151	3.065630	-2.162040
57	1	0	1.857764	3.849746	-2.720382
58	1	0	3.179912	3.530115	-1.580939
59	1	0	2.821305	2.371052	-2.883365

TS_{sIJ}

Center Number	Atomic Number	Atomic Type	Coordinates (Angstroms) X	Y	Z
1	6	0	-3.908301	-2.022690	0.545650
2	6	0	-2.425023	-2.283159	0.979564
3	6	0	-2.949982	-0.346471	2.047612
4	6	0	-4.265669	-0.686525	1.272362
5	1	0	-4.022338	-1.963416	-0.541795

6	1	0	-4.536971	-2.848946	0.893385
7	1	0	-4.560734	0.108812	0.580407
8	1	0	-5.087669	-0.823979	1.982656
9	6	0	-2.474614	-1.760661	2.434212
10	1	0	-1.514633	-1.747483	2.956656
11	1	0	-3.205403	-2.302796	3.043760
12	6	0	-1.659589	-1.190318	0.232407
13	1	0	-1.739864	-1.172522	-0.844915
14	6	0	-1.957550	0.033982	0.958769
15	1	0	-2.067928	0.981598	0.433168
16	1	0	-2.086015	-3.305867	0.812433
17	1	0	-3.056736	0.382003	2.850695
18	6	0	0.389958	-1.462708	-0.141818
19	6	0	0.936040	-2.748161	0.268739
20	6	0	0.817537	-0.859680	-1.298449
21	6	0	0.595964	-3.390653	1.477505
22	6	0	1.877214	-3.392142	-0.583894
23	6	0	1.928068	-1.449327	-2.066526
24	6	0	1.110487	-4.630985	1.820754
25	1	0	-0.076503	-2.887133	2.157683
26	6	0	2.380250	-4.661411	-0.236438
27	6	0	1.996779	-5.272990	0.948596
28	1	0	0.828444	-5.094465	2.761036
29	1	0	3.086666	-5.162608	-0.885643
30	1	0	2.403432	-6.248433	1.199728
31	8	0	2.500064	-0.825219	-2.970277
32	7	0	2.322304	-2.742910	-1.732155
33	46	0	0.046351	-0.102944	1.424268
34	6	0	3.350362	-3.360952	-2.572943
35	1	0	4.269560	-3.524196	-2.000746
36	1	0	2.993416	-4.318350	-2.964802
37	1	0	3.552464	-2.675322	-3.392523
38	35	0	0.167078	1.419606	3.428981
39	6	0	0.171821	0.340061	-1.942227
40	6	0	-0.958419	0.041908	-2.729555
41	6	0	0.610388	1.681092	-1.861614
42	6	0	-1.718702	1.030748	-3.345572
43	6	0	-0.189171	2.666533	-2.469189
44	6	0	-1.342719	2.363553	-3.186005
45	1	0	-2.586054	0.762157	-3.942155
46	1	0	0.113120	3.706455	-2.390958
47	1	0	-1.925078	3.161488	-3.638776
48	6	0	1.945108	2.140838	-1.232340

49	1	0	-1.231417	-1.001943	-2.871835
50	8	0	2.707192	1.041744	-0.734197
51	1	0	3.020182	0.548097	-1.514426
52	6	0	1.710618	3.062862	-0.028993
53	1	0	2.676661	3.388472	0.370371
54	1	0	1.133825	3.949259	-0.311555
55	1	0	1.173633	2.540442	0.768058
56	6	0	2.796431	2.851695	-2.308723
57	1	0	2.322702	3.762435	-2.687172
58	1	0	3.762910	3.121624	-1.870474
59	1	0	2.968233	2.181186	-3.158814

sJ

Center Number	Atomic Number	Atomic Type	Coordinates (Angstroms) X	Y	Z
1	6	0	-4.783460	-1.977571	1.077612
2	6	0	-3.324179	-1.845838	1.635814
3	6	0	-3.806714	0.267329	0.967178
4	6	0	-5.117854	-0.521549	0.621041
5	1	0	-4.848019	-2.705691	0.263309
6	1	0	-5.457608	-2.312453	1.873010
7	1	0	-5.371160	-0.453674	-0.441154
8	1	0	-5.958688	-0.107857	1.187846
9	6	0	-3.402248	-0.442902	2.280275
10	1	0	-2.451655	-0.096171	2.712725
11	1	0	-4.168839	-0.368926	3.059045
12	6	0	-2.467899	-1.564990	0.405865
13	1	0	-2.056789	-2.348323	-0.222595
14	6	0	-2.771333	-0.275884	-0.009820
15	1	0	-2.633521	0.124675	-1.005470
16	1	0	-2.999940	-2.673054	2.269670
17	1	0	-3.919830	1.352393	0.993941
18	6	0	0.518219	-1.000428	-0.413543
19	6	0	1.053451	-2.305005	-0.086468
20	6	0	0.863929	-0.332349	-1.545778
21	6	0	0.728154	-3.020958	1.084921
22	6	0	1.941736	-2.911859	-1.018603
23	6	0	1.871207	-0.927778	-2.447523
24	6	0	1.232581	-4.287736	1.330638

25	1	0	0.079048	-2.546654	1.812975
26	6	0	2.444842	-4.202466	-0.761263
27	6	0	2.090894	-4.880093	0.396660
28	1	0	0.968967	-4.809886	2.245297
29	1	0	3.120505	-4.673068	-1.464027
30	1	0	2.494354	-5.872739	0.575904
31	8	0	2.317761	-0.306209	-3.421766
32	7	0	2.319187	-2.214271	-2.161327
33	46	0	-0.724031	-0.214674	0.954312
34	6	0	3.289519	-2.800965	-3.088404
35	1	0	4.236145	-2.999586	-2.575686
36	1	0	2.901408	-3.735336	-3.506341
37	1	0	3.450529	-2.079081	-3.885560
38	35	0	1.153945	0.566678	2.414371
39	6	0	0.187669	0.902653	-2.075808
40	6	0	-1.040968	0.652052	-2.718110
41	6	0	0.683985	2.226907	-2.051142
42	6	0	-1.829185	1.669251	-3.247241
43	6	0	-0.151597	3.243614	-2.549883
44	6	0	-1.390034	2.987027	-3.129671
45	1	0	-2.767455	1.434772	-3.743210
46	1	0	0.190442	4.272486	-2.498880
47	1	0	-1.990956	3.808277	-3.511374
48	6	0	2.092696	2.637781	-1.566167
49	1	0	-1.367518	-0.380959	-2.810446
50	8	0	2.923229	1.508162	-1.327577
51	1	0	3.045992	1.065183	-2.188074
52	6	0	2.013296	3.394476	-0.233062
53	1	0	3.020530	3.699904	0.070154
54	1	0	1.388820	4.289514	-0.325748
55	1	0	1.601170	2.751941	0.548674
56	6	0	2.790673	3.505351	-2.640080
57	1	0	2.296463	4.467333	-2.806590
58	1	0	3.815675	3.700176	-2.308886
59	1	0	2.827782	2.972758	-3.597815

---

sK

Center Number	Atomic Number	Atomic Type	Coordinates (Angstroms)		
			X	Y	Z

1	6	0	-0.147077	-0.868026	-0.546243
2	6	0	0.221941	-2.256429	-0.344992
3	6	0	-1.311652	-0.546628	-1.178439
4	6	0	1.407536	-2.651035	0.315073
5	6	0	-0.664987	-3.272772	-0.806949
6	6	0	-2.203257	-1.581398	-1.725331
7	6	0	1.702826	-3.987644	0.552492
8	1	0	2.122871	-1.875135	0.572723
9	6	0	-0.352160	-4.626923	-0.560665
10	6	0	0.809411	-4.975176	0.116038
11	1	0	2.634192	-4.262985	1.039352
12	1	0	-1.017493	-5.409736	-0.902859
13	1	0	1.028489	-6.025662	0.287413
14	8	0	-3.243385	-1.326392	-2.331085
15	7	0	-1.820448	-2.919236	-1.490681
16	46	0	0.743927	0.643716	0.355315
17	6	0	-2.704944	-3.957124	-2.019610
18	1	0	-2.162927	-4.605043	-2.716208
19	1	0	-3.118252	-4.567862	-1.209107
20	1	0	-3.513642	-3.450087	-2.541960
21	35	0	2.892566	0.387400	-0.899616
22	6	0	-1.733775	0.873736	-1.422845
23	6	0	-1.656346	1.306384	-2.755351
24	6	0	-2.177249	1.782817	-0.425676
25	6	0	-1.982498	2.606229	-3.128470
26	6	0	-2.502773	3.088796	-0.827480
27	6	0	-2.407396	3.504673	-2.154013
28	1	0	-1.903479	2.910420	-4.168317
29	1	0	-2.843351	3.810867	-0.095606
30	1	0	-2.665953	4.526675	-2.416940
31	6	0	-2.352815	1.386822	1.054212
32	1	0	-1.326170	0.596760	-3.507275
33	8	0	-1.062170	0.954874	1.635778
34	1	0	-0.515933	1.746979	1.918741
35	6	0	1.830848	2.077902	2.857569
36	8	0	1.137039	2.355381	1.797180
37	8	0	1.648034	1.078114	3.583802
38	6	0	2.933555	3.070344	3.189251
39	1	0	2.542183	4.092315	3.166800
40	1	0	3.376807	2.855153	4.163853
41	1	0	3.705079	3.005653	2.413120
42	19	0	0.356879	-1.063710	3.046024
43	6	0	-2.836975	2.532624	1.955151

44	1	0	-3.836653	2.871865	1.668601
45	1	0	-2.885821	2.173368	2.988162
46	1	0	-2.156039	3.389449	1.924063
47	6	0	-3.305824	0.194747	1.218010
48	1	0	-3.437624	-0.036186	2.282337
49	1	0	-4.284570	0.439010	0.794177
50	1	0	-2.932407	-0.693567	0.707685

TS_{sKL}

Center Number	Atomic Number	Atomic Type	Coordinates (Angstroms) X	Y	Z
1	6	0	1.940378	-0.489798	-1.345961
2	6	0	2.354810	-1.854383	-1.099895
3	6	0	0.751715	-0.199453	-1.939729
4	6	0	3.574950	-2.196526	-0.474802
5	6	0	1.470336	-2.903546	-1.489083
6	6	0	-0.135811	-1.277137	-2.414089
7	6	0	3.906469	-3.516890	-0.198479
8	1	0	4.277929	-1.395058	-0.265758
9	6	0	1.822734	-4.240406	-1.204469
10	6	0	3.017494	-4.538061	-0.561911
11	1	0	4.860610	-3.754456	0.262978
12	1	0	1.162303	-5.049284	-1.490753
13	1	0	3.265959	-5.576382	-0.359860
14	8	0	-1.199825	-1.068814	-2.992319
15	7	0	0.283239	-2.597404	-2.139036
16	46	0	2.902455	1.048924	-0.597462
17	6	0	-0.601052	-3.672356	-2.590347
18	1	0	-0.074966	-4.336044	-3.284119
19	1	0	-0.967287	-4.258244	-1.739940
20	1	0	-1.441128	-3.202200	-3.097202
21	35	0	4.961209	0.835553	-1.984864
22	6	0	0.296636	1.203520	-2.204904
23	6	0	0.226425	1.578629	-3.554961
24	6	0	-0.030046	2.151214	-1.199806
25	6	0	-0.133971	2.866019	-3.940330
26	6	0	-0.391820	3.442556	-1.615021
27	6	0	-0.441374	3.805290	-2.960193
28	1	0	-0.170685	3.128856	-4.993706

29	1	0	-0.643375	4.194846	-0.877700
30	1	0	-0.721567	4.819001	-3.233307
31	6	0	-0.061199	1.799191	0.303123
32	1	0	0.466861	0.837546	-4.310311
33	8	0	1.230249	1.285844	0.755366
34	1	0	1.895312	2.054448	1.235131
35	6	0	3.671819	2.341019	2.514794
36	8	0	2.968494	2.781936	1.513288
37	8	0	3.427612	1.288874	3.136135
38	6	0	4.864876	3.203799	2.886973
39	1	0	4.548911	4.242842	3.023833
40	1	0	5.342640	2.832245	3.795476
41	1	0	5.586997	3.193822	2.062459
42	19	0	2.244678	-0.782499	2.171556
43	6	0	-0.376850	3.001072	1.209599
44	1	0	-1.381770	3.392701	1.025267
45	1	0	-0.332069	2.673924	2.253826
46	1	0	0.350115	3.807703	1.079427
47	6	0	-1.102841	0.697727	0.576069
48	1	0	-1.137638	0.476578	1.651026
49	1	0	-2.099568	1.026304	0.264226
50	1	0	-0.869887	-0.220603	0.033378

sL

Center Number	Atomic Number	Atomic Type	Coordinates (Angstroms) X	Y	Z
1	6	0	1.898679	-0.478865	-1.540400
2	6	0	2.363777	-1.822886	-1.268490
3	6	0	0.652844	-0.238179	-2.032296
4	6	0	3.648407	-2.110373	-0.754134
5	6	0	1.469192	-2.906589	-1.514906
6	6	0	-0.256243	-1.355940	-2.353913
7	6	0	4.035965	-3.408636	-0.448830
8	1	0	4.344903	-1.284053	-0.645104
9	6	0	1.878580	-4.220831	-1.201574
10	6	0	3.139132	-4.463121	-0.670182
11	1	0	5.034904	-3.604144	-0.070343
12	1	0	1.211709	-5.055142	-1.379503
13	1	0	3.430523	-5.484950	-0.442876

14	8	0	-1.379026	-1.200010	-2.827583
15	7	0	0.216030	-2.653607	-2.053982
16	46	0	2.948901	1.107395	-1.047478
17	6	0	-0.685696	-3.765132	-2.357958
18	1	0	-0.216700	-4.457159	-3.064864
19	1	0	-0.953897	-4.310338	-1.445943
20	1	0	-1.581361	-3.336200	-2.802184
21	35	0	4.875921	0.847315	-2.616480
22	6	0	0.158016	1.138697	-2.347590
23	6	0	-0.125058	1.392263	-3.698685
24	6	0	0.006975	2.177460	-1.392786
25	6	0	-0.526293	2.649325	-4.139232
26	6	0	-0.401942	3.435101	-1.864456
27	6	0	-0.660335	3.679230	-3.212816
28	1	0	-0.727500	2.818528	-5.193305
29	1	0	-0.527931	4.255538	-1.169033
30	1	0	-0.968383	4.672064	-3.529829
31	6	0	0.213823	1.954310	0.124729
32	1	0	-0.017428	0.581708	-4.411694
33	8	0	1.537347	1.486758	0.436049
34	1	0	2.420300	2.331807	1.403519
35	6	0	3.584932	2.175643	2.975551
36	8	0	3.084698	2.877981	1.976067
37	8	0	3.295487	1.001100	3.209971
38	6	0	4.569167	2.959901	3.811064
39	1	0	4.117687	3.901586	4.138629
40	1	0	4.883354	2.370885	4.673565
41	1	0	5.442416	3.215563	3.200749
42	19	0	2.083252	-0.767529	1.738493
43	6	0	0.002833	3.240569	0.948380
44	1	0	-1.017733	3.627287	0.860454
45	1	0	0.182054	3.014625	2.004863
46	1	0	0.705994	4.024188	0.651095
47	6	0	-0.811980	0.909578	0.633389
48	1	0	-0.687804	0.766840	1.716929
49	1	0	-1.840199	1.245502	0.460869
50	1	0	-0.692801	-0.052624	0.127693

---

sN

Center Number	Atomic Number	Atomic Type	Coordinates (Angstroms)		
			X	Y	Z

1	6	0	-0.483103	-1.342546	-0.324494
2	6	0	-0.264739	-2.768517	-0.479773
3	6	0	-1.522519	-0.699728	-0.921500
4	6	0	0.785869	-3.463783	0.160506
5	6	0	-1.135462	-3.497556	-1.335514
6	6	0	-2.477596	-1.474977	-1.755388
7	6	0	0.993439	-4.819430	-0.045001
8	1	0	1.448696	-2.925664	0.835589
9	6	0	-0.917538	-4.875804	-1.533445
10	6	0	0.135889	-5.521948	-0.899645
11	1	0	1.812586	-5.326637	0.455267
12	1	0	-1.573833	-5.444328	-2.180161
13	1	0	0.285046	-6.584717	-1.069675
14	8	0	-3.476283	-0.975274	-2.270638
15	7	0	-2.195909	-2.840194	-1.944560
16	46	0	0.975833	-0.489300	0.683467
17	6	0	-3.117012	-3.588726	-2.800022
18	1	0	-2.578700	-4.033685	-3.643353
19	1	0	-3.613900	-4.382900	-2.232189
20	1	0	-3.860011	-2.884114	-3.166596
21	35	0	3.450784	-0.351068	1.684741
22	6	0	-1.801776	0.765245	-0.834615
23	6	0	-1.970733	1.442193	-2.055903
24	6	0	-1.929509	1.490302	0.376018
25	6	0	-2.262002	2.800135	-2.111314
26	6	0	-2.241541	2.856820	0.293989
27	6	0	-2.412201	3.510950	-0.923554
28	1	0	-2.381246	3.291393	-3.073063
29	1	0	-2.355338	3.437518	1.201194
30	1	0	-2.656319	4.569943	-0.937934
31	6	0	-1.644757	0.848161	1.748112
32	1	0	-1.881236	0.879914	-2.978260
33	8	0	-0.218361	0.670600	1.871160
34	1	0	0.517804	2.146131	1.501690
35	6	0	1.579063	3.563093	2.299506
36	8	0	0.982307	3.011202	1.255314
37	8	0	1.539287	3.099819	3.438315
38	6	0	2.335601	4.818894	1.942077
39	1	0	1.741593	5.449047	1.274426
40	1	0	2.604857	5.363749	2.847931
41	1	0	3.248687	4.539430	1.403677
42	19	0	1.415748	0.560523	4.028318

43	6	0	-1.994320	1.783879	2.931054
44	1	0	-3.053768	2.060675	2.919448
45	1	0	-1.808322	1.245534	3.869070
46	1	0	-1.389564	2.694037	2.938267
47	6	0	-2.423777	-0.480615	1.987006
48	1	0	-3.011712	-0.790297	1.122020
49	1	0	-1.722607	-1.286110	2.224738
50	1	0	-3.116378	-0.376859	2.829997

sO

Center Number	Atomic Number	Atomic Type	Coordinates (Angstroms)		
			X	Y	Z
1	6	0	-0.127843	-0.447429	0.337178
2	6	0	0.236186	-1.820342	0.603751
3	6	0	-1.391580	-0.094925	-0.031657
4	6	0	1.521535	-2.206095	1.036977
5	6	0	-0.751277	-2.829193	0.420641
6	6	0	-2.423103	-1.135294	-0.235599
7	6	0	1.841384	-3.534413	1.274697
8	1	0	2.257761	-1.424015	1.195844
9	6	0	-0.413440	-4.177254	0.658564
10	6	0	0.865777	-4.519842	1.078140
11	1	0	2.836372	-3.805070	1.615534
12	1	0	-1.151442	-4.957770	0.521479
13	1	0	1.101146	-5.565295	1.259210
14	8	0	-3.573121	-0.890552	-0.598173
15	7	0	-2.028786	-2.467140	0.011701
16	46	0	1.213423	0.989041	0.547585
17	6	0	-3.054853	-3.495202	-0.156406
18	1	0	-2.762930	-4.211563	-0.932418
19	1	0	-3.217741	-4.033322	0.783450
20	1	0	-3.970231	-2.987411	-0.452541
21	6	0	-1.803074	1.317942	-0.298964
22	6	0	-2.353360	1.578138	-1.565622
23	6	0	-1.638989	2.386568	0.623285
24	6	0	-2.720703	2.860812	-1.960454
25	6	0	-2.026565	3.668132	0.200098
26	6	0	-2.553173	3.915106	-1.067345
27	1	0	-3.138171	3.028738	-2.949911

28	1	0	-1.921950	4.506477	0.877440
29	1	0	-2.836343	4.927340	-1.345396
30	6	0	-1.085149	2.182233	2.057637
31	1	0	-2.501967	0.746727	-2.246380
32	8	0	0.263672	1.738541	2.069481
33	6	0	2.140522	-0.123860	-2.099523
34	8	0	2.535039	0.390008	-1.035717
35	1	0	1.093832	-0.419531	-2.234902
36	7	0	2.935286	-0.363379	-3.152100
37	6	0	2.415646	-0.974607	-4.367599
38	1	0	2.558931	-0.304342	-5.223206
39	1	0	2.933427	-1.919125	-4.571826
40	1	0	1.348032	-1.176306	-4.254286
41	6	0	4.353302	-0.021256	-3.122744
42	1	0	4.585387	0.432331	-2.159969
43	1	0	4.960105	-0.923735	-3.259763
44	1	0	4.584569	0.685602	-3.927780
45	6	0	-1.037373	3.496671	2.869769
46	1	0	-2.032042	3.929403	3.026952
47	1	0	-0.604294	3.268593	3.848044
48	1	0	-0.392360	4.235555	2.384862
49	6	0	-1.978639	1.193265	2.845518
50	1	0	-2.997558	1.584207	2.953906
51	1	0	-2.037122	0.218136	2.360068
52	1	0	-1.550558	1.048369	3.843388

TS_{sOP}

Center Number	Atomic Number	Atomic Type	Coordinates (Angstroms) X	Y	Z
1	6	0	-0.335403	-0.441411	0.789436
2	6	0	-0.069663	-1.767622	1.298078
3	6	0	-1.460498	-0.154229	0.054884
4	6	0	1.022595	-2.065239	2.134337
5	6	0	-0.960329	-2.809713	0.922965
6	6	0	-2.352747	-1.230818	-0.373944
7	6	0	1.247852	-3.353141	2.596001
8	1	0	1.682807	-1.250727	2.415651
9	6	0	-0.713068	-4.117676	1.390280
10	6	0	0.373695	-4.379241	2.214846

11	1	0	2.092230	-3.561731	3.246151
12	1	0	-1.374273	-4.929340	1.113443
13	1	0	0.538756	-5.394689	2.565439
14	8	0	-3.330307	-1.065753	-1.109567
15	7	0	-2.043838	-2.529383	0.098657
16	46	0	1.215095	0.830172	0.675154
17	6	0	-2.946948	-3.604988	-0.301722
18	1	0	-2.407789	-4.378066	-0.861173
19	1	0	-3.417464	-4.064142	0.574927
20	1	0	-3.711433	-3.159624	-0.935052
21	6	0	-1.789177	1.259011	-0.252310
22	6	0	-2.294684	1.627882	-1.509962
23	6	0	-1.567420	2.260191	0.730081
24	6	0	-2.559772	2.960356	-1.818951
25	6	0	-1.840537	3.592618	0.394206
26	6	0	-2.325222	3.948932	-0.865482
27	1	0	-2.949871	3.221539	-2.799594
28	1	0	-1.684975	4.375029	1.128456
29	1	0	-2.526475	4.993223	-1.090819
30	6	0	-1.153677	1.884424	2.169487
31	1	0	-2.496814	0.849287	-2.235241
32	8	0	-0.120409	0.884941	2.216241
33	6	0	1.923954	0.183955	-2.139539
34	8	0	2.442710	0.547216	-1.067215
35	1	0	0.877089	-0.141896	-2.184725
36	7	0	2.574679	0.149976	-3.311963
37	6	0	1.911818	-0.292106	-4.531001
38	1	0	1.916319	0.508823	-5.279600
39	1	0	2.424299	-1.166053	-4.950281
40	1	0	0.876159	-0.562499	-4.312529
41	6	0	3.970517	0.561335	-3.412033
42	1	0	4.315140	0.871399	-2.426233
43	1	0	4.583825	-0.273511	-3.770383
44	1	0	4.065867	1.396768	-4.115185
45	6	0	-0.601422	3.079662	2.968192
46	1	0	-1.371180	3.827184	3.190386
47	1	0	-0.213074	2.703412	3.919268
48	1	0	0.220214	3.565065	2.430603
49	6	0	-2.371616	1.293856	2.914767
50	1	0	-3.181292	2.029232	2.980415
51	1	0	-2.753151	0.408363	2.399882
52	1	0	-2.074467	1.003963	3.928570

----

sP

Center Number	Atomic Number	Atomic Type	Coordinates (Angstroms) X	Y	Z
1	6	0	-0.582087	-0.209625	1.269080
2	6	0	-0.315952	-1.540689	1.807036
3	6	0	-1.529262	-0.011592	0.232896
4	6	0	0.576492	-1.740289	2.872451
5	6	0	-1.000333	-2.648450	1.246068
6	6	0	-2.232808	-1.185514	-0.334873
7	6	0	0.826604	-3.014227	3.366504
8	1	0	1.067391	-0.871489	3.296431
9	6	0	-0.733395	-3.933701	1.753160
10	6	0	0.171331	-4.110451	2.796675
11	1	0	1.523560	-3.154928	4.187643
12	1	0	-1.234912	-4.799449	1.339074
13	1	0	0.357760	-5.113801	3.170337
14	8	0	-3.080316	-1.102602	-1.226544
15	7	0	-1.929010	-2.446622	0.223443
16	46	0	0.473241	-0.156887	-0.609540
17	6	0	-2.626225	-3.597024	-0.343622
18	1	0	-1.915199	-4.294068	-0.802877
19	1	0	-3.192821	-4.125655	0.430847
20	1	0	-3.308791	-3.219582	-1.101688
21	6	0	-2.037370	1.367369	0.012573
22	6	0	-2.722033	1.753963	-1.153546
23	6	0	-1.804983	2.332501	1.019457
24	6	0	-3.171370	3.062389	-1.315336
25	6	0	-2.260473	3.641196	0.838548
26	6	0	-2.941358	4.013350	-0.321263
27	1	0	-3.703037	3.337263	-2.222921
28	1	0	-2.091068	4.383964	1.612202
29	1	0	-3.288758	5.036079	-0.442039
30	6	0	-1.129754	1.901103	2.318971
31	1	0	-2.907812	1.009688	-1.915602
32	8	0	-0.167324	0.839003	2.058945
33	6	0	1.874755	-0.121565	-3.379977
34	8	0	2.034443	-0.134410	-2.152975
35	1	0	0.874577	-0.126759	-3.835024
36	7	0	2.883038	-0.099987	-4.275197

37	6	0	2.626538	-0.084841	-5.706317
38	1	0	3.051384	0.816329	-6.165360
39	1	0	3.072876	-0.963546	-6.187947
40	1	0	1.549196	-0.095504	-5.889787
41	6	0	4.272615	-0.088997	-3.836377
42	1	0	4.292438	-0.100321	-2.747010
43	1	0	4.798579	-0.970115	-4.222926
44	1	0	4.778293	0.811813	-4.204132
45	6	0	-0.271002	2.995064	2.958553
46	1	0	-0.886043	3.811995	3.346611
47	1	0	0.288627	2.565356	3.795192
48	1	0	0.440897	3.400027	2.233442
49	6	0	-2.176725	1.373711	3.317774
50	1	0	-2.890515	2.165149	3.570015
51	1	0	-2.740461	0.538843	2.889419
52	1	0	-1.686812	1.030753	4.235825

3a

Center Number	Atomic Number	Atomic Type	Coordinates (Angstroms) X	Y	Z
1	6	0	-0.309481	-0.479282	0.078889
2	6	0	1.013255	-0.486447	-0.354360
3	6	0	1.740379	0.701906	-0.392496
4	6	0	1.201947	1.945387	-0.009483
5	6	0	-0.136229	1.898175	0.412097
6	6	0	-0.888785	0.727786	0.465896
7	1	0	-0.891623	-1.395355	0.118958
8	1	0	1.487316	-1.414409	-0.661347
9	1	0	2.768504	0.661275	-0.730355
10	1	0	-1.917084	0.762316	0.807356
11	6	0	2.068667	3.230845	-0.095697
12	8	0	2.057188	3.943829	1.142525
13	1	0	1.161123	4.283161	1.296512
14	35	0	-1.155301	3.520863	0.996199
15	6	0	1.578712	4.123070	-1.255803
16	1	0	1.663604	3.598382	-2.214080
17	1	0	0.535221	4.423019	-1.128684
18	1	0	2.194413	5.028041	-1.296532
19	6	0	3.558763	2.923777	-0.303507

20	1	0	4.102053	3.870982	-0.249755
21	1	0	3.940392	2.270807	0.487116
22	1	0	3.759613	2.469667	-1.278178

---

8. NMR Charts

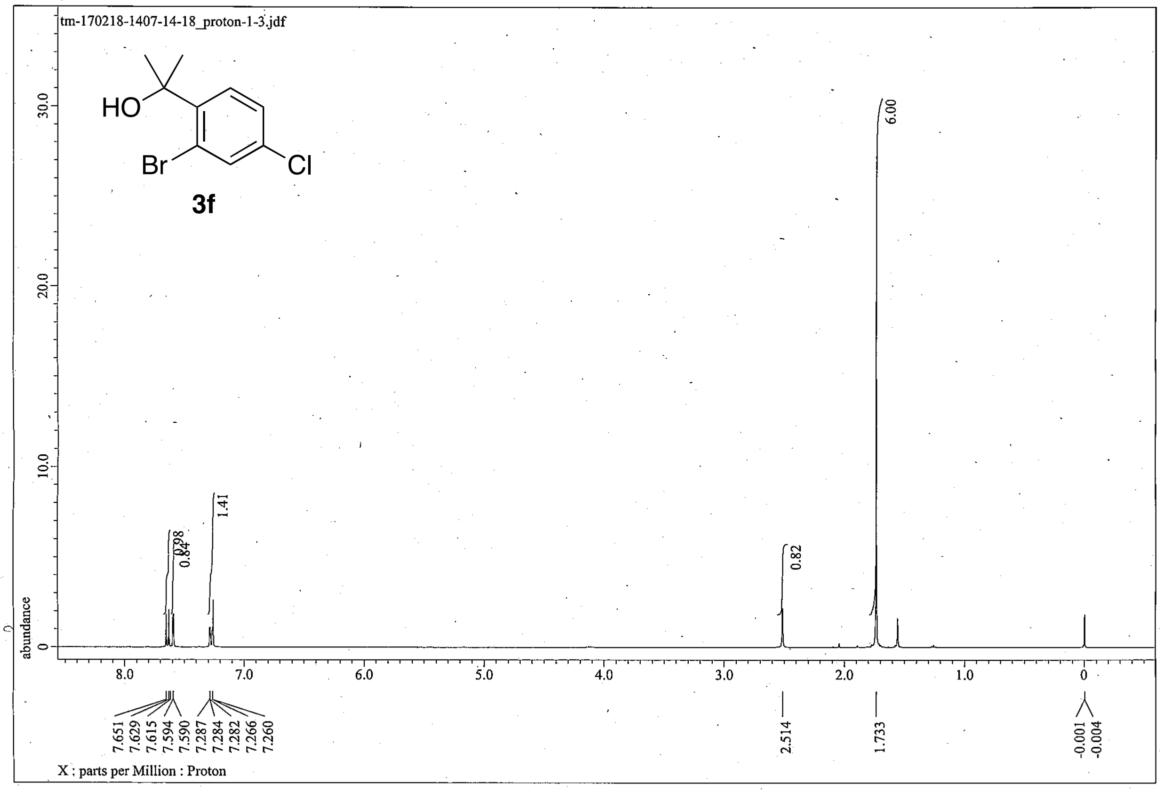
tm-170218-1407-14-18_proton-1-3.jdf
HO
Br
Cl
3f
6.00
1.41
0.82
abundance
X : parts per Million : Proton
7.651
7.629
7.615
7.594
7.590
7.287
7.284
7.282
7.266
7.260
2.514
1.733
-0.001
-0.004

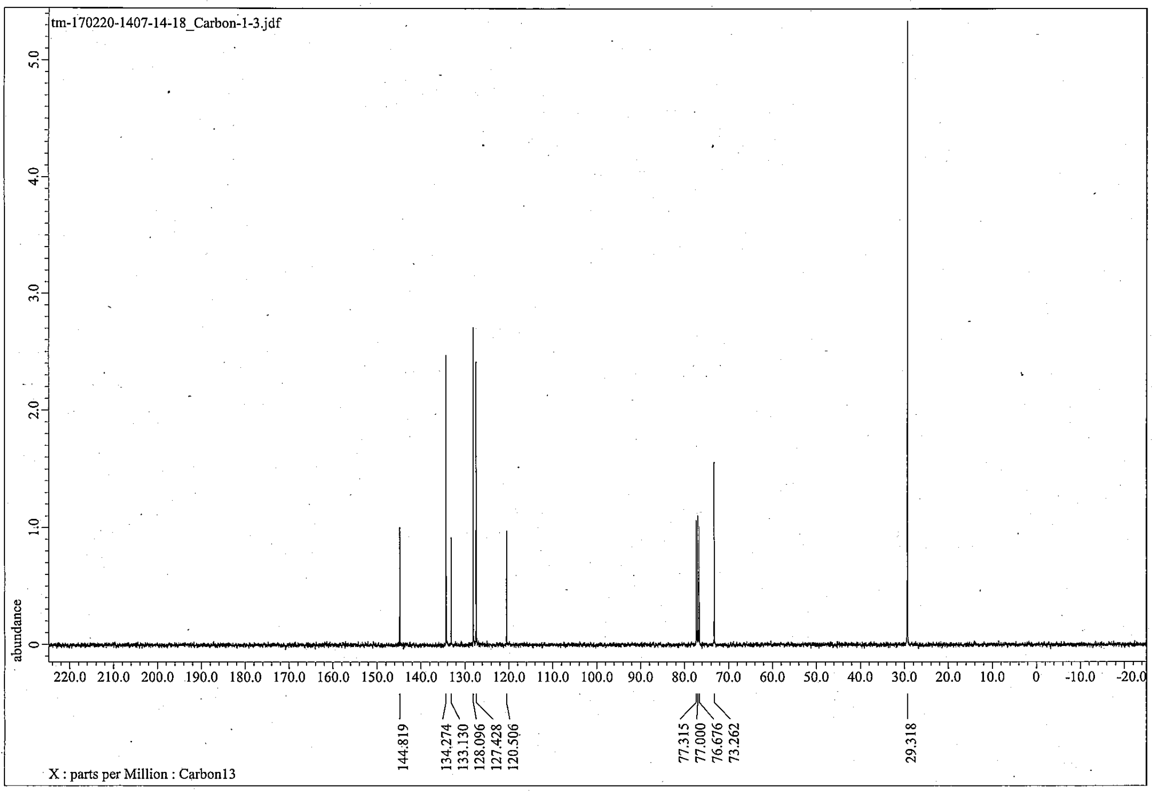
tm-170220-1407-14-18_Carbon-1-3.jdf
abundance
X : parts per Million : Carbon13
144.819
134.274
133.130
128.096
127.428
120.506
77.315
77.000
76.676
73.262
29.318

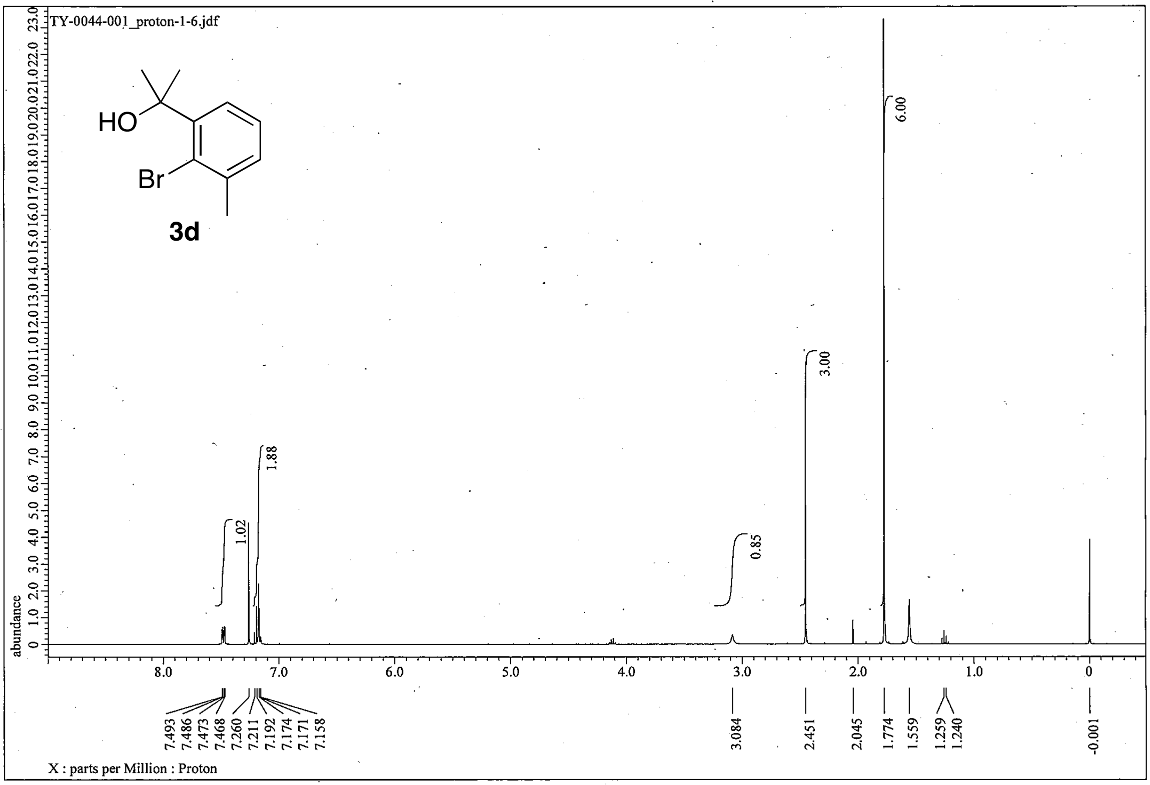
TY-0044-001_proton-1-6.jdf
HO
Br
3d
X : parts per Million : Proton
abundance
1.02
1.88
0.85
3.00
6.00
7.493
7.486
7.473
7.468
7.260
7.211
7.192
7.174
7.171
7.158
3.084
2.451
2.045
1.774
1.559
1.259
1.240
-0.001

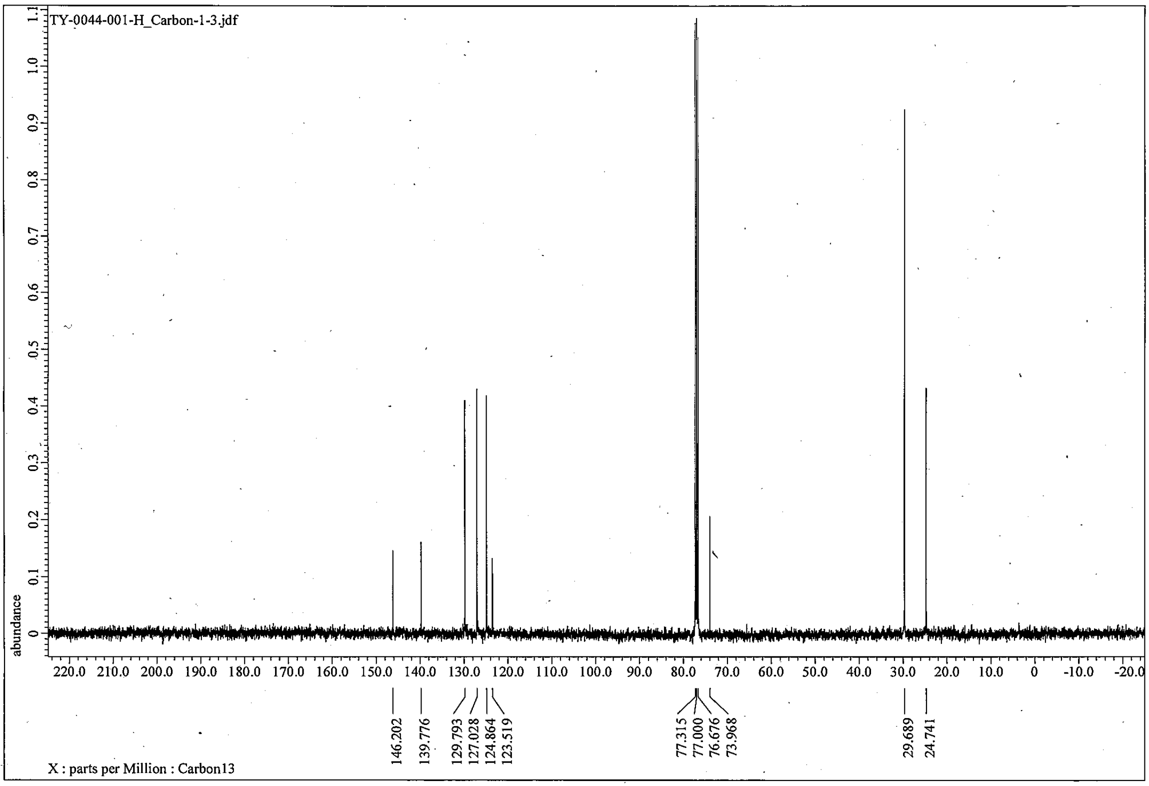
TY-0044-001-H_Carbon-1-3.jdf
X : parts per Million : Carbon13
abundance
146.202
139.776
129.793
127.028
124.864
123.519
77.315
77.000
76.676
73.968
29.689
24.741

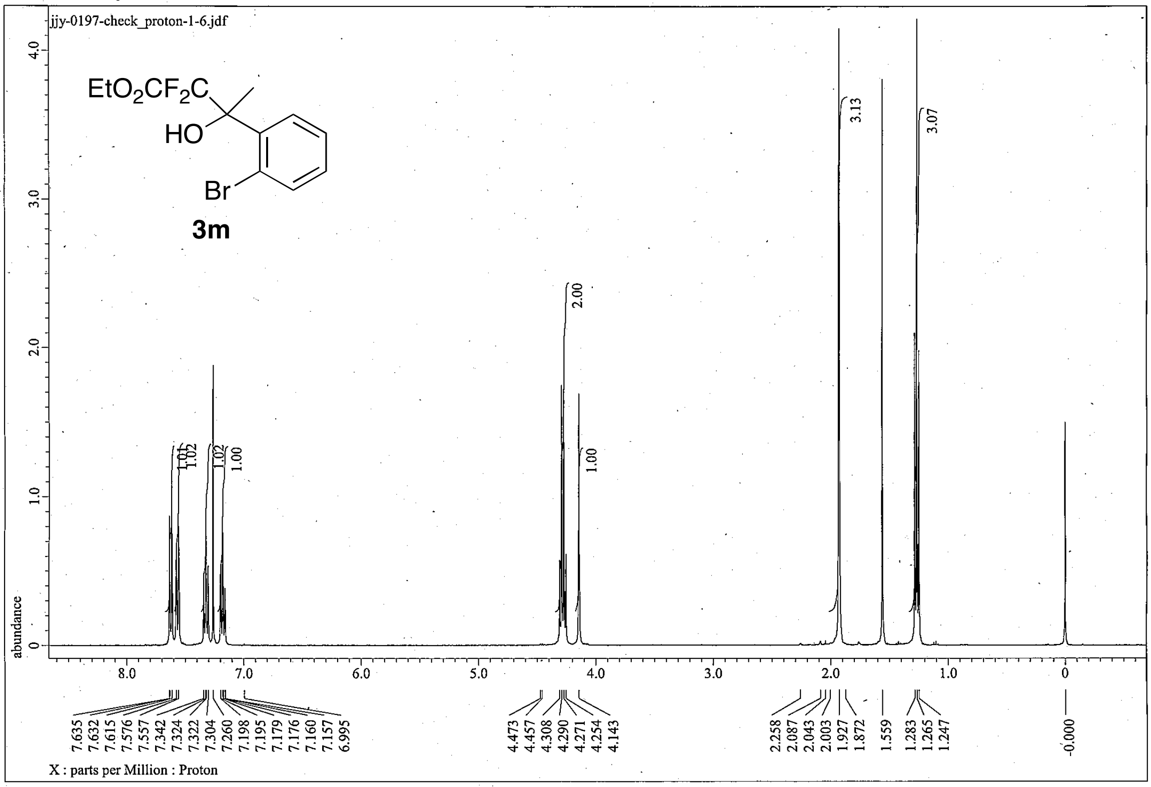
jjy-0197-check_proton-1-6.jdf
EtO2CF2C
HO
Br
3m
3.13
3.07
2.00
1.00
1.01
1.02
1.02
1.00
7.635
7.632
7.615
7.576
7.557
7.342
7.324
7.322
7.304
7.260
7.198
7.195
7.179
7.176
7.160
7.157
6.995
4.473
4.457
4.308
4.290
4.271
4.254
4.143
2.258
2.087
2.043
2.003
1.927
1.872
1.559
1.283
1.265
1.247
-0.000
X : parts per Million : Proton
abundance

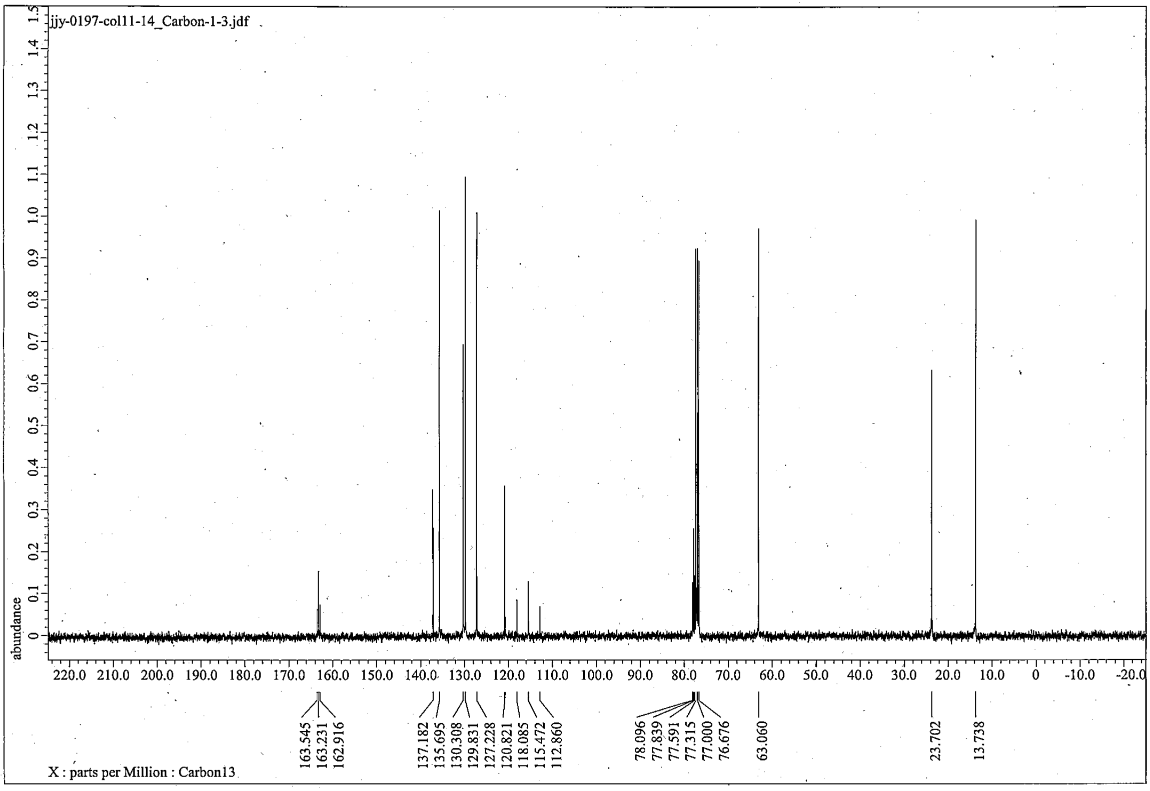
jjy-0197-col11-14_Carbon-1-3.jdf
163.545
163.231
162.916
137.182
135.695
130.308
129.831
127.228
120.821
118.085
115.472
112.860
78.096
77.839
77.591
77.315
77.000
76.676
63.060
23.702
13.738
X : parts per Million : Carbon13
abundance

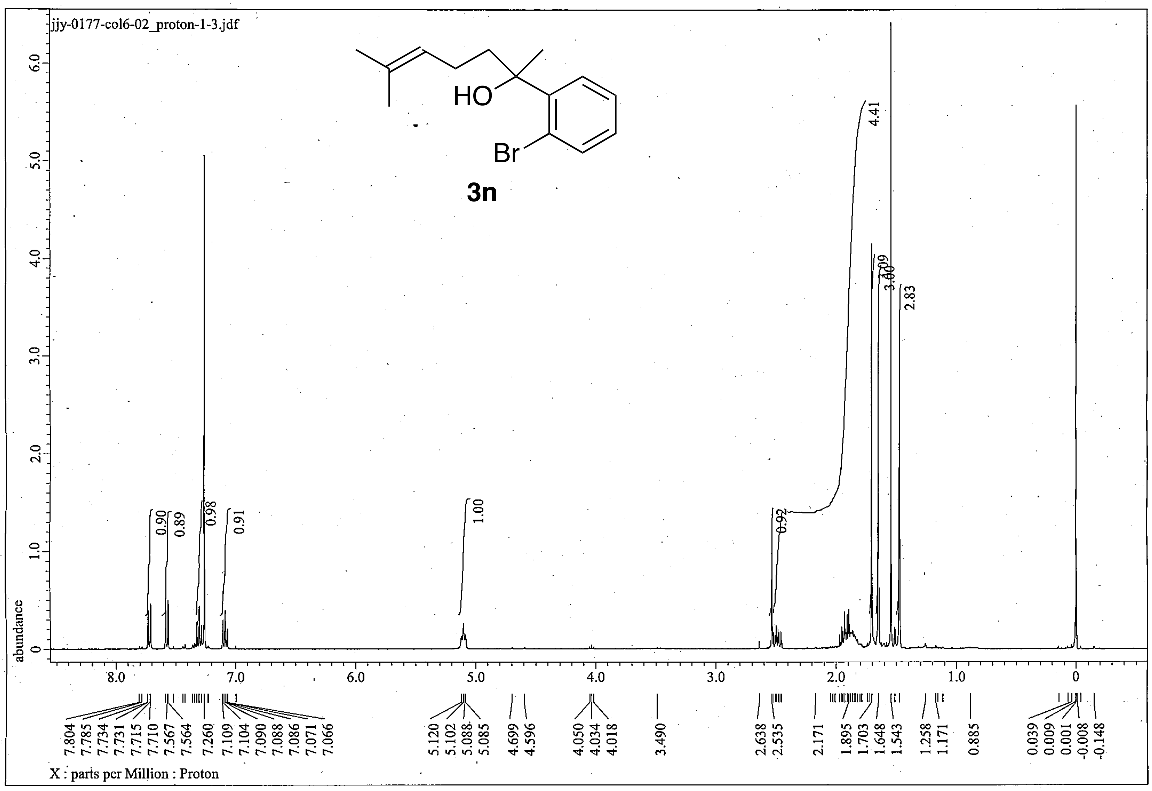
jjy-0177-col6-02_proton-1-3.jdf
HO
Br
3n
X : parts per Million : Proton

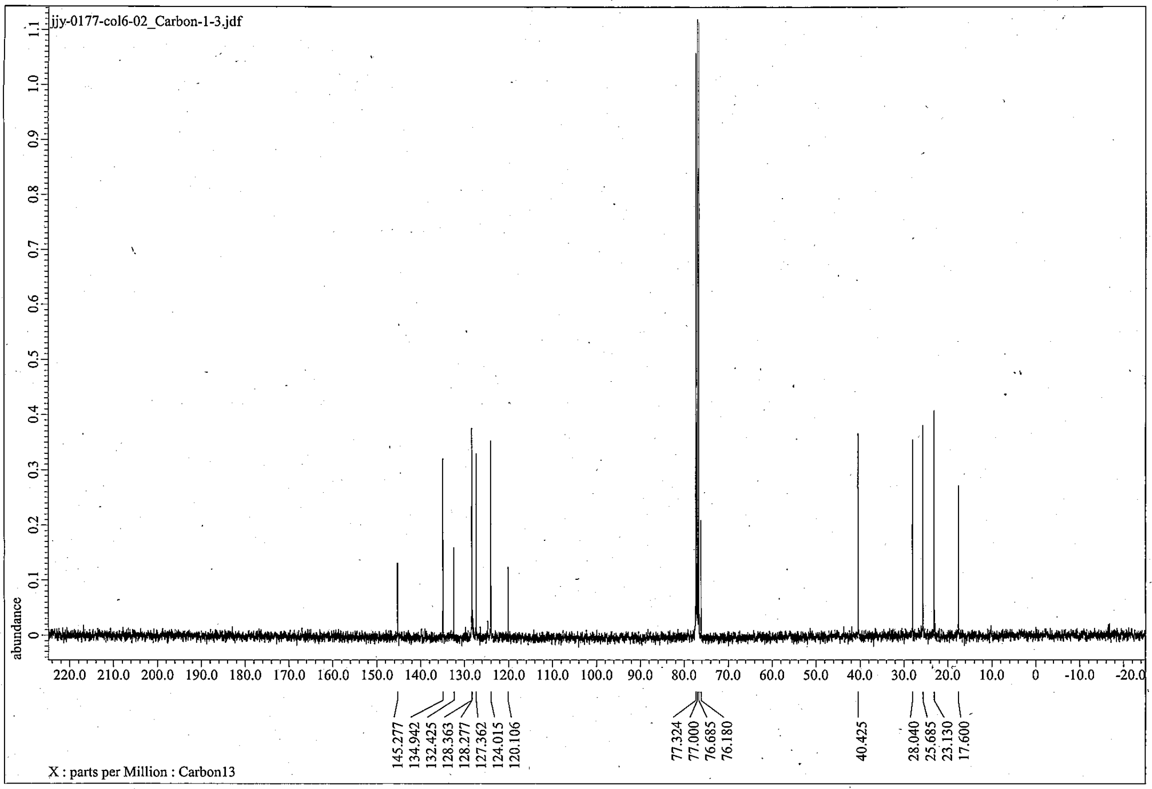
jjy-0177-col6-02_Carbon-1-3.jdf
X : parts per Million : Carbon13

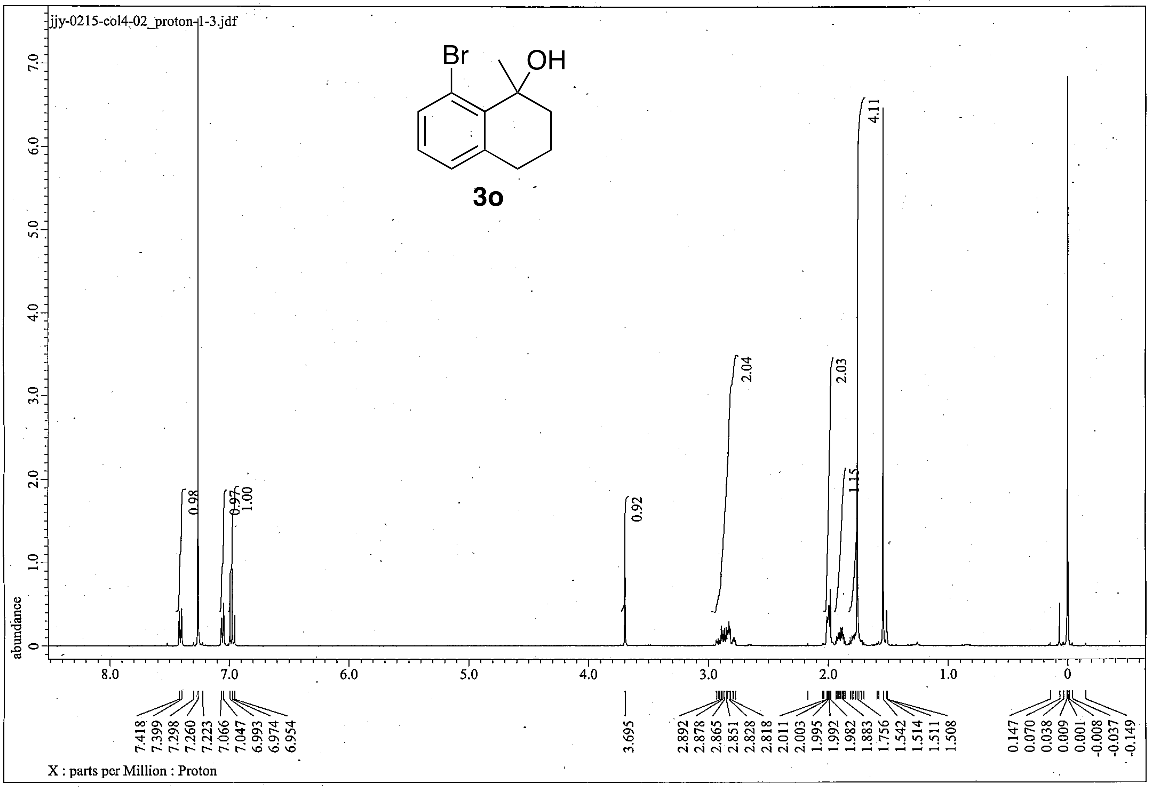
jjy-0215-col4-02_proton-1-3.jdf
Br
OH
3o
abundance
X : parts per Million : Proton

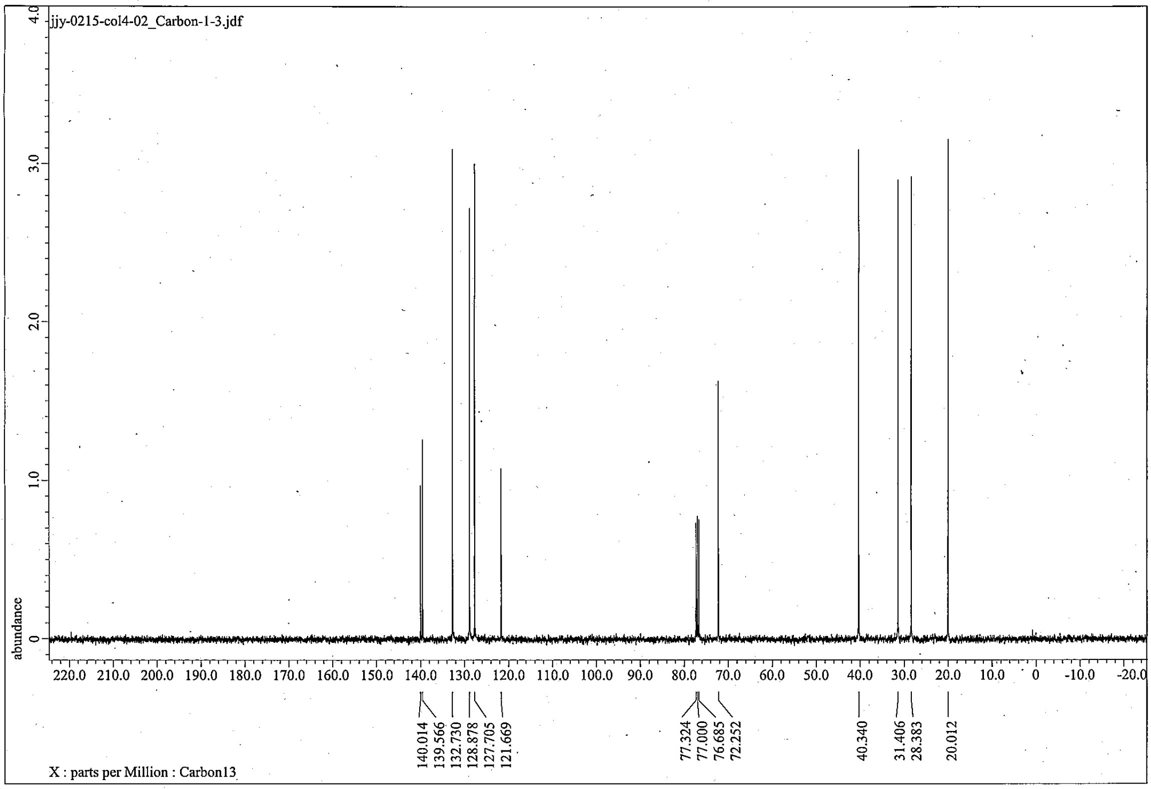
jjy-0215-col4-02_Carbon-1-3.jdf
abundance
140.014
139.566
132.730
128.878
127.705
121.669
77.324
77.000
76.685
72.252
40.340
31.406
28.383
20.012
X : parts per Million : Carbon13

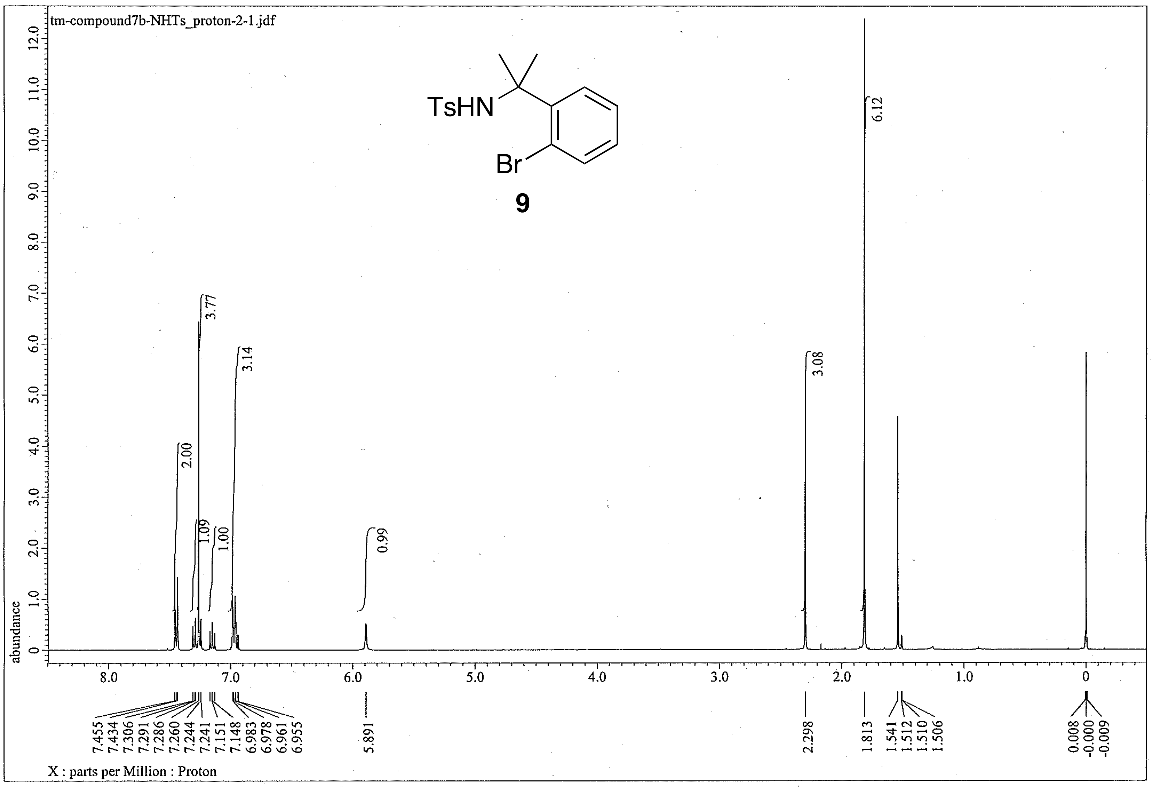
tm-compound7b-NHTs_proton-2-1.jdf
TsHN
Br
9
X : parts per Million : Proton
abundance

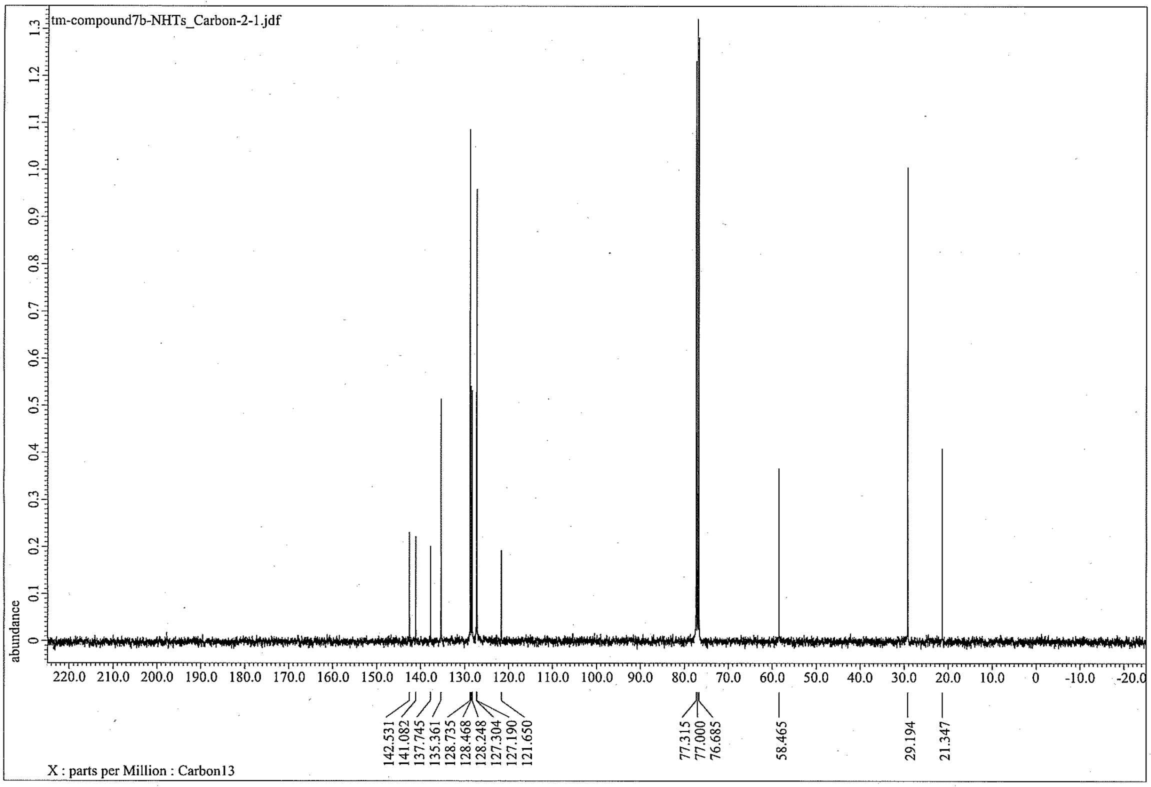
tm-compound7b-NHTs_Carbon-2-1.jdf
X : parts per Million : Carbon13
abundance

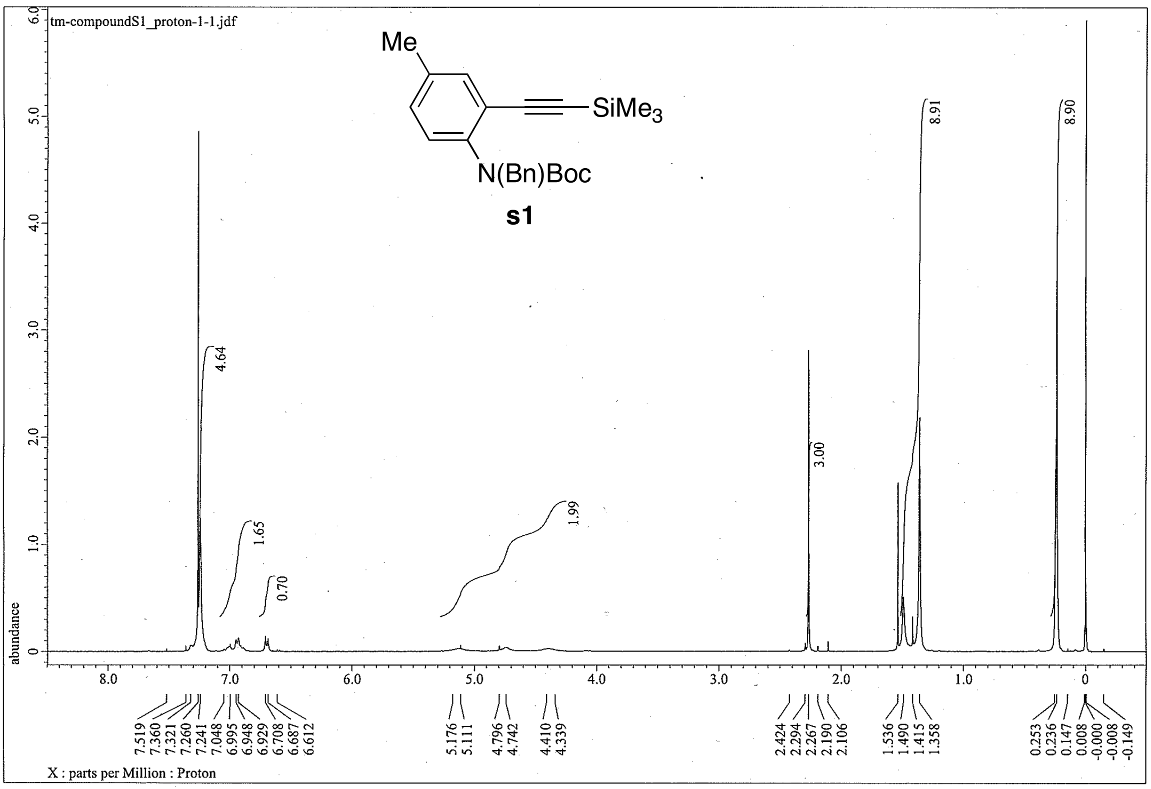
tm-compoundS1_proton-1-1.jdf
Me
SiMe3
N(Bn)Boc
s1
X : parts per Million : Proton
abundance

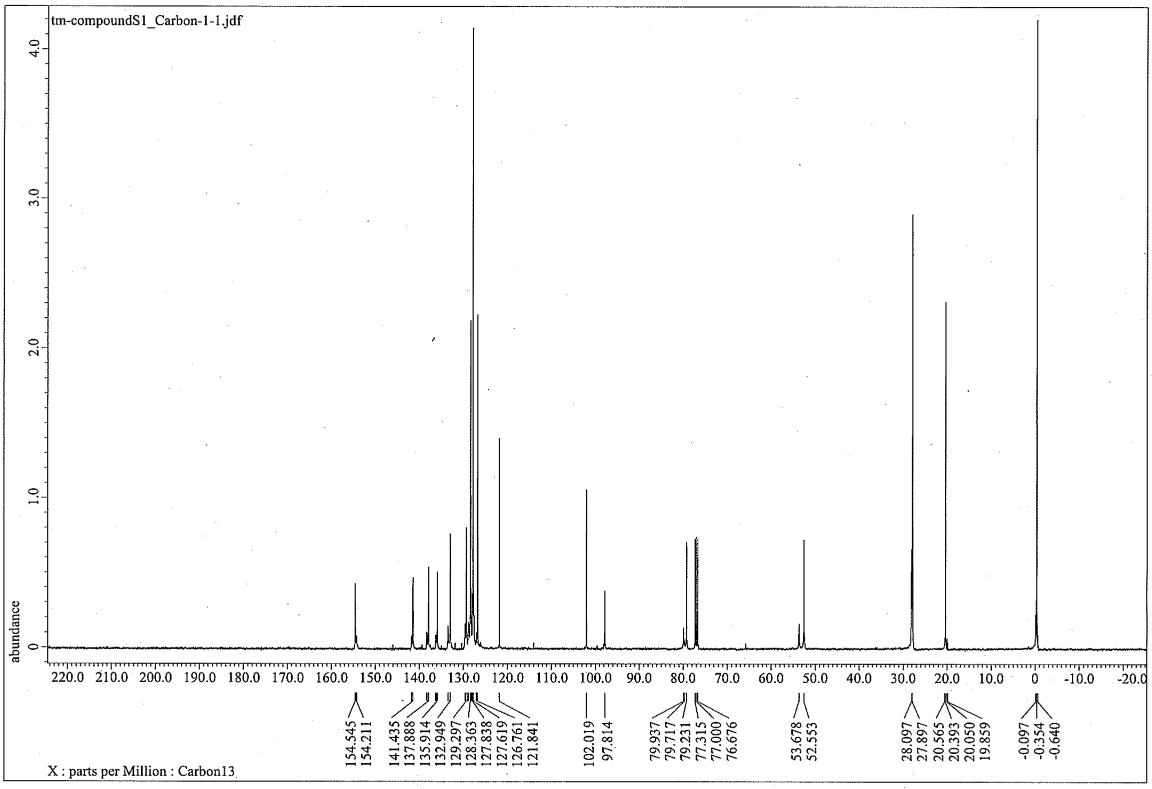
tm-compoundS1_Carbon-1-1.jdf
X : parts per Million : Carbon13
abundance

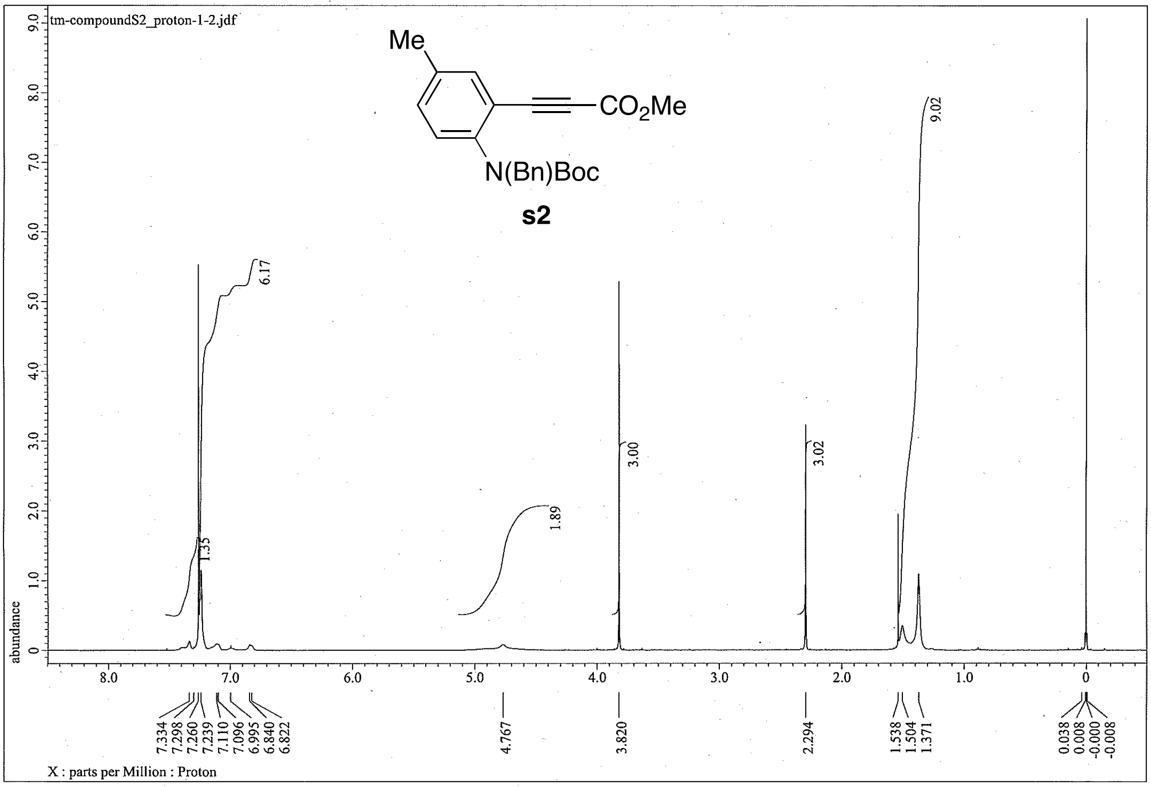
tm-compoundS2_proton-1-2.jdf
Me
CO2Me
N(Bn)Boc
s2
6.17
1.35
1.89
3.00
3.02
9.02
abundance
X : parts per Million : Proton
7.334
7.298
7.260
7.239
7.110
7.096
6.995
6.840
6.822
4.767
3.820
2.294
1.538
1.504
1.371
0.038
0.008
-0.000
-0.008

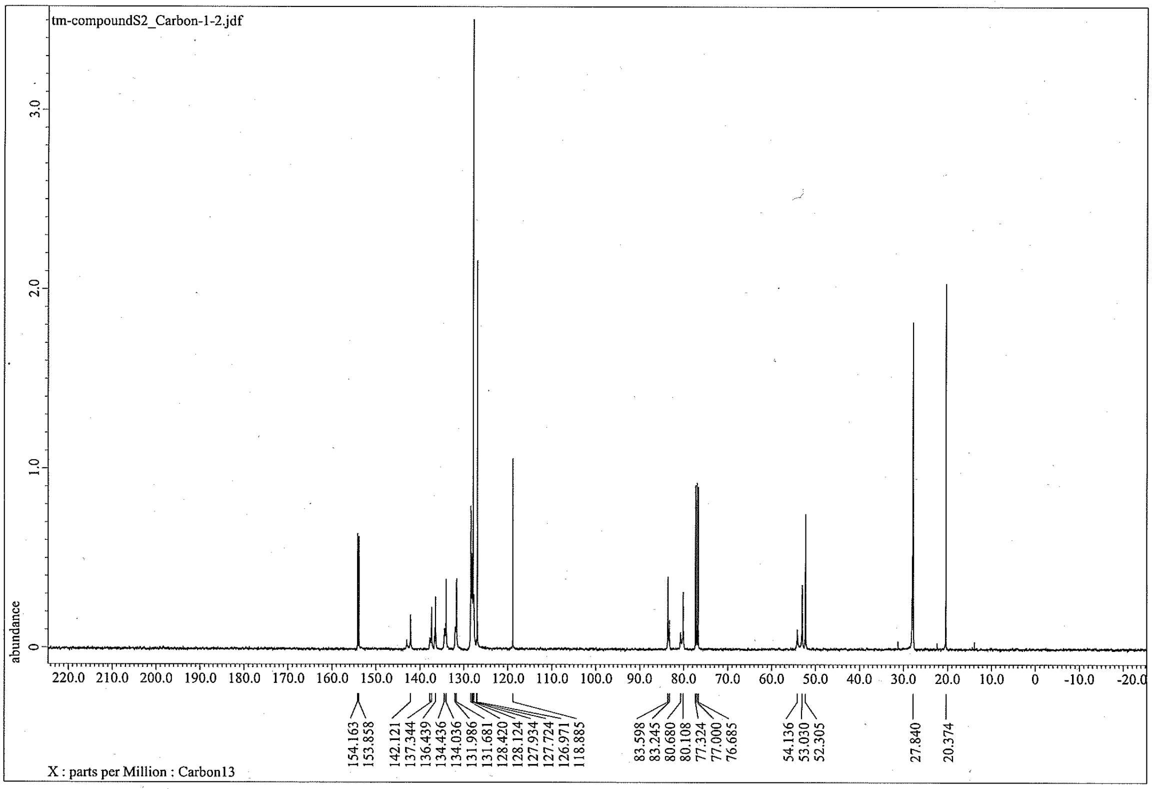
tm-compoundS2_Carbon-1-2.jdf
abundance
X : parts per Million : Carbon13
154.163
153.858
142.121
137.344
136.439
134.436
134.036
131.986
131.681
128.420
128.124
127.934
127.724
126.971
118.885
83.598
83.245
80.680
80.108
77.324
77.000
76.685
54.136
53.030
52.305
27.840
20.374

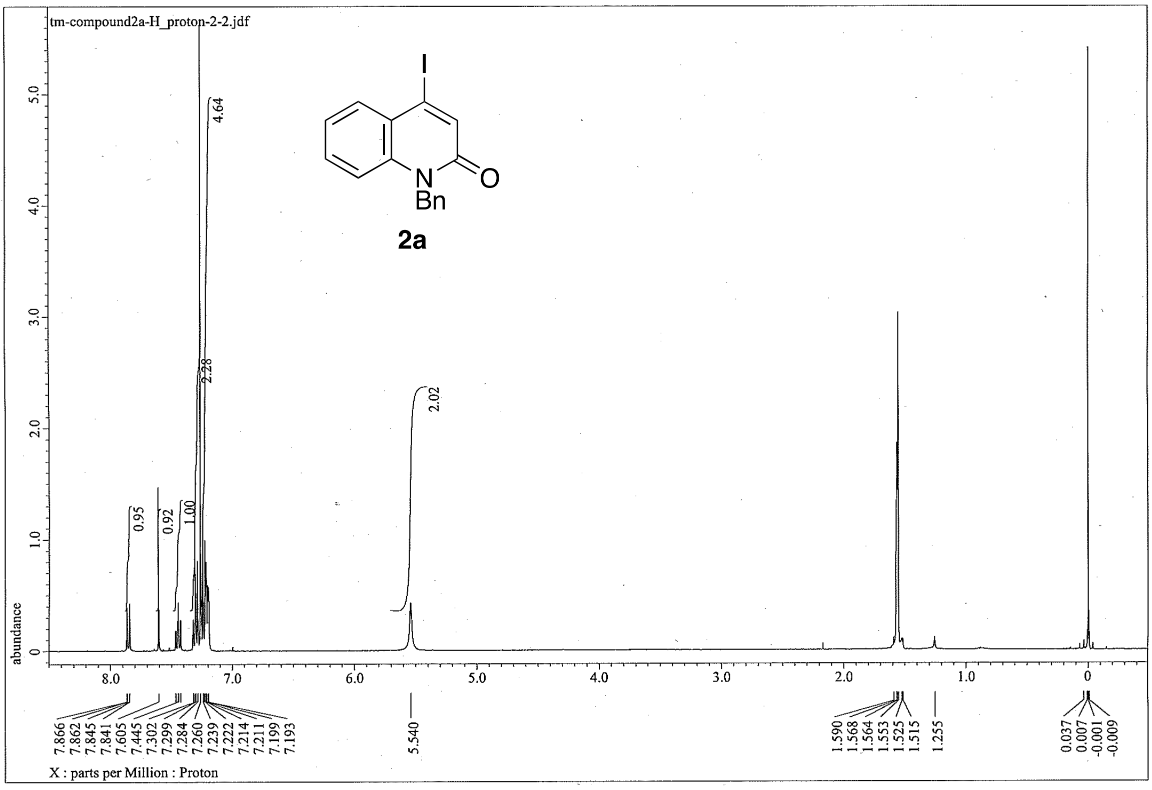
tm-compound2a-H_proton-2-2.jdf
2a
abundance
X : parts per Million : Proton

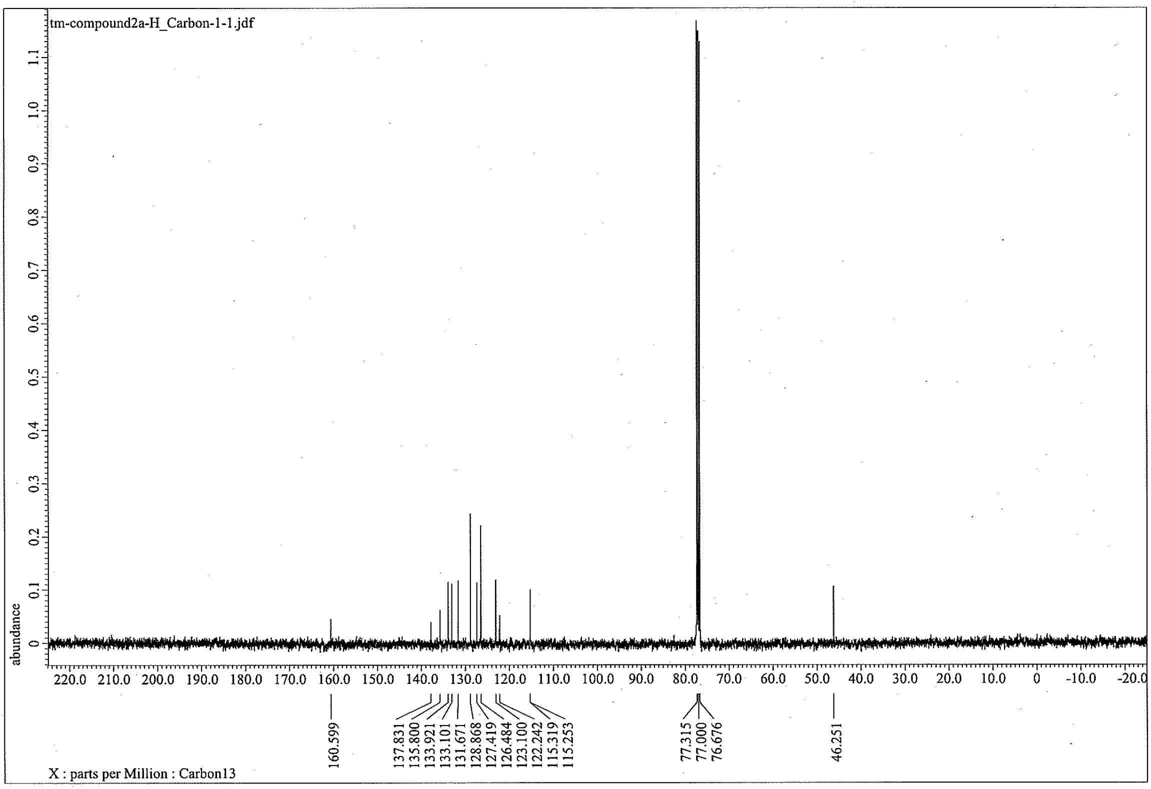
tm-compound2a-H_Carbon-1-1.jdf
abundance
X : parts per Million : Carbon13

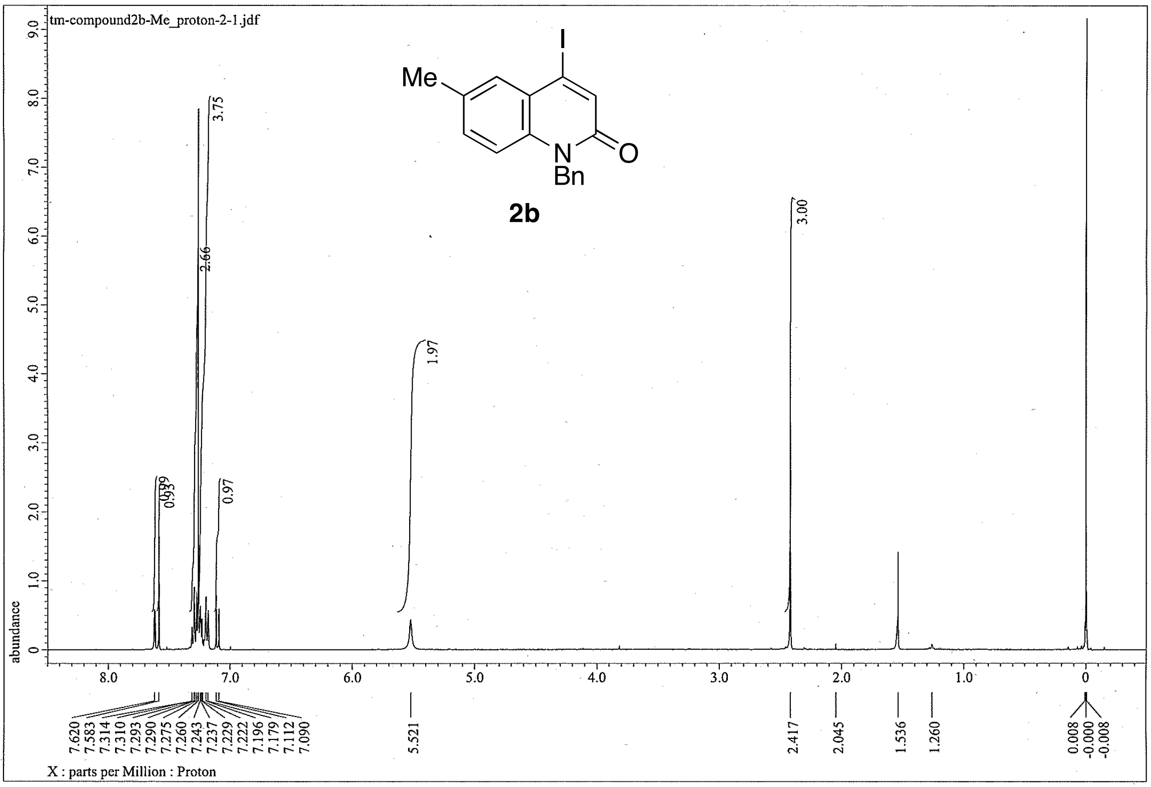
tm-compound2b-Me_proton-2-1.jdf
Me
I
N
O
Bn
2b
abundance
X : parts per Million : Proton
5.521
2.417
2.045
1.536
1.260

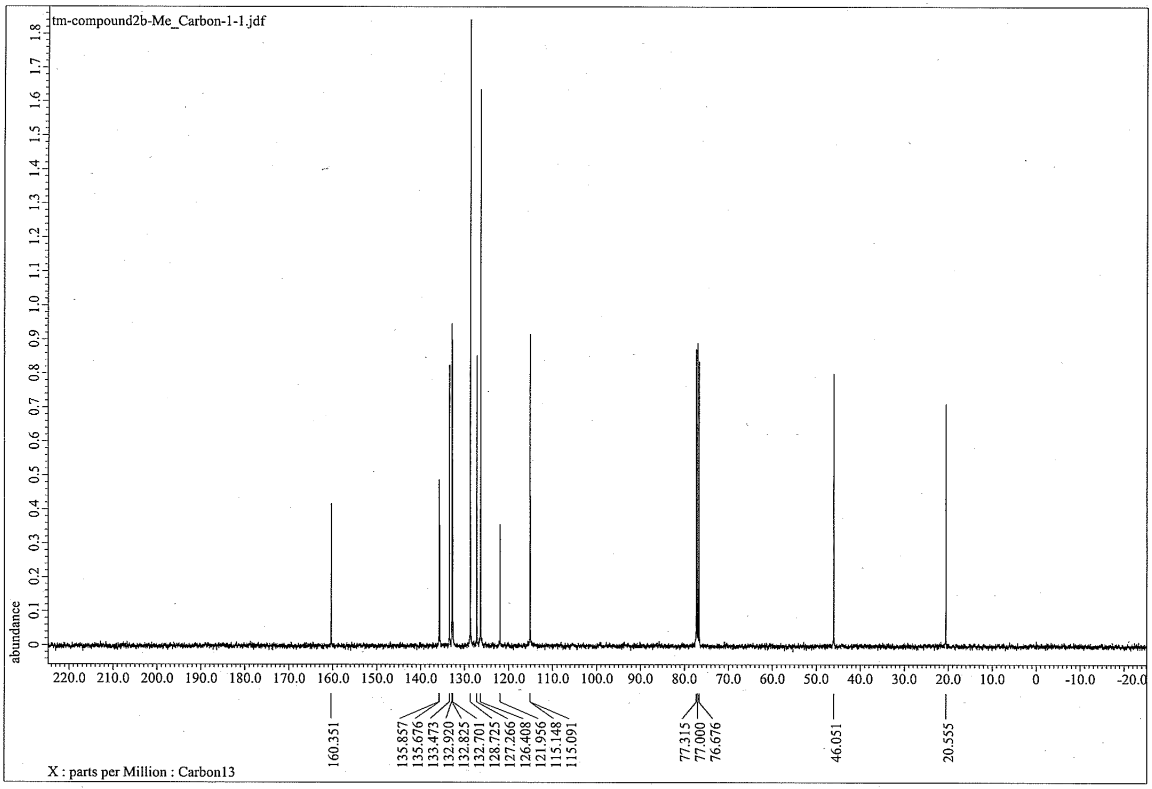
tm-compound2b-Me_Carbon-1-1.jdf
abundance
X : parts per Million : Carbon13
160.351
77.315
77.000
76.676
46.051
20.555

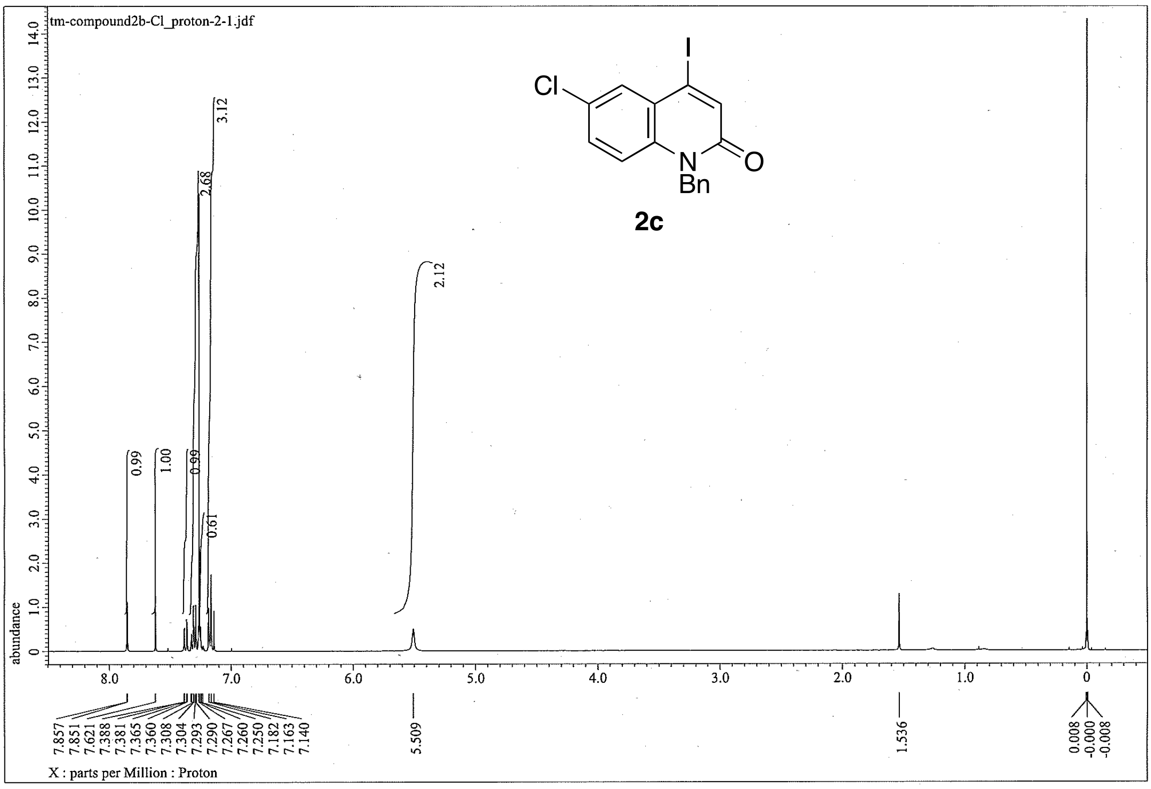

tm-compound2b-Cl_proton-2-1.jdf
2c
X : parts per Million : Proton

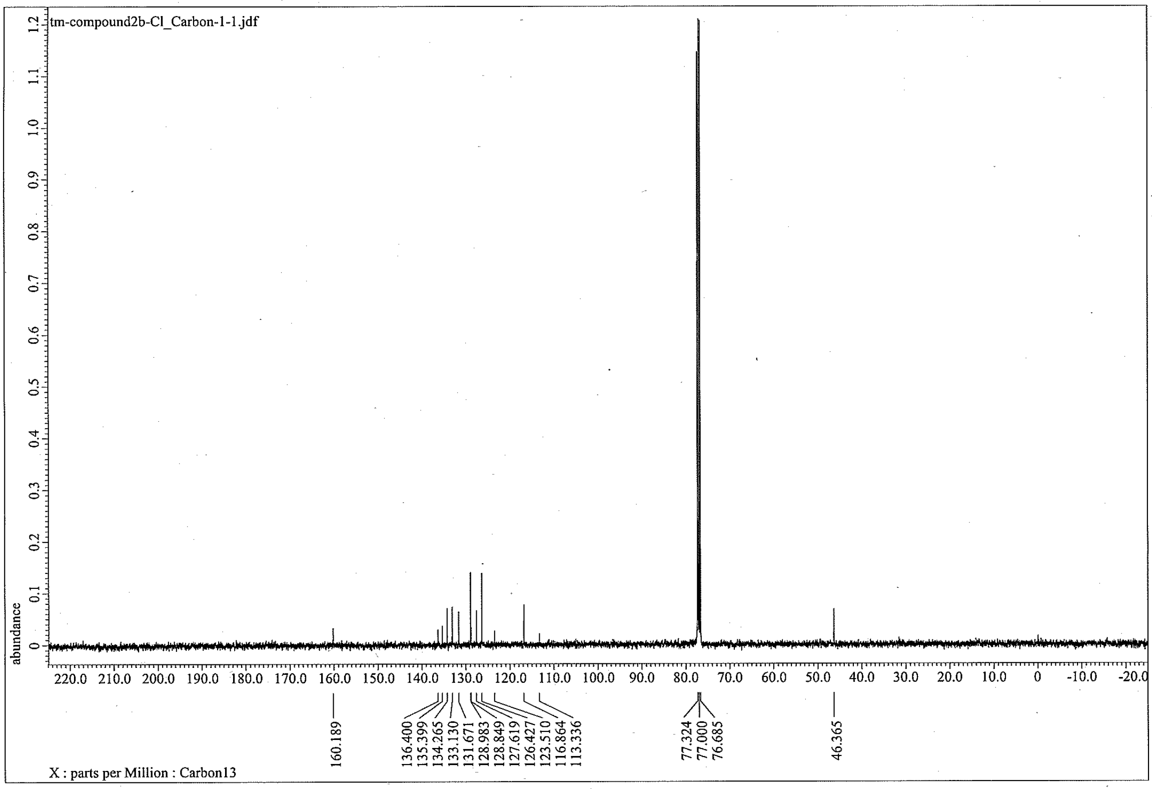

tm-compound2b-Cl_Carbon-1-1.jdf
X : parts per Million : Carbon13

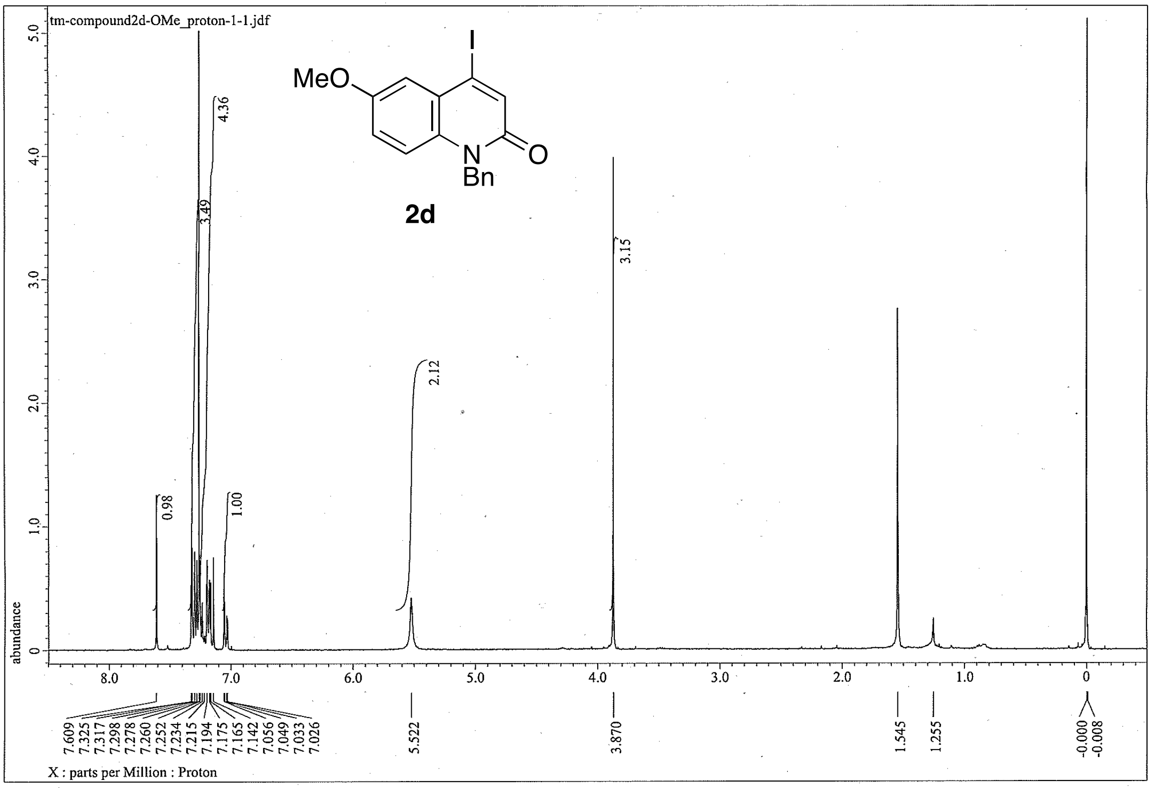
tm-compound2d-OMe_proton-1-1.jdf
MeO
I
N
O
Bn
2d
0.98
3.49
4.36
1.00
2.12
3.15
7.609
7.325
7.317
7.298
7.278
7.260
7.252
7.234
7.215
7.194
7.175
7.165
7.142
7.056
7.049
7.033
7.026
5.522
3.870
1.545
1.255
-0.000
-0.008
abundance
X : parts per Million : Proton

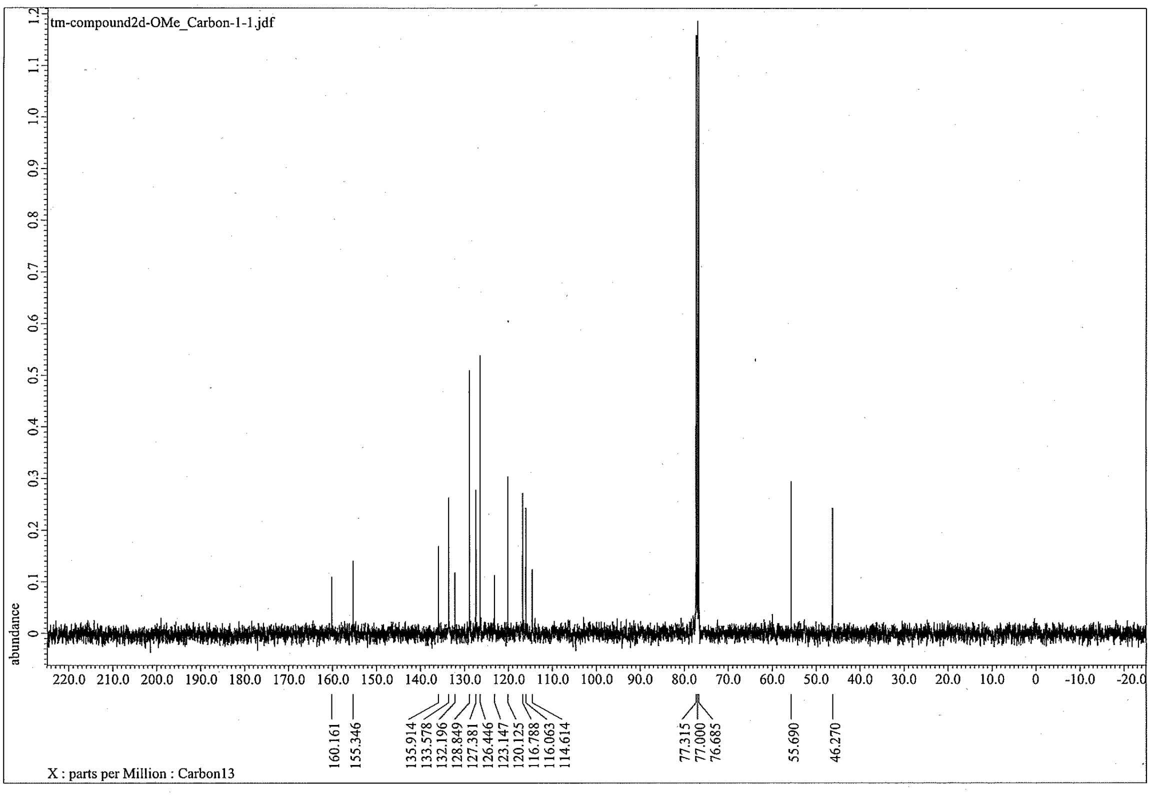
tm-compound2d-OMe_Carbon-1-1.jdf
160.161
155.346
135.914
133.578
132.196
128.849
127.381
126.446
123.147
120.125
116.788
116.063
114.614
77.315
77.000
76.685
55.690
46.270
abundance
X : parts per Million : Carbon13

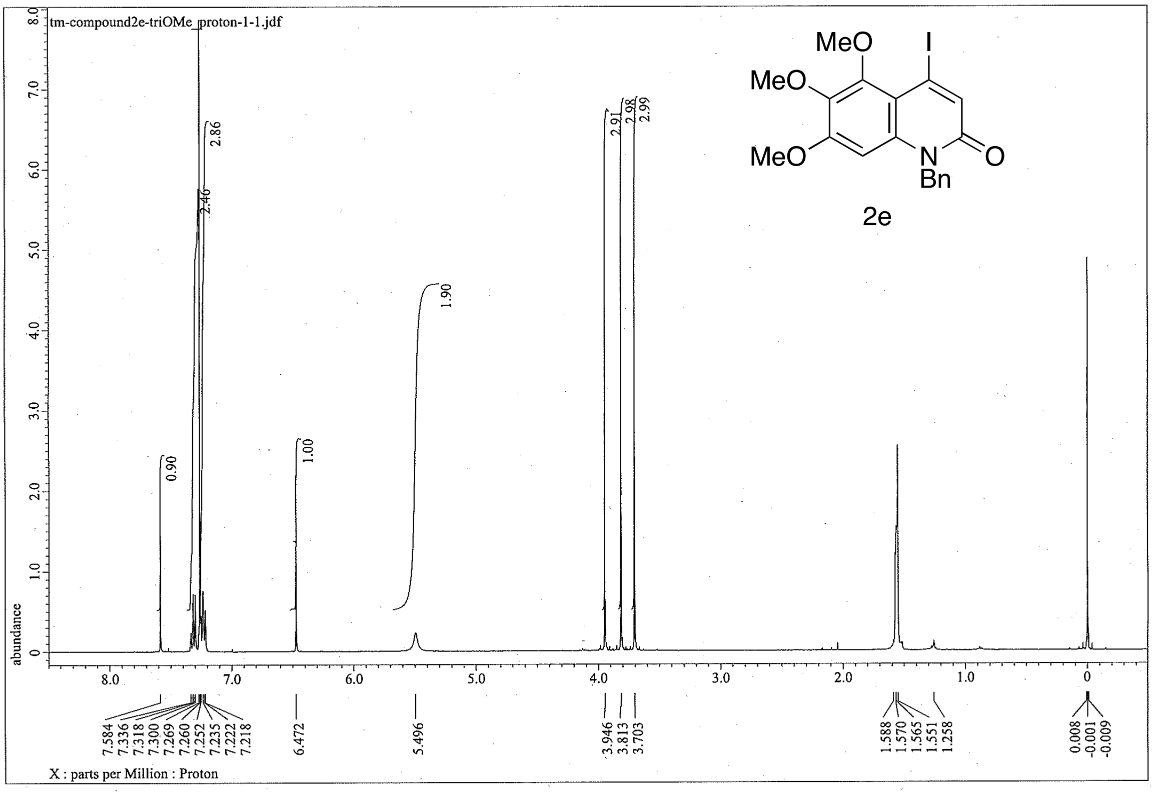

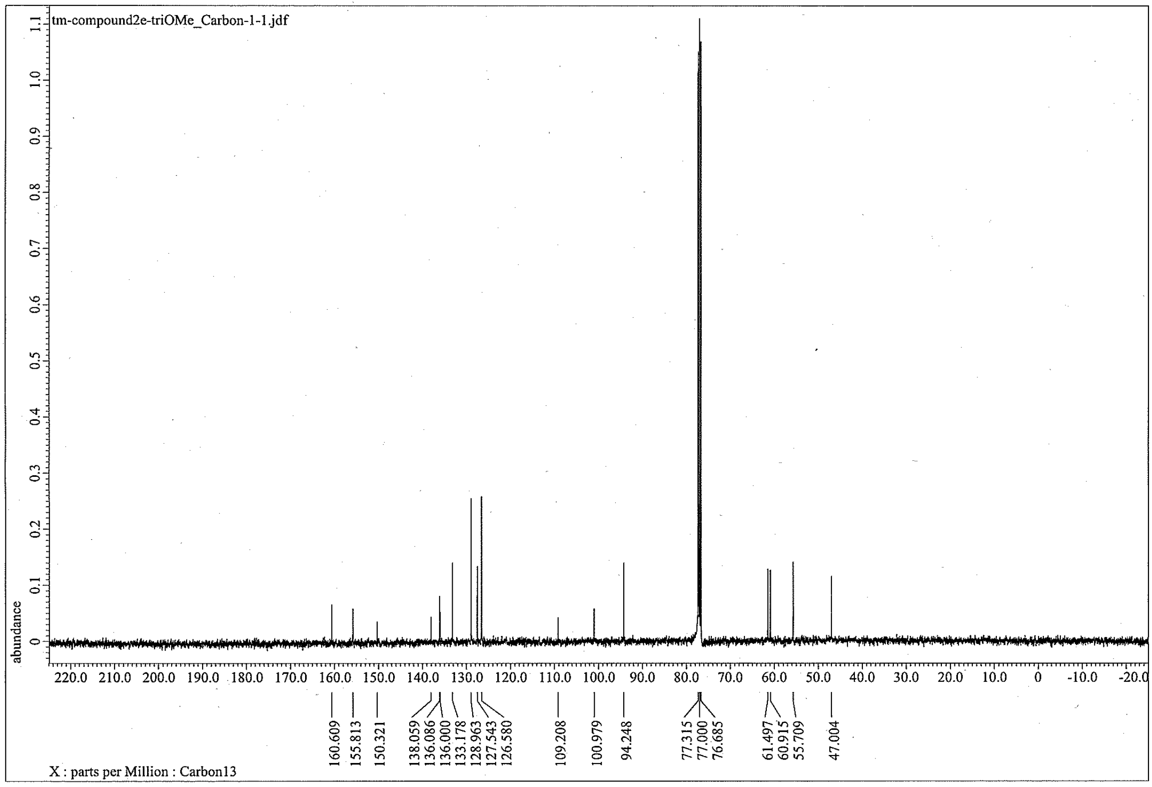

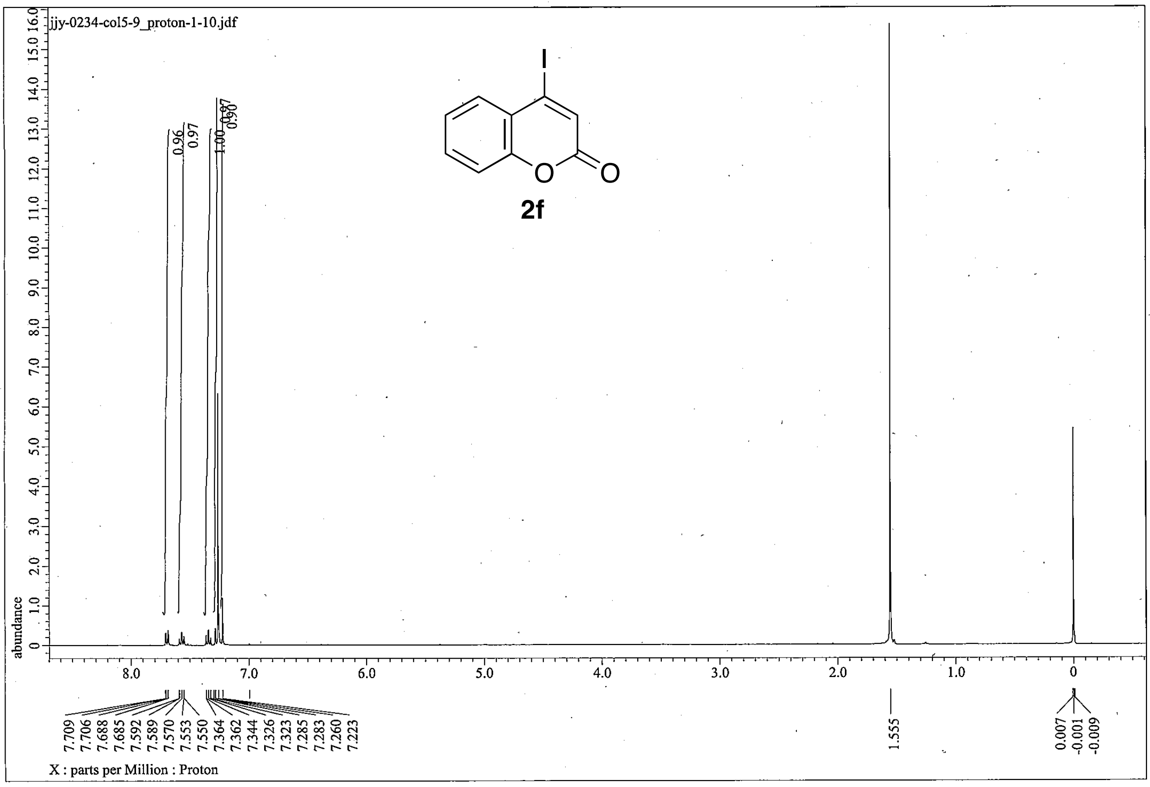
jjy-0234-col5-9_proton-1-10.jdf
2f
X : parts per Million : Proton
abundance

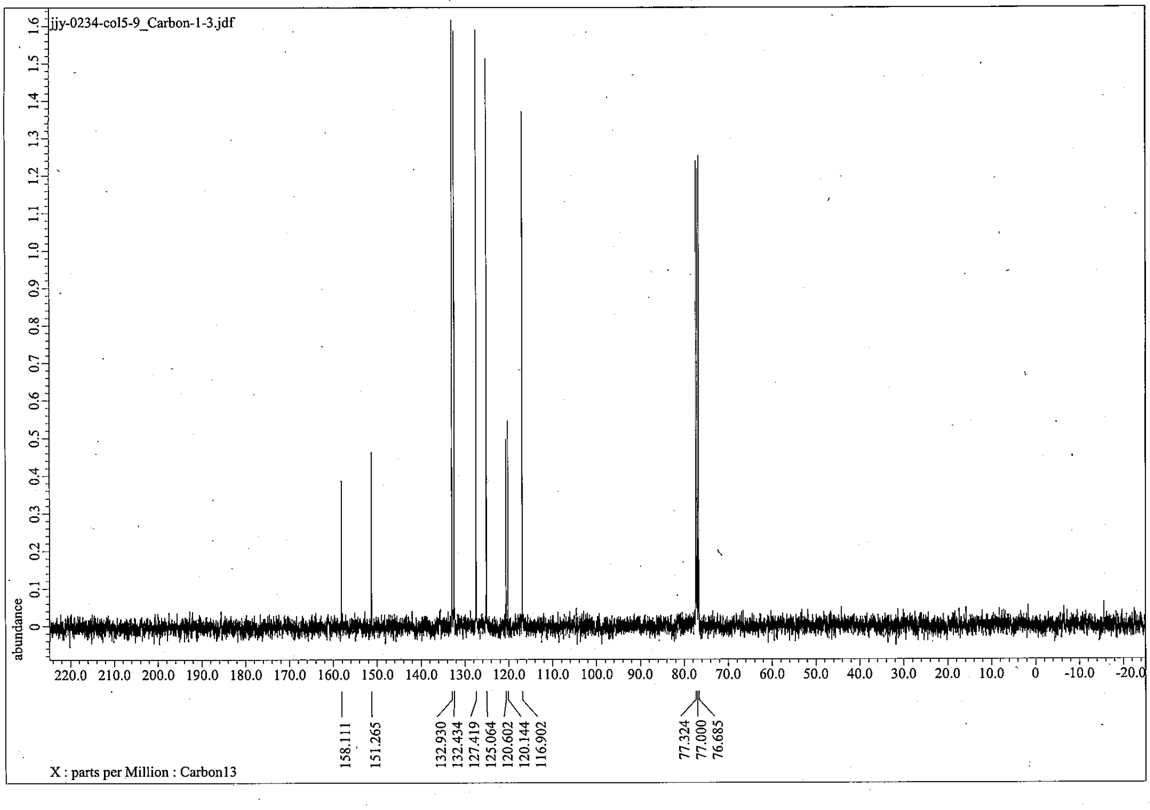
jjy-0234-col5-9_Carbon-1-3.jdf
X : parts per Million : Carbon13
abundance

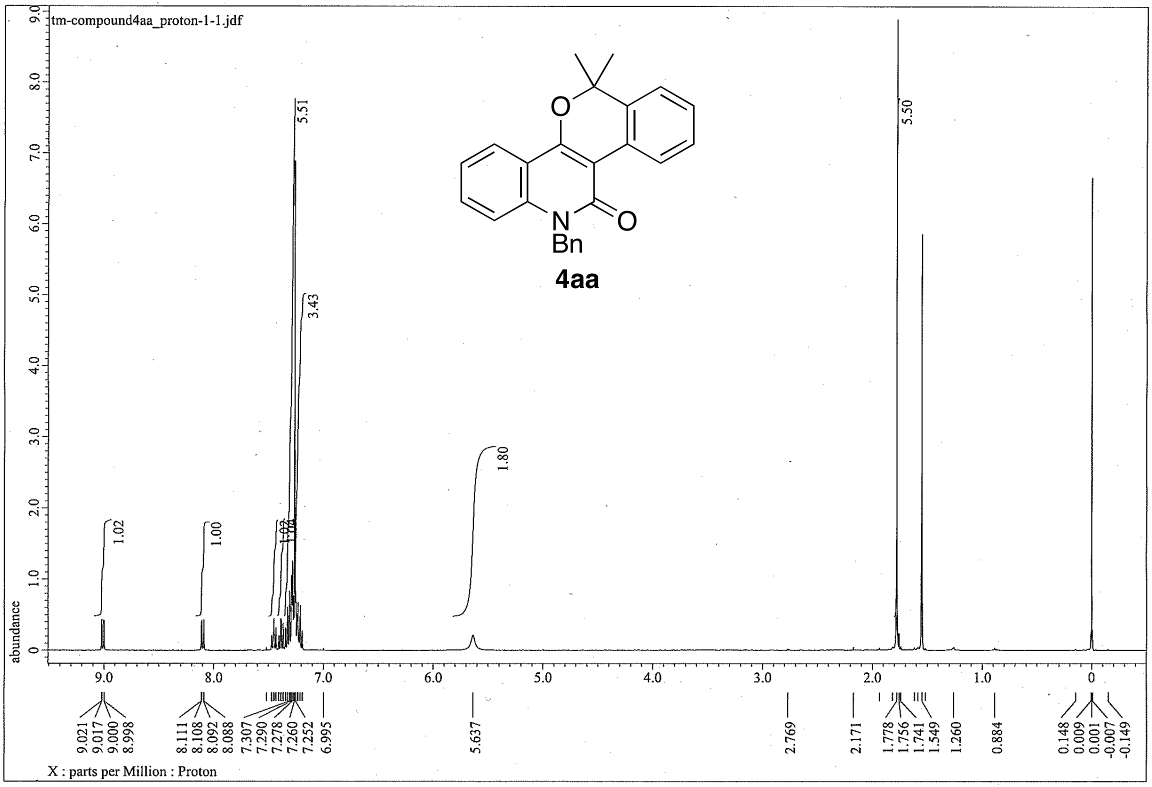
tm-compound4aa_proton-1-1.jdf
4aa
X : parts per Million : Proton
abundance

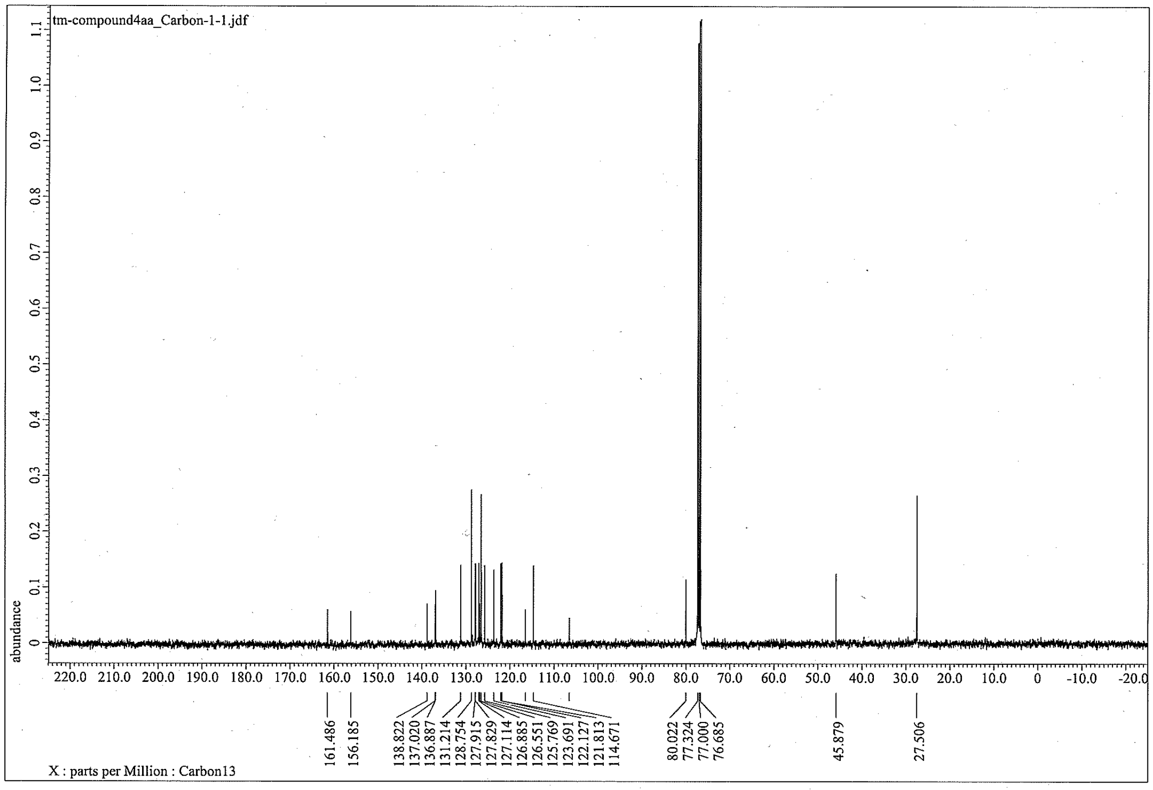
tm-compound4aa_Carbon-1-1.jdf
X : parts per Million : Carbon13
abundance

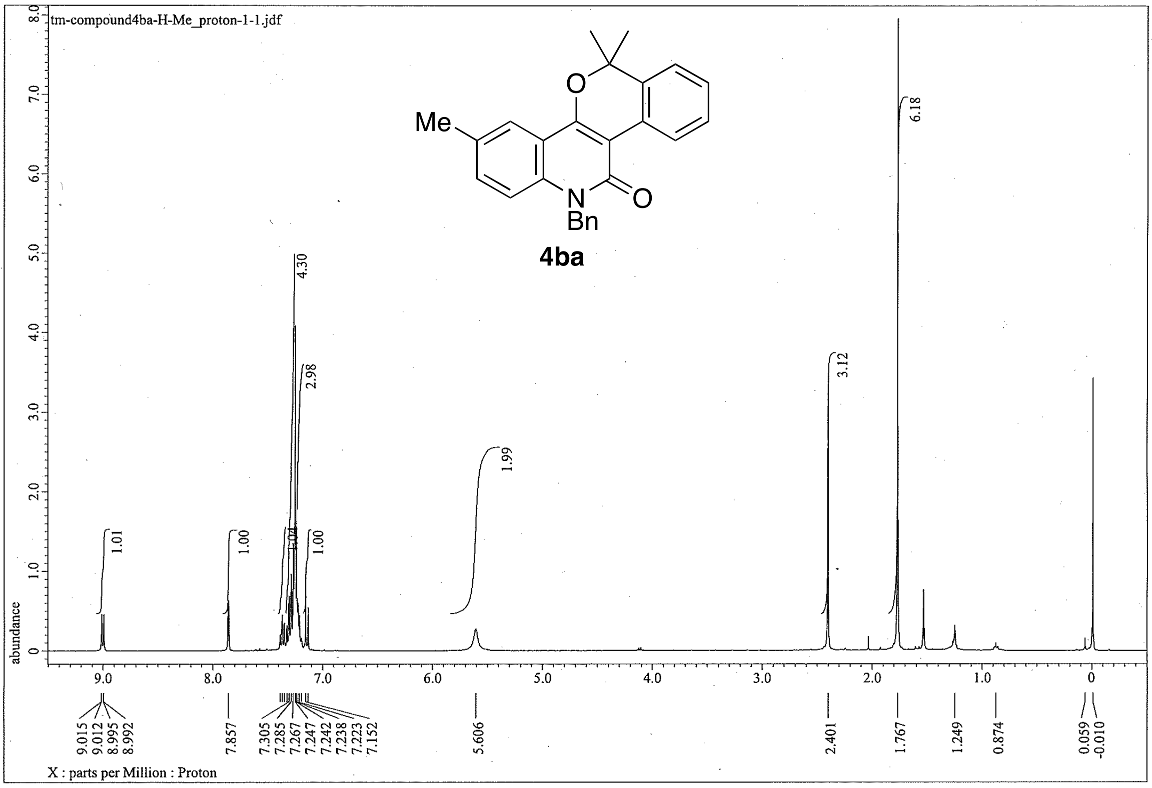
tm-compound4ba-H-Me_proton-1-1.jdf
Me
O
N
O
Bn
4ba
abundance
X : parts per Million : Proton

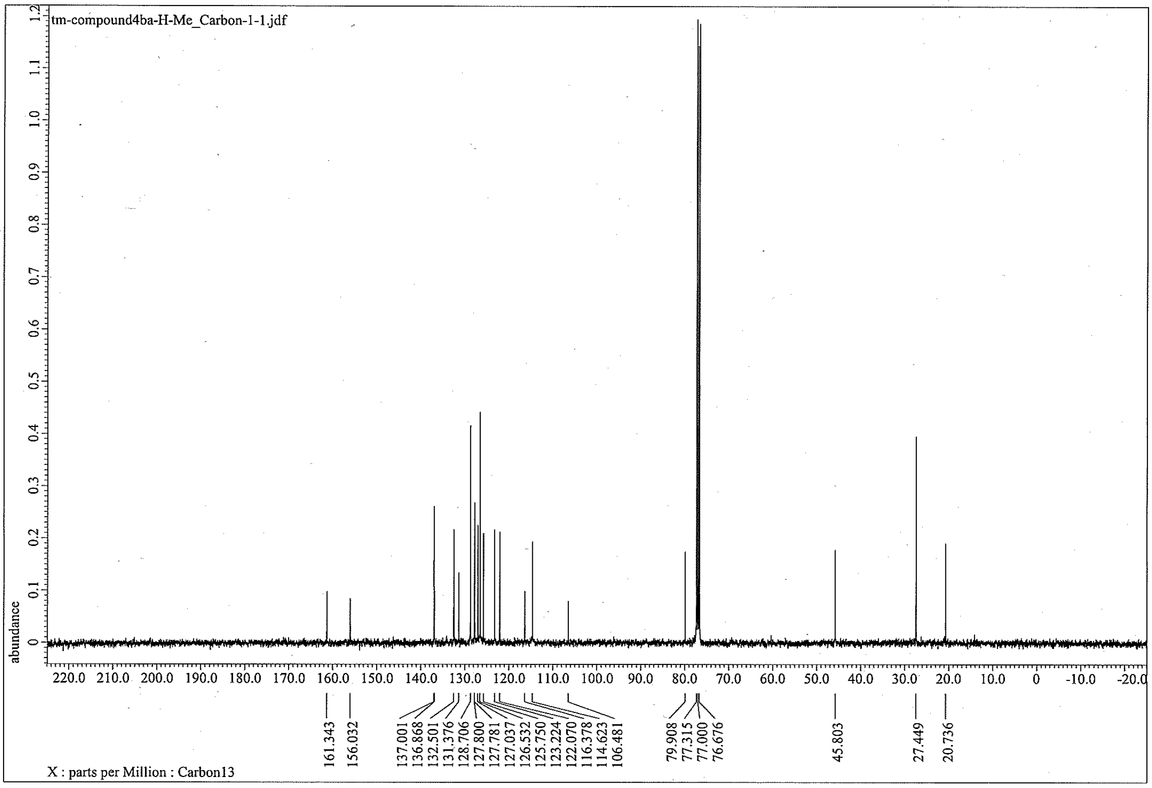
tm-compound4ba-H-Me_Carbon-1-1.jdf
abundance
X : parts per Million : Carbon13

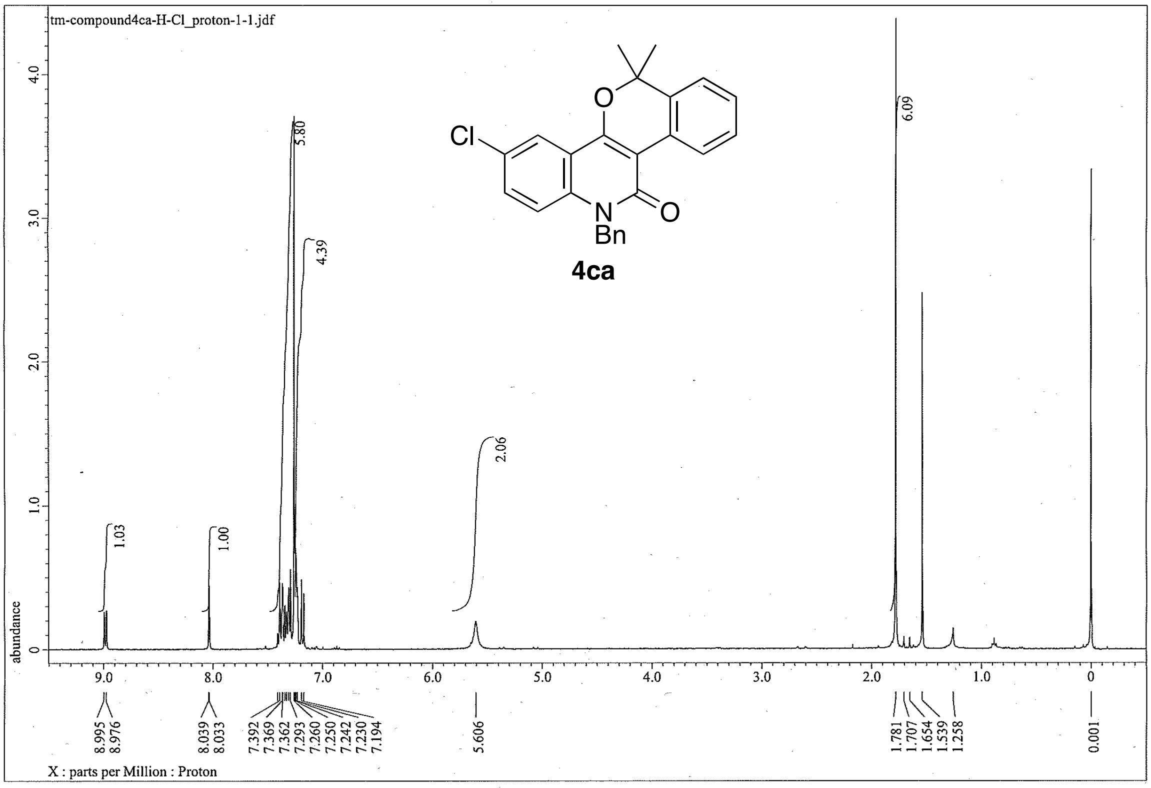
tm-compound4ca-H-Cl_proton-1-1.jdf
Cl
O
N
O
Bn
4ca
abundance
X : parts per Million : Proton
1.03
1.00
5.80
4.39
2.06
6.09
8.995
8.976
8.039
8.033
7.392
7.369
7.362
7.293
7.260
7.250
7.242
7.230
7.194
5.606
1.781
1.707
1.654
1.539
1.258
0.001

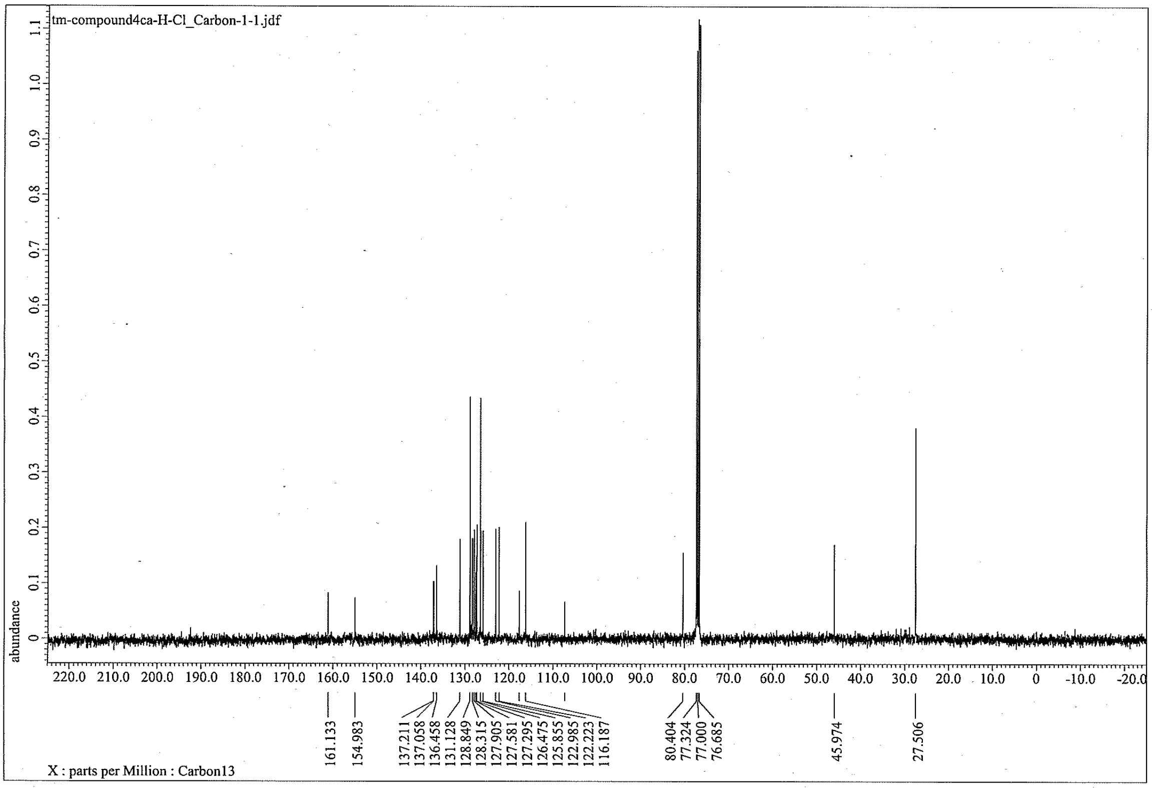
tm-compound4ca-H-Cl_Carbon-1-1.jdf
abundance
X : parts per Million : Carbon13
161.133
154.983
137.211
137.058
136.458
131.128
128.849
128.315
127.905
127.581
127.295
126.475
125.855
122.985
122.223
116.187
80.404
77.324
77.000
76.685
45.974
27.506

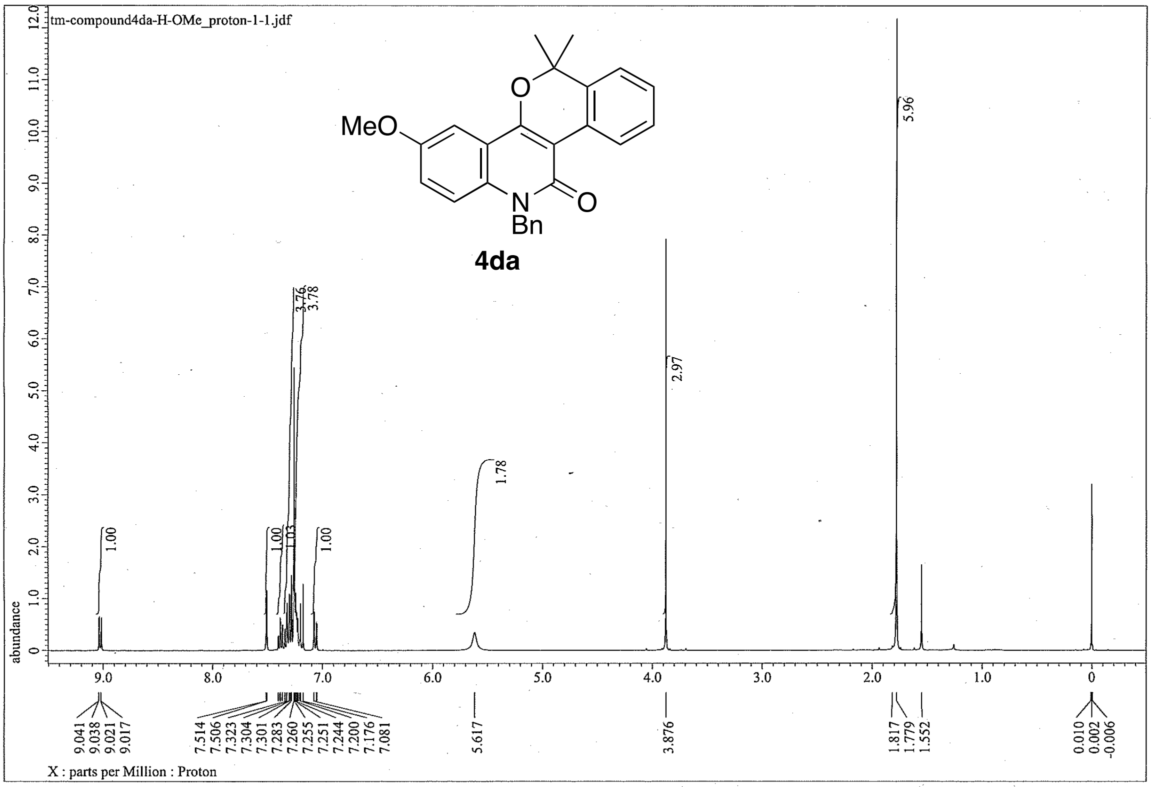
tm-compound4da-H-OMe_proton-1-1.jdf
MeO
O
N
Bn
O
4da
abundance
X : parts per Million : Proton

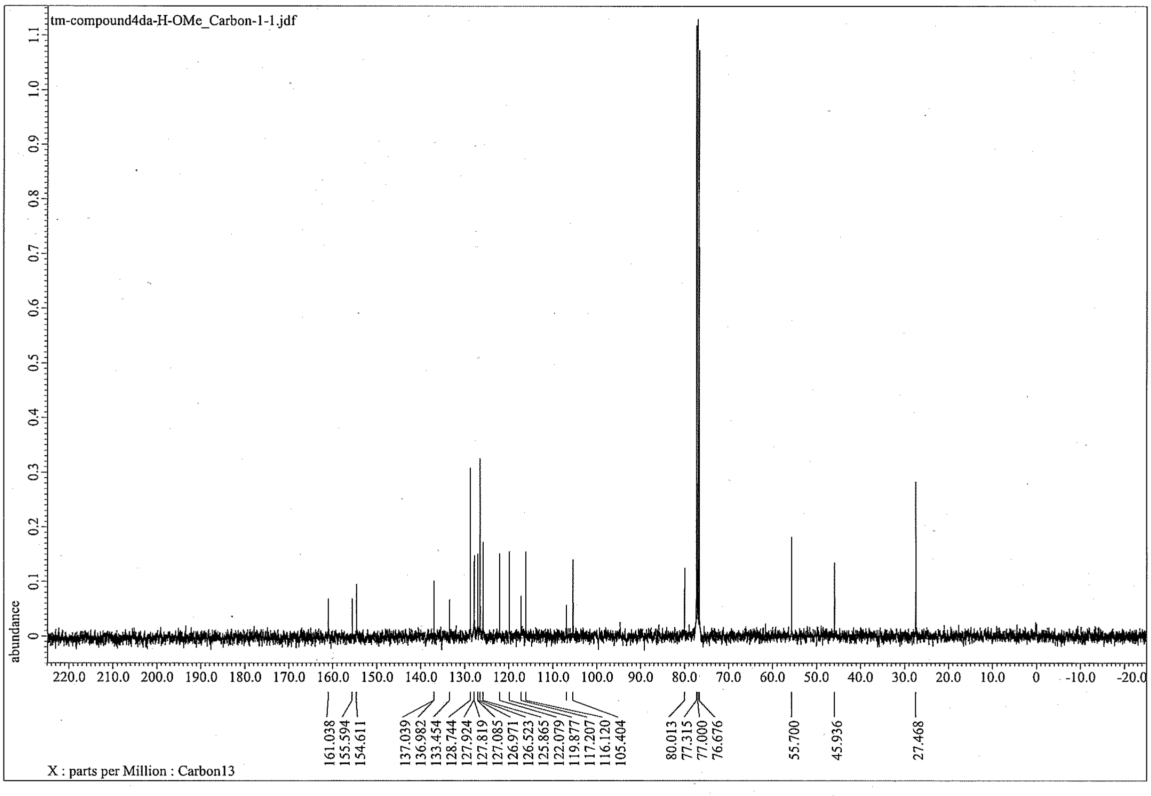
tm-compound4da-H-OMe_Carbon-1-1.jdf
abundance
X : parts per Million : Carbon13

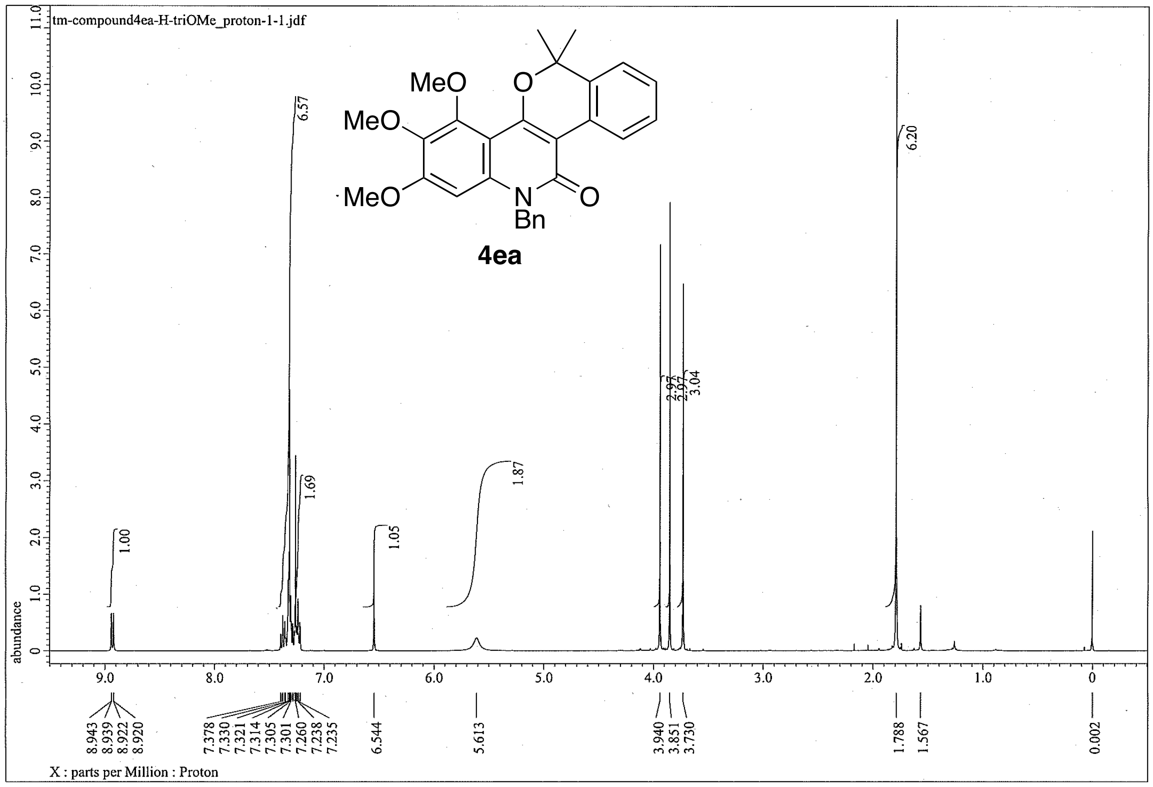
tm-compound4ea-H-triOMe_proton-1-1.jdf
MeO
MeO
MeO
O
N
O
Bn
4ea
abundance
1.00
6.57
1.69
1.05
1.87
2.97
2.97
3.04
6.20
8.943
8.939
8.922
8.920
7.378
7.330
7.321
7.314
7.305
7.301
7.260
7.238
7.235
6.544
5.613
3.940
3.851
3.730
1.788
1.567
0.002
X : parts per Million : Proton

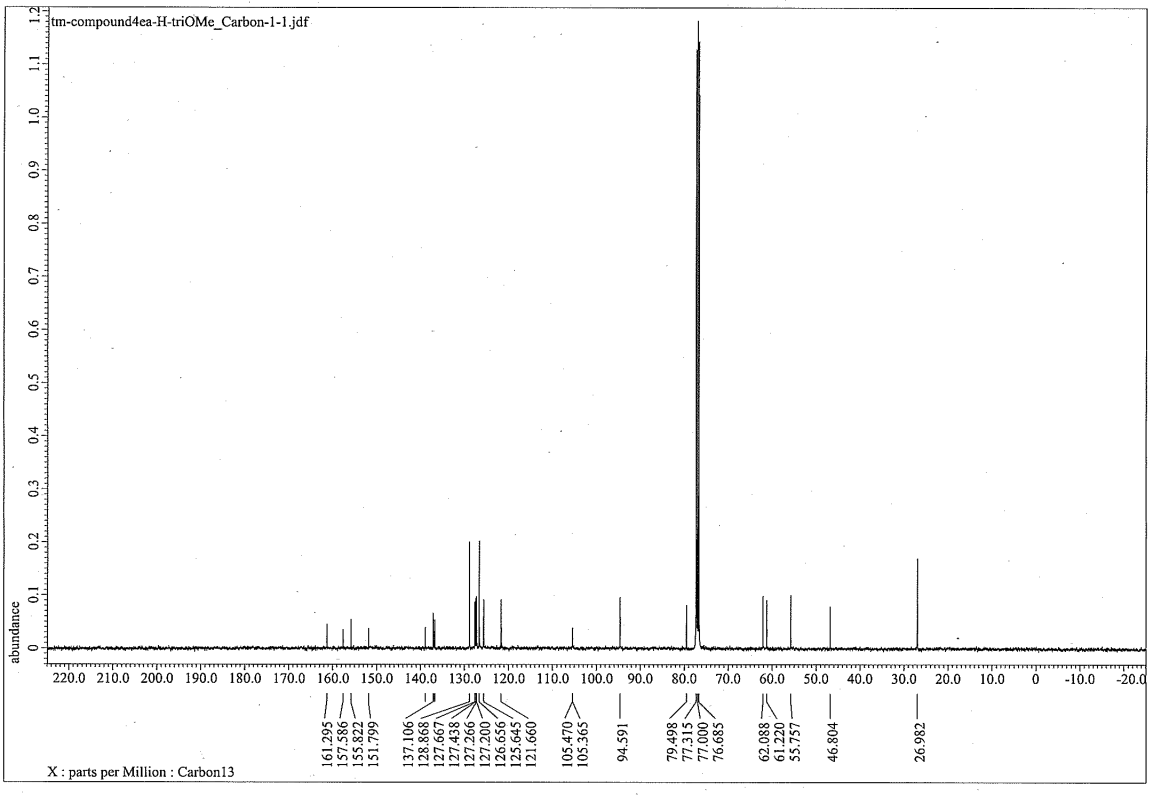
tm-compound4ea-H-triOMe_Carbon-1-1.jdf
abundance
161.295
157.586
155.822
151.799
137.106
128.868
127.667
127.438
127.266
127.200
126.656
125.645
121.660
105.470
105.365
94.591
79.498
77.315
77.000
76.685
62.088
61.220
55.757
46.804
26.982
X : parts per Million : Carbon13

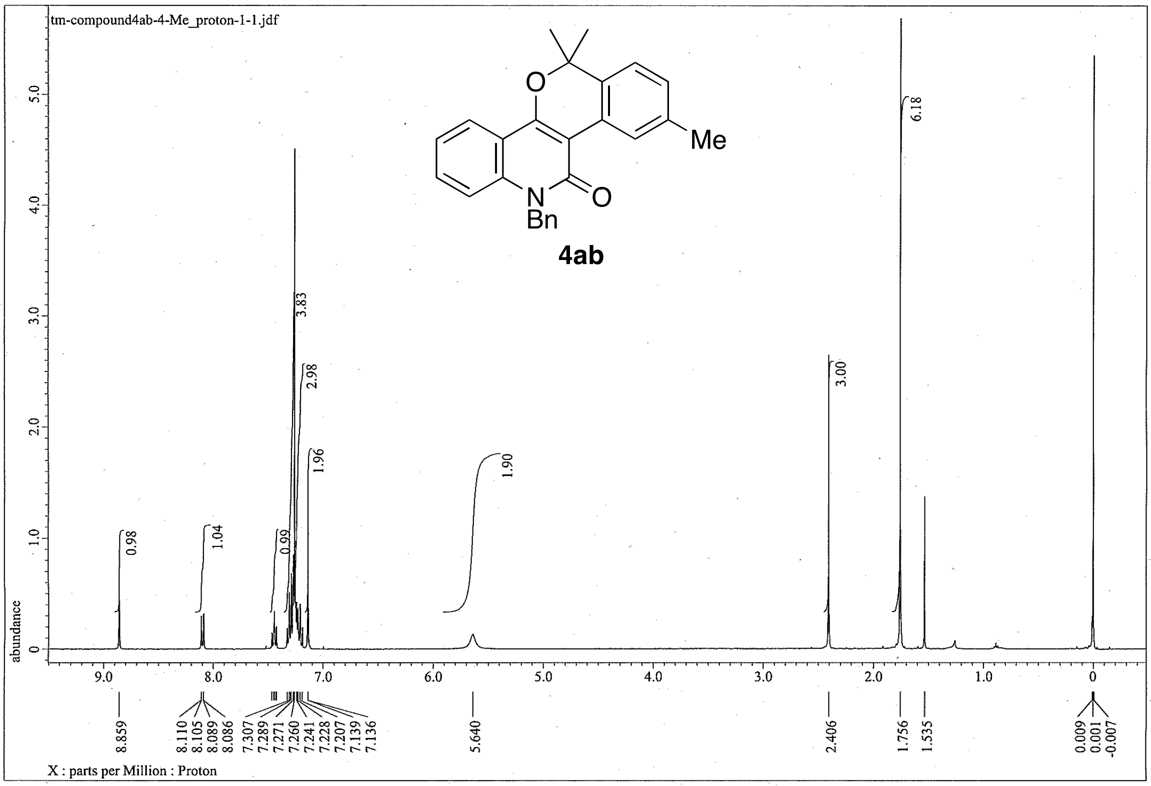
tm-compound4ab-4-Me_proton-1-1.jdf
O
Me
N
O
Bn
4ab
0.98
1.04
0.99
3.83
2.98
1.96
1.90
3.00
6.18
abundance
X : parts per Million : Proton
8.859
8.110
8.105
8.089
8.086
7.307
7.289
7.271
7.260
7.241
7.228
7.207
7.139
7.136
5.640
2.406
1.756
1.535
0.009
0.001
-0.007

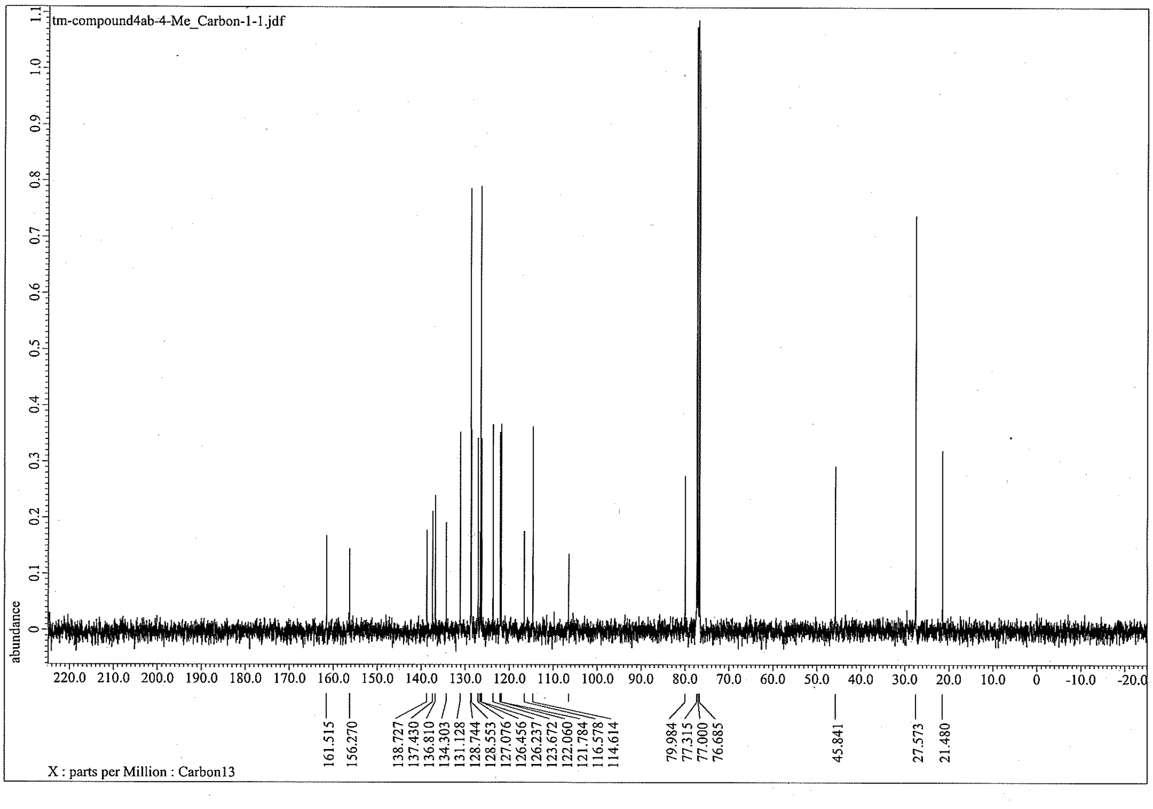
tm-compound4ab-4-Me_Carbon-1-1.jdf
abundance
X : parts per Million : Carbon13
161.515
156.270
138.727
137.430
136.810
134.303
131.128
128.744
128.553
127.076
126.456
126.237
123.672
122.060
121.784
116.578
114.614
79.984
77.315
77.000
76.685
45.841
27.573
21.480

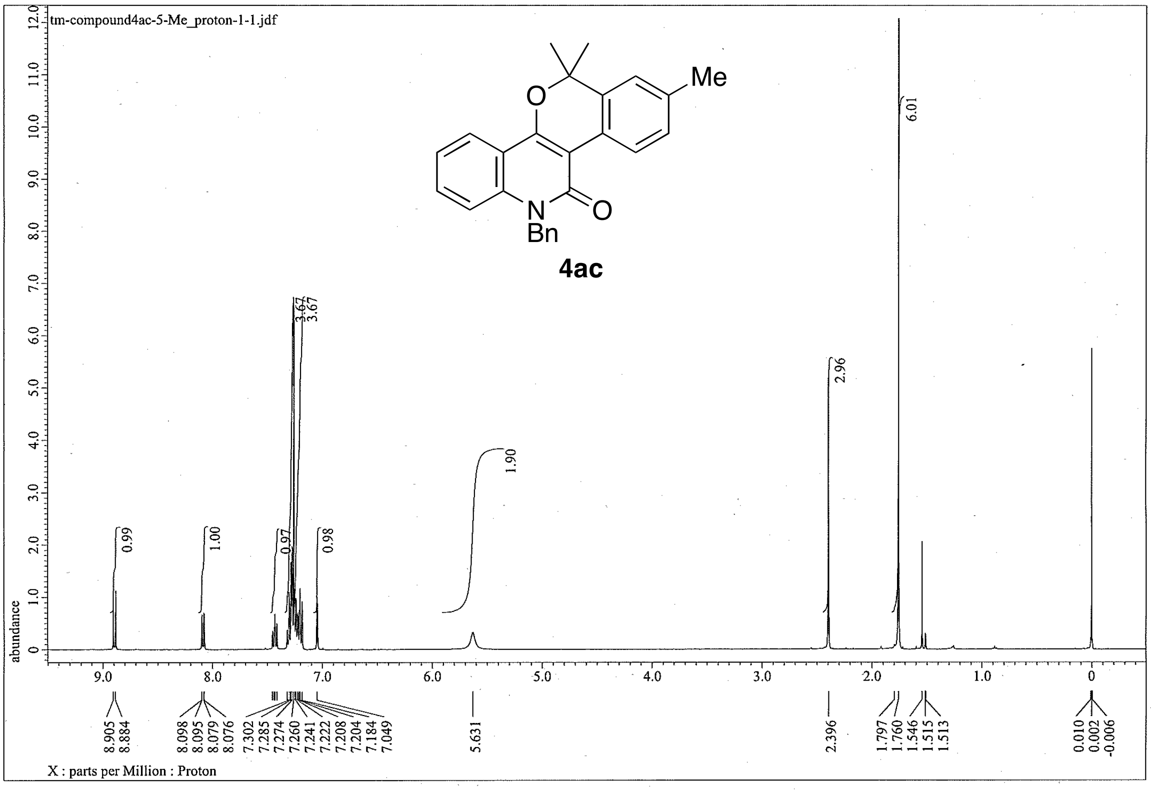
tm-compound4ac-5-Me_proton-1-1.jdf
Me
O
N
O
Bn
4ac
X : parts per Million : Proton
abundance

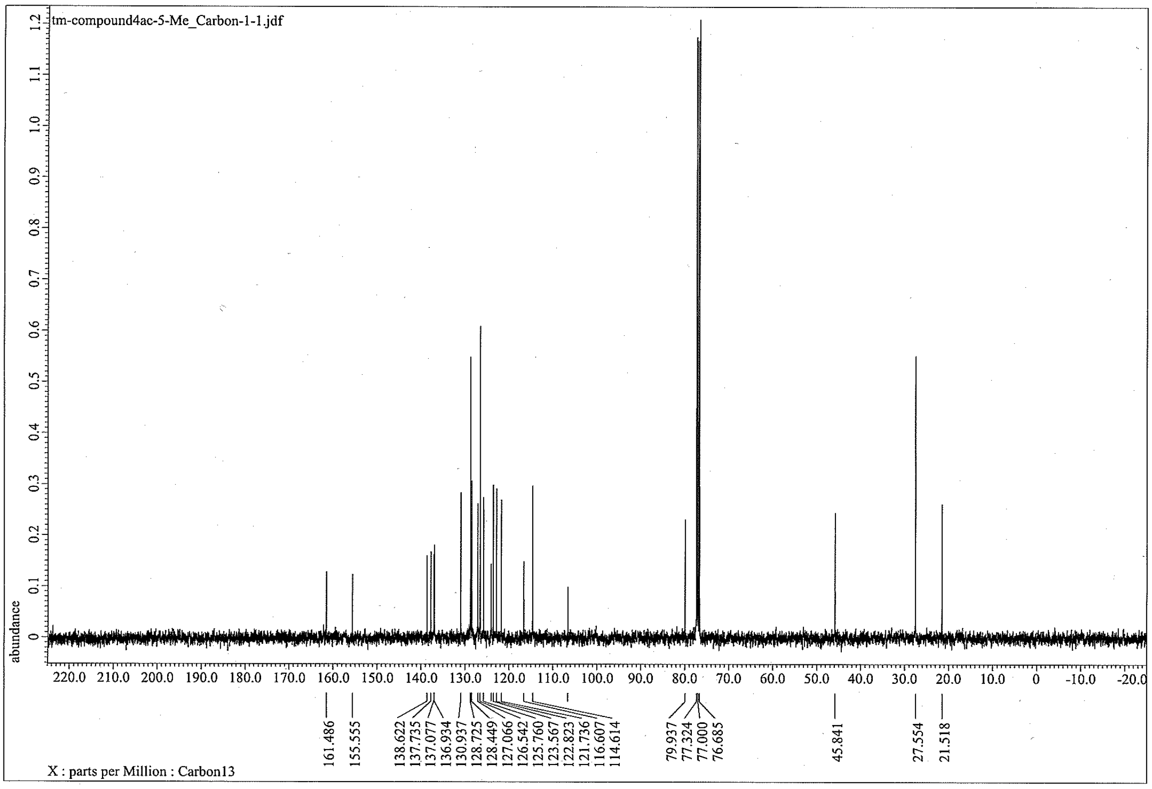
tm-compound4ac-5-Me_Carbon-1-1.jdf
X : parts per Million : Carbon13
abundance

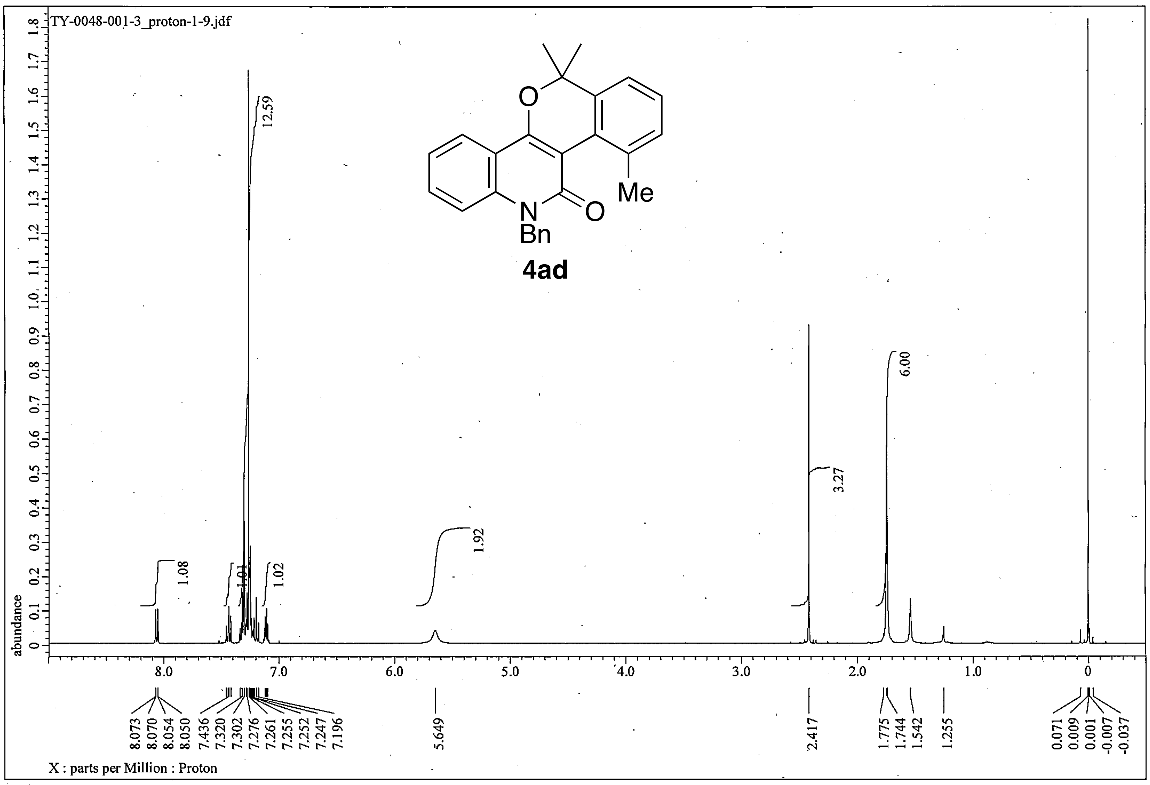
TY-0048-001-3_proton-1-9.jdf
4ad
Me
Bn
X : parts per Million : Proton
abundance

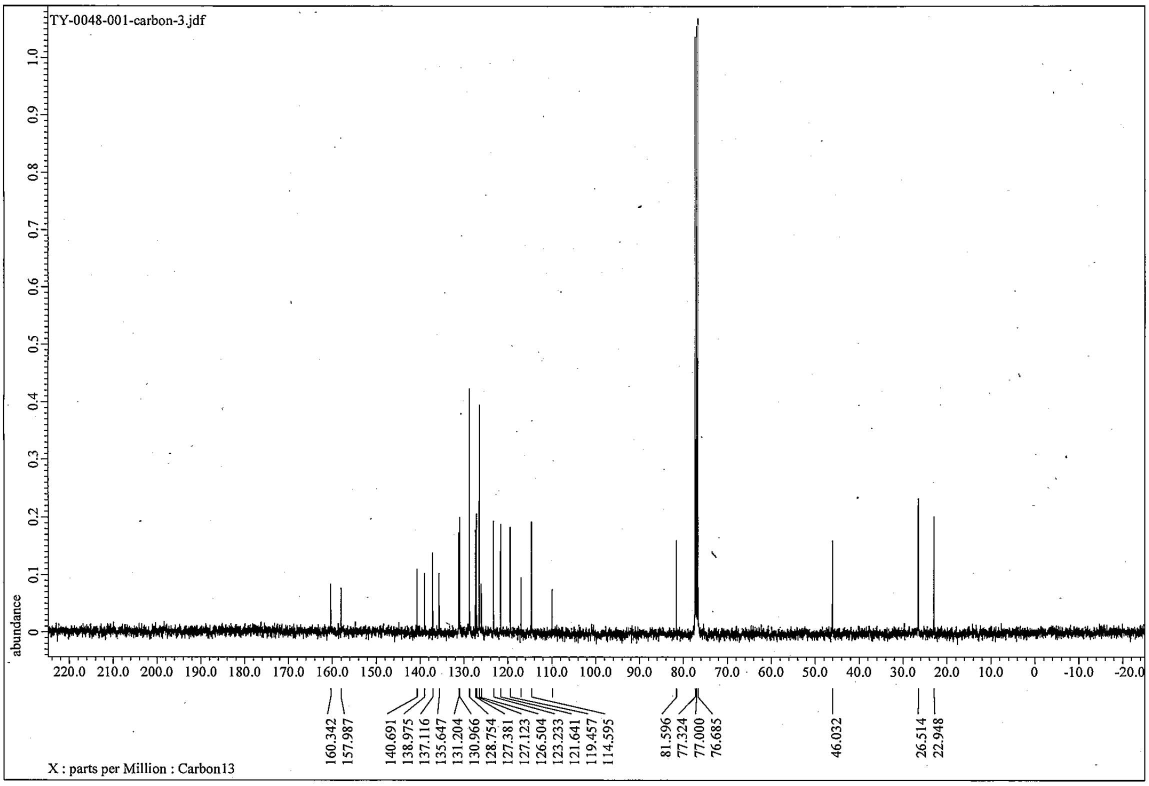
TY-0048-001-carbon-3.jdf
X : parts per Million : Carbon13
abundance

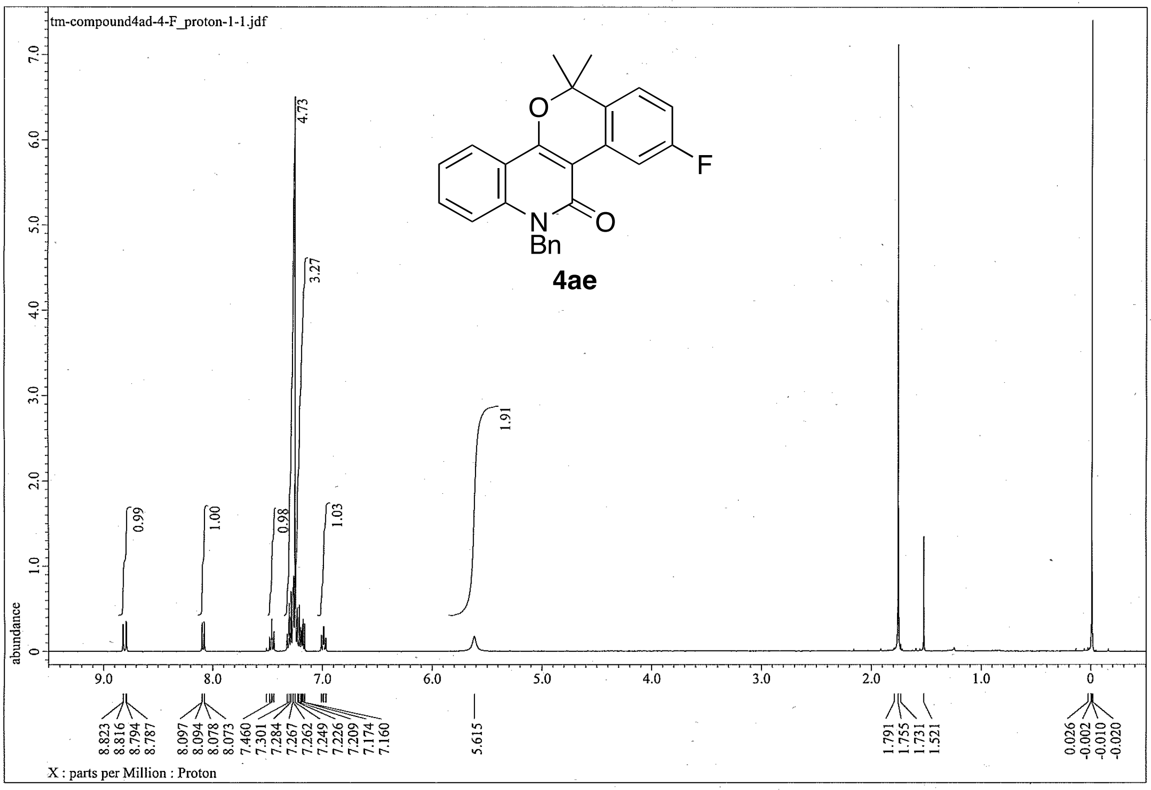
tm-compound4ad-4-F_proton-1-1.jdf
O
F
N
O
Bn
4ae
0.99
1.00
0.98
4.73
3.27
1.03
1.91
abundance
9.0
8.0
7.0
6.0
5.0
4.0
3.0
2.0
1.0
0
8.823
8.816
8.794
8.787
8.097
8.094
8.078
8.073
7.460
7.301
7.284
7.267
7.262
7.249
7.226
7.209
7.174
7.160
5.615
1.791
1.755
1.731
1.521
0.026
-0.002
-0.010
-0.020
X : parts per Million : Proton

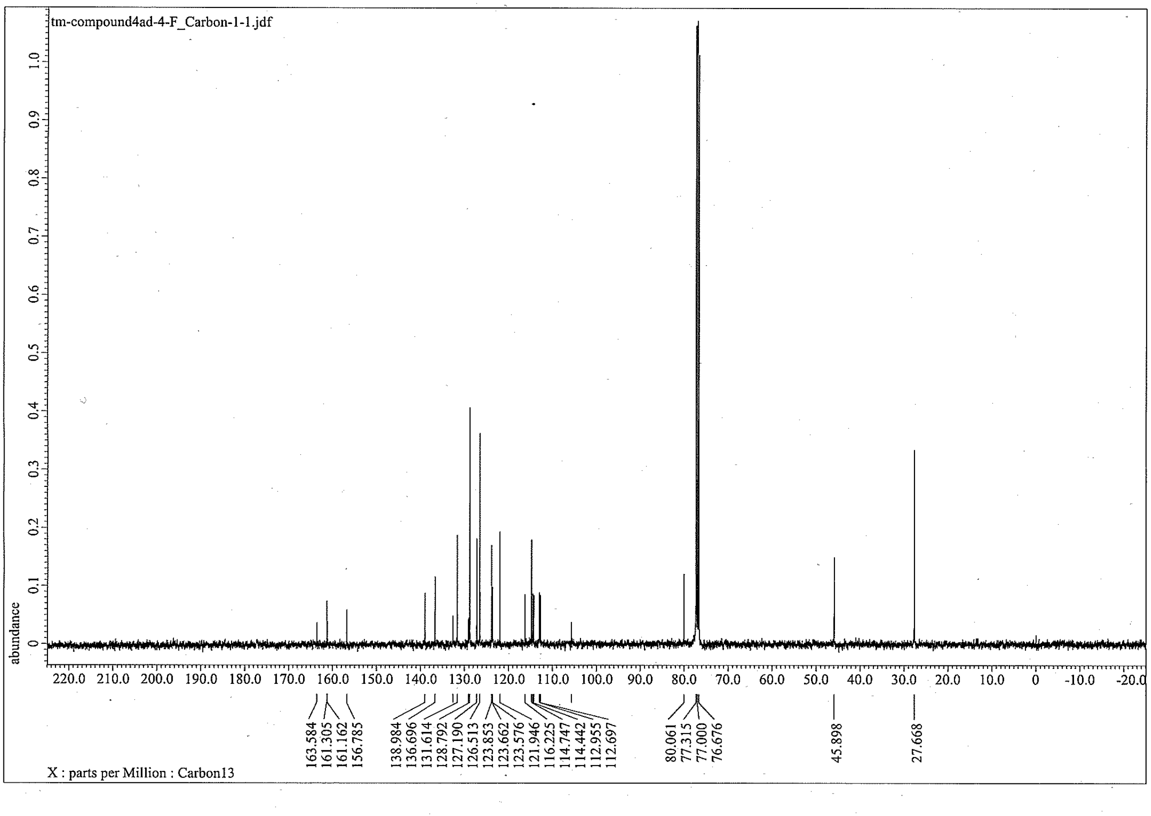
tm-compound4ad-4-F_Carbon-1-1.jdf
abundance
220.0 210.0 200.0 190.0 180.0 170.0 160.0 150.0 140.0 130.0 120.0 110.0 100.0 90.0 80.0 70.0 60.0 50.0 40.0 30.0 20.0 10.0 0 -10.0 -20.0
163.584
161.305
161.162
156.785
138.984
136.696
131.614
128.792
127.190
126.513
123.853
123.662
123.576
121.946
116.225
114.747
114.442
112.955
112.697
80.061
77.315
77.000
76.676
45.898
27.668
X : parts per Million : Carbon13

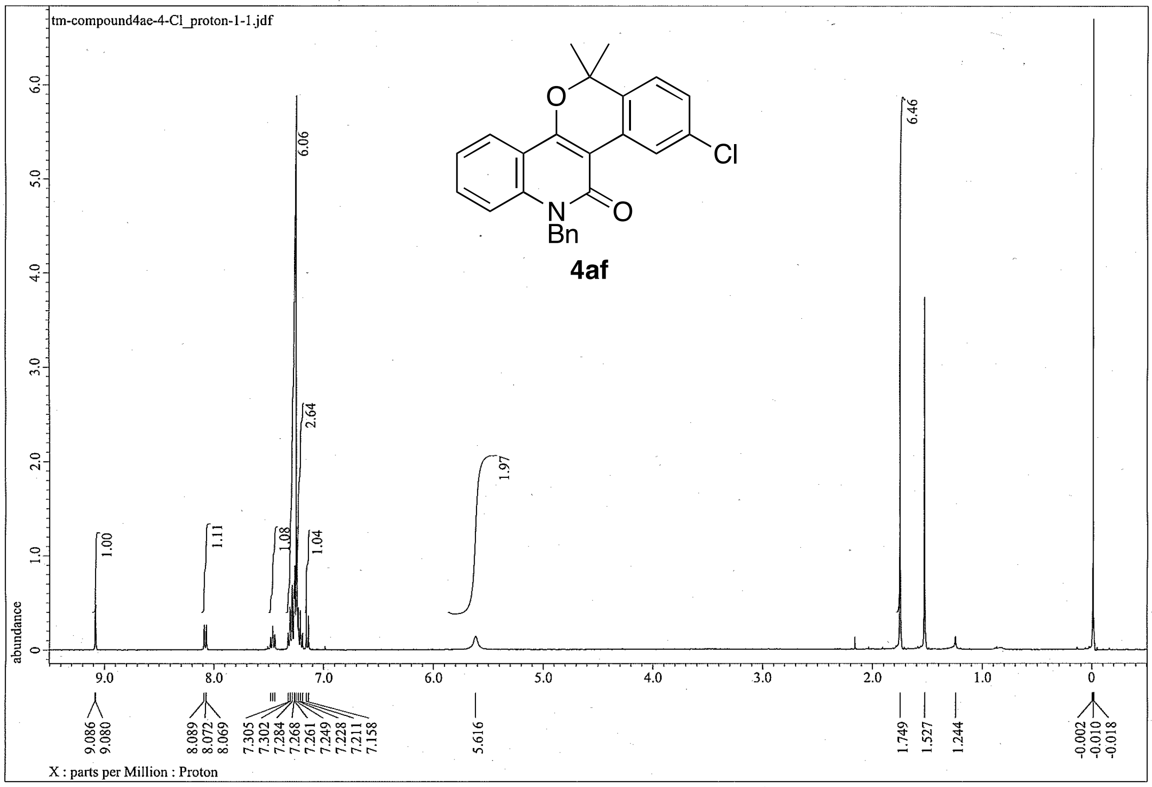
tm-compound4ae-4-Cl_proton-1-1.jdf
Cl
O
N
Bn
4af
abundance
X : parts per Million : Proton

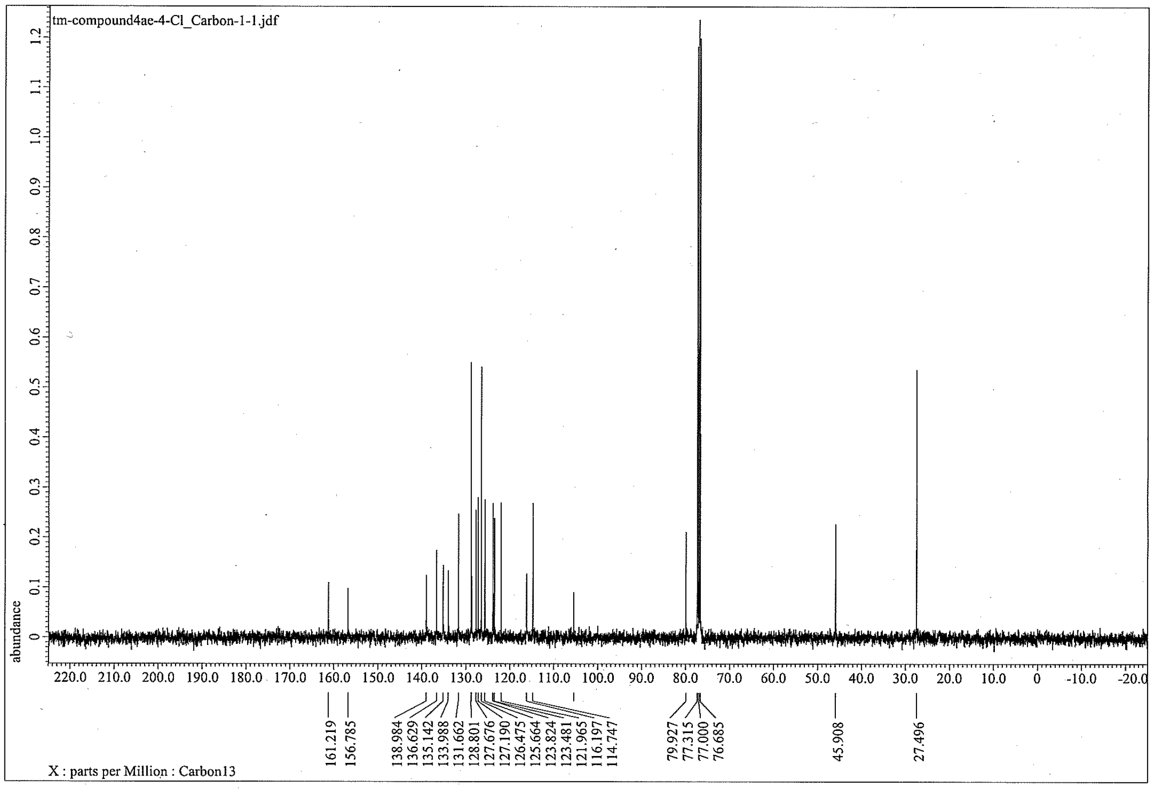
tm-compound4ae-4-Cl_Carbon-1-1.jdf
abundance
X : parts per Million : Carbon13

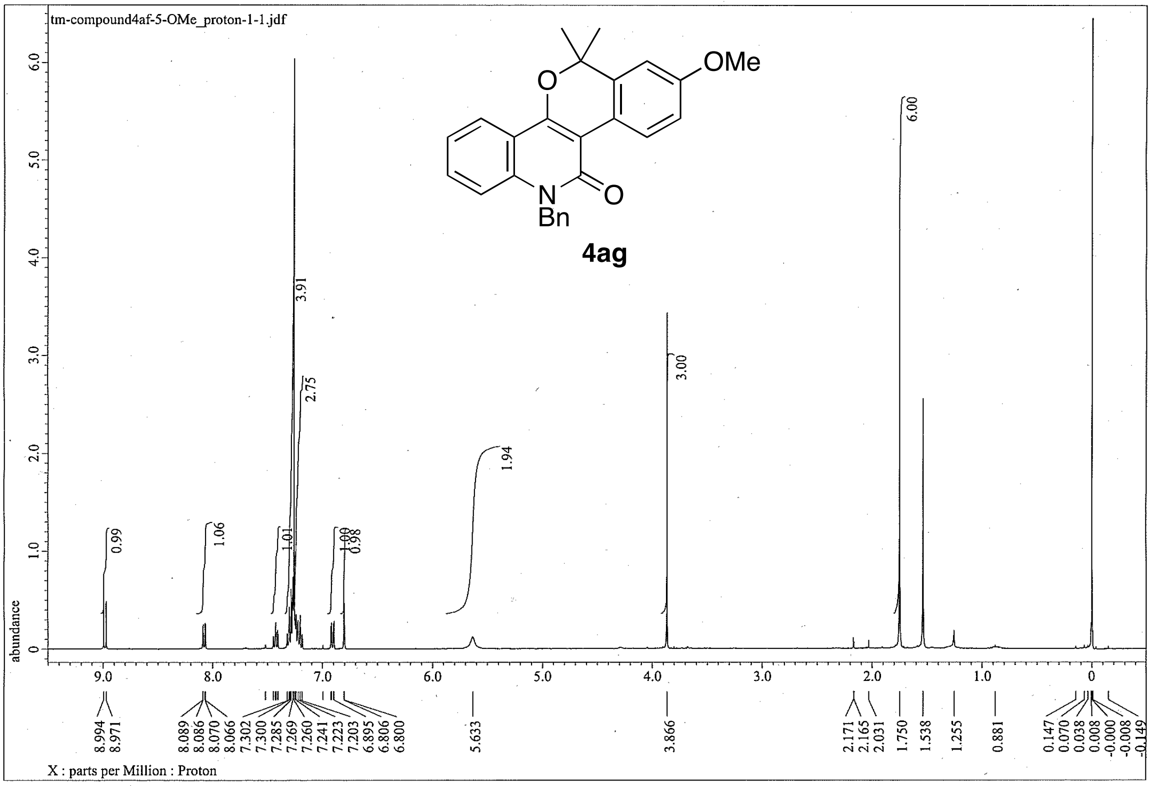
tm-compound4af-5-OMe_proton-1-1.jdf
OMe
O
N
O
Bn
4ag
0.99
1.06
1.01
3.91
2.75
1.00
0.98
1.94
3.00
6.00
abundance
8.994
8.971
8.089
8.086
8.070
8.066
7.302
7.300
7.285
7.269
7.260
7.241
7.223
7.203
6.895
6.806
6.800
5.633
3.866
2.171
2.165
2.031
1.750
1.538
1.255
0.881
0.147
0.070
0.038
0.008
-0.000
-0.008
-0.149
X : parts per Million : Proton

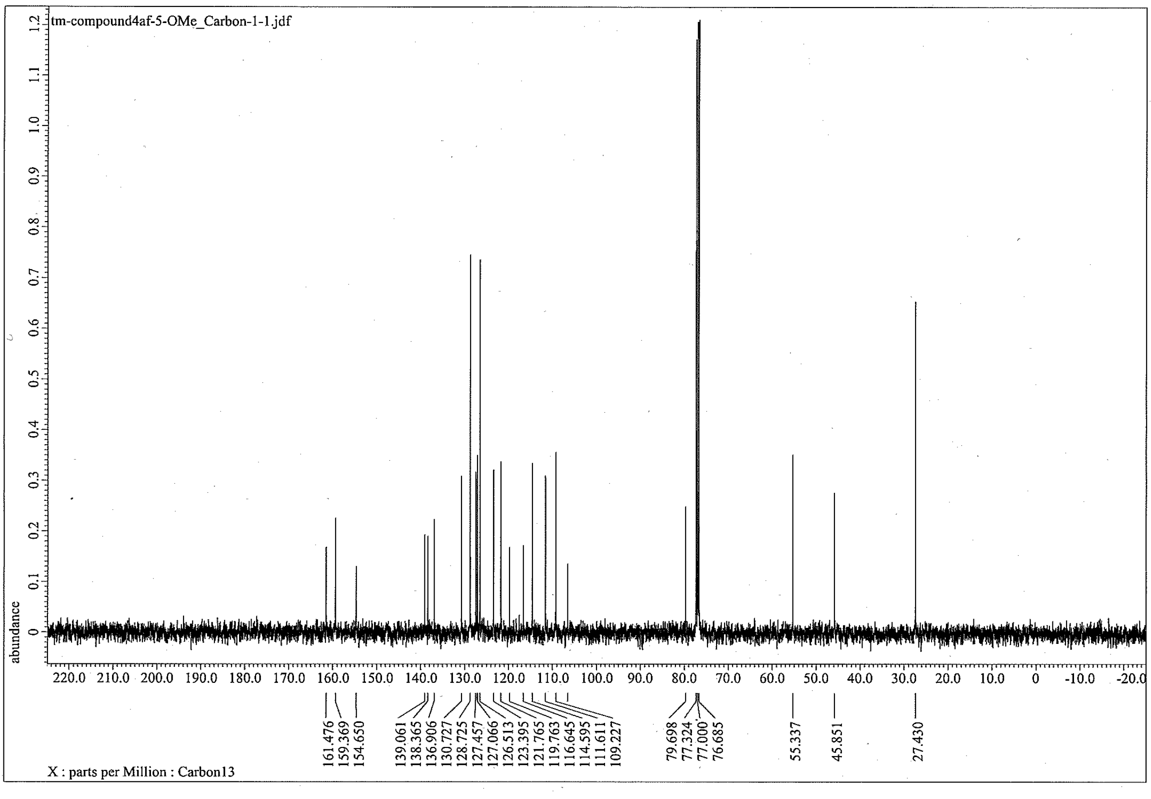
tm-compound4af-5-OMe_Carbon-1-1.jdf
abundance
161.476
159.369
154.650
139.061
138.365
136.906
130.727
128.725
127.457
127.066
126.513
123.395
121.765
119.763
116.645
114.595
111.611
109.227
79.698
77.324
77.000
76.685
55.337
45.851
27.430
X : parts per Million : Carbon13

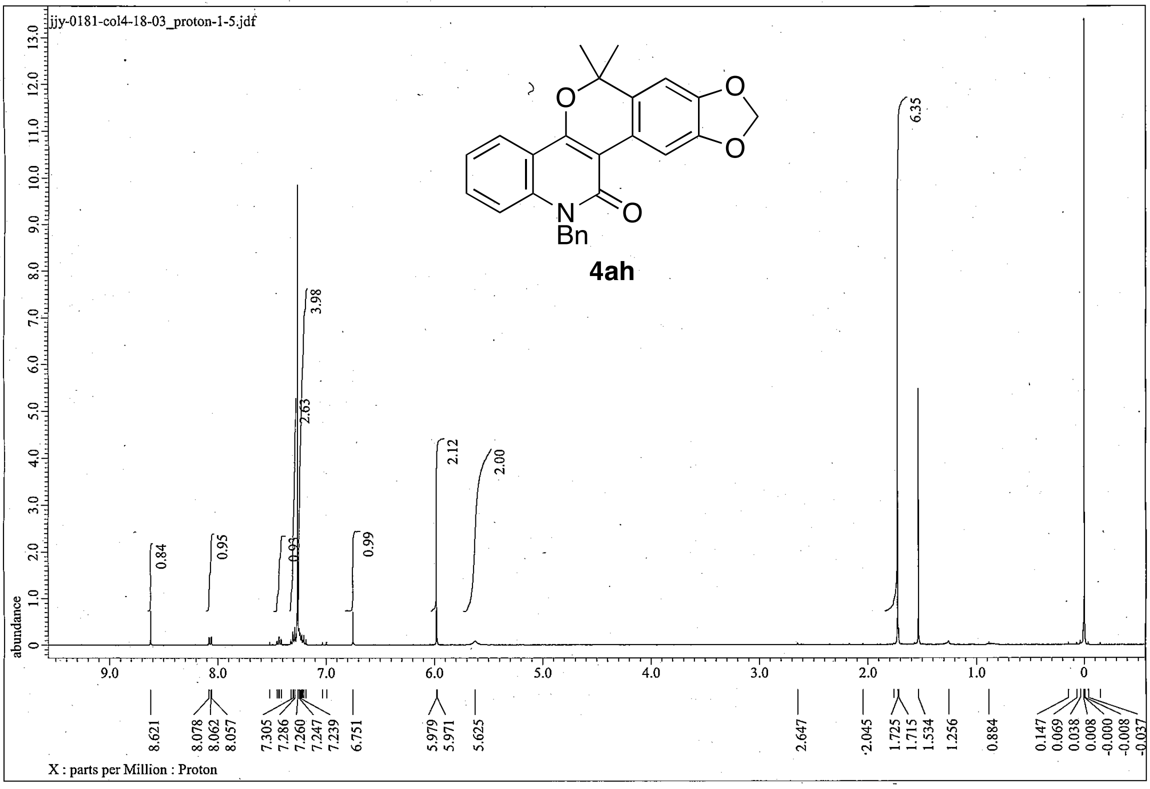
jjy-0181-col4-18-03_proton-1-5.jdf
4ah
X : parts per Million : Proton
abundance

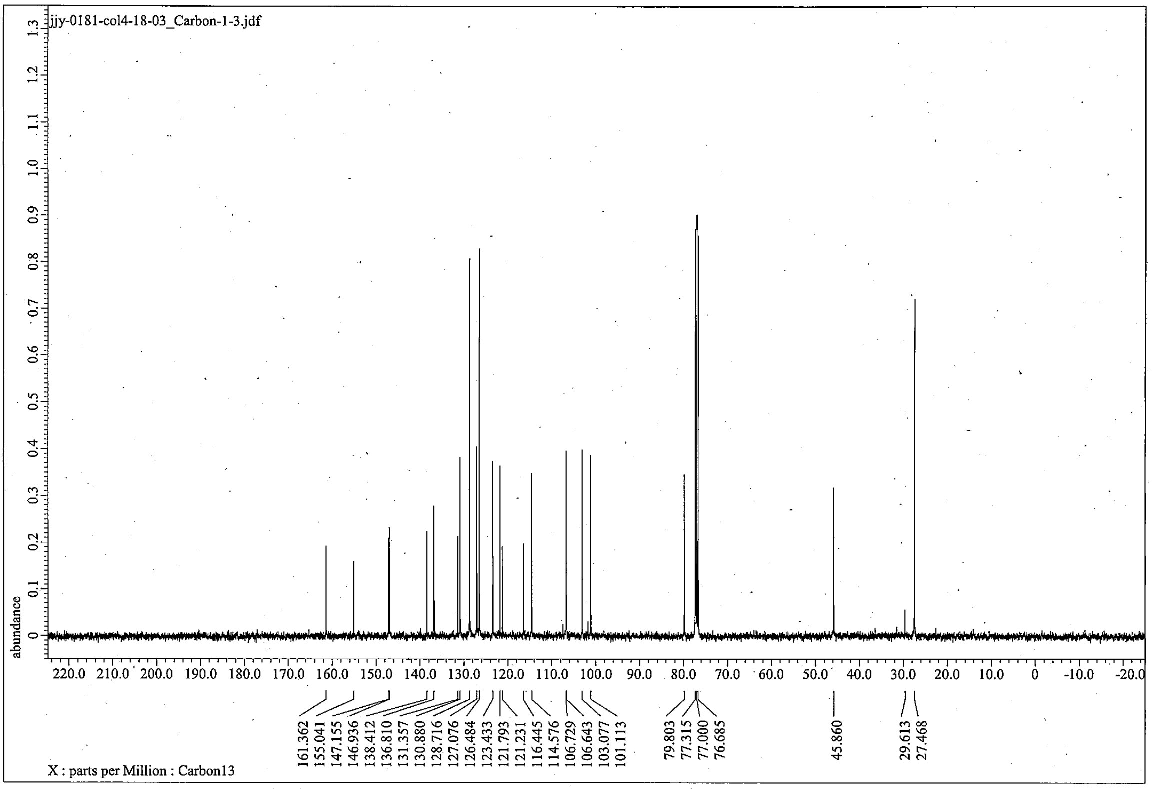
jjy-0181-col4-18-03_Carbon-1-3.jdf
X : parts per Million : Carbon13
abundance

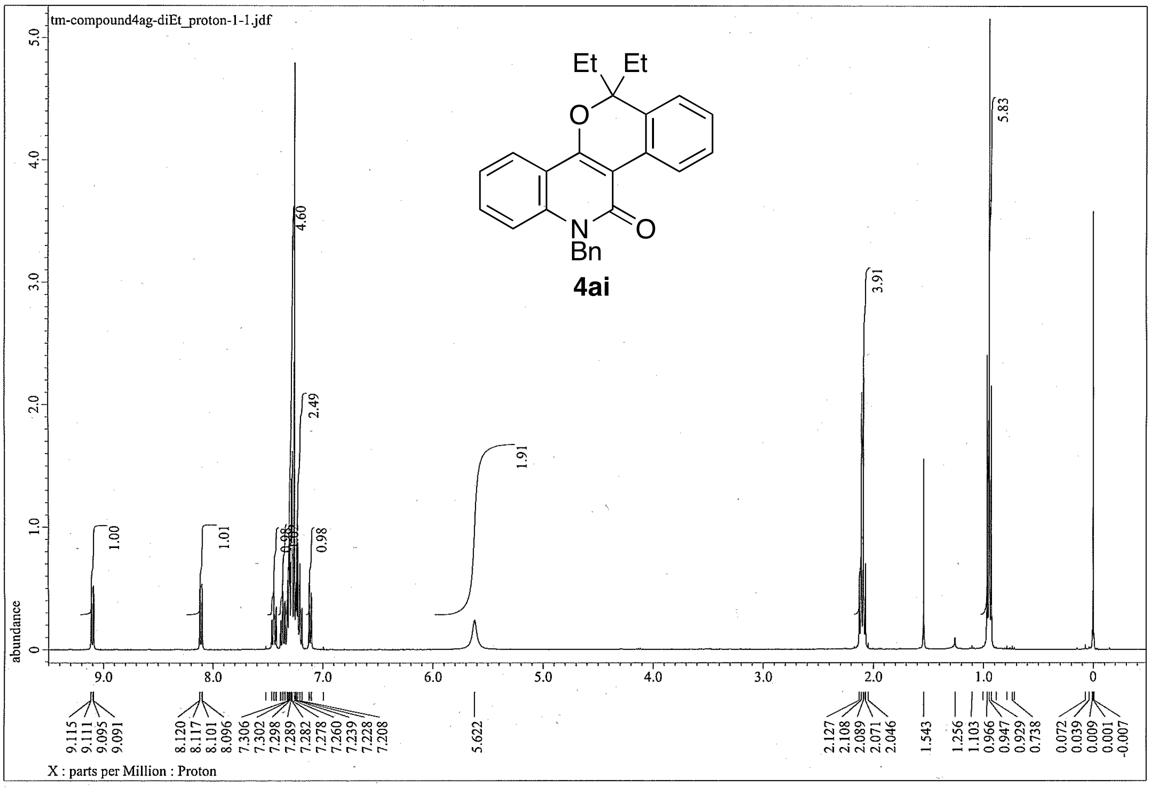
tm-compound4ag-diEt_proton-1-1.jdf
4ai
X : parts per Million : Proton

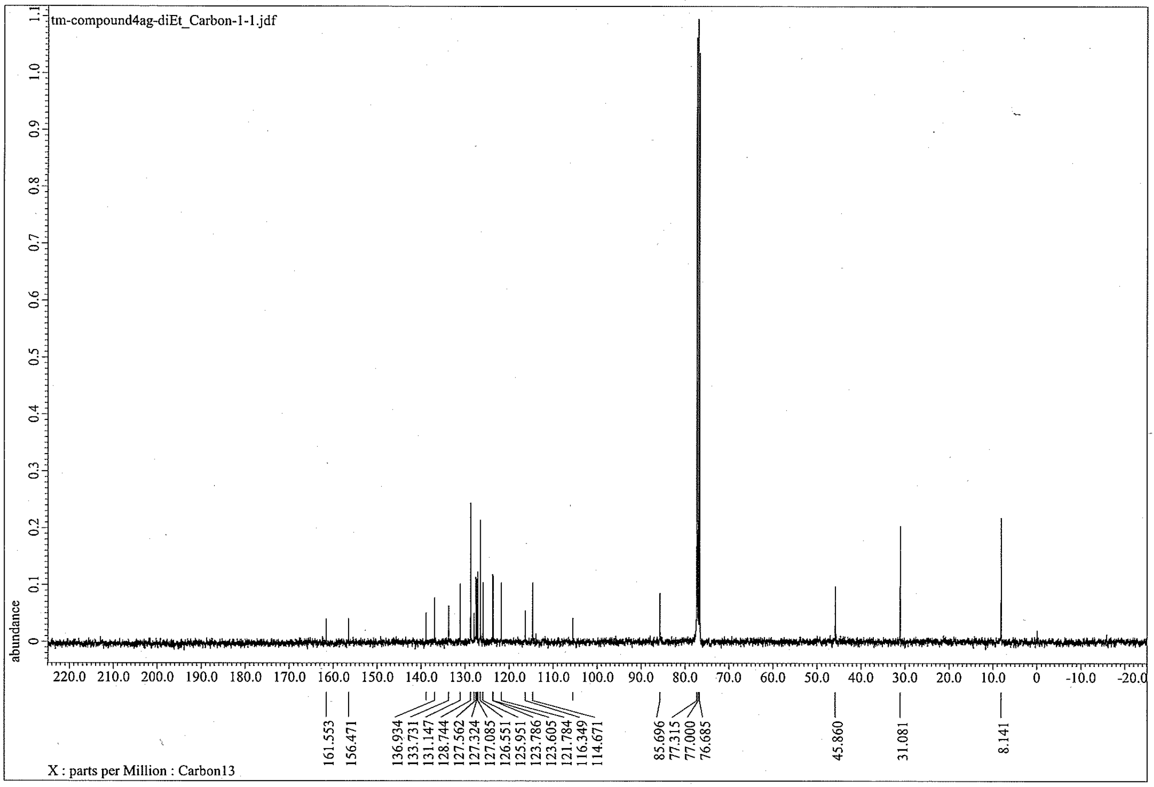
tm-compound4ag-diEt_Carbon-1-1.jdf
X : parts per Million : Carbon13

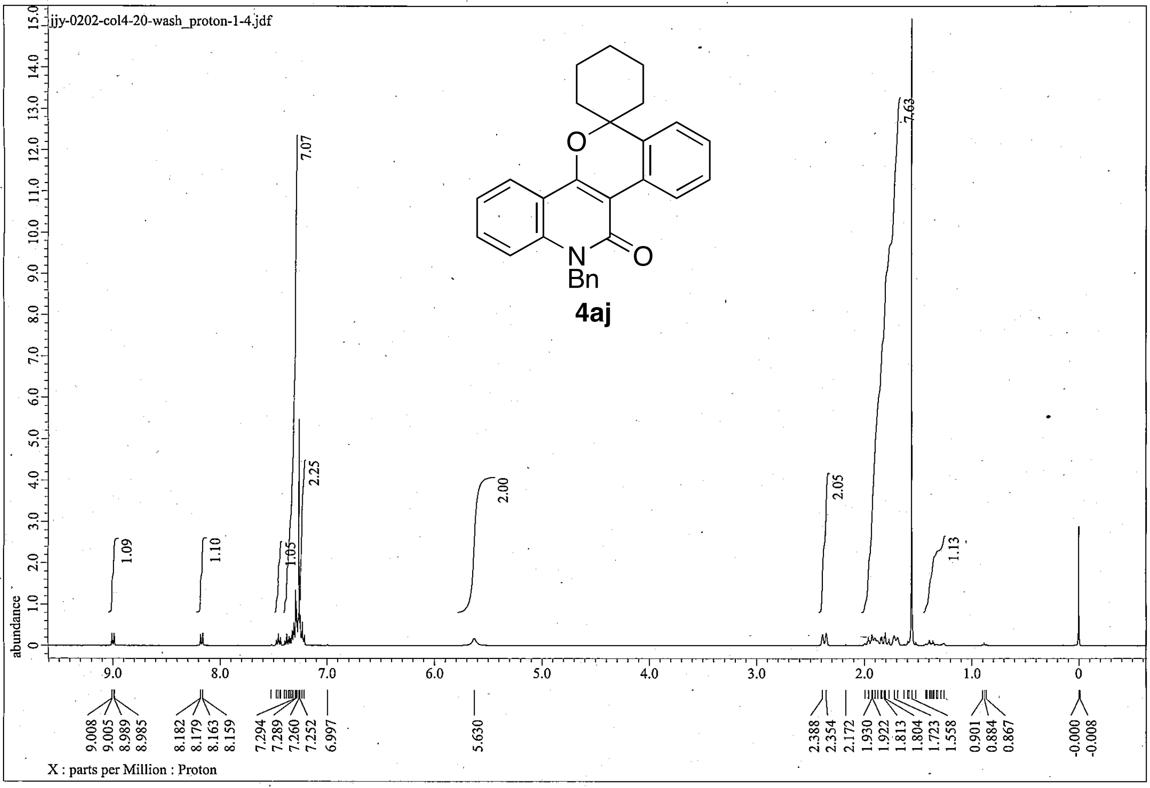
jjy-0202-col4-20-wash_proton-1-4.jdf
4aj
X : parts per Million : Proton
abundance

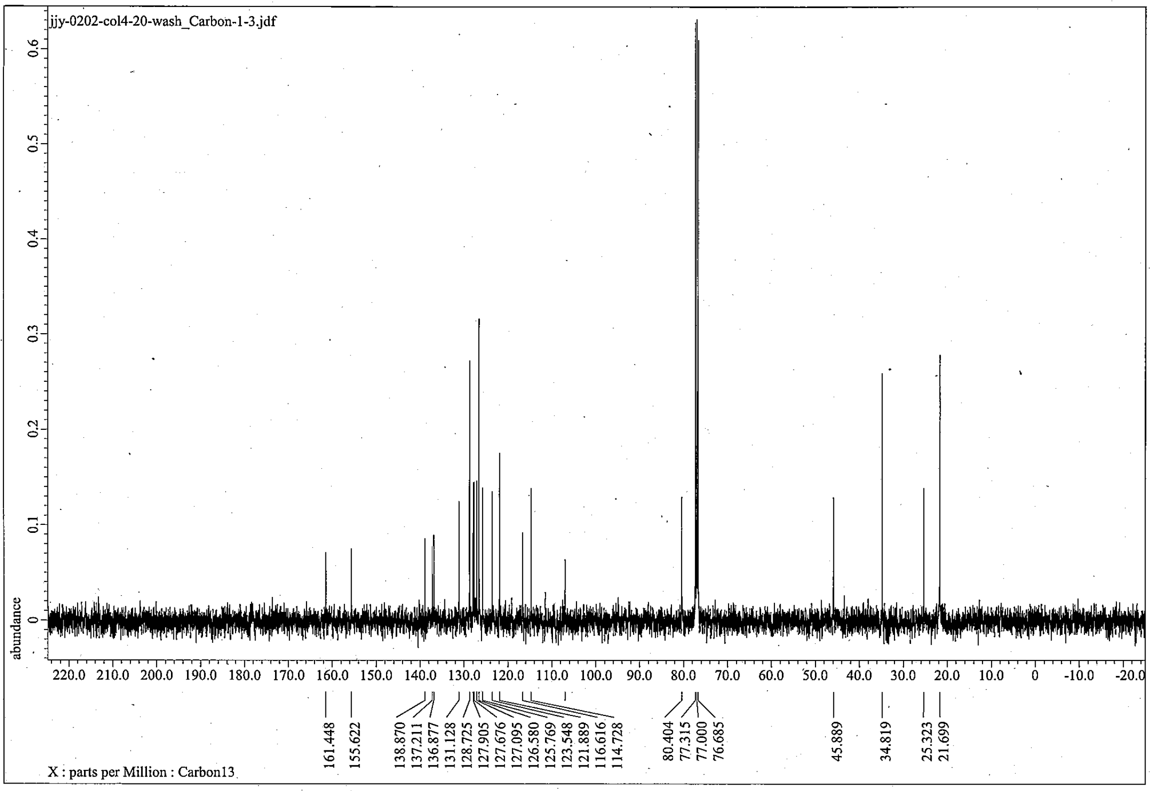
jjy-0202-col4-20-wash_Carbon-1-3.jdf
X : parts per Million : Carbon13
abundance

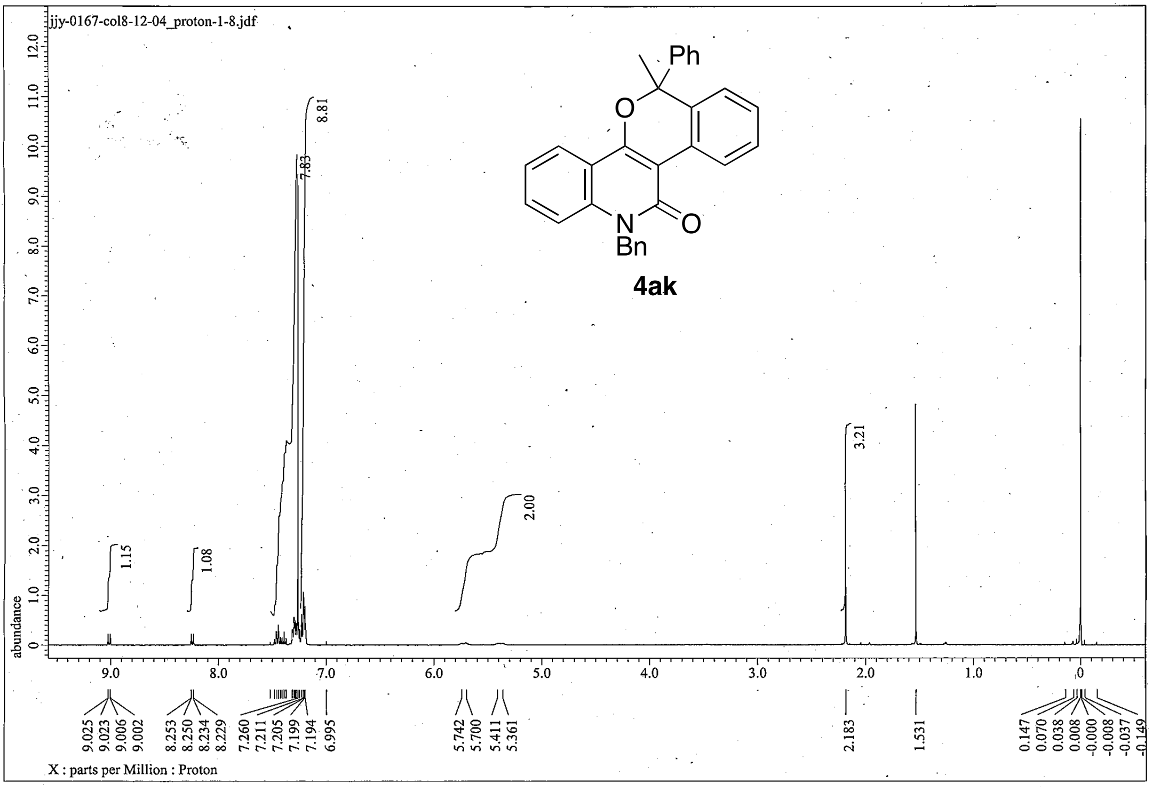
jjy-0167-col8-12-04_proton-1-8.jdf
Ph
O
N
O
Bn
4ak
abundance
X : parts per Million : Proton
1.15
1.08
7.83
8.81
2.00
3.21
9.025 9.023 9.006 9.002
8.253 8.250 8.234 8.229
7.260 7.211 7.205 7.199 7.194 6.995
5.742 5.700 5.411 5.361
2.183
1.531
0.147 0.070 0.038 0.008 -0.000 -0.008 -0.037 -0.149

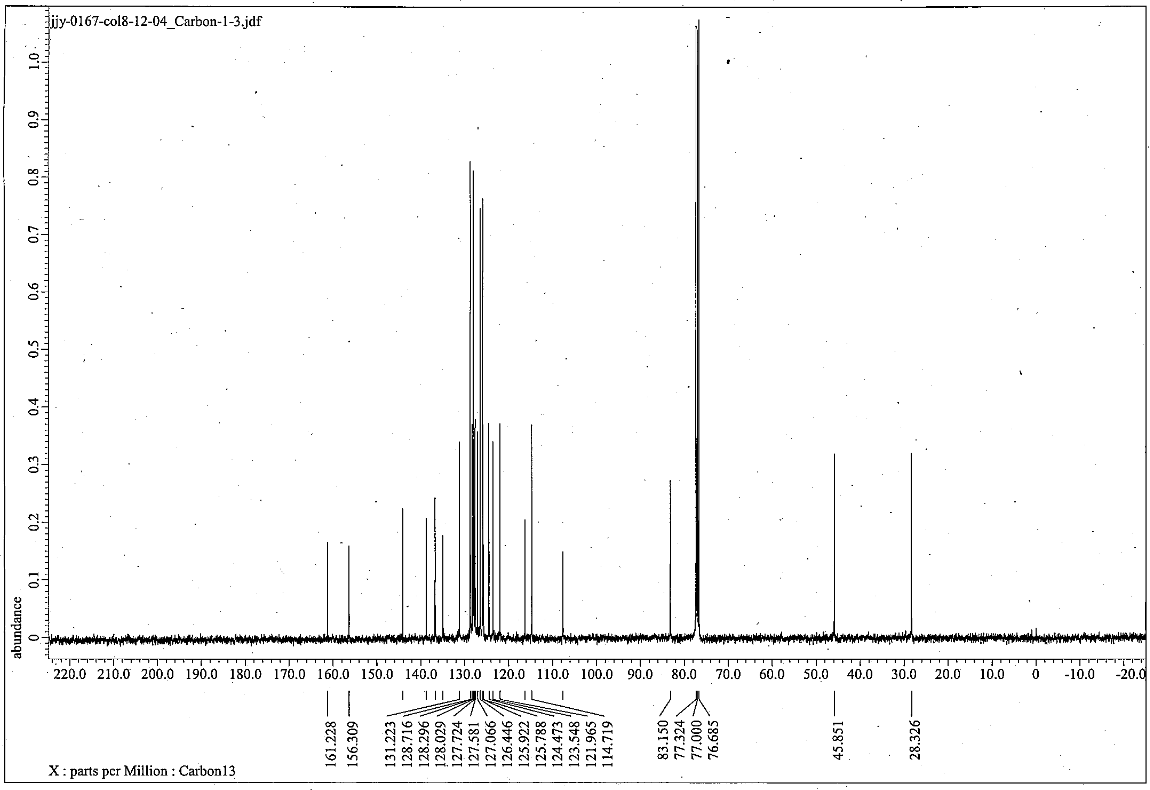
jjy-0167-col8-12-04_Carbon-1-3.jdf
abundance
X : parts per Million : Carbon13
161.228
156.309
131.223 128.716 128.296 128.029 127.724 127.581 127.066 126.446 125.922 125.788 124.473 123.548 121.965 114.719
83.150 77.324 77.000 76.685
45.851
28.326

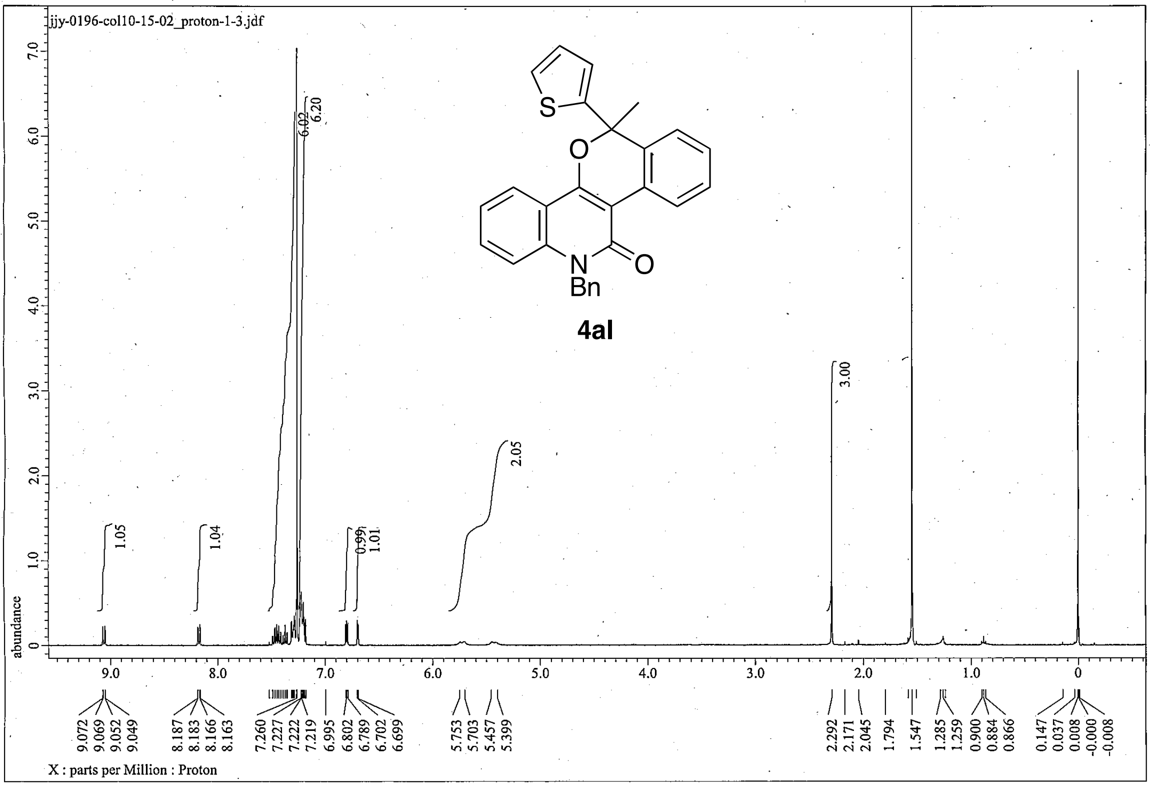
jjy-0196-col10-15-02_proton-1-3.jdf
4al
X : parts per Million : Proton
abundance

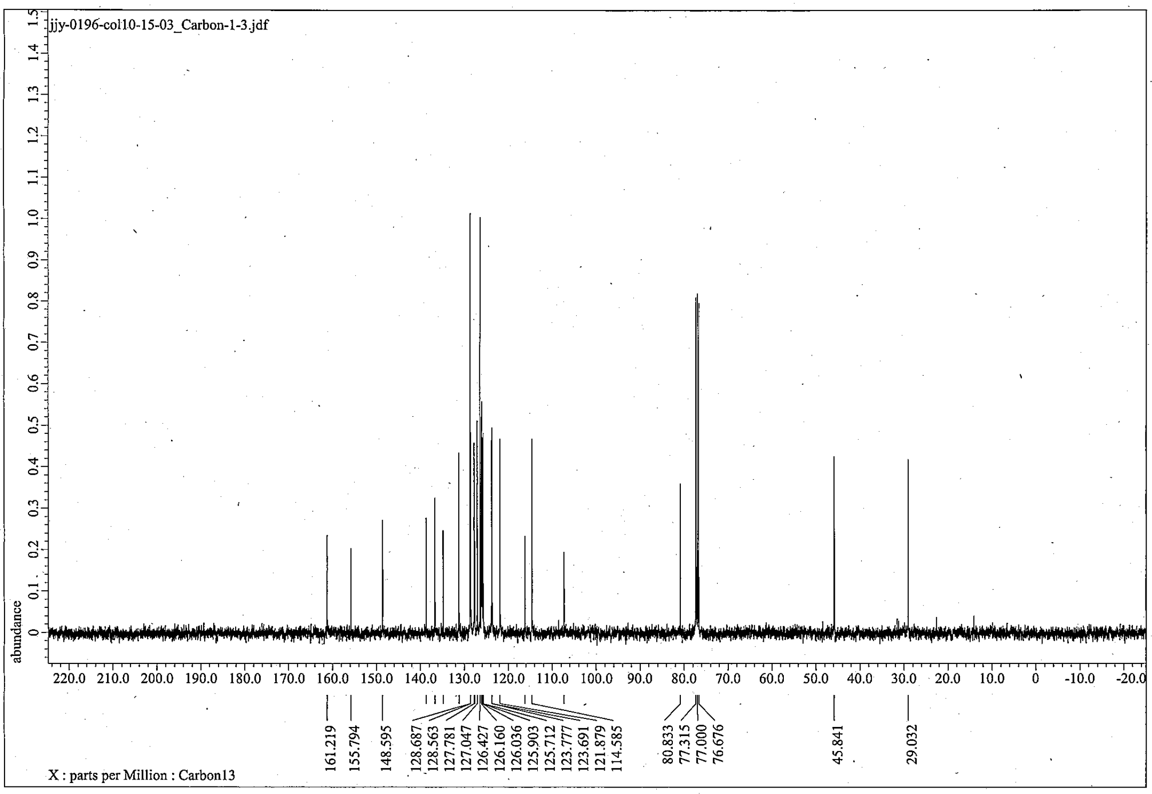
jjy-0196-col10-15-03_Carbon-1-3.jdf
X : parts per Million : Carbon13
abundance

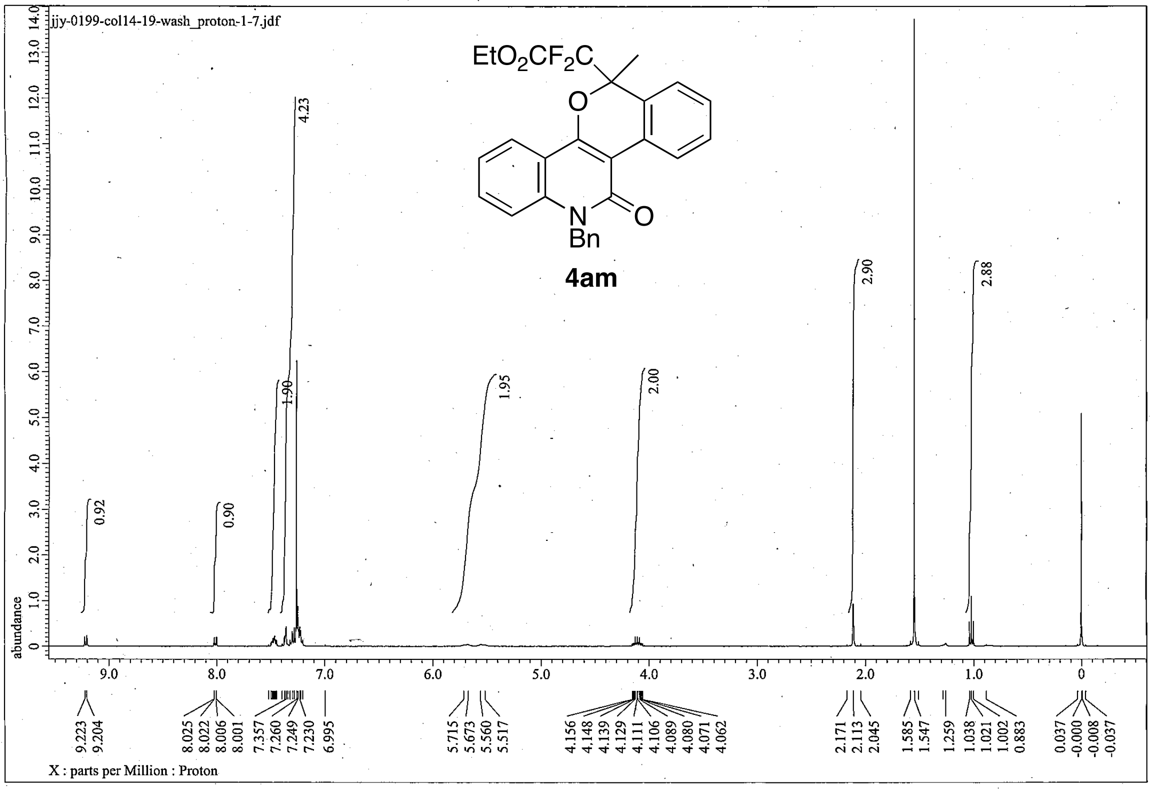
jjy-0199-col14-19-wash_proton-1-7.jdf
EtO2CF2C
O
N
O
Bn
4am
X : parts per Million : Proton
abundance

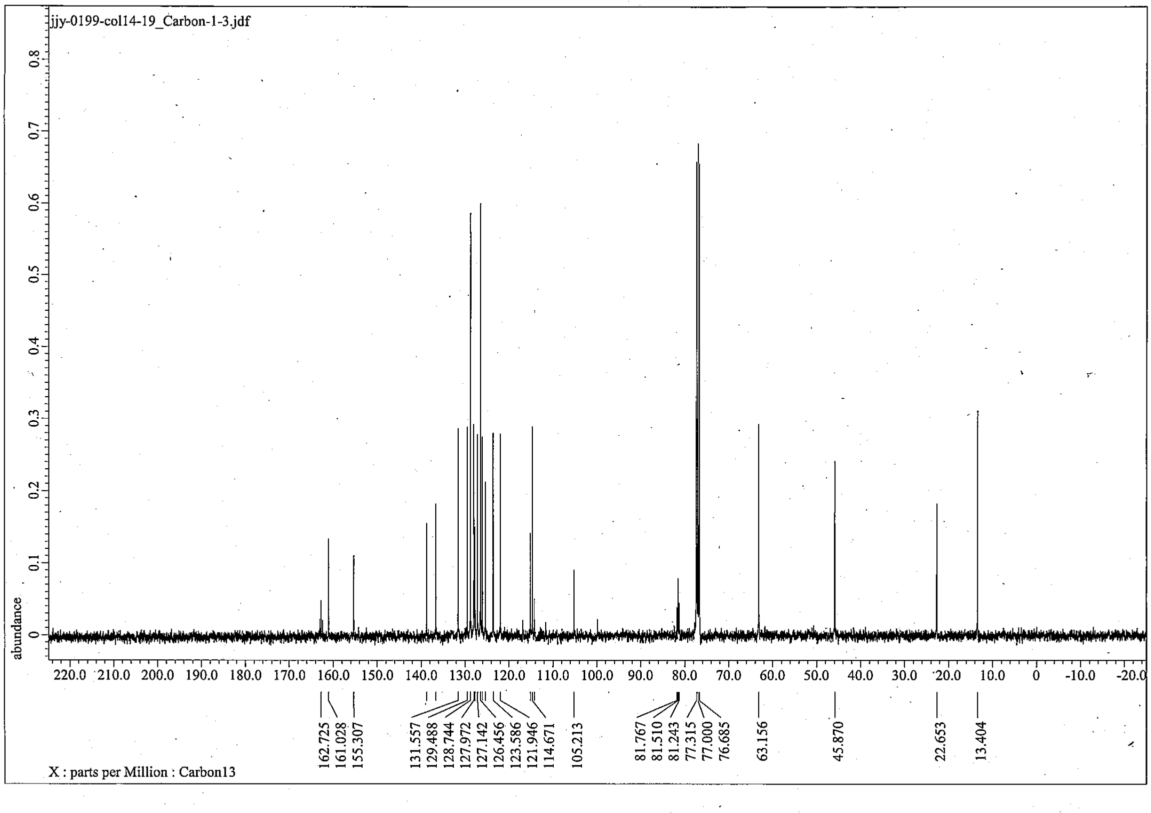
jjy-0199-col14-19_Carbon-1-3.jdf
X : parts per Million : Carbon13
abundance

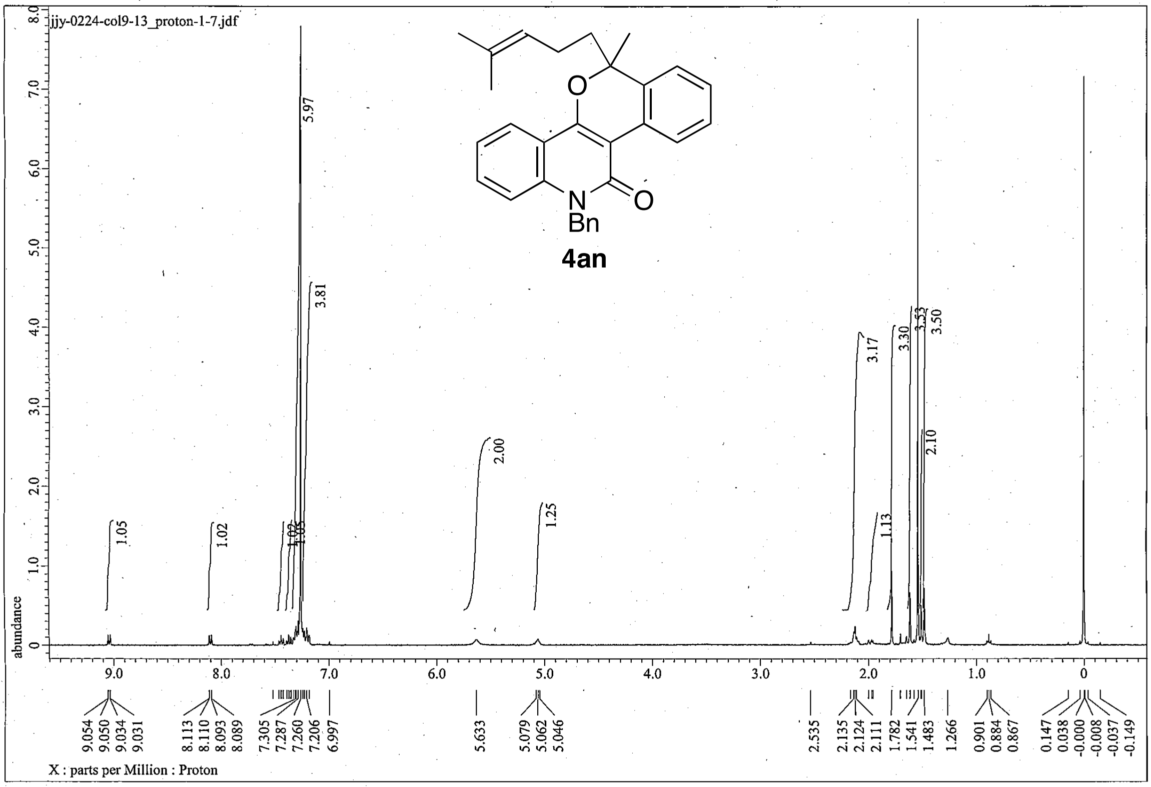
jjy-0224-col9-13_proton-1-7.jdf
4an
X : parts per Million : Proton
abundance

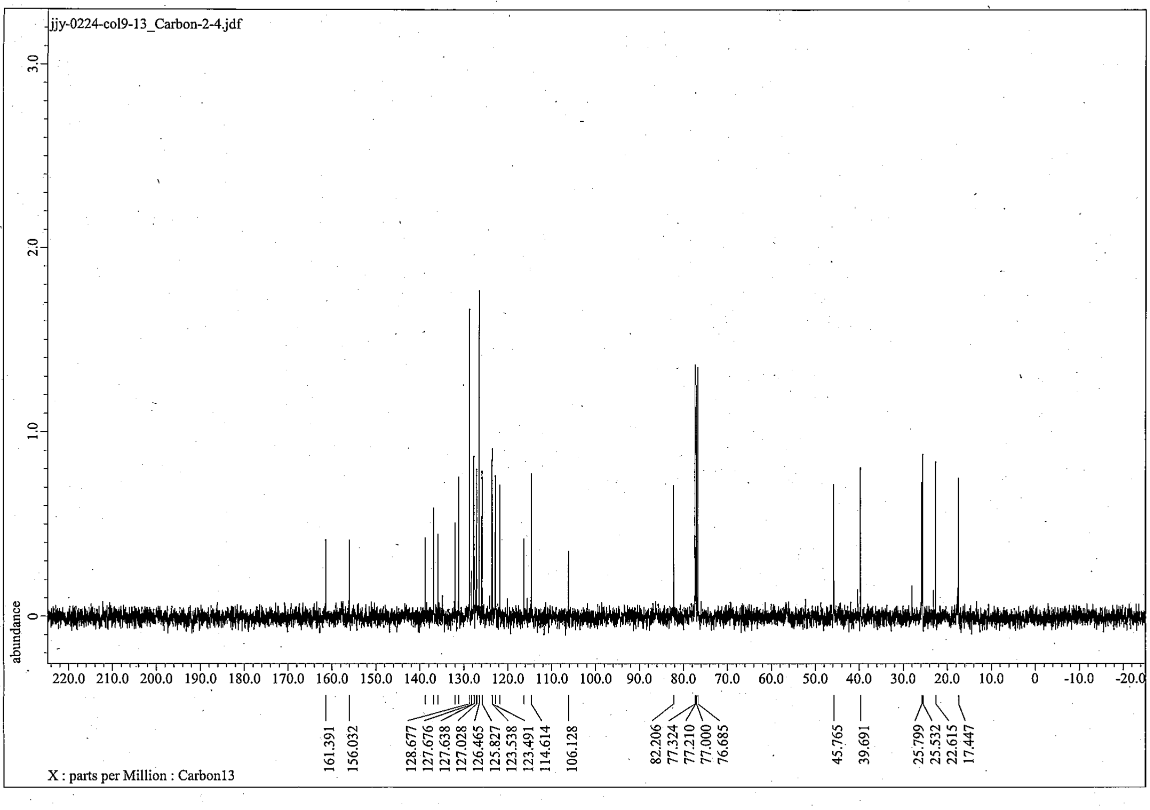
jjy-0224-col9-13_Carbon-2-4.jdf
161.391
156.032
128.677
127.676
127.638
127.028
126.465
125.827
123.538
123.491
114.614
106.128
82.206
77.324
77.210
77.000
76.685
45.765
39.691
25.799
25.532
22.615
17.447
X : parts per Million : Carbon13
abundance

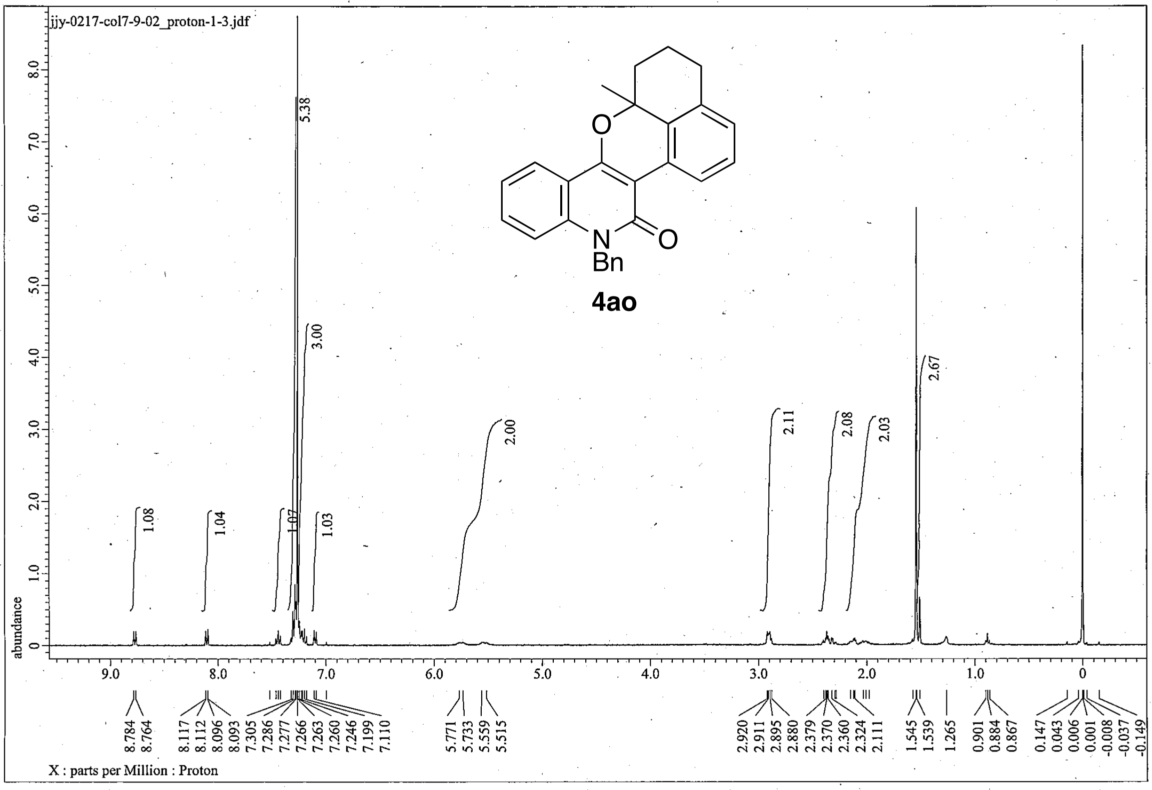
jjy-0217-col7-9-02_proton-1-3.jdf
4ao
N
Bn
O
X : parts per Million : Proton
abundance
8.784
8.764
8.117
8.112
8.096
8.093
7.305
7.286
7.277
7.266
7.263
7.260
7.246
7.199
7.110
5.771
5.733
5.559
5.515
2.920
2.911
2.895
2.880
2.379
2.370
2.360
2.324
2.111
1.545
1.539
1.265
0.901
0.884
0.867
0.147
0.043
0.006
0.001
-0.008
-0.037
-0.149
1.08
1.04
1.07
5.38
3.00
1.03
2.00
2.11
2.08
2.03
2.67

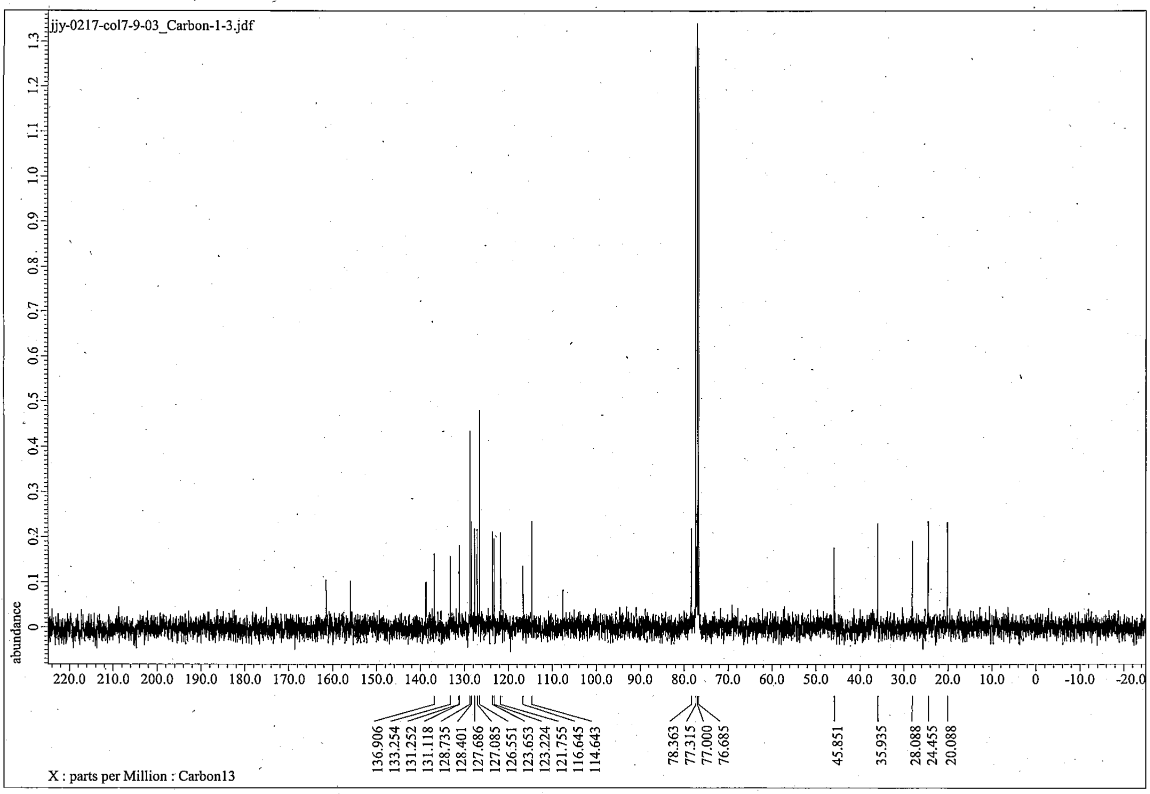
jjy-0217-col7-9-03_Carbon-1-3.jdf
X : parts per Million : Carbon13
abundance
136.906
133.254
131.252
131.118
128.735
128.401
127.686
127.085
126.551
123.653
123.224
121.755
116.645
114.643
78.363
77.315
77.000
76.685
45.851
35.935
28.088
24.455
20.088

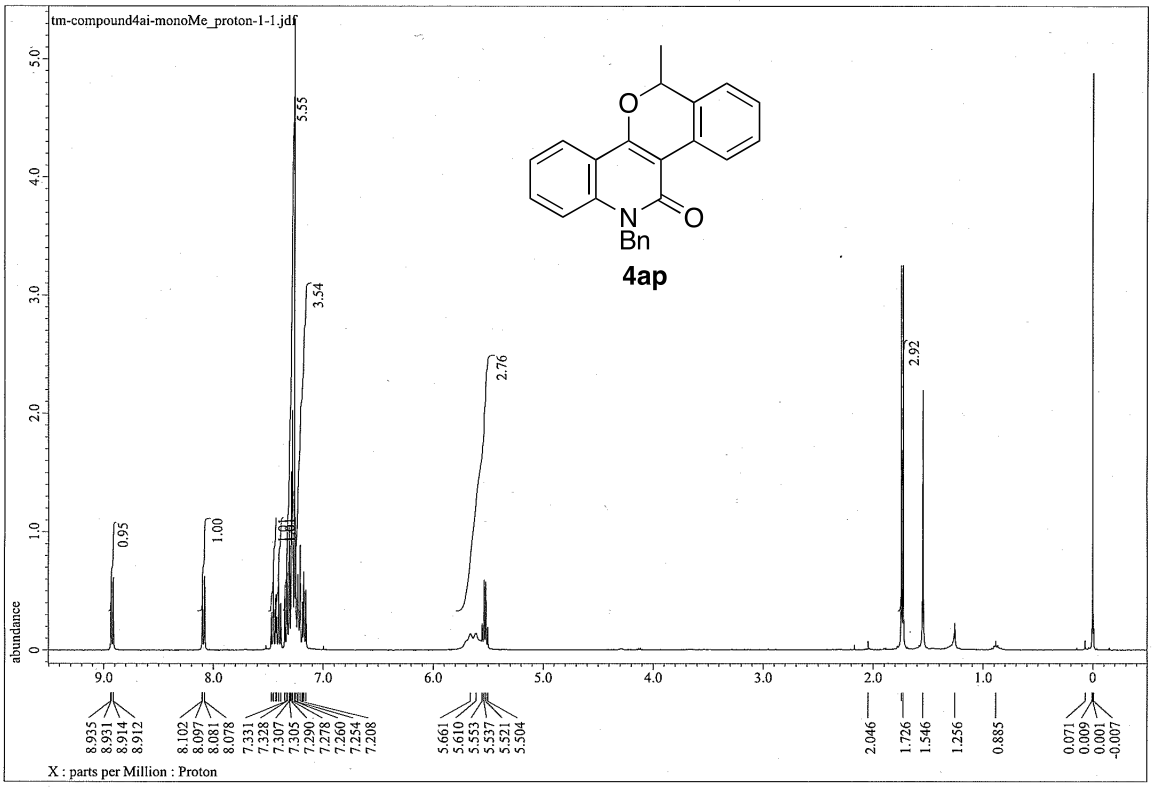
tm-compound4ai-monoMe_proton-1-1.jdf
4ap
Bn
X : parts per Million : Proton
abundance

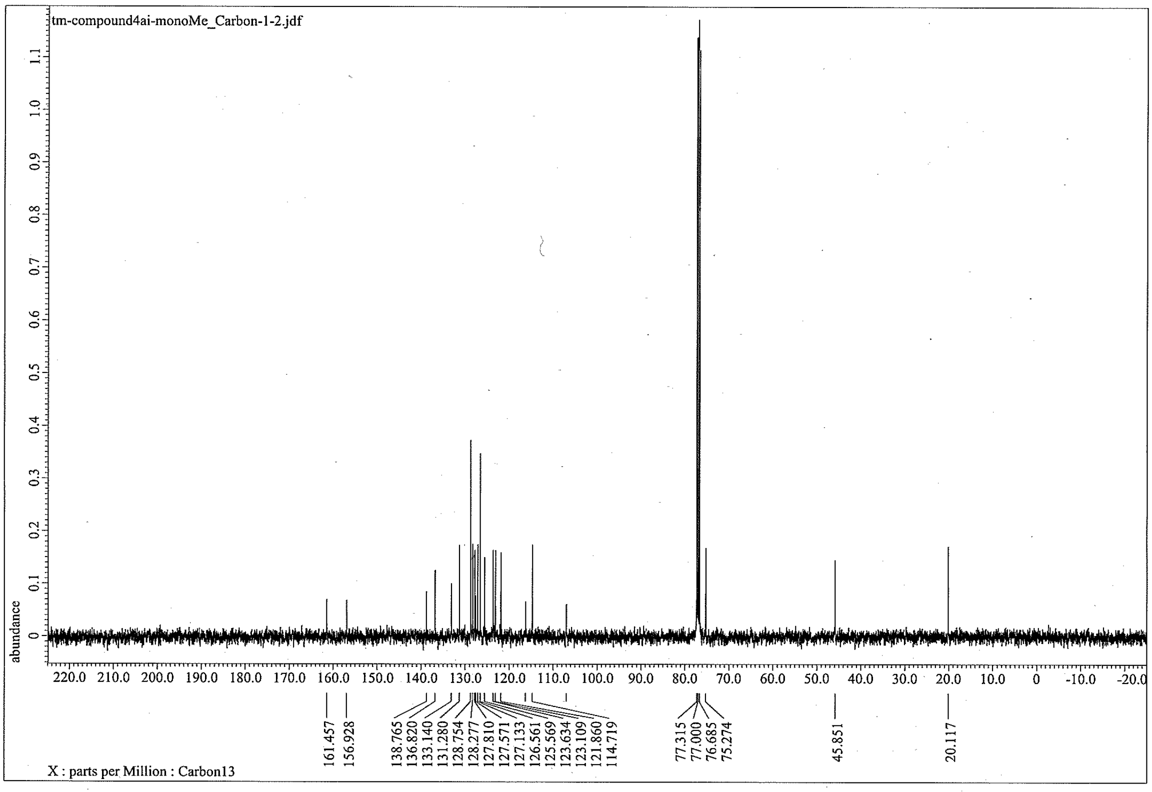
tm-compound4ai-monoMe_Carbon-1-2.jdf
X : parts per Million : Carbon13
abundance

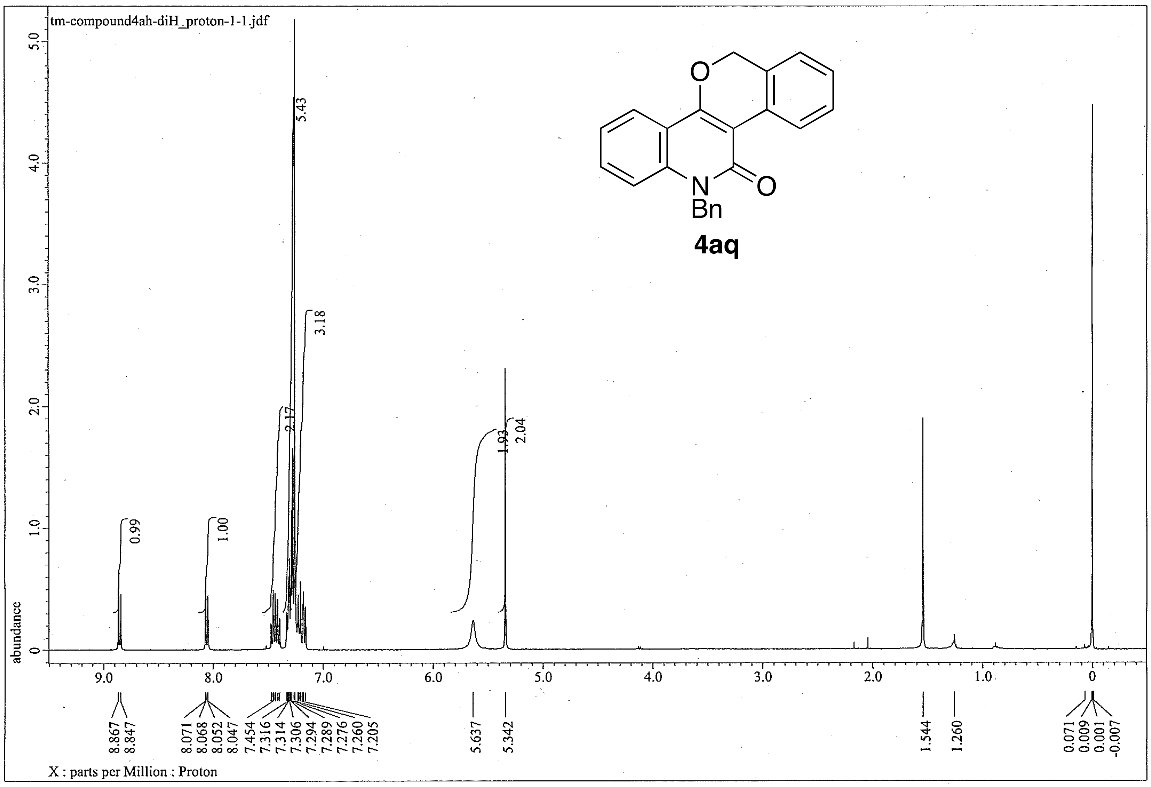
tm-compound4ah-diH_proton-1-1.jdf
4aq
O
N
Bn
O
5.43
3.18
2.17
2.04
1.93
0.99
1.00
abundance
8.867
8.847
8.071
8.068
8.052
8.047
7.454
7.316
7.314
7.306
7.294
7.289
7.276
7.260
7.205
5.637
5.342
1.544
1.260
0.071
0.009
0.001
-0.007
X : parts per Million : Proton

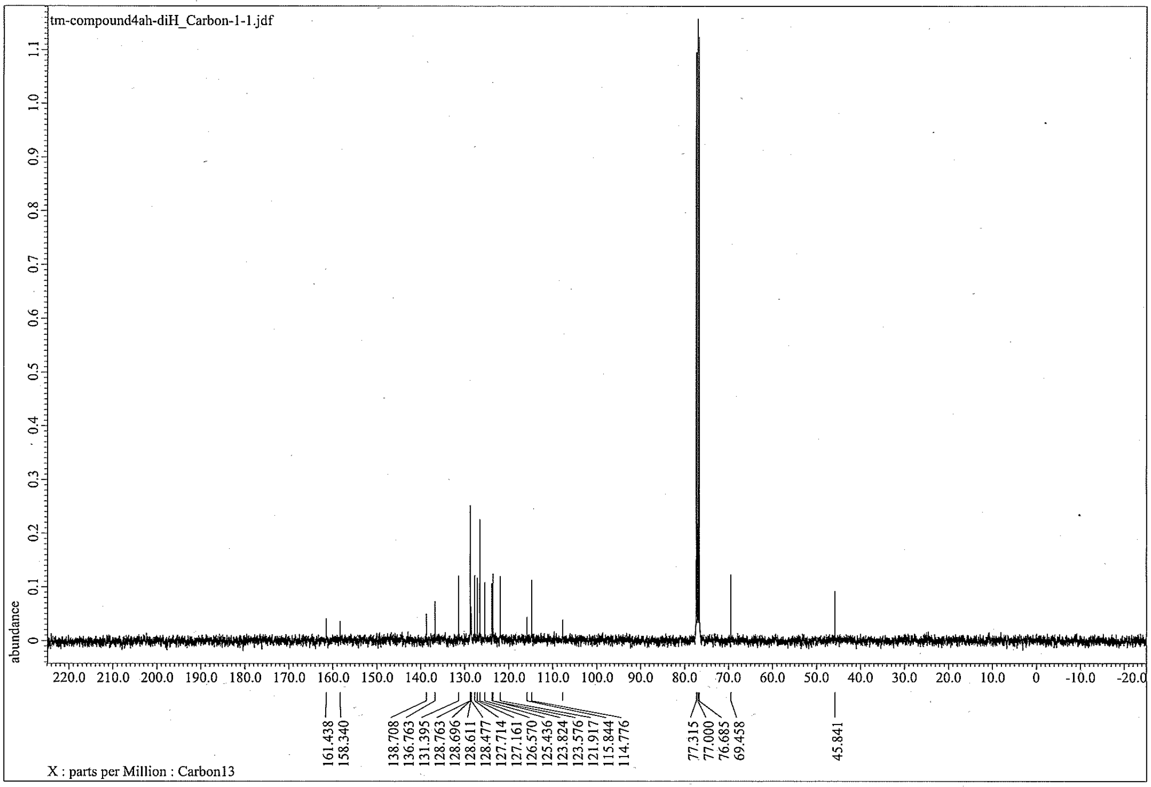
tm-compound4ah-diH_Carbon-1-1.jdf
abundance
161.438
158.340
138.708
136.763
131.395
128.763
128.696
128.611
128.477
127.714
127.161
126.570
125.436
123.824
123.576
121.917
115.844
114.776
77.315
77.000
76.685
69.458
45.841
X : parts per Million : Carbon13

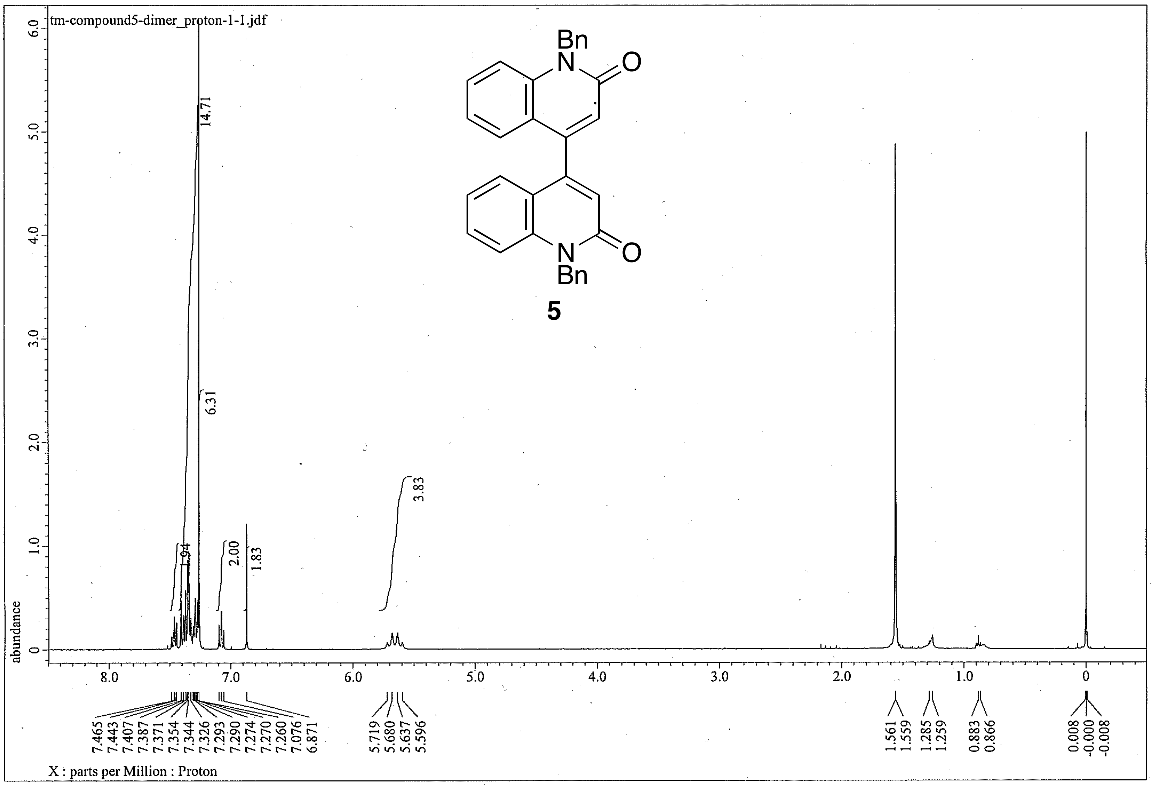
tm-compound5-dimer_proton-1-1.jdf
Bn
N
O
Bn
N
O
5
14.71
6.31
3.83
1.94
2.00
1.83
abundance
7.465 7.443 7.407 7.387 7.371 7.354 7.344 7.326 7.293 7.290 7.274 7.270 7.260 7.076 6.871
5.719 5.680 5.637 5.596
1.561 1.559 1.285 1.259 0.883 0.866 0.008 -0.000 -0.008
X : parts per Million : Proton

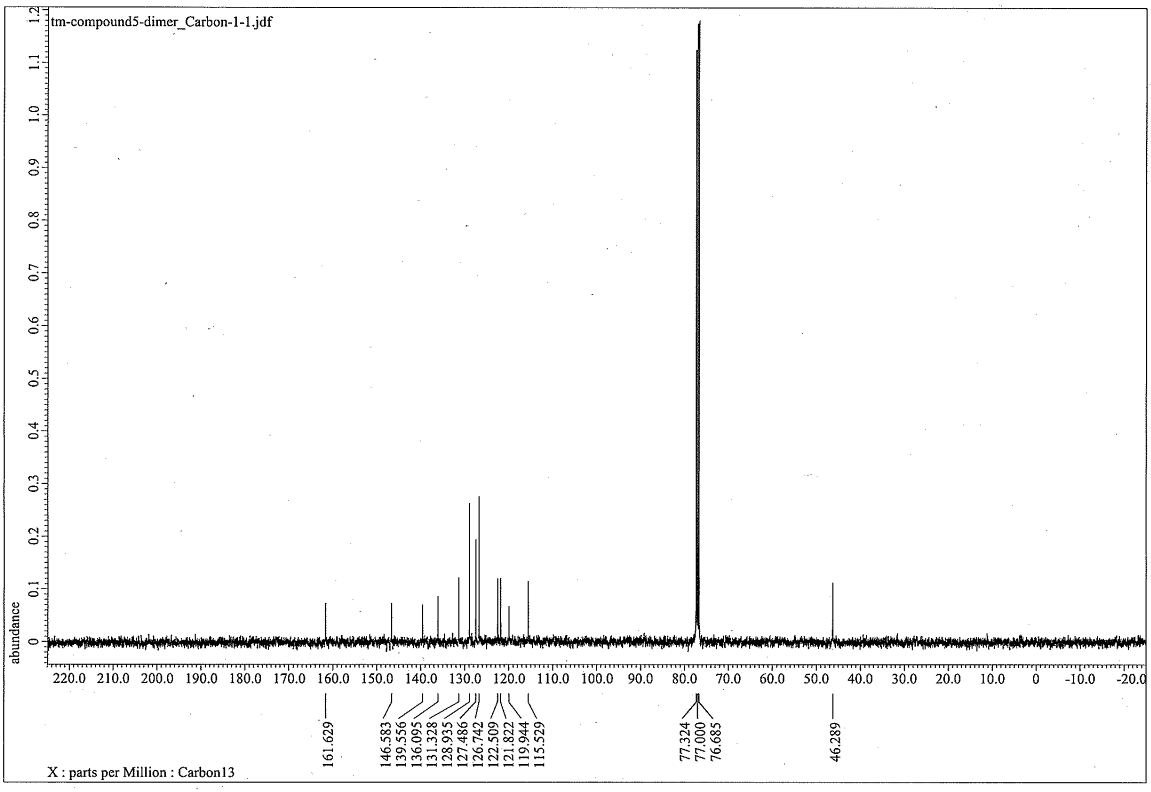
tm-compound5-dimer_Carbon-1-1.jdf
abundance
161.629
146.583 139.556 136.095 131.328 128.935 127.486 126.742 122.509 121.822 119.944 115.529
77.324 77.000 76.685
46.289
X : parts per Million : Carbon13

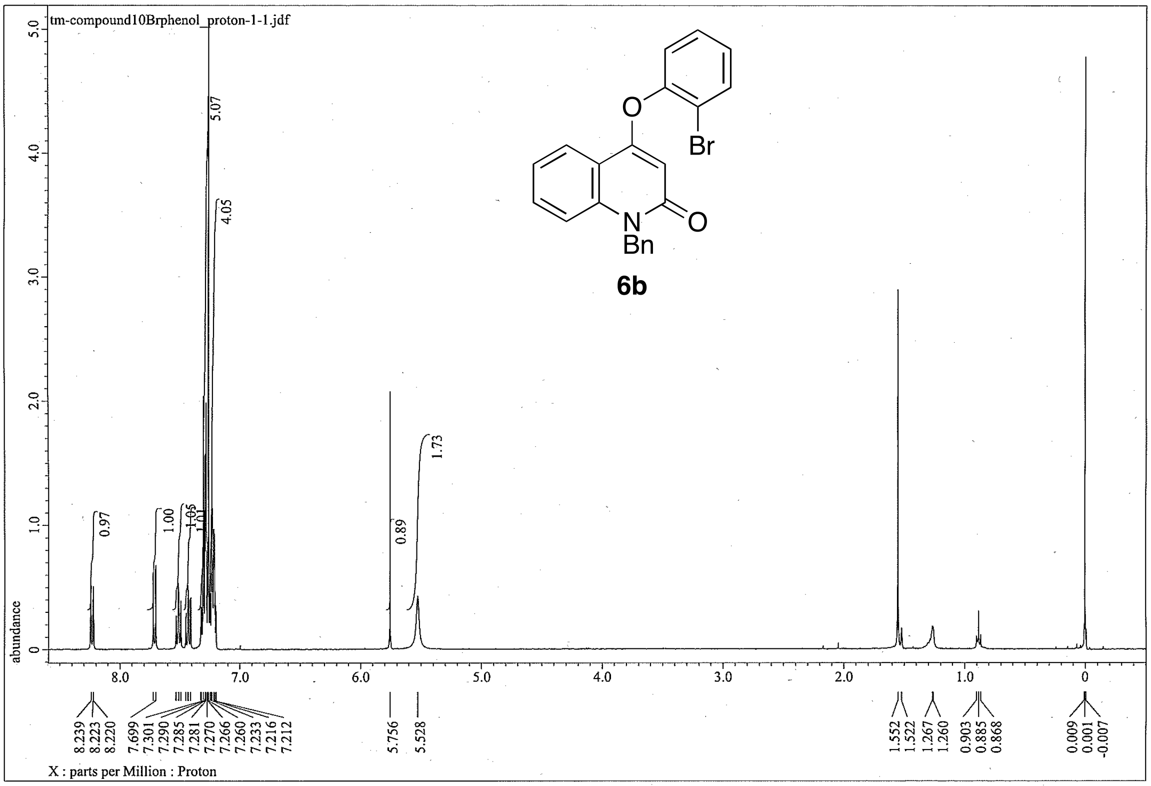
tm-compound10Brphenol_proton-1-1.jdf
Br
O
N
O
Bn
6b
X : parts per Million : Proton
abundance

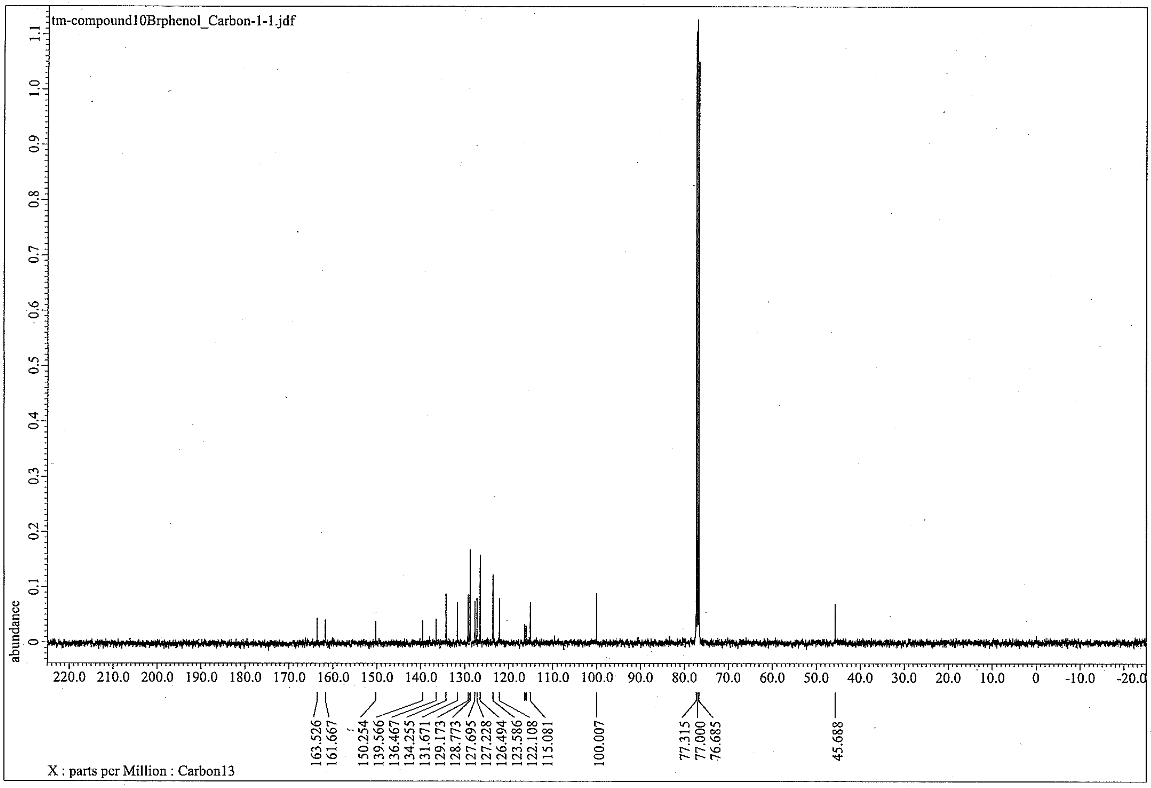
tm-compound10Brphenol_Carbon-1-1.jdf
X : parts per Million : Carbon13
abundance

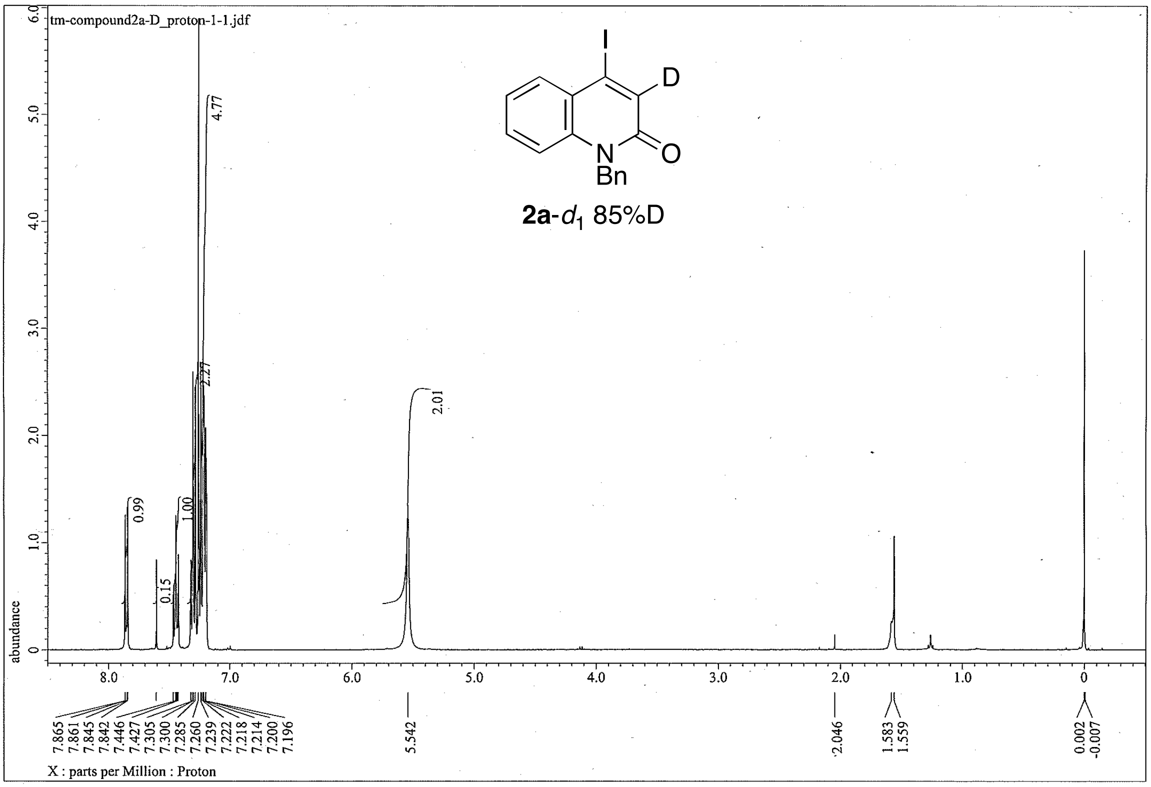
tm-compound2a-D_proton-1-1.jdf
I
D
N
O
Bn
2a-d1 85%D
0.99
0.15
1.00
2.27
4.77
2.01
7.865
7.861
7.845
7.842
7.446
7.427
7.305
7.300
7.285
7.260
7.239
7.222
7.218
7.214
7.200
7.196
5.542
2.046
1.583
1.559
0.002
-0.007
abundance
X : parts per Million : Proton

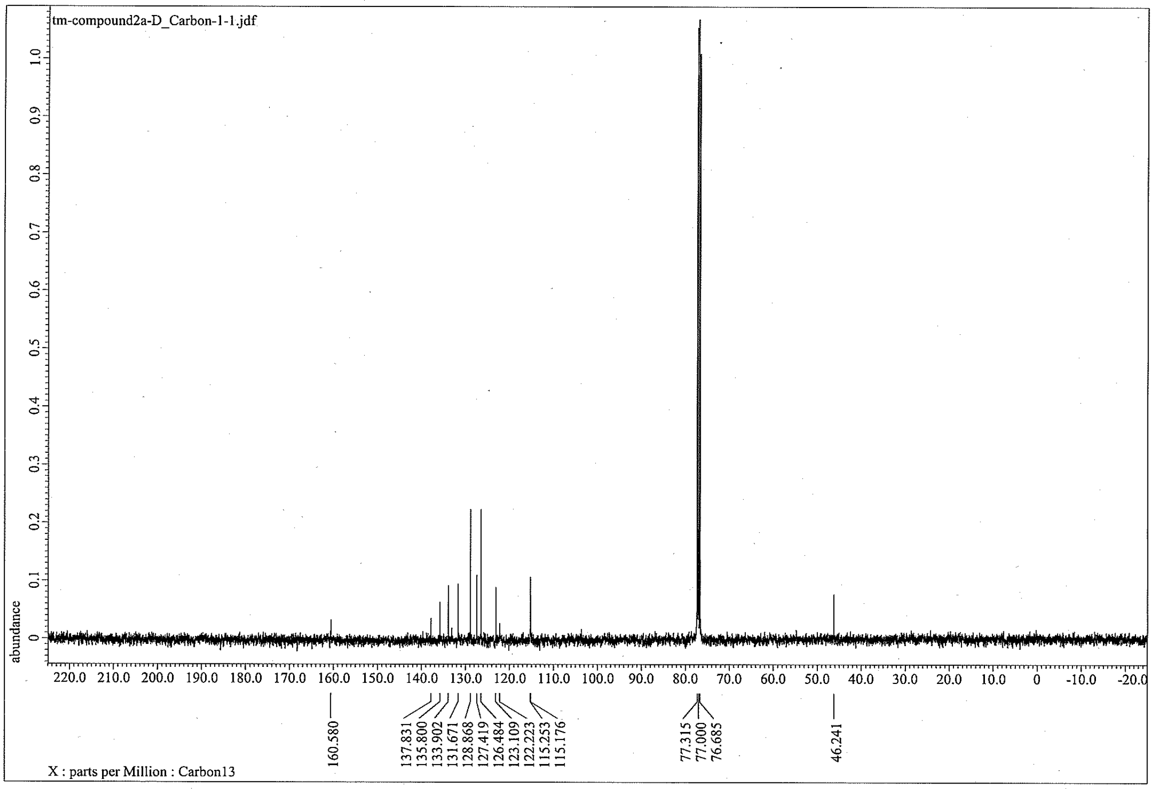
tm-compound2a-D_Carbon-1-1.jdf
160.580
137.831
135.800
133.902
131.671
128.868
127.419
126.484
123.109
122.223
115.253
115.176
77.315
77.000
76.685
46.241
abundance
X : parts per Million : Carbon13

Data for Scheme 5

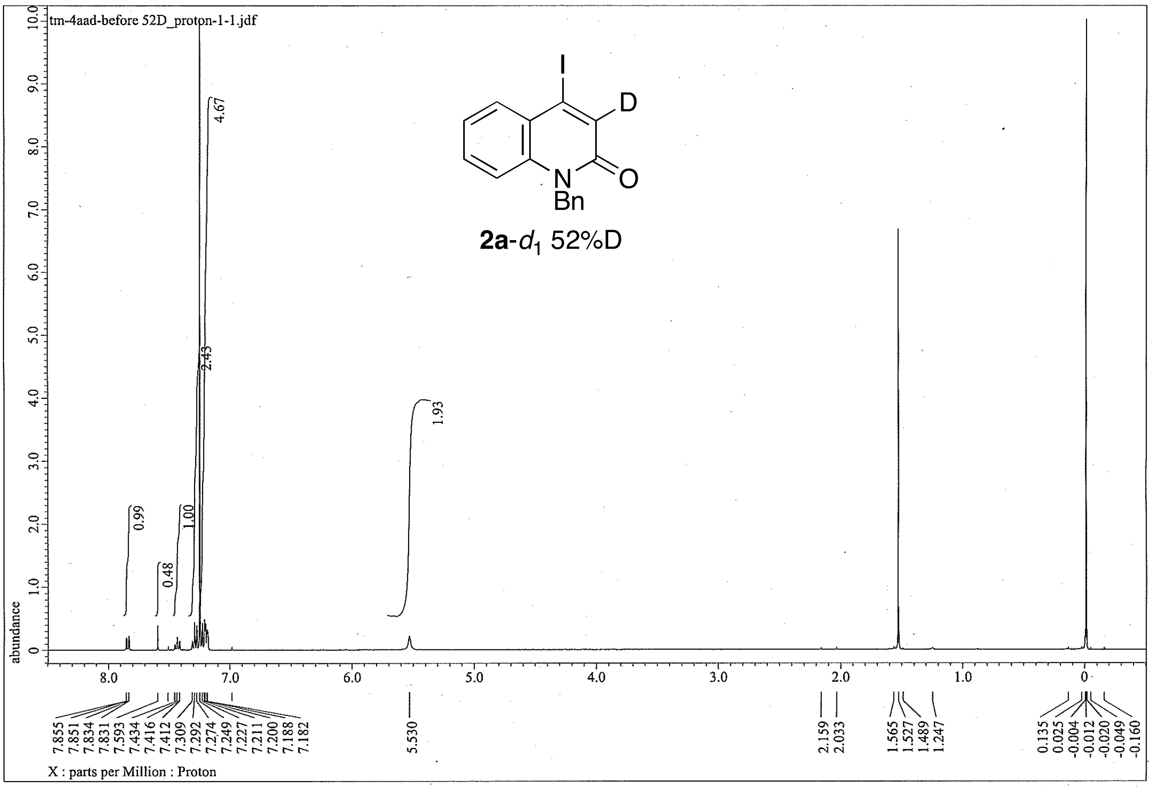

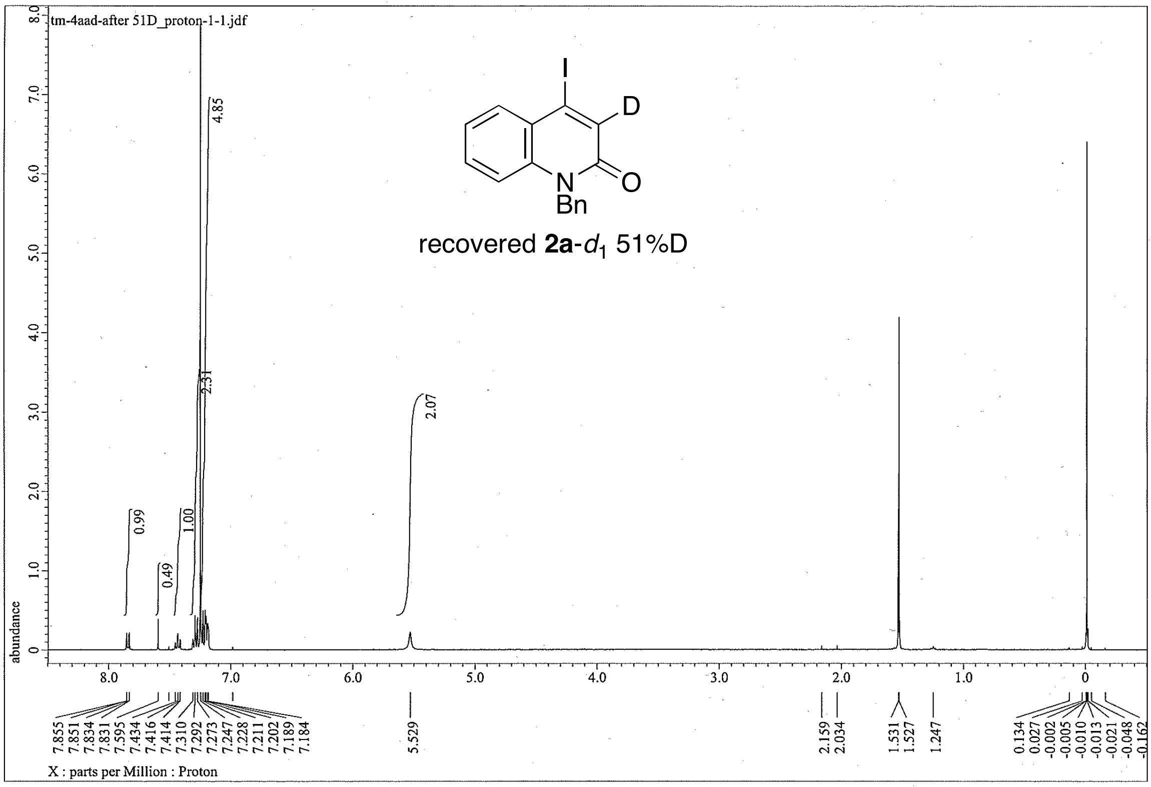

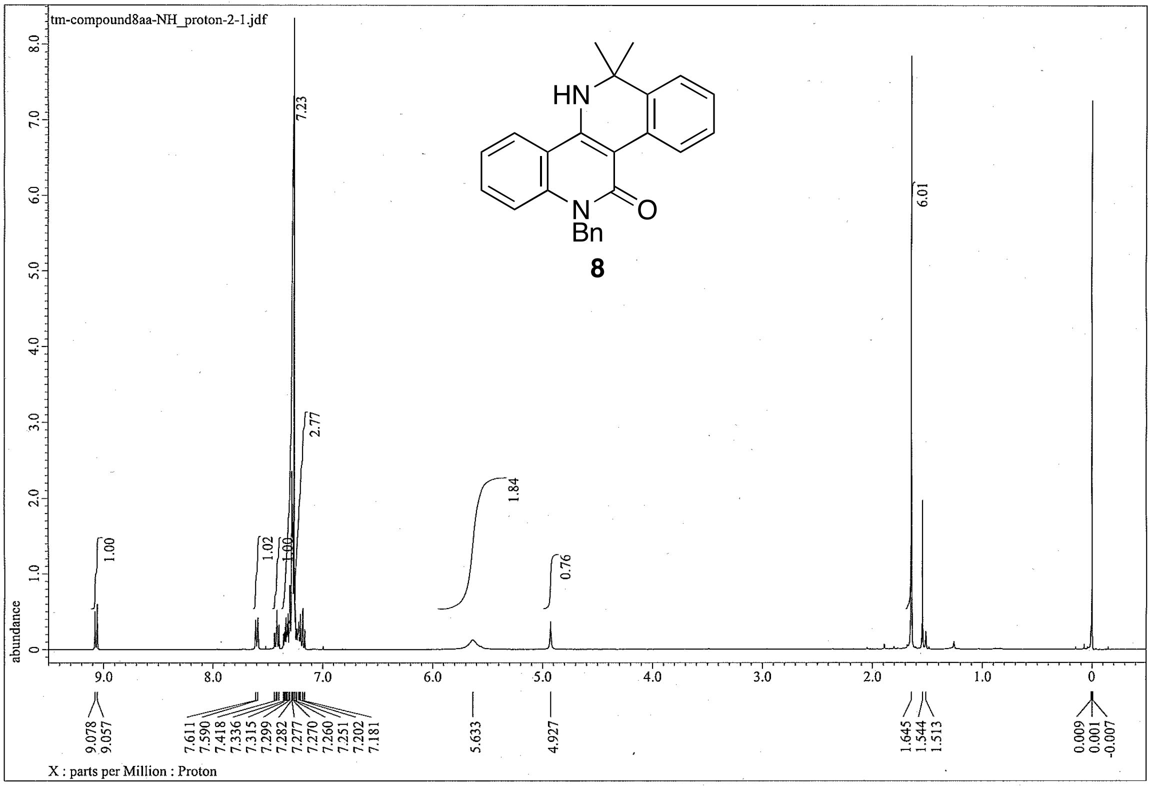

tm-compound8aa-NH_proton-2-1.jdf
HN
N
O
Bn
8
1.00
1.02
1.00
7.23
2.77
1.84
0.76
6.01
9.078
9.057
7.611
7.590
7.418
7.336
7.315
7.299
7.282
7.277
7.270
7.260
7.251
7.202
7.181
5.633
4.927
1.645
1.544
1.513
0.009
0.001
-0.007
abundance
X : parts per Million : Proton

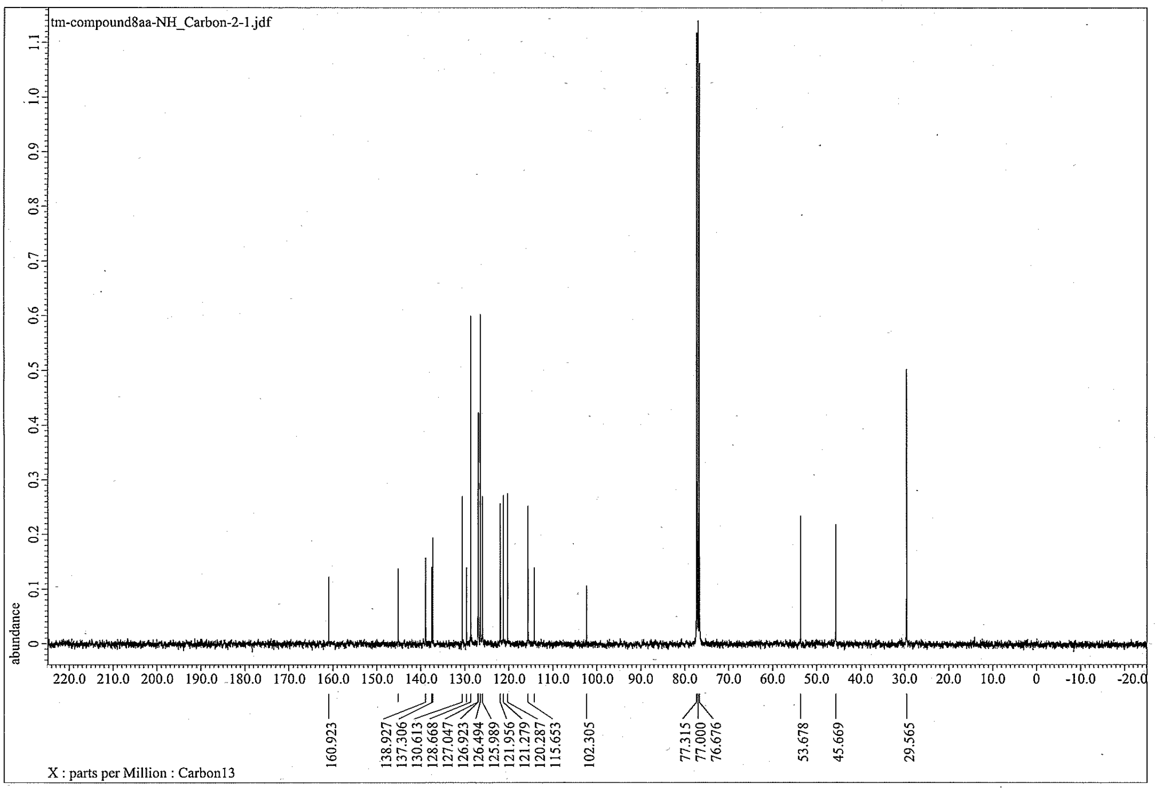

tm-compound8aa-NH_Carbon-2-1.jdf
160.923
138.927
137.306
130.613
128.668
127.047
126.923
126.494
125.989
121.956
121.279
120.287
115.653
102.305
77.315
77.000
76.676
53.678
45.669
29.565
abundance
X : parts per Million : Carbon13

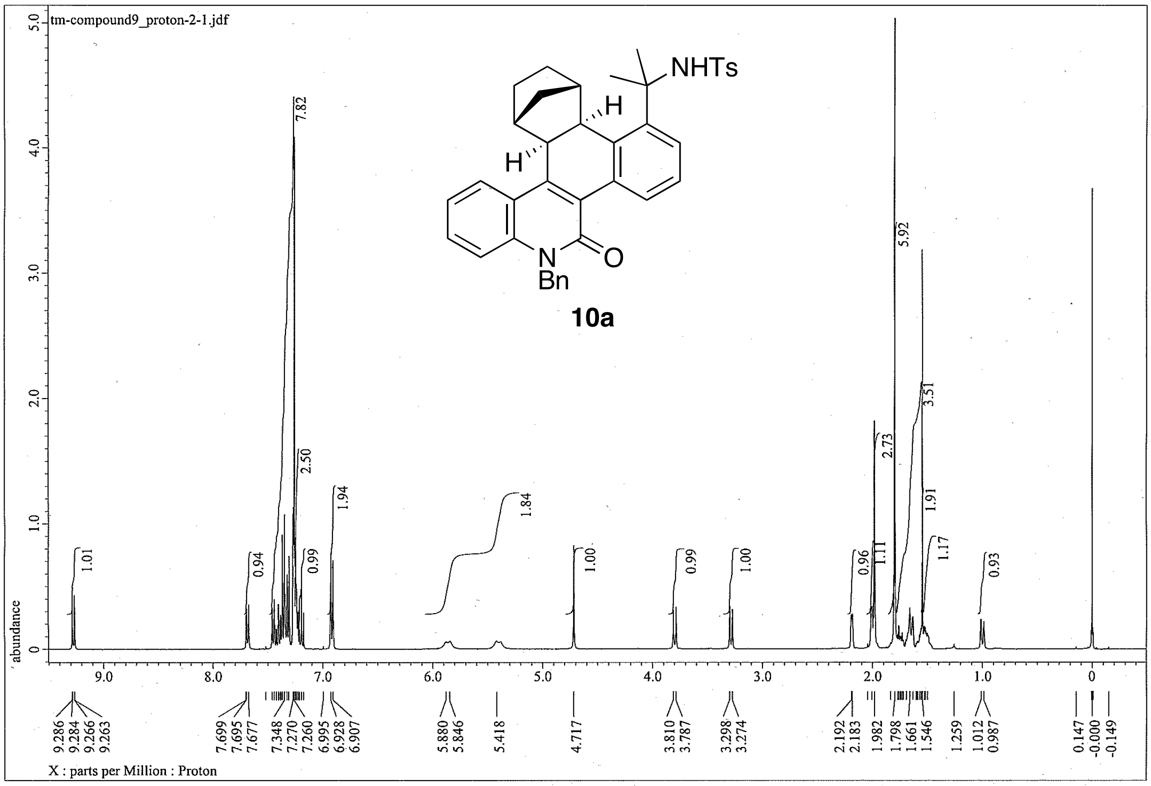
tm-compound9_proton-2-1.jdf
NHTs
H
H
N
O
Bn
10a
X : parts per Million : Proton
abundance

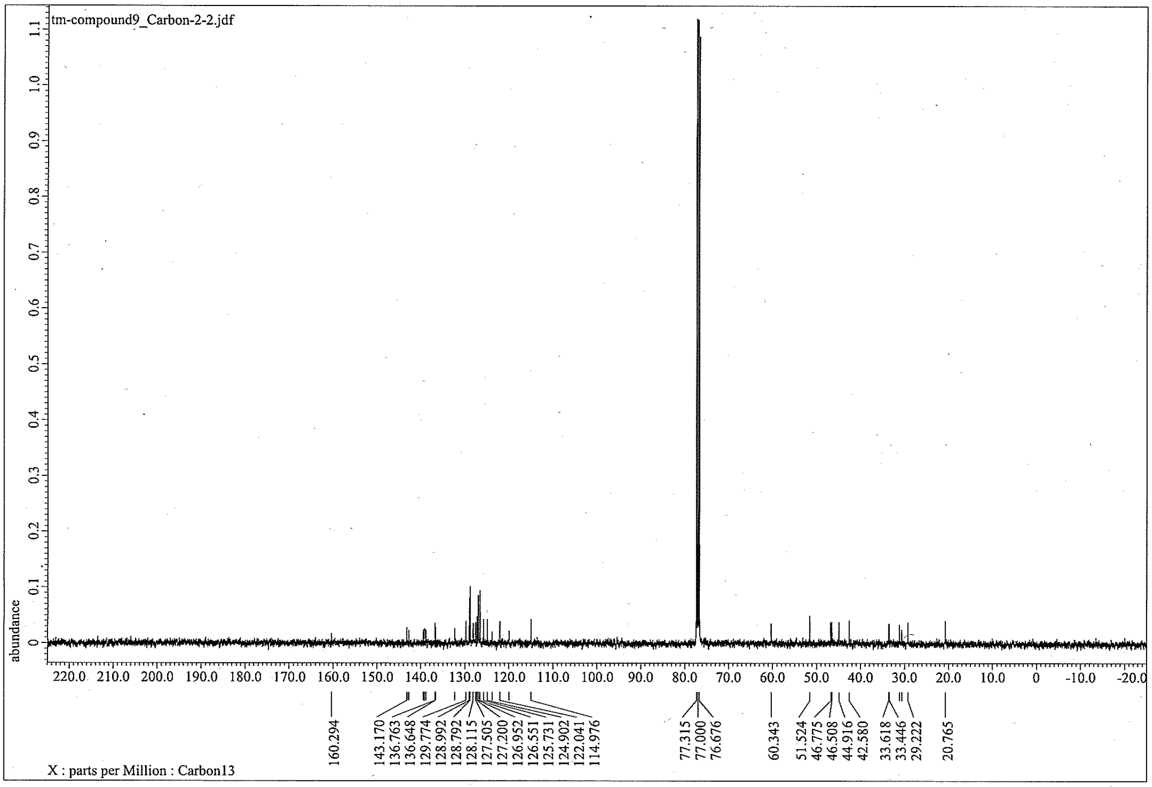
tm-compound9_Carbon-2-2.jdf
X : parts per Million : Carbon13
abundance

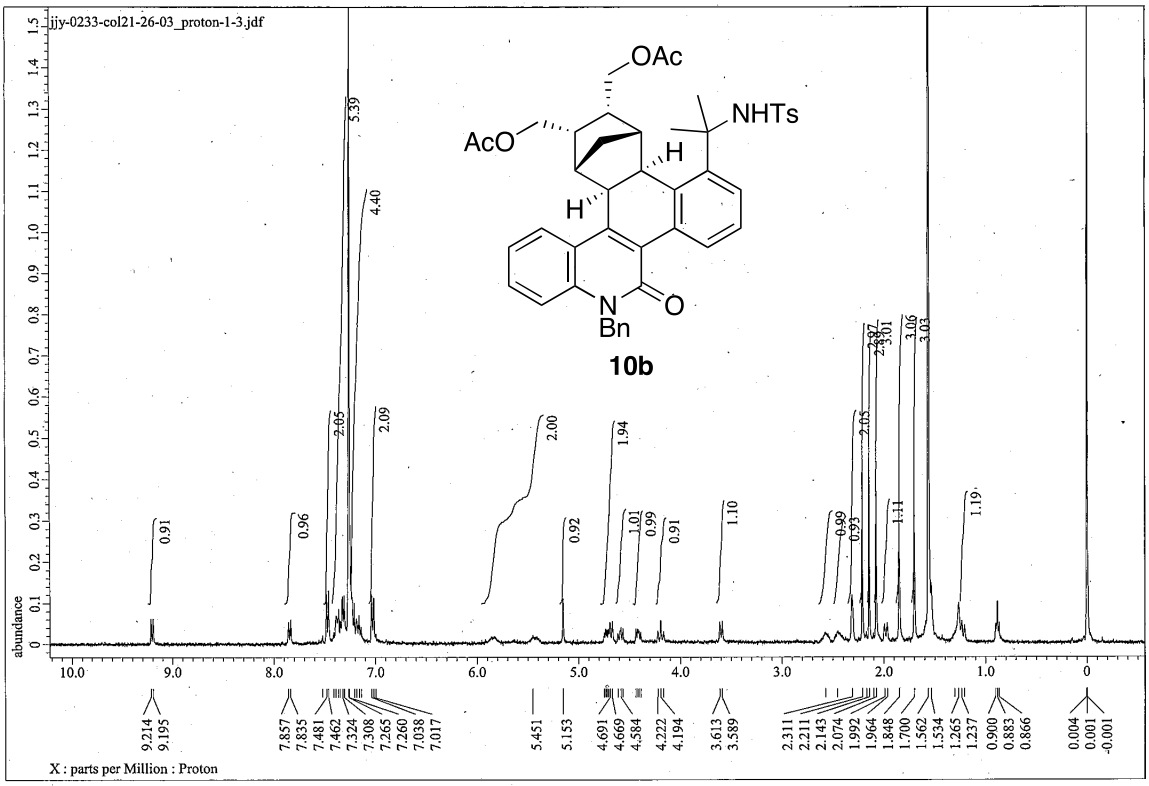
jjy-0233-col21-26-03_proton-1-3.jdf
OAc
AcO
NHTs
H
H
N
O
Bn
10b
X : parts per Million : Proton
abundance

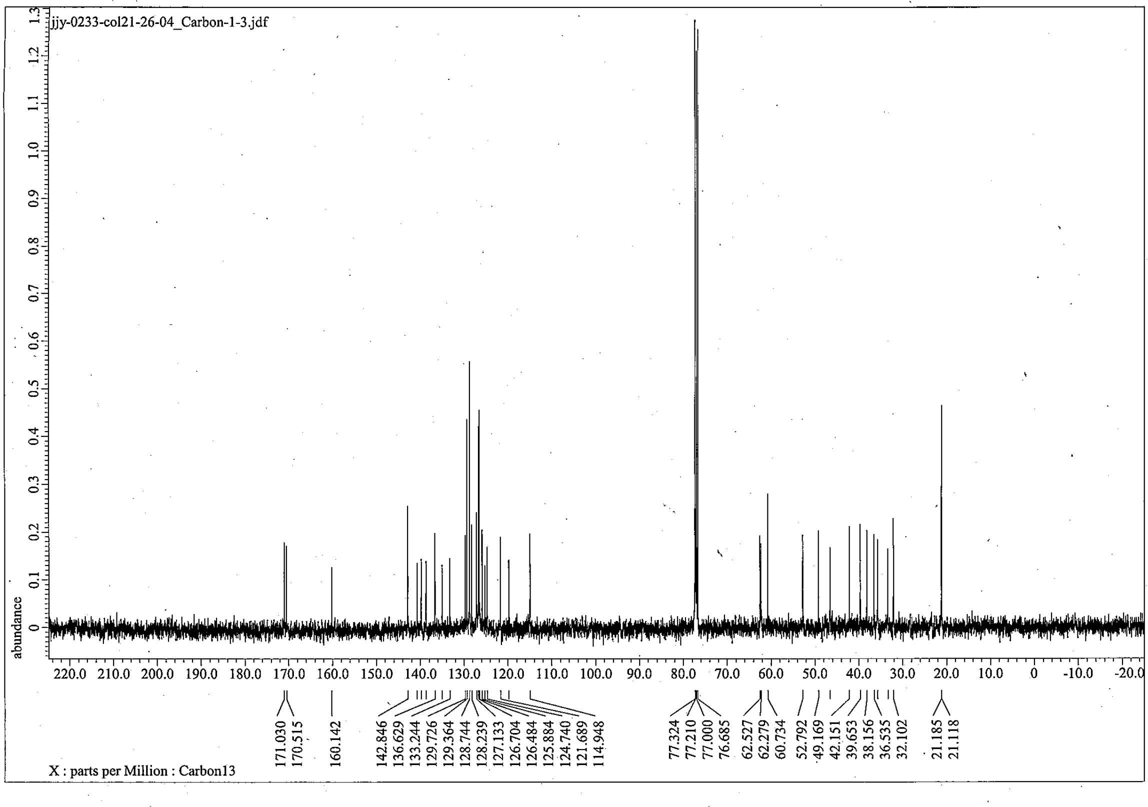
jjy-0233-col21-26-04_Carbon-1-3.jdf
X : parts per Million : Carbon13
abundance

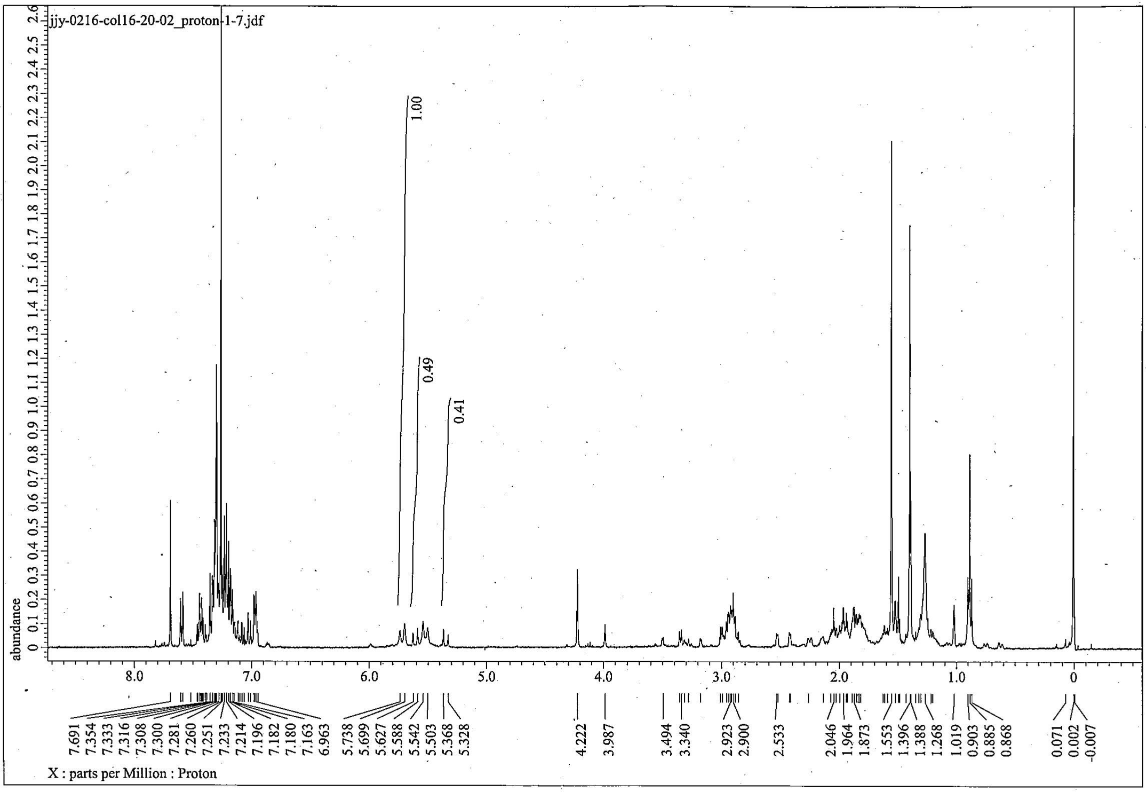

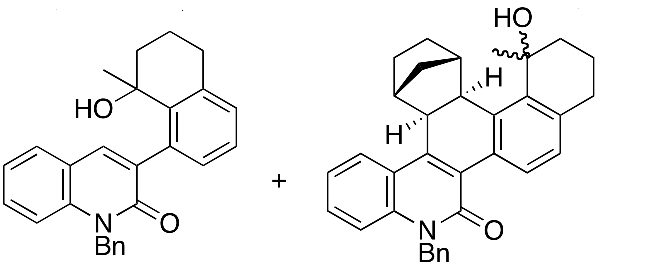

S3 **S4**

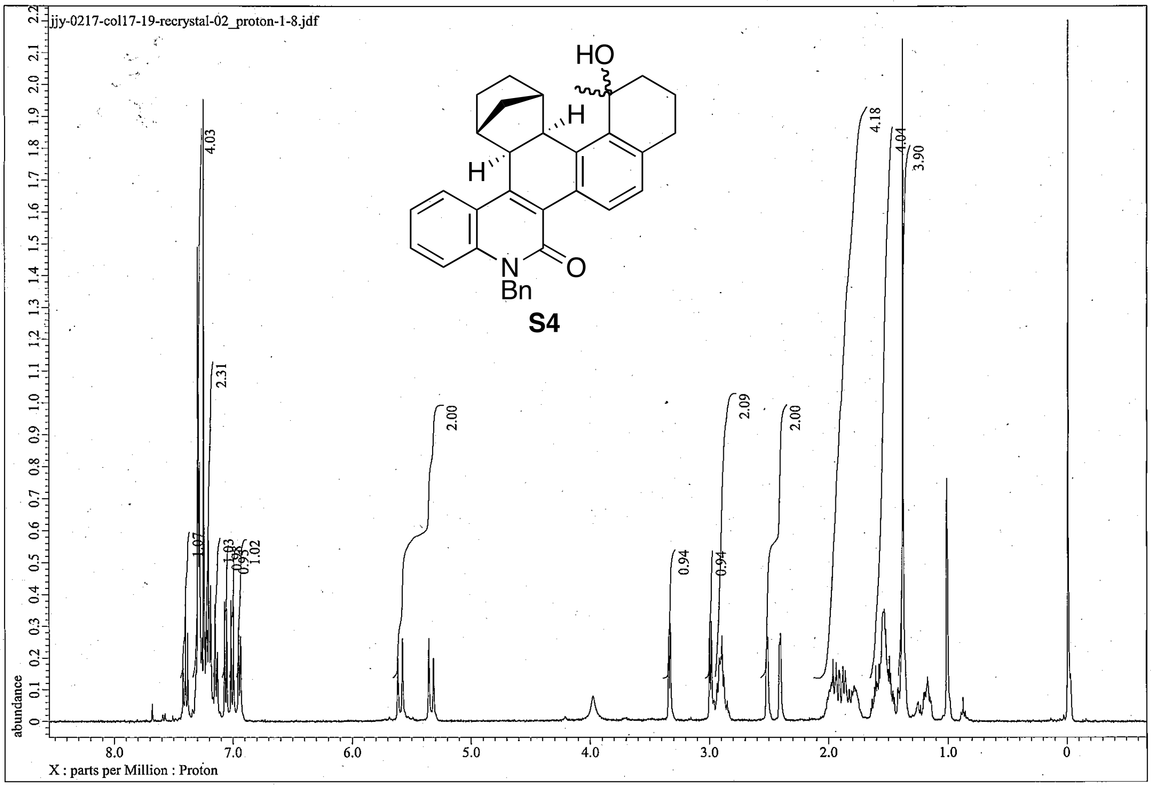
jjy-0217-col17-19-recrystal-02_proton-1-8.jdf
HO
H
H
N
O
Bn
S4
4.03
2.31
1.07
1.03
1.02
2.00
0.94
0.94
2.09
2.00
4.18
4.04
3.90
abundance
X : parts per Million : Proton

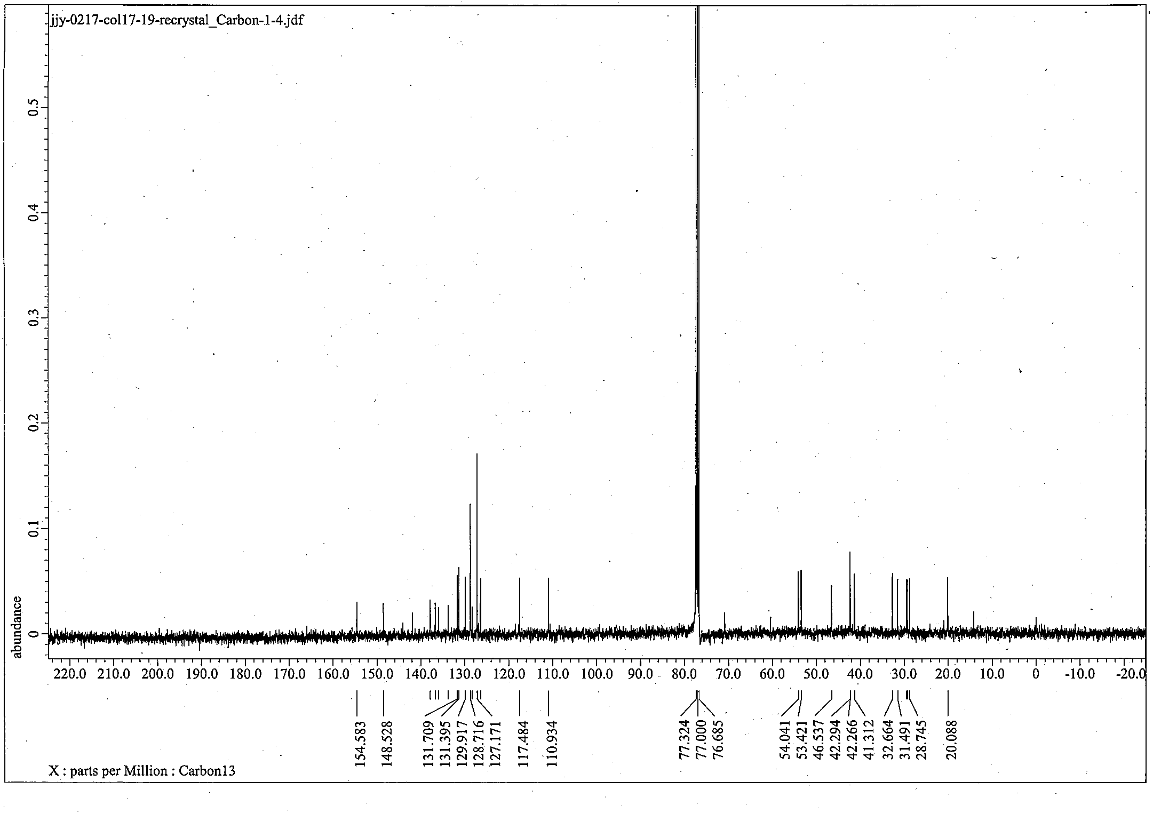
jjy-0217-col17-19-recrystal_Carbon-1-4.jdf
abundance
X : parts per Million : Carbon13
154.583
148.528
131.709
131.395
129.917
128.716
127.171
117.484
110.934
77.324
77.000
76.685
54.041
53.421
46.537
42.294
42.266
41.312
32.664
31.491
28.745
20.088

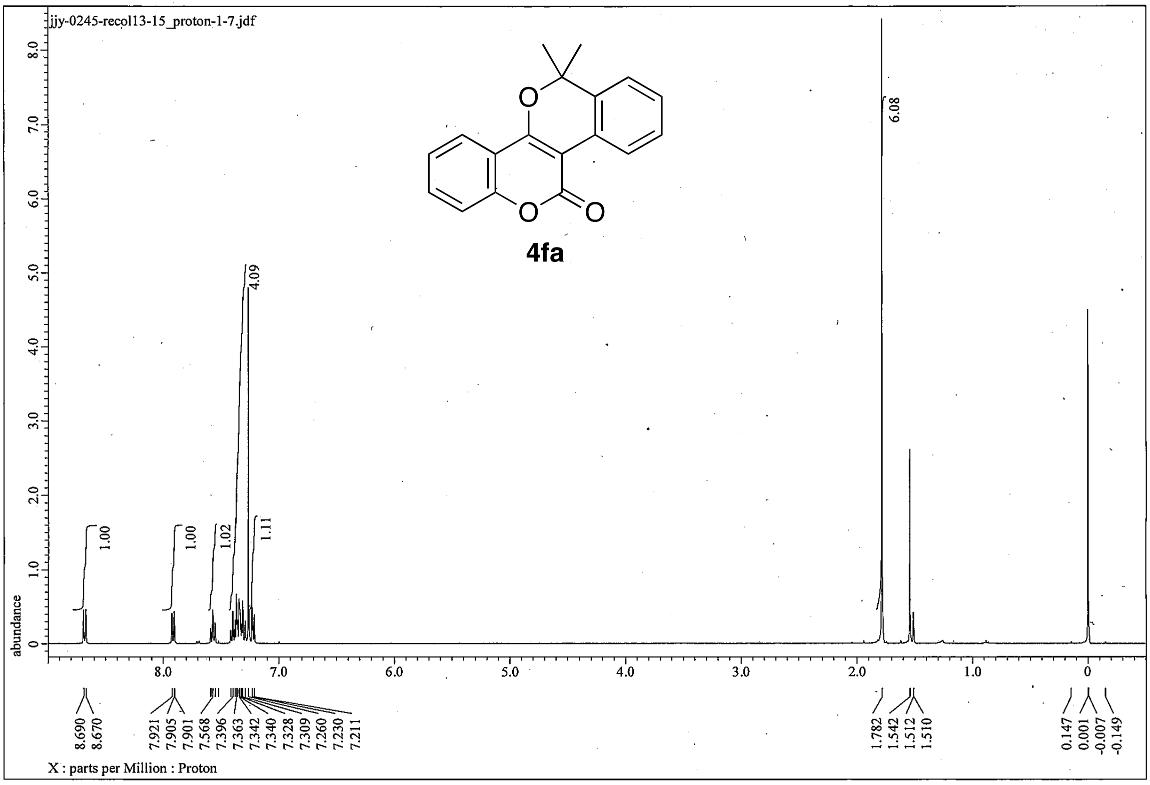
jjy-0245-recol13-15_proton-1-7.jdf
4fa
X : parts per Million : Proton
abundance

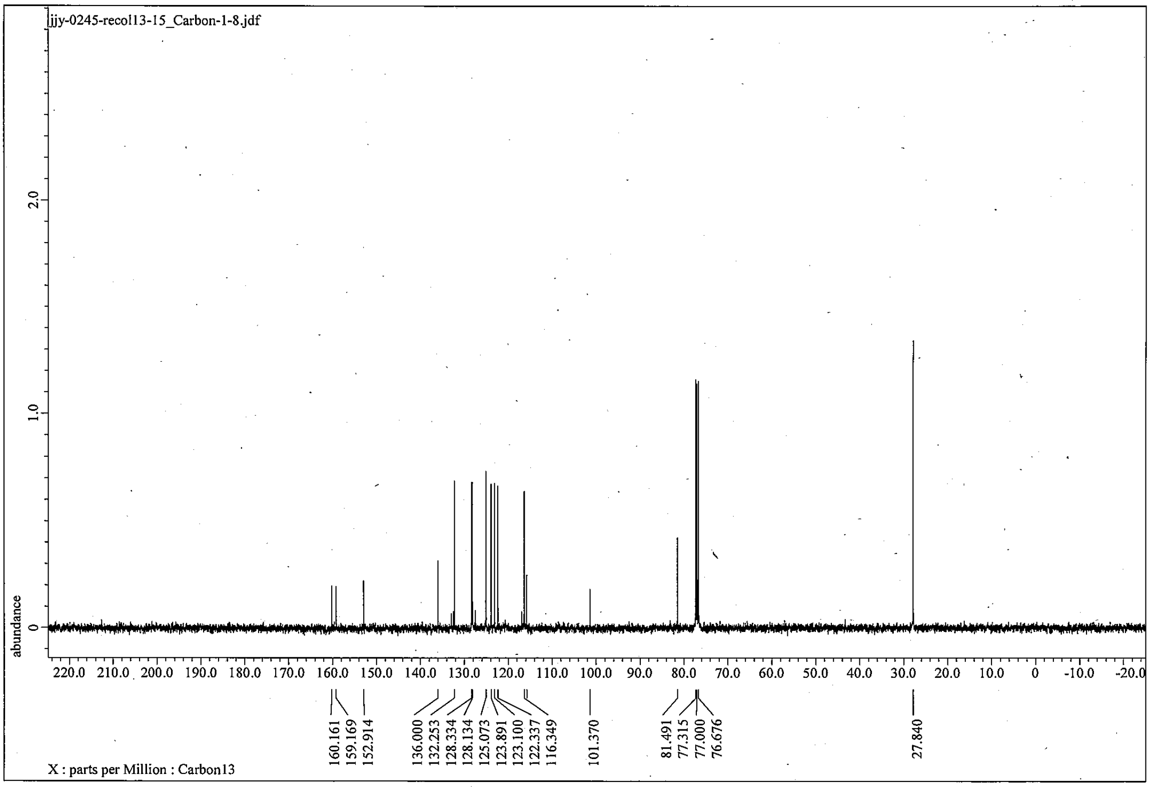
jjy-0245-recol13-15_Carbon-1-8.jdf
X : parts per Million : Carbon13
abundance
